# Genetic Associations and Architecture of Asthma-COPD Overlap

**DOI:** 10.1016/j.chest.2021.12.674

**Published:** 2022-01-31

**Authors:** Catherine John, Anna L. Guyatt, Nick Shrine, Richard Packer, Thorunn A. Olafsdottir, Jiangyuan Liu, Lystra P. Hayden, Su H. Chu, Jukka T. Koskela, Jian’an Luan, Xingnan Li, Natalie Terzikhan, Hanfei Xu, Traci M. Bartz, Hans Petersen, Shuguang Leng, Steven A. Belinsky, Aivaras Cepelis, Ana I. Hernández Cordero, Ma’en Obeidat, Gudmar Thorleifsson, Deborah A. Meyers, Eugene R. Bleecker, Lori C. Sakoda, Carlos Iribarren, Yohannes Tesfaigzi, Sina A. Gharib, Josée Dupuis, Guy Brusselle, Lies Lahousse, Victor E. Ortega, Ingileif Jonsdottir, Don D. Sin, Yohan Bossé, Maarten van den Berge, David Nickle, Jennifer K. Quint, Ian Sayers, Ian P. Hall, Claudia Langenberg, Samuli Ripatti, Tarja Laitinen, Ann C. Wu, Jessica Lasky-Su, Per Bakke, Amund Gulsvik, Craig P. Hersh, Caroline Hayward, Arnulf Langhammer, Ben Brumpton, Kari Stefansson, Michael H. Cho, Louise V. Wain, Martin D. Tobin

**Affiliations:** a Department of Health Sciences, University of Leicester, Leicester, England; b deCODE Genetics/Amgen, Reykjavik, Iceland; c Channing Division of Network Medicine, Brigham and Women’s Hospital and Harvard Medical School, Boston, MA; d Institute for Molecular Medicine Finland, University of Helsinki, Helsinki, Finland; e MRC Epidemiology Unit, University of Cambridge School of Clinical Medicine, Cambridge, England; f Division of Genetics, Genomics and Precision Medicine, Department of Medicine, University of Arizona, Tucson, AZ; g Department of Epidemiology, Erasmus Medical Center, Rotterdam, the Netherlands; h Department of Biostatistics, Boston University School of Public Health, Boston, MA; i Cardiovascular Health Research Unit, Department of Medicine and Department of Biostatistics, University of Washington, Seattle, WA; j Lovelace Respiratory Research Institute, Albuquerque, NM; k Division of Epidemiology, Biostatistics, and Preventive Medicine, Department of Internal Medicine, University of New Mexico, Albuquerque, NM; l Department of Public Health and Nursing, Faculty of Medicine and Health Sciences, Norwegian University of Science and Technology (NTNU), Levanger, Norway; m Centre for Heart Lung Innovation, University of British Columbia, Vancouver, BC, Canada; n Faculty of Medicine, School of Health Sciences, University of Iceland, Reykjavik, Iceland; o Division of Research, Kaiser Permanente of Northern California, Oakland, CA; p Brigham and Women’s Hospital and Harvard Medical School, Boston, MA; q Computational Medicine Core, Center for Lung Biology and UW Medicine Sleep Center, Medicine, University of Washington, Seattle, WA; r Department of Respiratory Medicine, Ghent University Hospital, Ghent, Belgium; s Department of Bioanalysis, Ghent University, Ghent, Belgium; t Department of Medicine, Wake Forest School of Medicine, Winston-Salem, NC; u Institut Universitaire de Cardiologie et de Pneumologie de Québec, Laval University, Quebec, QC, Canada; v Department of Pulmonology, University Medical Center Groningen, University of Groningen, and GRIAC Research Institute, Groningen, the Netherlands; w Global Health, University of Washington, Seattle, WA; x Gossamer Bio, San Diego, CA; y National Heart and Lung Institute, Imperial College London, London, UK; z Division of Respiratory Medicine and NIHR Nottingham Biomedical Research Centre, University of Nottingham, Nottingham, England; aa Biodiscovery Institute, University of Nottingham, Nottingham, England; bb Broad Institute of MIT and Harvard, Cambridge, MA; cc Division of Medicine, Department of Pulmonary Diseases, Turku University Hospital, Turku, Finland; dd Department of Pulmonary Diseases and Clinical Allergology, University of Turku, Turku, Finland; ee Center for Healthcare Research in Pediatrics (CHeRP) and PRecisiOn Medicine Translational Research (PROMoTeR) Center, Department of Population Medicine, Harvard Pilgrim Health Care Institute and Harvard Medical School, Boston, MA; ff Department of Clinical Science, University of Bergen, Bergen, Norway; gg MRC Human Genetics Unit, Institute of Genetics and Cancer, University of Edinburgh, Edinburgh, Scotland; hh K. G. Jebsen Center for Genetic Epidemiology, Department of Public Health and Nursing, Norwegian University of Science and Technology (NTNU), Trondheim, Norway; ii Clinic of Thoracic and Occupational Medicine, St. Olav’s Hospital, Trondheim University Hospital, Trondheim, Norway; jj Leicester NIHR Biomedical Research Centre, Leicester, England

**Keywords:** asthma, COPD, epidemiology, genome-wide association study, spirometry, ACO, asthma-COPD overlap, EAF, effect allele frequency, eQTL, expression quantitative trait locus, GWAS, genome-wide association study, LDSC, linkage disequilibrium score regression, *r*_g_, genetic correlation, SNP, single-nucleotide polymorphism

## Abstract

**Background:**

Some people have characteristics of both asthma and COPD (asthma-COPD overlap), and evidence suggests they experience worse outcomes than those with either condition alone.

**Research Question:**

What is the genetic architecture of asthma-COPD overlap, and do the determinants of risk for asthma-COPD overlap differ from those for COPD or asthma?

**Study Design and Methods:**

We conducted a genome-wide association study in 8,068 asthma-COPD overlap case subjects and 40,360 control subjects without asthma or COPD of European ancestry in UK Biobank (stage 1). We followed up promising signals (*P* < 5 × 10^–6^) that remained associated in analyses comparing (1) asthma-COPD overlap vs asthma-only control subjects, and (2) asthma-COPD overlap vs COPD-only control subjects. These variants were analyzed in 12 independent cohorts (stage 2).

**Results:**

We selected 31 independent variants for further investigation in stage 2, and discovered eight novel signals (*P* < 5 × 10^–8^) for asthma-COPD overlap (meta-analysis of stage 1 and 2 studies). These signals suggest a spectrum of shared genetic influences, some predominantly influencing asthma (*FAM105A*, *GLB1*, *PHB*, *TSLP*), others predominantly influencing fixed airflow obstruction (*IL17RD*, *C5orf56*, *HLA-DQB1*). One intergenic signal on chromosome 5 had not been previously associated with asthma, COPD, or lung function. Subgroup analyses suggested that associations at these eight signals were not driven by smoking or age at asthma diagnosis, and in phenome-wide scans, eosinophil counts, atopy, and asthma traits were prominent.

**Interpretation:**

We identified eight signals for asthma-COPD overlap, which may represent loci that predispose to type 2 inflammation, and serious long-term consequences of asthma.

## Graphical Abstract

**Figure:**
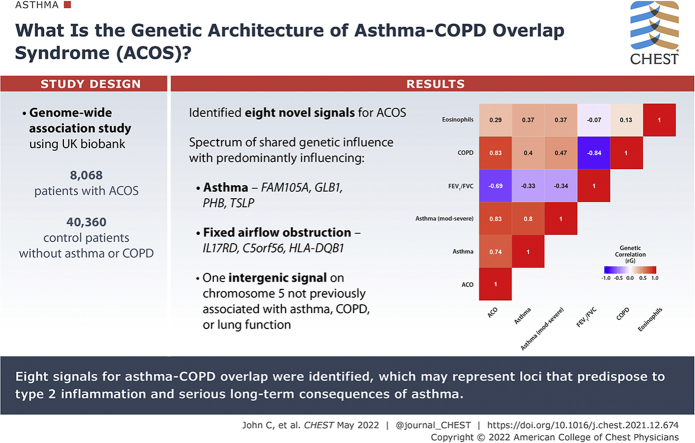

FOR EDITORIAL COMMENT, SEE PAGE 1125

**Take-home Points**

**Study Question:** What are the genetic determinants of risk for asthma-COPD overlap, and how do these differ from those for COPD or asthma?

**Results:** We discovered eight novel signals for asthma-COPD overlap in a meta-analysis of 12,369 case subjects and 88,969 control subjects; most signals suggested a spectrum of shared genetic influences on asthma, COPD, or lung function, and in phenome-wide scans of these signals, eosinophil counts, atopy, and asthma traits were prominent.

**Interpretation:** We identified eight signals for asthma-COPD overlap, not driven by smoking or age at asthma diagnosis, which may represent loci that predispose to type 2 inflammation, and serious long-term consequences of asthma.

Asthma and COPD have substantial global impacts.^1^ They are heterogeneous conditions^2^, ^3^, ^4^ that share some common features, including airflow obstruction with differing degrees of reversibility. Inflammatory processes are important in both conditions, and cytokine profiles identify both distinct and overlapping groups of patients.^5^ People with characteristics of both conditions have until recently been referred to as having “asthma-COPD overlap” (ACO),^4^ and a number of studies have suggested that such patients have significantly worse outcomes than those with either condition alone.^6^, ^7^, ^8^, ^9^, ^10^, ^11^, ^12^, ^13^ Guidelines emphasize that asthma and COPD are different conditions, but may coexist in the same patient.^14^ People with features of both diseases risk being excluded from research that might provide evidence about the most effective treatment strategies.^3^

Environmental risk factors—notably smoking in COPD—are central, but genetics also plays an important role in both asthma and COPD,^15^, ^16^, ^17^ and it has long been hypothesized that there may be a shared, underlying genetic predisposition to both diseases.^2^^,^^18^ Genome-wide association studies (GWASs) examine variants across the genome in an unbiased manner, to identify variant-trait associations that inform our understanding of disease biology and potential treatment strategies. GWASs have identified many loci associated with asthma or COPD in European populations (e-Appendix 1). The genetic correlation (*r*_g_) between asthma and COPD is 0.38 (*P* = 6.2 × 10^−5^), suggesting a shared genetic etiology.^19^ A GWAS of ACO compared with COPD alone (n = 3,570) did not identify any variants associated at the conventional threshold,^8^ and a meta-analysis of an asthma and COPD GWAS found one association, driven by COPD.^20^ Eighteen loci outside the HLA (human leukocyte antigen) region have been identified as associated with both asthma and lung function/COPD at *P* < 5 × 10^–8^, but have not been specifically described as ACO loci.

Notwithstanding the controversies of changing terminology for people with both asthma and COPD, we refer to this case status as “ACO.” Improved knowledge of genetic variants associated with coexisting asthma and COPD would contribute to our understanding of underlying molecular pathways, and potentially inform diagnostic terminology and specific management strategies for those with coexisting asthma and COPD.

Accordingly, using spirometry, self-report, and electronic health care record data to define case subjects with both asthma and COPD (ACO) and suitable control subjects, we undertook the largest GWAS of coexisting asthma and COPD to date, including up to 12,369 case subjects and 88,969 control subjects, in a two-stage design incorporating 13 studies.

## Study Design and Methods

### Stage 1

The data source for this study was UK Biobank.^21^ Eligibility criteria, genotyping, and quality control are described in e-Appendix 1. A total of 321,057 people and 37 million single-nucleotide polymorphisms (SNPs) were included.

We defined cases of ACO if patients had self-reported asthma (see e-Appendix 1) and FEV_1_/FVC < 0.7 with Global Initiative for Chronic Obstructive Lung Disease (GOLD) 2+ airflow limitation (FEV_1_ < 80% predicted). Case subjects who reported alpha-1 antitrypsin deficiency were excluded. Control subjects reported no asthma or COPD (e-Appendix 1), and had FEV_1_ ≥ 80% predicted and FEV_1_/FVC > 0.7. Five control subjects were randomly selected for each case. Case subjects and control subjects were unrelated (second degree or closer). Two additional control sets were defined for signal prioritization: people with asthma but without COPD, and people with COPD but without asthma. Asthma and COPD were defined as above.

Association testing was undertaken in SNPTEST, version 2.5.2 (“score” option),^22^ under an additive model. Age, sex, smoking status (ever/never), genotyping array, and 10 principal components were included as covariates. Variants were filtered on the basis of minor allele frequency (MAF) > 0.01 and imputation quality (INFO) > 0.5. *P* values and SEs were adjusted for the linkage disequilibrium score regression (LDSC) intercept^23^ (e-Fig 1).

In stage 1, we defined distinct signals passing a *P* value threshold of *P* < 5 × 10^–6^. We defined regions of association around the most strongly associated variant (sentinel variant) ± 1 Mb. To identify distinct signals, and additional signals within the regions described above, conditional analyses were undertaken with GCTA-COJO^24^ (e-Appendix 1, e-Fig 2).

Two further “signal prioritization” analyses were undertaken to ascertain the extent to which signals were driven by association with COPD and/or asthma alone. These included the same cases as the primary analysis, plus the two additional control sets described above. Variants were selected for follow-up in stage 2 if they were associated at *P* < 5 × 10^–6^ in the main stage 1 analysis and at *P* < .01 in both signal prioritization analyses.

### Stage 2 and Joint Analysis

SNPs identified in stage 1 signal prioritization analyses were tested for association in 12 independent studies of European ancestry (up to 4,301 case subjects and 48,609 control subjects, in CHS [Cardiovascular Health Study], COPDGene [Genetic Epidemiology of COPD], deCODE, ECLIPSE [Evaluation of COPD Longitudinally to Identify Predictive Surrogate Endpoints], EPIC-Norfolk [European Prospective Investigation of Cancer in Norfolk], FHS [Framingham Heart Study], Generation Scotland, GenKOLS [Genetics of Chronic Obstructive Lung Disease Study], Trøndelag Health Study [HUNT], Lovelace Smokers' Cohort, Rotterdam Study, and SPIROMICS [Subpopulations and Intermediate Outcome Measures in COPD Study]) and one cohort of participants of self-reported African American ethnicity (COPDGene; 297 case subjects, 1,335 control subjects) (e-Appendix 1, e-Tables 1, 2).

Case subjects had both asthma and COPD. Asthma was defined as any lifetime self-report of asthma, or asthma diagnosis in the health care record, including billing codes (see e-Appendix 1 for further details and validation).^25^ All case subjects had spirometry results indicating FEV_1_/FVC < 0.7, and FEV_1_ < 80% predicted. All control subjects had FEV_1_/FVC > 0.7, FEV_1_ ≥ 80% predicted, and no asthma diagnosis. When possible, studies excluded people with alpha-1 antitrypsin deficiency.

Details of statistical analysis in stage 2 studies are presented in e-Appendix 1 (e-Table 3). Results were combined across stage 2 studies, using fixed-effect meta-analysis. Heterogeneity was assessed using the *I*^2^ statistic. We combined these results with those from UK Biobank (stage 1).

We performed a sensitivity analysis to assess whether the way COPD was defined changed our stage 2 results (e-Appendix 1).

To assess whether associations with our stage 1 signals changed according to age at asthma diagnosis, we divided case subjects into those who self-reported their age at asthma diagnosis as < 12 years, and > 25 years.^26^ We then repeated the association tests in UK Biobank. In addition, we repeated association testing after stratifying our sample into ever/never smokers.

### Definition of Top Signals for Bioinformatic Analyses

We undertook bioinformatic analyses on ACO signals reaching *P* < 5 × 10^–8^ in the joint analysis of stages 1 and 2, and which also had a lower *P* value in the joint analysis than in UK Biobank (stage 1) alone or had *P* < .05 in stage 2. For each of these, we identified the set of SNPs that was 99% likely to contain the causal variant (“99% credible set”), assuming that the causal variant was included in the data set (e-Appendix 1).^27^ For bioinformatic analysis methods, see e-Appendix 1.

Using LDSC,^28^ we computed genetic correlations between ACO (stage 1 results), asthma,^29^ moderate-severe asthma,^30^ COPD,^31^ eosinophil counts,^32^ and FEV_1_/FVC.^33^ We also computed genetic correlations between ACO and atopic, autoimmune, and smoking behavior traits.^34^

### Approvals

The research was conducted using UK Biobank, under application 648. UK Biobank has ethical approval from the UK National Health Service National Research Ethics Service (11/NW/0382). All included studies were approved by the appropriate research ethics committee or institutional review board (e-Appendix 1). All participants gave informed consent.

## Results

In stage 1, 8,068 ACO case subjects were selected from UK Biobank, and 40,360 as healthy control subjects free of asthma and COPD. For signal prioritization analyses, another 16,762 people were selected as control subjects with COPD alone (without asthma), and 26,815 as control subjects with asthma alone (without COPD). Descriptive statistics for case subjects and control subjects are presented in Table 1. ACO case subjects were slightly older than healthy control subjects, and included more men and ever smokers.

**Table 1:** Descriptive Characteristics of Case Subjects and Control Subjects Included in Stage 1 (UK Biobank Primary and Signal Prioritization Analyses)

Characteristic	ACO Case Subjects (n = 8,068)	Healthy Control Subjects (n = 40,360)	Control Subjects With COPD But No Asthma (n = 16,762)	Control Subjects With Asthma But No COPD (n = 26,815)
Age at recruitment, median (IQR), y	60 (53-65)	57 (49-63)	62 (56-65)	55 (48-61)
Sex, No. (%)				
Male	4,179 (51.8%)	17,598 (43.6%)	9,147 (54.6%)	9,703 (36.2%)
Female	3,889 (48.2%)	22,762 (56.4%)	7,615 (45.4%)	17,112 (63.8%)
Smoking status, No. (%)				
Ever smoked	4,367 (54.1%)	17,316 (42.9%)	11,752 (70.1%)	11,231 (41.9%)
Never smoked	3,701 (45.9%)	23,044 (57.1%)	5,010 (29.9%)	15,584 (58.1%)
Pack-years of smoking, median (IQR)^a^	25.5 (13.5-39.5)	15.8 (8.3-26.4)	32.0 (19.0-45.5)	16.5 (8.5-28.1)
Allergic rhinitis (including hay fever) or eczema, No. (%)				
Yes	3,325 (41.2%)	8,468 (21.0%)	2,691 (16.1%)	13,010 (48.5%)
No	4,743 (58.8%)	31,892 (79.0%)	14,071 (83.9%)	13,805 (51.5%)
Eosinophil count (median, IQR),^b^ × 10^9^ cells/L	0.20 (0.13-0.32)	0.13 (0.08-0.20)	0.16 (0.10-0.24)	0.21 (0.11-0.27)
Lung function, median (IQR)				
FEV_1_/FVC	0.63 (0.58-0.67)	0.78 (0.75-0.81)	0.65 (0.61-0.68)	0.77 (0.74-0.80)
% predicted FEV_1_	66.1% (56.5%-73.3%)	97.3% (89.9%-105.6%)	68.7% (60.0%-74.8%)	90.8% (81.6%-100.0%)

ACO = asthma-COPD overlap; IQR = interquartile range.

a In ever smokers with nonmissing data, 3,270 of 4,367 case subjects, 11,196 of 17,316 main control subjects, 9,672 of 11,752 COPD-not asthma control subjects, 7,443 of 11,231 asthma-not COPD control subjects

b In those with nonmissing data after cleaning as per Astle et al,^32^ n = 7,666 case subjects, n = 38,259 main control subjects, n = 15,845 COPD-not asthma control subjects, n = 25,292 asthma-not COPD control subjects.

After filtering on MAF and INFO, 7,693,381 variants were analyzed. The LDSC intercept was 1.018, suggesting that results were not strongly inflated due to population structure (e-Fig 1).^28^

### ACO Association Signals

**Figure 1:**
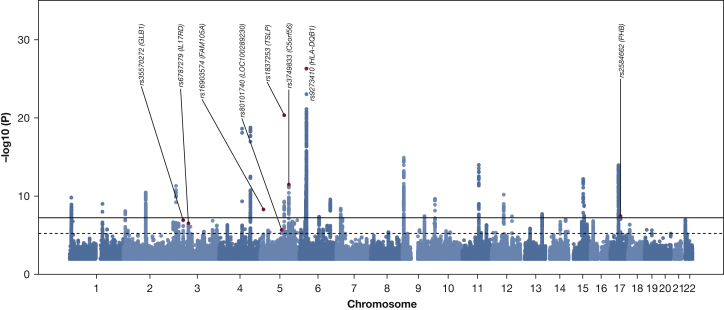
Manhattan plot of association results for asthma-COPD overlap in stage 1 (UK Biobank). The *x* axis shows genomic location by chromosome, the *y* axis shows the –log_10_*P* value, corrected for the intercept of linkage disequilibrium score regression (1.018). The eight top signals (from joint analysis) are highlighted in red, and labeled with rsIDs (reference SNP [single-nucleotide polymorphism] ID numbers). The black line indicates *P* = 5 × 10^–8^ (commonly known as genome-wide significance), and the dotted line corresponds to *P* = 5 × 10^–6^ (genome-wide suggestive threshold). A quantile-quantile plot is shown in e-Figure 1. For further details on the eight SNPs shown here, see also Table 2.

In stage 1, there were 83 distinct signals at *P* < 5 × 10^–6^ (Fig 1,^32^ e-Appendix 1, e-Fig 2 for the signal selection; e-Table 4 for results). Of these, 31 retained significance (*P* < .01) in signal prioritization analyses comparing ACO case subjects separately with either COPD case subjects or asthma case subjects, to determine whether signals were driven by asthma or COPD alone (e-Table 4). In stage 2, comprising 12 independent cohorts (4,301 case subjects, 48,609 control subjects) (e-Tables 1, 2), 26 of 31 signals had a direction of effect concordant with stage 1 (e-Table 5), and the median value for heterogeneity (*I*^2^) across these signals was 15%. Although the sample size of participants of African American ethnicity was small (297 case subjects, 1,335 control subjects) and CIs were broad, 22 of 31 signals had a direction of effect consistent with the European ancestry studies (e-Table 5).

Results for the stage 2 sensitivity analysis (9,638 case subjects and 128,273 control subjects from 15 studies), in which COPD was defined either by available spirometry or, alternatively, by electronic health care record diagnoses (e-Appendix 1), are in e-Table 6.

### Subgroup Analyses

Effect sizes for the 31 signals among case subjects with childhood-onset asthma were highly correlated with those among people with adult-onset asthma (*R* = 0.883) (e-Table 7, e-Fig 3). Effect sizes in ever and never smokers were also closely correlated (*R* = 0.911) (e-Table 7, e-Fig 4).

### Eight Top Signals for ACO Defined From Joint Analysis

After meta-analysis combining stage 1 and stage 2, 13 signals were genome-wide significant (*P* < 5 × 10^–8^) (e-Table 4; e-Fig 2 for flow diagram). Of these, eight either had a lower *P* value in the joint analysis than in stage 1 alone, or *P* < .05 in stage 2 studies alone (Table 2, e-Figs 5, 6). None of these eight signals were previously reported as associated specifically with ACO.^8^

**Table 2:** Eight Genome-Wide Signals for Asthma-COPD Overlap

rsID	Chr:Pos (Effect/Noneffect Allele)	Nearest Gene	Location	EAF	Stage 1 (UK Biobank; Case Subjects, 8,068; Control Subjects, 40,360)		Stage 2 (12 Independent Studies; Case Subjects, 4,301; Control Subjects, 48,609)^a^		Joint Analysis of Stage 1 and Stage 2	
					OR (95% CI)	*P* Value	OR (95% CI)	*P* Value	OR (95% CI)	*P* Value
rs80101740	5:98471135 (C/A)	*LOC100289230*	Intergenic	0.015	1.44 (1.24-1.68)^a^	1.87 × 10^–6^^a^	1.37 (1.10-1.71)	5.49 × 10^–3^	1.42 (1.25-1.61)	3.72 × 10^–8^
rs35570272	3:33047662 (T/G)	*GLB1*	Intronic	0.398	1.11 (1.07-1.15)	1.06 × 10^–7^	1.08 (1.02-1.14)	4.67 × 10^–3^	1.10 (1.06-1.13)	2.44 × 10^–9^
rs16903574	5:14610309 (G/C)	*FAM105A*	Exonic	0.077	1.23 (1.15-1.32)	4.47 × 10^–9^	1.13 (1.03-1.25)	9.96 × 10^–3^	1.20 (1.13-1.27)	3.8 × 10^–10^
rs2584662	17:47470487 (C/A)	*PHB*	Intergenic	0.42	0.90 (0.86-0.94)	3.20 × 10^–8^	0.95 (0.90-1.00)	5.89 × 10^–2^	0.92 (0.89-0.95)	2.21 × 10^–8^
rs1837253	5:110401872 (C/T)	*TSLP*	Intergenic	0.739	1.22 (1.17-1.27)	4.22 × 10^–21^	1.06 (1.00-1.12)	4.44 × 10^–2^	1.16 (1.12-1.20)	1.53 × 10^–18^
rs6787279	3:57163751 (C/T)	*IL17RD*	Intronic	0.169	0.88 (0.84-0.92)	2.69 × 10^–7^	0.91 (0.85-0.97)	6.51 × 10^–3^	0.89 (0.86-0.93)	7.87 × 10^–9^
rs9273410	6:32627250 (A/C)	*HLA-DQB1*	UTR3	0.445	1.24 (1.19-1.29)	4.37 × 10^–27^	1.11 (1.05-1.18)	6.42 × 10^–4^	1.20 (1.16-1.24)	9.19 × 10^–28^
rs3749833	5:131799626 (C/T)	*C5orf56*	ncRNA intronic	0.263	1.16 (1.11-1.21)	3.10 × 10^–12^	1.06 (1.00-1.12)	4.21 × 10^–2^	1.12 (1.09-1.16)	9.37 × 10^–12^

Variants were annotated with nearest gene and type of region, using ANNOVAR software (and genome build hg19). OR, 95% CI, and *P* value were all calculated by score testing. The Firth test for rs80101740 gave OR, 1.40 (95% CI, 1.22-1.60) and *P* = 1.56 × 10^–6^. Chr:Pos = chromosome:position; EAF = effect allele frequency; ncRNA = noncoding RNA; rsID = reference SNP (single-nucleotide polymorphism) ID number; UTR3 = 3' (three prime) untranslated region.

a Stage 2 studies: CHS (Cardiovascular Health Study), COPDGene (Genetic Epidemiology of COPD), deCODE, ECLIPSE (Evaluation of COPD Longitudinally to Identify Predictive Surrogate Endpoints), EPIC-Norfolk (European Prospective Investigation of Cancer in Norfolk), FHS (Framingham Heart Study), Generation Scotland, GenKOLS (Genetics of Chronic Obstructive Lung Disease Study), Trøndelag Health Study (HUNT), Lovelace Smokers' Cohort, Rotterdam Study, SPIROMICS (Subpopulations and Intermediate Outcome Measures in COPD Study).

For the novel intergenic ACO signal on chromosome 5 (rs80101740, effect allele frequency [EAF], 0.015; OR, 1.42; *P* = 3.72 × 10^–8^) (e-Table 5), which has not been previously associated with asthma, lung function, or COPD, the sentinel SNP had the largest posterior probability (0.77) of being the true causal variant, assuming the causal variant was genotyped/imputed (e-Table 8). There was no evidence of colocalization with expression quantitative trait locus (eQTL) signals at this locus (e-Tables 9, 10), and no chromatin interactions were identified.

Four of our novel signals for ACO were previously reported for asthma but not COPD/lung function.^35^, ^36^, ^37^ For rs35570272 in *GLB1* (OR, 1.10; EAF, 0.398; *P* = 2.44 × 10^–9^), there were 11 SNPs in the credible set, and the intronic sentinel SNP had the highest posterior probability (0.655). There were significant chromatin interactions nearby in *GLB1* in fetal lung fibroblasts. *GLB1* encodes the β-galactosidase enzyme involved in lysosomal function, and an elastin-binding protein involved in extracellular elastic fiber formation. Two SNPs (both with a posterior probability of ∼0.13) in the 99% credible set, rs7646283 and rs34064757, were eQTLs for the gene encoding cartilage-associated protein (*CRTAP*) in lung (e-Table 9), implicated in bone development and osteogenesis imperfecta.

Another signal (previously reported for asthma)^26^^,^^35^ was rs16903574 (EAF, 0.077; OR, 1.20; *P* = 3.8 × 10^–10^), a missense variant in *FAM105A*, deleterious according to its combined annotation-dependent depletion (CADD) score (22.6).^38^ *FAM105A* encodes a pseudoenzyme, possibly involved in protein-protein interactions.^39^ This sentinel had a posterior probability of 0.99. A previous study in asthma predicted *FAM105A* as the target based on chromatin interactions and correlation between enhancer epigenetic marks and gene expression, although we did not identify any eQTL evidence in lung or whole blood.^35^ We also identified a highly significant chromatin interaction in fetal lung fibroblasts overlapping *FAM105A* and another nearby gene (*TRIO*), but not in adult lung.

An intergenic signal between *PHB* and *ZNF652* (rs2584662) (EAF, 0.42; OR, 0.92; *P* = 2.21 × 10^–8^) was previously associated with asthma and reported as a blood eQTL for *GNGT2* (implicated in NF-κB activation),^29^^,^^35^ although we did not identify this in our eQTL analysis. In our analysis, eight SNPs were in the credible set (posterior probabilities all ≤ 0.2). Hi-C data suggested a significant chromatin interaction in *ZNF652*, with another, less significant peak close to *GNGT2*. Nearby loci in *ZNF652* have previously been associated with asthma/allergic disease and moderate-to-severe asthma.^35^

We also identified rs1837253, an intergenic signal near *TSLP* (EAF, 0.739; OR, 1.16; *P* = 1.53 × 10^–18^), with a posterior probability of 1, that is, the only variant in the credible set. No eQTL evidence was identified. There were highly significant chromatin interactions with *SLC25A46* in fetal lung fibroblasts and in adult lung tissue, and with a region between *TSLP* and *SLC25A46* in fetal lung fibroblasts only. The gene encoding thymic stromal lymphopoietin (*TSLP*) was implicated in asthma and allergic disease before the GWAS era,^40^ and an anti-TSLP antibody has been trialled in allergic asthma.^41^

Another signal, rs6787279 in *IL17RD* (EAF, 0.169; OR, 0.89; *P* = 7.87 × 10^–9^), has been previously reported for lung function and COPD.^31^^,^^42^ There were 55 variants in the credible set, all with posterior probability ≤ 0.12, meaning it is not yet possible to fine-map this signal confidently. One SNP in the credible set was exonic and possibly damaging (rs17057718), but the posterior probability was only 0.012. Multiple SNPs at this locus were eQTLs for *IL17RD* in lung, with the ACO risk allele corresponding to decreased *IL17RD* expression. IL17RD is in the IL-17 signaling pathway, which is implicated in asthma,^43^ and in COPD pathogenesis,^44^^,^^45^ potentially by mediating effects of cigarette smoke.

Two ACO signals have previously been reported separately for both asthma and lung function or COPD: rs9273410 in *HLA-DQB1* (EAF, 0.445; OR, 1.20; *P* = 9.19 × 10^–28^) and rs3749833 in *C5orf56* (EAF, 0.263; OR, 1.12; *P* = 9.37 × 10^–12^). *HLA-DQB1* encodes a major histocompatibility complex type II molecule involved in antigen presentation. *HLA-DQB1* alleles are associated with numerous inflammatory and autoimmune diseases. In our analysis, the sentinel was the only SNP in the credible set. For lung function, an amino acid change in the gene product HLA-DQβ1 has been identified as the main driver of signals in the major histocompatibility complex region.^33^ Analyses in asthma have identified *HLA-DQA1* as the likely driver gene.^35^

*C5orf56* is located on a cytokine gene cluster on chromosome 5, including *IL3*, *IL4*, and *IL5*. Several interleukins in this region have been considered as therapeutic targets in asthma. In severe eosinophilic asthma, the anti-IL-5 monoclonal antibodies mepolizumab and reslizumab reduce exacerbations and improve quality of life.^46^, ^47^, ^48^ SNPs in the credible set were eQTLs in lung and/or blood for *SLC22A5*, *AC116366*.6, *RAD50*, and a noncoding Y RNA. *SLC22A4* has been identified as the most likely candidate gene for the lung function association.^33^ The gene products of *SLC22A4* and *SLC22A5* are involved in bronchial uptake of bronchodilators and anticholinergic drugs.^49^ An analysis in asthma predicted *C5orf56* (which encodes the interferon regulatory factor 1 antisense RNA, *IRF1-AS1*) as the causal gene.^35^

In our phenome-wide scan, all ACO loci previously associated with asthma showed association with blood cell counts, particularly eosinophils and neutrophils, and atopic traits (e-Table 11). The *HLA* locus was associated with a wide range of autoimmune/inflammatory traits. Another locus (rs2584662, near *PHB* and *ZNF652*) was associated with anthropometric traits, cardiovascular phenotypes, and chronic diseases/multimorbidity, whereas rs3749833 (near *C5orf56*) was associated with anthropometric traits and inflammatory bowel disease. SNPs in the credible set for the intergenic chromosome 5 signal (rs80101740) were associated with cardiovascular and a range of other traits.

### ACO Shares Genetic Architecture With Other Traits

**Figure 2:**
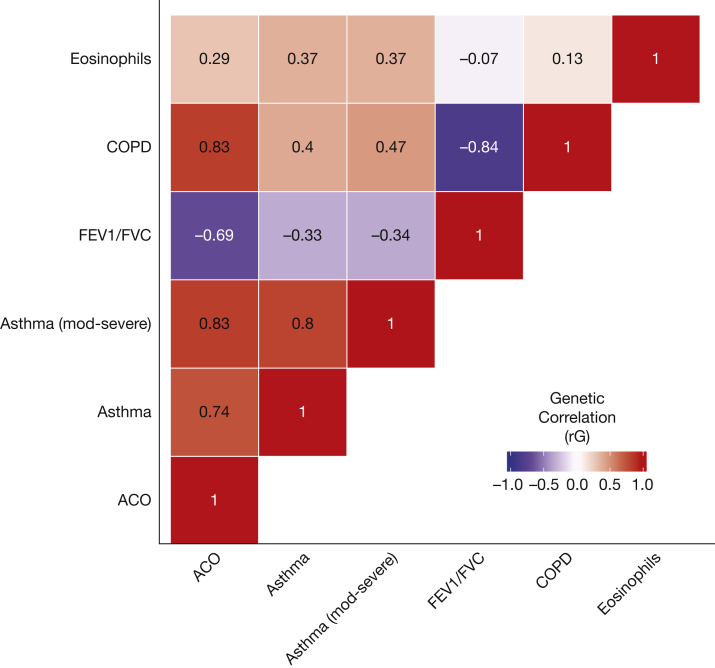
Genetic correlations between asthma-COPD overlap (ACO) and asthma, moderate-severe asthma, COPD, FEV_1_/FVC, and blood eosinophil counts. Genetic correlations were computed by linkage disequilibrium score regression. The annotation in each tile represents the magnitude of the genetic correlation estimate (rG), and intensity is proportional to the magnitude of effect. Note that for FEV_1_/FVC, a negative correlation shows that the other trait is associated with reduced FEV_1_/FVC (reduced FEV_1_/FVC implies worse lung function). Data sets used: ACO = current discovery results from UK Biobank; Asthma = GWAS results from Demenais et al^29^; Asthma (moderate-severe) = genome-wide association study (GWAS) of asthma by Shrine et al^30^; COPD = GWAS of COPD by Sakornsakolpat et al^31^; FEV_1_/FVC = GWAS of FEV_1_/FVC (UK Biobank and SpiroMeta) by Shrine et al^33^; Eosinophils = blood eosinophil counts published by Astle et al.^32^

We observed genetic correlations (*r*_g_) of broadly similar magnitude between ACO and COPD (*r*_g_ = 0.828; *P* = 3.19 × 10^–299^), ACO and asthma (*r*_g_ = 0.743; *P* = 6.18 × 10^−44^), and ACO and FEV_1_/FVC (*r*_g_ = –0.692; *P* = 7.48 × 10^−33^) (Fig 2, e-Table 12). The genetic correlation (*r*_g_) between asthma and FEV_1_/FVC was –0.333 (*P* = 8.71 × 10^−7^) (ie, increased asthma risk was correlated with lower FEV_1_/FVC). Blood eosinophil counts were moderately correlated with ACO (*r*_g_ = 0.292; *P* = 4.87 × 10^−11^), similar in magnitude to the correlation of eosinophils with asthma (*r*_g_ = 0.371; *P* = 3.15 × 10^−7^), whereas the correlations of eosinophils with FEV_1_/FVC (*r*_g_ = –0.070; *P* = .002) and COPD (*r*_g_ = 0.130; *P* = 4.83 × 10^–6^) were smaller. We additionally computed genetic correlations between ACO and 16 autoimmune traits, and between ACO and smoking behavior (*r*_g_ = 0.046, *P* = .417) (e-Table 12). After asthma, the next strongest correlation was with eczema (*r*_g_ = 0.255, *P* = .004), then multiple sclerosis (*r*_g_ = 0.323, *P* = .011).

## Discussion

We conducted the largest GWAS of ACO to date, and identified 83 independent signals associated at *P* < 5 × 10^–6^ in stage 1. After excluding variants associated with asthma only or COPD only, we studied 31 variants in stage 2, with eight distinct signals for ACO showing genome-wide significance (*P* < 5 × 10^–8^) in a stage 1 and stage 2 meta-analysis.

Our study contributes to understanding of the genetic architecture of ACO. We showed strong genetic correlation between ACO and COPD/lung function, and between ACO and asthma, especially moderate-severe asthma. Furthermore, we showed that the genetic correlation of blood eosinophil counts with ACO was more similar in magnitude to the genetic correlation of eosinophils with asthma than of eosinophils with FEV_1_/FVC and COPD. Increased eosinophils are associated with asthma and COPD exacerbations,^50^, ^51^, ^52^ and with lung function decline in subjects with and without asthma.^53^ Eosinophil counts, atopy, and asthma traits were prominent in phenome-wide scans of our top eight signals, consistent with an important role for type 2 inflammation in ACO.^54^^,^^55^

One intergenic signal on chromosome 5 (rs80101740) is not previously reported as associated with asthma, COPD, or lung function. Although near to a putative signal for lung function without replication support (rs377731, *r*^2^ = 0.02 with rs80101740),^33^ the ACO sentinel was independent of this lung function signal in conditional analyses. Evidence from eQTL studies suggests that the nearby lung function signal is associated with *RGMB* and *LINC02062* expression*.*

Four of the eight signals identified as novel (*GLB1*, *FAM105A*, *PHB*, *TSLP*) are known signals for asthma or allergic disease, but not COPD. Our results suggest that these loci also have a role in COPD. All four have been associated with child- and adult-onset asthma, and could represent an opportunity to intervene in early life to prevent serious long-term sequelae.^26^ One ACO signal (*IL17RD*) is a known lung function and COPD locus; our findings demonstrate its relevance in reversible airflow obstruction. Together, these loci could represent targets for intervention, potentially to prevent development of fixed airflow obstruction.

Two signals are known to be associated with asthma and COPD/lung function, including the *HLA-DQB1* locus (the first signal identified as associated with both asthma and COPD), and a signal at *C5orf56*, encoding *IRF1-AS1*, on chromosome 5, near a cytokine gene cluster.

In subgroup analyses, there was a strong positive correlation between stage 1 effect sizes for ACO in ever and never smokers, suggesting that ACO is not due solely to smoking in people with asthma, although childhood asthma in smokers increases COPD risk compared with nonasthmatics, possibly via early lung development.^56^ Similarly, when stratifying by child- vs adult-onset asthma, there was a strong correlation between effect sizes in both groups. Nevertheless, for some of the eight top signals, we found evidence of chromatin interactions in fetal but not adult lung. Although this may implicate developmental processes in ACO, inference is difficult, due to differences in experimental conditions, sample sizes, and reporting practices. Clearer conclusions may become possible as functional genomic assays advance.

Our study has some potential limitations. The stage 2 sample size (4,301 case subjects) was substantial, although relatively underpowered compared with stage 1 (8,068 case subjects). All signals reported met commonly adopted criteria for genome-wide significance, but stricter criteria are starting to be used for genome sequencing studies^57^; future work using sequence data would provide an opportunity to reevaluate the genomic regions we highlight. Misclassification of asthma and COPD diagnoses is possible: asthma in older patients may mimic COPD, and physicians may be less likely to suspect COPD in nonsmokers. To mitigate this, we used GOLD 2+ spirometric criteria to define COPD wherever possible, and note that self-reported asthma has been shown to accurately identify subjects with clinical and genetic characteristics of asthma.^56^ We hypothesize that any remaining misclassification would attenuate effect estimates toward the null, that is, reduce power to detect true genetic associations with ACO. Our main analysis was undertaken in European ancestry populations only; although for many loci there was good concordance in a small sample of participants of African American ethnicity, it is essential to study this trait further in diverse populations.

## Interpretation

In the largest genome-wide association study to date, we identified eight signals associated with ACO. Our findings suggest a spectrum of shared genetic influences, from variants predominantly influencing asthma to those predominantly influencing fixed airflow obstruction. We focus on variants that tend toward an intermediate phenotype with features of both asthma and fixed airflow obstruction, with pathways implicating innate and adaptive immunity and potentially bone development, and signals for which the biology remains unclear. Further biological understanding is likely to be important for therapeutics to prevent the development of fixed airflow obstruction among people with asthma.

## Supplementary Data

e-Online Data
