# Supplementary material for: Genetic Associations and Architecture of Asthma-COPD Overlap
Source: Chest. 2022 Jan 31;161(5):1155–66. doi: 10.1016/j.chest.2021.12.674 (PMC9131047; doi:10.1016/j.chest.2021.12.674)
Supplement: e-Online Data [file mmc1.pdf]

CHEST Online Supplement

Genetic Associations and Architecture of Asthma-COPD Overlap

Catherine John, MBBChir; Anna L. Guyatt, MBChB, PhD; Nick Shrine, PhD; Richard Packer, MBBS; Thorunn A. Olafsdottir, PhD; Jiangyuan Liu, PhD; Lystra P. Hayden, MD; Su H. Chu, PhD; Jukka T. Koskela, MD, PhD; Jian'an Luan, PhD; Xingnan Li, PhD; Natalie Terzikhan, PhD; Hanfei Xu, PhD; Traci M. Bartz, MS; Hans Petersen, MS; Shuguang Leng, MD, PhD; Steven A. Belinsky, PhD; Aivaras Cepelis, PhD; Ana I. Hernández Cordero, PhD; Ma'en Obeidat, BSPharm, PhD; Gudmar Thorleifsson, PhD; Deborah A. Meyers, PhD; Eugene R. Bleecker, MD; Lori C. Sakoda, PhD; Carlos Iribarren, MD, PhD; Yohannes Tesfaigzi, PhD; Sina A. Gharib, MD; Josée Dupuis, PhD; Guy Brusselle, MD, PhD; Lies Lahousse, PhD; Victor E. Ortega, MD, PhD; Ingileif Jonsdottir, PhD; Don D. Sin, MD; Yohan Bossé, PhD; Maarten van den Berge, MD, PhD; David Nickle, PhD; Jennifer K. Quint, MBBS, PhD; Ian Sayers, PhD; Ian P. Hall, MBBCh, PhD; Claudia Langenberg, MD, PhD; Samuli Ripatti, PhD; Tarja Laitinen, MD, PhD; Ann C. Wu, MD; Jessica Lasky-Su, ScD; Per Bakke, MD; Amund Gulsvik, MD, PhD; Craig P. Hersh, MD; Caroline Hayward, PhD; Arnulf Langhammer, MD, PhD; Ben Brumpton, MD, PhD; Kari Stefansson, MD, PhD; Michael H. Cho, MD; Louise V. Wain, PhD; and Martin D. Tobin, MBChB, PhD

CHEST 2022; 161(5):1155-1166

Online supplements are not copyedited prior to posting and the author(s) take full responsibility for the accuracy of all data.

© 2018 AMERICAN COLLEGE OF CHEST PHYSICIANS. Reproduction of this article is prohibited without written permission from the American College of Chest Physicians. See online for more details. **DOI:** 10.1016/j.chest.2021.12.674

Supplementary Material

Supplementary Tables 1-12 are included at the end of the online supplement.

Contents

e-Appendix 3

Previous signals for risk of asthma or COPD (including lung function) 3

Study Descriptions........ 3

Supplementary Methods: Stage 1 6

Data source and study population 6

Case and control selection 6

Identification of distinct signals........ 7

Supplementary Methods: Stage 2 and joint analysis 7

Studies and meta-analysis........ 7

Validation of asthma diagnosis definition for Stage 2 7

Sensitivity analysis 7

Signal fine-mapping and bioinformatic analyses 8

Supplementary Results........ 9

Signal selection........ 9

Sensitivity analysis 9

Study acknowledgements and funding 10

e-Figure 1: Quantile-quantile plot of ACO GWAS results from discovery analysis in UK Biobank........14

e-Figure 2: Flowchart detailing the signal selection process in the UK Biobank discovery analysis 15

e-Figure 3 Scatterplots comparing effects in 31 signals taken forward for replication, with results when ACO cases are split into those with adult- (>25 years) and child-onset (<12 years) asthma........16

e-Figure 4 Scatterplots comparing effects in 31 signals taken forward for replication, with smoking-stratified results (by ever- and never-smokers)........19

e-Figure 5 Region plots for eight signals taken forward for downstream analyses........22

rs80101740........22

rs35570272........22

rs16903574........23

rs2584662........23

rs1837253........23

rs6787279........24

rs9273410........24

rs3749833........24

e-Figure 6 Forest plots for eight signals taken forward for downstream analyses (results from Stage 1 + Stage 2 joint analysis)....25
rs80101740....25
rs35570272....26
rs16903574....27
rs2584662....28
rs1837253....29
rs6787279....30
rs9273410....31
rs3749833....32
References....33

e-Appendix

Previous signals for risk of asthma or COPD (including lung function)

References for GWAS publications detailing loci associated with risk of asthma or COPD (as mentioned in the main text introduction) are given in [1-41].

Study Descriptions

The **Cardiovascular Health Study (CHS)** is a population-based cohort study of risk factors for coronary heart disease and stroke in adults ≥65 years conducted across four field centers.[42] The original predominantly European ancestry cohort of 5,201 persons was recruited in 1989-1990 from random samples of the Medicare eligibility lists; subsequently, an additional predominantly African-American cohort of 687 persons was enrolled for a total sample of 5,888. Blood samples were drawn from all participants at their baseline examination and DNA was subsequently extracted from available samples. Genotyping was performed at the General Clinical Research Center's Phenotyping/Genotyping Laboratory at Cedars-Sinai among CHS participants who consented to genetic testing and had DNA available using the Illumina 370CNV BeadChip system (for European ancestry participants, in 2007) or the Illumina HumanOmni1-Quad_v1 BeadChip system (for African-American participants, in 2010). Only European ancestry participants were included in this analysis due to low numbers of African-Americans with the outcome. For European ancestry participants in this analysis, spirometry values to define COPD and self-reported asthma are from the 1989-1990 exam. CHS was approved by institutional review committees at each field center and individuals in the present analysis had available DNA and gave informed consent including consent to use of genetic information for the study of cardiovascular disease.

COPDGene: Eligible subjects in COPDGene Study (NCT00608764, www.copdgene.org) were of nonHispanic white (NHW) or African-American (AA) ancestry, aged 45-80 years old, with at least 10 packyears of smoking and no diagnosed lung disease other than COPD or asthma.[29,43] IRB approval was obtained at all study centers, and all study participants provided written informed consent. Illumina (San Diego, CA) performed genotyping on the HumanOmniExpress array.

deCODE: The Icelandic GWAS is based on 32.5 million variants identified through whole-genome sequencing of 28,075 Icelandic individuals and subsequently imputed for 155,250 Icelandic individuals, genotyped using the Illumina SNP chips, as well as 285,664 of their first- and second-degree relatives as previously described.[44] All participating individuals who donated blood signed informed consent. The personal identities of participants were encrypted using a third-party system approved and monitored by the Icelandic Data Protection Authority.[45] The study was approved by the National Bioethics Committee in Iceland (Approval no. VSN 19-157 and VSN 15-099).

The Icelandic spirometry data were collected from records at the Department of Respiratory Medicine and Sleep, Landspitali, the University Hospital of Iceland and from a health study performed by deCODE genetics at the participant recruitment center. Spirometry was performed using the EasyOne Pro equipment from ndd Medical Technologies in a standardized manner and following published guidelines https://www.atsjournals.org/doi/10.1164/rccm.201908-1590ST.

ECLIPSE: Evaluation of COPD Longitudinally to Identify Predictive Surrogate End-points (ECLIPSE; SCO104960, NCT00292552, www.eclipse-copd.com): Details of the ECLIPSE study and genome-wide association 4 analysis have been described previously.[46] The ECLIPSE study was approved by the relevant ethics and review boards at the participating clinical centers. All participants provided

written informed consent. Cases and controls were aged 40-75 with at least a 10 pack-year smoking history without other respiratory diseases. Genotyping was performed using the Illumina HumanHap 550 V3 (Illumina, San Diego, CA).

European Prospective Investigation of Cancer (EPIC)-Norfolk (DOI 10.22025/2019.10.105.00004) is a prospective population-based cohort study which recruited 25,639 men and women aged 40-79 years at baseline between 1993 and 1997 from 35 participating general practices in Norfolk, UK.[47] Individuals attended for a baseline health check including the provision of blood samples for concurrent and future analysis. Further health check visits have been conducted since the baseline visit. Participants have contributed information about their diet, lifestyle and health through questionnaires and health checks over two decades. DNA has been extracted from all EPIC participants and stored blood has been analysed for an extensive range of classical and novel biomarkers. Sample quality control was performed including gender check, relatedness check, and ancestry check. The Norwich Local Research Ethics Committee granted ethical approval for the study. All participants gave written informed consent.

FinnCAD/Generisk: The Finnish Chronic Obstructive Airway Disease (CAD) cohort has been enrolled through the Pulmonary Clinics of Helsinki and Turku University Hospitals during the years 2005-2007.[48] All out- and inpatients aged 18–75 years who had been discharged with a diagnosis of asthma or COPD (ICD10 code J44–J46) were invited to the study and 1855 patients participated. Three hundred twenty one of the patients full filled both asthma and COPD criteria and were included to the study. The Coordinating Ethics Committee of the Helsinki and Uusimaa Hospital District (Coordinating Ethics Committee decision 125/E0/04) has approved the study, and the permission to conduct research was granted by the Helsinki and Turku University Hospitals. Controls for this study were selected from the Generisk cohort. The GeneRISK study is being carried out according to the principles of the Helsinki declaration and the Council of Europe's (COE) Convention of Human Rights and Biomedicine. All study participants have given their informed consent that permits the use of collected samples and data for the original study and for biobank research through THL Biobank, and the study protocol has been approved by the Ethical Committee of the Helsinki and Uusimaa Hospital district on 9 December 2014.

Framingham Heart Study (FHS; NCT00005121): Details on pulmonary function in the FHS have been previously published.[22,27] FHS was IRB-approved at the relevant institutions, and all participants provided written informed consent. We analyzed data from the most recent exam for each of the three generations of families participating in the FHS were analyzed. Genotypes were from the Affymetrix 500K array supplemented by the Affymetrix MIPS 50K.

Generation Scotland is a multi-institution collaboration that has created an ethically sound, family-based and population-based resource for identifying the genetic basis of common complex diseases.[49] The Scottish Family Health Study component (GS:SFHS) has DNA and sociodemographic, psychological and clinical data from ~24,000 adult volunteers from across Scotland. The ethnicity of the cohort is 99% Caucasian, with 96% born in the UK and 87% in Scotland. Features of GS:SFHS include the family-based recruitment, breadth and depth of phenotype information, 'broad' consent from participants to use their data and samples for a wide range of medical research and for re-contact, and consent and mechanisms for linkage of all data to comprehensive routine healthcare records. These features were designed to maximise the power of the resource to identify, replicate or control for genetic factors associated with a wide spectrum of illnesses and risk factors. GS:SFHS has Research Tissue Bank status from the East of Scotland Research Ethics Service (REC Reference Number: 15/ES/0040). This provides a favourable opinion for a wide range of data and sample uses within medical research. Research that includes access to individual-level EHR data is notified to the

Research Ethics Committee by the GS management team on behalf of the researchers, through a notice of substantial amendment. Only data from those GS:SFHS participants who gave written informed consent for record linkage of their GS:SFHS study data to their medical records are used.

GenKOLS (Norway): The Norwegian GenKOLS (Genetics of Chronic Obstructive Lung Disease, GSK code RES11080) recruited subjects with > 2.5 pack years of smoking history from Bergen, Norway.[50] Subjects with severe alpha-1 antitrypsin deficiency and other lung diseases (aside from asthma) were excluded. The Regional Committee for Medical Research Ethics (REK Vest), the Norwegian Data Inspectorate and the Norwegian Department of Health approved the case–control study. Written informed consent was obtained from all participants. Genotyping was performed using Illumina HumanHap 550 arrays (Illumina, San Diego, CA).

GERA: This study utilized genome-wide genetic data available on the Genetic Epidemiology Resource in Adult Health and Aging (GERA) cohort of 110,266 adult male and female Kaiser Permanente of Northern California (KPNC) members. The cohort has been described in detail elsewhere.[51] In brief, the GERA cohort was formed by including all racial and ethnic minority participants in the larger cohort of the Research Program on Genes, Environment and Health (RPGEH) with saliva samples (19% of the total); the remaining participants were drawn randomly from White non-Hispanic participants (81% of the total). All RPGEH participants responded to a self-administered questionnaire in 2007/08 that included information on medical history, ancestry, health behaviors (smoking, alcohol consumption, diet, physical activity and reproductive history) and current weight and height. Asthma and COPD phenotypes were derived from the KPNC electronic health record and linked with the genetic data by virtue of the unique medical record number. The institutional review boards for human subject research of both KPNC and University of California, San Francisco (UCSF), approved the project (AG036607; Schaefer/Risch, PIs).

HUNT: The Trøndelag Health Study (HUNT)[52] is a population-based health survey conducted in the county of Nord Trøndelag, Norway. Individuals were included at four different time points during approximately 20 years (HUNT1 [1984-1986], HUNT2 [1995-1997], HUNT3 [2006-2008]) and HUNT4 [2017-2019])(PMID: 22879362). At each time point, the entire adult population (≥ 20 years) was invited to participate by completing questionnaires, attending clinical examinations and interviews. Participation rates have generally been high: 89.4% (n = 77,212), 69.5% (n = 65 237), 54.1% (n = 50 807) and 54.0% (n=56042) in HUNT1, HUNT2, HUNT3 and HUNT4, respectively (PMID: 22879362). Taken together, the health studies include information from over 120,000 different individuals from Nord-Trøndelag. Biological samples including DNA have been collected for approximately 90,000 participants. Participation was based on informed, written consent, and the study was approved by the Regional Committee for Medical and Health Research (#2015/616-1 REK midt). In addition, the HUNT Study was approved by the Norwegian Data Inspectorate.

Lovelace: The Lovelace Smokers Cohort (LSC) has been actively enrolling smokers from the Albuquerque, NM metropolitan area since 2001.[53] All participants provided written informed consent, and the study was approved by the relevant IRB. Enrollment was restricted to current and former smokers age 40 to 74 years old with a minimum of 10 pack-years of smoking and no personal history of lung cancer. A detailed questionnaire written in English was used to collect information on demographics; medical, cigarette smoking, and exposure history; socioeconomic status; diet; and quality of life. Pulmonary function testing was performed at each visit. All participants signed a consent form, and the Western Institutional Review Board approved this project. The GWAS discovery set was comprised of 1200 Caucasian (self-reported) smokers. The HumanOmni2.5-4v1-H BeadChip (Illumina, San Diego, CA) was used to genotype 2,450,000 SNPs in 1200 Caucasian smokers from the LSC.

MGB-Biobank: The Massachusetts General and Brigham Biobank (MGB-Biobank) Biobank is a large bio-repository that provides access to research data and approximately 90,000 high-quality banked samples (plasma, serum, and DNA) from >129,000 consented patients enrolled in the Partners Healthcare system, that enable these samples to be linked to corresponding Electronic Medical Record (EMR) data in addition to survey data on lifestyle, environment, and family history. The protocol for this study was approved by the Brigham and Women's Hospital and the Mass General Brigham institutional review boards.

The **Rotterdam Study** is a prospective population-based cohort study founded in 1990 in a suburb of Rotterdam, the Netherlands.[54,55] The first cohort (RS-I) consists of 7,983 participants, aged 55 years and over. The second cohort (RS-II) was recruited in 2000 with the same inclusion criteria. The third cohort (RS-III) consists of 3,932 participants, aged 45 years and over and was recruited in 2006. The Rotterdam Study was approved by the institutional review board (Medical Ethics Committee) of the Erasmus Medical Center and by the review board of The Netherlands Ministry of Health, Welfare and Sports. All participants provided written informed consent. Spirometry was performed using the Master Screen® PFT Pro (CareFusion, San Diego, CA). A total of 6,291 subjects for RS I, 2,157 for RS II and 3,048 for RS III passed genotyping quality control.

SPIROMICS is a prospective cohort (n=2,981) with the goal to identify COPD subphenotypes and biomarkers of disease progression.[56] SPIROMICS is a well-characterized longitudinal cohort with comprehensive phenotyping including measurements of lung function and quantitative CT scans. Smokers with COPD were defined as smokers (smoking≥20 packs/year) with post-bronchodilator FEV1/FVC<0.7 (GOLD stage 1-4) and 'healthy' smoking controls were defined as smokers (smoking≥20 packs/year) with post-bronchodilator FEV1/FVC≥0.7 (GOLD stage 0). Healthy non-smoking controls were also recruited. Participants were recruited at each center using a common detailed protocol (www.spiromics.com). The study was approved by the institutional review boards of all participating sites with written informed consent from all participants. DNA was isolated using standard protocols, SNP genotyping performed using Illumina HumanOmniExpressExome BeadChip and BeadStudio (Illumina, Inc., San Diego, CA), and quality control and genetic association analysis were described previously.[57]

Supplementary Methods: Stage 1

Data source and study population

Individuals were eligible for inclusion in this study if they met the following criteria: (i) had data on age, sex and height; (ii) had spirometry that met quality control requirements (acceptability, reproducibility and blow curve metrics); (iii) had genome-wide imputed genetic data that met quality control requirements; and (iv) were of European ancestry based on *k*-means clustering after principal components analysis. Quality control processes have been described previously.[58]

Genotyping was undertaken using the Affymetrix Axiom® UK BiLEVE array and the Affymetrix Axiom® UK Biobank array,[30] with imputation to the Haplotype Reference Consortium panel.[59]

Case and control selection

Individuals were selected as cases of ACO if they had evidence of asthma from either the touchscreen questionnaire (UK Biobank data field 6152, "Has a doctor ever told you that you have had any of the following conditions? You can select more than one answer") or verbal interview with a trained nurse (field 20002) AND FEV_1/FVC <0.7 with classification of airflow limitation GOLD 2+ (FEV_1 <80% of predicted) at any study visit. Individuals were excluded from the cases if they reported a diagnosis of alpha-1-antitrypsin deficiency in the verbal interview (field 20002) at any study visit.

Where there were related pairs within the cases (second degree or closer), the individual with the lower genotype call rate was excluded.

Controls were free of both asthma and COPD, based on no reported bronchitis, emphysema or asthma on the touchscreen questionnaire, and no reported asthma, COPD, emphysema/chronic bronchitis, bronchitis, emphysema, alpha-1-antitrypsin deficiency on the verbal interview. Controls had FEV_1 ≥80% predicted and FEV_1/FVC >0.7. Individuals related to cases were excluded, and where pairs of controls were related, the individual with the lower genotype call rate was excluded (as for cases). Controls were randomly selected in a ratio of five to each case.

Identification of distinct signals

In stage 1, we sought to defined distinct signals across the genome passing a P-value threshold of P<5x10-6.

We defined regions of association around the most strongly associated variant (sentinel variant) ±1Mb. First, sentinel variants and regions of association were identified by taking the variant with the lowest P-value and extracting this sentinel variant and the surrounding region ±1Mb. Then, the variant with the next lowest P-value outside this region was identified, proceeding iteratively until there were no further variants with a P-value less than 5x10-6. All coordinates are given according to GRCh37.

To identify distinct signals, and additional signals within the regions described above, conditional analyses were undertaken using GCTA-COJO (http://cnsgenomics.com/software/gcta/#COJO). For non-HLA sentinel variants, GCTA-COJO was used to implement a joint, stepwise selection procedure for each of the sentinels and its corresponding 2Mb surrounding region. For the HLA region, one conditional analysis was performed for the region of chr6:26,000,000-34,000,000.

Supplementary Methods: Stage 2 and joint analysis

Studies and meta-analysis

Studies undertook logistic regression with ACO as the outcome, using an additive genetic model, and adjusting for age, sex and, where available, smoking status. An appropriate number of ancestry principal components were also included, or a mixed linear model used to account for fine-scale population structure or familial relatedness. Proxies (r^2>0.3) were provided if SNPs were missing, or if a SNP was poorly imputed (info <0.5) in a study (see **e-Table 3**).

Validation of asthma diagnosis definition for Stage 2

In Stage 2 analyses, we permitted asthma to be diagnosed by either self-report of doctor-diagnosed asthma, or a diagnosis from the electronic healthcare record. We undertook pilot work to compare prevalence of ACO defined in UK Biobank, using self-report of asthma (not necessarily doctor-diagnosed), and in the EXCEED cohort,[60] using asthma codes identified from primary care data. GOLD2+ spirometry was used to define COPD in both cohorts. The proportion of ACO cases amongst individuals with spirometry data passing quality control was very similar in both studies (2.5% in UK Biobank, 2.4% in EXCEED).

Sensitivity analysis

In addition to the main meta-analysis, we undertook a sensitivity meta-analysis in which we varied the definition of COPD.

We expanded the definition of COPD to include a diagnosis in the healthcare record (including billing codes) in three cohorts already included in the main analysis where this information was also available (deCODE, HUNT, Rotterdam), and in three additional cohorts which were not included in

the main analysis, due to lack of spirometry data (FinnCAD/Generisk, GERA, MGB Biobank). Thus, in the sensitivity analysis, cases of ACO had COPD defined either by spirometry (if available) or by a diagnosis in the healthcare record (in addition to evidence of asthma).

Controls had either normal spirometry (if available) or absence of a COPD diagnosis in the healthcare record (in addition to no evidence of asthma) (**e-Appendix**).

As in the main analysis, studies undertook logistic regression with ACO as the outcome, using an additive genetic model, and adjusting for age, sex and, where available, smoking status. An appropriate number of ancestry principal components were also included, or a mixed linear model used to account for fine-scale population structure. Proxies (r^2>0.3) were provided if SNPs were missing or poorly imputed (info<0.5) in a study (see **e-Table 3**).

Signal fine-mapping and bioinformatic analyses

For each of the top signals, we identified the set of SNPs that was 99% likely to contain the causal variant (the '99% credible set', assuming that the causal variant was included in the dataset) **(e-Appendix)**.[61] This method calculates the posterior probabilities for each variant being causal, using approximate Bayes factors (ABFs), as proposed by Wakefield.[61] The credible set is constructed by adding the variants with the highest probabilities to the set until the sum of the probabilities exceeds 0.99. We set the value of W (the variance of the prior distribution of effect sizes) in the approximate Bayes factor formula to 0.04.[58,61]

We annotated relevant variants using wANNOVAR[62] (http://wannovar.wglab.org/), SIFT,[63] FATHMM [implemented using https://www.ensembl.org/vep and http://fathmm.biocompute.org.uk/],[64] and PolyPhen-2 [implemented using the HumDiv model via http://genetics.bwh.harvard.edu/pph2/][65], and CADD.[58,66]

We queried the 99% credible sets against expression quantitative trait locus (eQTL) resources in lung (n=1,038),[30,67-69] and the GTEx online portal (v8) [lung N=515, whole blood N=670],[70] to determine whether the locus was significantly associated with gene expression, i.e. whether it was an eQTL.

Functional information about nearby genes, or those implicated through eQTL analysis, was retrieved from the National Institute of Health Genetics Home Reference.

For one signal not previously associated with asthma, lung function or COPD, we performed formal testing for Bayesian colocalisation with cis-eQTL signals (gene genomic position ±0.5 Mb) using the R package COLOC.[71] Briefly, this tests whether the GWAS signal and eQTL signal for this region are consistent with shared causal variant(s).[72] A large posterior probability is supporting evidence of a single shared causal variant.

We entered each sentinel SNP into the Hi-C Unifying Genomic Interrogator (HUGIn, http://yunliweb.its.unc.edu/HUGIn) and looked for long-range chromatin interactions with other genomic regions (in adult lung and a fetal lung fibroblast cell line) where the false discovery rate (FDR) was <5%.[73-75]

To assess the associations of SNPs in the credible sets with other traits, we undertook a phenome-wide scan using PhenoScanner (http://www.phenoscanner.medschl.cam.ac.uk/).[76] We used an FDR threshold (calculated by the Benjamini-Hochberg method) <1%. Evidence of association with rare diseases was sought in OMIM and Orphanet for the nearest gene to any SNP in the credible set, and genes implicated by eQTL evidence.

Supplementary Results

Signal selection

There were 80 sentinel variants associated with ACO at $P<5x10^{-6}$, of which 28 reached genome-wide significance ($P<5x10^{-8}$). Of the 80 sentinels, four were located in the major histocompatibility complex (MHC) region (chr6:28,477,797-33,448,354), with a further three located just outside this region (chr6:26,000,000-34,000,000).

Conditional analysis of the 73 non-HLA sentinel variants identified five additional independent signals within 1Mb of these sentinels, plus two conditional signals (only associated at $P<5x10^{-6}$ after conditioning on the sentinel variant), giving a total of 80 conditionally independent non-HLA signals. Separate conditional analysis of the extended HLA region identified three HLA signals.

Sensitivity analysis

9638 cases were included in this analysis, and 26/31 signals had a direction of effect that was concordant with the Stage 1 analysis. The strongest signal was rs9273410, OR 1.103, 95% CI 1.055, 1.153, $P=1.55x10^{-5}$, $I^2=0\%$, **e-Table 7**). Of the 26/31 signals directionally concordant with Stage 1, 24/31 were also directionally concordant with the main Stage 2 meta-analysis. Three of the 8 signals which met the criteria for follow-up in the main analysis also met the criteria for follow-up in this analysis, and no other signals of the remaining 23/31 met these criteria (p<5x10-8 in the joint analysis of stage 1 and stage 2, and either a lower p-value in the joint analysis than in UK Biobank alone, or p<0.05 in stage 2 alone).

Study acknowledgements and funding

Cardiovascular Health Study: This CHS research was supported by NHLBI contracts HHSN268201200036C, HHSN268200800007C, HHSN268201800001C, N01HC55222, N01HC85079, N01HC85080, N01HC85081, N01HC85082, N01HC85083, N01HC85086; and NHLBI grants U01HL080295, R01HL087652, R01HL105756, R01HL103612, R01HL120393, and U01HL130114 with additional contribution from the National Institute of Neurological Disorders and Stroke (NINDS). Additional support was provided through R01AG023629 from the National Institute on Aging (NIA). A full list of principal CHS investigators and institutions can be found at CHS-NHLBI.org. The provision of genotyping data was supported in part by the National Center for Advancing Translational Sciences, CTSI grant UL1TR001881, and the National Institute of Diabetes and Digestive and Kidney Disease Diabetes Research Center (DRC) grant DK063491 to the Southern California Diabetes Endocrinology Research Center. The content is solely the responsibility of the authors and does not necessarily represent the official views of the National Institutes of Health.

COPDGene: *Grant Support and Disclaimer:* The project described was supported by Award Number U01 HL089897 and Award Number U01 HL089856 from the National Heart, Lung, and Blood Institute. The content is solely the responsibility of the authors and does not necessarily represent the official views of the National Heart, Lung, and Blood Institute or the National Institutes of Health. *COPD Foundation Funding:* The COPDGene® project is also supported by the COPD Foundation through contributions made to an Industry Advisory Board comprised of AstraZeneca, Boehringer Ingelheim, GlaxoSmithKline, Novartis, Pfizer, Siemens and Sunovion. **COPDGene® Investigators – Core Units.** *Administrative Center*: James D. Crapo, MD (PI); Edwin K. Silverman, MD, PhD (PI); Barry J. Make, MD; Elizabeth A. Regan, MD, PhD. *Genetic Analysis Center*: Terri Beaty, PhD; Ferdouse Begum, PhD; Peter J. Castaldi, MD, MSc; Michael Cho, MD; Dawn L. DeMeo, MD, MPH; Adel R. Boueiz, MD; Marilyn G. Foreman, MD, MS; Eitan Halper-Stromberg; Lystra P. Hayden, MD, MMSc; Craig P. Hersh, MD, MPH; Jacqueline Hetmanski, MS, MPH; Brian D. Hobbs, MD; John E. Hokanson, MPH, PhD; Nan Laird, PhD; Christoph Lange, PhD; Sharon M. Lutz, PhD; Merry-Lynn McDonald, PhD; Margaret M. Parker, PhD; Dmitry Prokopenko, Ph.D; Dandi Qiao, PhD; Elizabeth A. Regan, MD, PhD; Phuwanat Sakornsakolpat, MD; Edwin K. Silverman, MD, PhD; Emily S. Wan, MD; Sungho Won, PhD. *Imaging Center*: Juan Pablo Centeno; Jean-Paul Charbonnier, PhD; Harvey O. Coxson, PhD; Craig J. Galban, PhD; MeiLan K. Han, MD, MS; Eric A. Hoffman, Stephen Humphries, PhD; Francine L. Jacobson, MD, MPH; Philip F. Judy, PhD; Ella A. Kazerooni, MD; Alex Kluiber; David A. Lynch, MB; Pietro Nardelli, PhD; John D. Newell, Jr., MD; Aleena Notary; Andrea Oh, MD; Elizabeth A. Regan, MD, PhD; James C. Ross, PhD; Raul San Jose Estepar, PhD; Joyce Schroeder, MD; Jered Sieren; Berend C. Stoel, PhD; Juerg Tschirren, PhD; Edwin Van Beek, MD, PhD; Bram van Ginneken, PhD; Eva van Rikxoort, PhD; Gonzalo Vegas Sanchez-Ferrero, PhD; Lucas Veitel; George R. Washko, MD; Carla G. Wilson, MS; *PFT QA Center, Salt Lake City, UT*: Robert Jensen, PhD. *Data Coordinating Center and Biostatistics, National Jewish Health, Denver, CO*: Douglas Everett, PhD; Jim Crooks, PhD; Katherine Pratte, PhD; Matt Strand, PhD; Carla G. Wilson, MS. *Epidemiology Core, University of Colorado Anschutz Medical Campus, Aurora, CO*: John E. Hokanson, MPH, PhD; Gregory Kinney, MPH, PhD; Sharon M. Lutz, PhD; Kendra A. Young, PhD. *Mortality Adjudication Core:* Surya P. Bhatt, MD; Jessica Bon, MD; Alejandro A. Diaz, MD, MPH; MeiLan K. Han, MD, MS; Barry Make, MD; Susan Murray, ScD; Elizabeth Regan, MD; Xavier Soler, MD; Carla G. Wilson, MS. *Biomarker Core*: Russell P. Bowler, MD, PhD; Katerina Kechris, PhD; Farnoush Banaei-Kashani, Ph.D. **COPDGene® Investigators – Clinical Centers.** *Ann Arbor VA:* Jeffrey L. Curtis, MD; Perry G. Pernicano, MD. *Baylor College of Medicine, Houston, TX*: Nicola Hanania, MD, MS; Mustafa Atik, MD; Aladin Boriek, PhD; Kalpatha Guntupalli, MD; Elizabeth Guy, MD; Amit Parulekar, MD. *Brigham and Women's Hospital, Boston, MA*: Dawn L. DeMeo, MD, MPH; Alejandro A. Diaz, MD, MPH; Lystra P. Hayden, MD; Brian D. Hobbs, MD; Craig

Hersh, MD, MPH; Francine L. Jacobson, MD, MPH; George Washko, MD. *Columbia University, New York, NY*: R. Graham Barr, MD, DrPH; John Austin, MD; Belinda D'Souza, MD; Byron Thomashow, MD. *Duke University Medical Center, Durham, NC*: Neil MacIntyre, Jr., MD; H. Page McAdams, MD; Lacey Washington, MD. *Grady Memorial Hospital, Atlanta, GA: Eric Flenaugh, MD; Silanth Terpenning, MD. HealthPartners Research Institute, Minneapolis, MN*: Charlene McEvoy, MD, MPH; Joseph Tashjian, MD. *Johns Hopkins University, Baltimore, MD*: Robert Wise, MD; Robert Brown, MD; Nadia N. Hansel, MD, MPH; Karen Horton, MD; Allison Lambert, MD, MHS; Nirupama Putcha, MD, MHS. *Lundquist Institute for Biomedical Innovationat Harbor UCLA Medical Center, Torrance, CA*: Richard Casaburi, PhD, MD; Alessandra Adami, PhD; Matthew Budoff, MD; Hans Fischer, MD; Janos Porszasz, MD, PhD; Harry Rossiter, PhD; William Stringer, MD. *Michael E. DeBakey VAMC, Houston, TX*: Amir Sharafkhaneh, MD, PhD; Charlie Lan, DO. *Minneapolis VA:* Christine Wendt, MD; Brian Bell, MD; Ken M. Kunisaki, MD, MS. *National Jewish Health, Denver, CO*: Russell Bowler, MD, PhD; David A. Lynch, MB. *Reliant Medical Group, Worcester, MA*: Richard Rosiello, MD; David Pace, MD. *Temple University, Philadelphia, PA:* Gerard Criner, MD; David Ciccolella, MD; Francis Cordova, MD; Chandra Dass, MD; Gilbert D'Alonzo, DO; Parag Desai, MD; Michael Jacobs, PharmD; Steven Kelsen, MD, PhD; Victor Kim, MD; A. James Mamary, MD; Nathaniel Marchetti, DO; Aditi Satti, MD; Kartik Shenoy, MD; Robert M. Steiner, MD; Alex Swift, MD; Irene Swift, MD; Maria Elena Vega-Sanchez, MD*. University of Alabama, Birmingham, AL:* Mark Dransfield, MD; William Bailey, MD; Surya P. Bhatt, MD; Anand Iyer, MD; Hrudaya Nath, MD; J. Michael Wells, MD. *University of California, San Diego, CA*: Douglas Conrad, MD; Xavier Soler, MD, PhD; Andrew Yen, MD. *University of Iowa, Iowa City, IA*: Alejandro P. Comellas, MD; Karin F. Hoth, PhD; John Newell, Jr., MD; Brad Thompson, MD. *University of Michigan, Ann Arbor, MI:* MeiLan K. Han, MD MS; Ella Kazerooni, MD MS; Wassim Labaki, MD MS; Craig Galban, PhD; Dharshan Vummidi, MD*. University of Minnesota, Minneapolis, MN*: Joanne Billings, MD; Abbie Begnaud, MD; Tadashi Allen, MD. *University of Pittsburgh, Pittsburgh, PA*: Frank Sciurba, MD; Jessica Bon, MD; Divay Chandra, MD, MSc; Carl Fuhrman, MD; Joel Weissfeld, MD, MPH. *University of Texas Health, San Antonio, San Antonio, TX*: Antonio Anzueto, MD; Sandra Adams, MD; Diego Maselli-Caceres, MD; Mario E. Ruiz, MD; Harjinder Singh.

ECLIPSE: The ECLIPSE study (NCT00292552; GSK code SCO104960) was funded by GSK.

EPIC-Norfolk: The EPIC-Norfolk study team are grateful to all the participants who have been part of the project and to the many members of the study teams at the University of Cambridge who have enabled this research. The study is funded by Medical Research Council (MR/N003284/1, MC-UU_12015/1, MC_PC_13048) Cancer Research UK (C864/A14136).

FHS: This work was partially supported by the National Heart, Lung and Blood Institute's **Framingham Heart Study** (contract number N01-HC-25195) and its contract with Affymetrix, Inc for genotyping services (contract number N02-HL-6-4278). Also supported by NIH P01 AI050516.

GASP (moderate-severe asthma correlations): cohort collection and genotyping was funded by; Asthma UK Grant to I.S. (AUK-PG-2013-188), Rosetrees Tust (Grant to I.S.), AirPROM, U-BIOPRED (EU-IMI 115010) and an MRC Strategic Award to I.P.H., M.D.T., L.V.W. and Professor David Strachan (MC_PC_12010). Recruitment of asthma patients in Manchester was funded by the NIHR and the North West Lung Centre Charity.

Generation Scotland received core support from the Chief Scientist Office of the Scottish Government Health Directorates [CZD/16/6] and the Scottish Funding Council [HR03006]. Genotyping was funded by the Medical Research Council UK and the Wellcome Trust (Wellcome Trust Strategic Award "STratifying Resilience and Depression Longitudinally" (STRADL) Reference 104036/Z/14/Z). .CH is supported by an MRC University Unit Programme Grant MC_UU_00007/10

(QTL in Health and Disease). We thank all the families who took part, the general practitioners and the Scottish School of Primary Care for their help in recruiting them, and the whole Generation Scotland team, which includes interviewers, computer and laboratory technicians, clerical workers, research scientists, volunteers, managers, receptionists, healthcare assistants and nurses. Genotyping of the GS:SFHS samples was carried out by the Genetics Core Laboratory at the Edinburgh Clinical Research Facility, University of Edinburgh, Scotland.

GenKOLS: The Norway GenKOLS study (Genetics of Chronic Obstructive Lung Disease, GSK code RES11080) was funded by GSK.

HUNT: The Trøndelag Health Study (The HUNT Study) is a collaboration between HUNT Research Center (Faculty of Medicine and Health Sciences, NTNU, Norwegian University of Science and Technology), Nord-Trøndelag County Council, Central Norway Regional Health Authority, and the Norwegian Institute of Public Health. The K.G. Jebsen Center for Genetic Epidemiology is funded by Stiftelsen Kristian Gerhard Jebsen; Faculty of Medicine and Health Sciences, NTNU; The Liaison Committee for education, research and innovation in Central Norway; and the Joint Research Committee between St. Olavs Hospital and the Faculty of Medicine and Health Sciences, NTNU. The genotyping in HUNT was financed by the National Institute of Health (NIH); University of Michigan; The Research Council of Norway; The Liaison Committee for education, research and innovation in Central Norway; and the Joint Research Committee between St. Olavs Hospital and the Faculty of Medicine and Health Sciences, NTNU.

Lovelace Smokers Cohort: The cohort and analysis was primarily supported by National Cancer Institute grant R01 CA097356 (SAB). The State of New Mexico as a direct appropriation from the Tobacco Settlement Fund to SAB through collaboration with University of New Mexico provided initial support to establish the LSC. Additional support was provided by NIH/NCI P30 CA118100 (SAB), HL68111 (Y.T.), and HL107873-01 (YT and SB).

Rotterdam Study: The study is funded by Erasmus Medical Center and Erasmus University, Rotterdam, Netherlands Organization for the Health Research and Development (ZonMw), the Research Institute for Diseases in the Elderly (RIDE), the Ministry of Education, Culture and Science, the Ministry for Health, Welfare and Sports, the European Commission (DG XII), and the Municipality of Rotterdam. The generation and management of GWAS genotype data for the Rotterdam Study (RS I, RS II, RS III) was executed by the Human Genotyping Facility of the Genetic Laboratory of the Department of Internal Medicine, Erasmus MC, Rotterdam, The Netherlands. The GWAS datasets are supported by the Netherlands Organisation of Scientific Research NWO Investments (nr. 175.010.2005.011, 911-03-012), the Genetic Laboratory of the Department of Internal Medicine, Erasmus MC, the Research Institute for Diseases in the Elderly (014-93-015; RIDE2), the Netherlands Genomics Initiative (NGI)/Netherlands Organisation for Scientific Research (NWO) Netherlands Consortium for Healthy Aging (NCHA), project nr. 050-060-810. The generation and management of spirometric data was supported by FWO project G035014N.

SPIROMICS: The Subpopulations and Intermediate Outcomes in COPD Study was supported by contracts from the NIH/NHLBI (HHSN268200900013C, HHSN268200900014C, HHSN268200900015C, HHSN268200900016C, HHSN268200900017C, HHSN268200900018C, HHSN268200900019C, HHSN268200900020C), which were supplemented by contributions made through the Foundation for the NIH from AstraZeneca; Bellerophon Therapeutics; Boehringer-Ingelheim Pharmaceuticals, Inc; Chiesi Farmaceutici SpA; Forest Research Institute, Inc; GSK; Grifols Therapeutics, Inc; Ikaria, Inc; Nycomed GmbH; Takeda Pharmaceutical Company; Novartis Pharmaceuticals Corporation;

Regeneron Pharmaceuticals, Inc; and Sanofi. We acknowledge all investigators, staff, and participants in SPIROMICS.

e-Figure 1: Quantile-quantile plot of ACO GWAS results from discovery analysis in UK Biobank

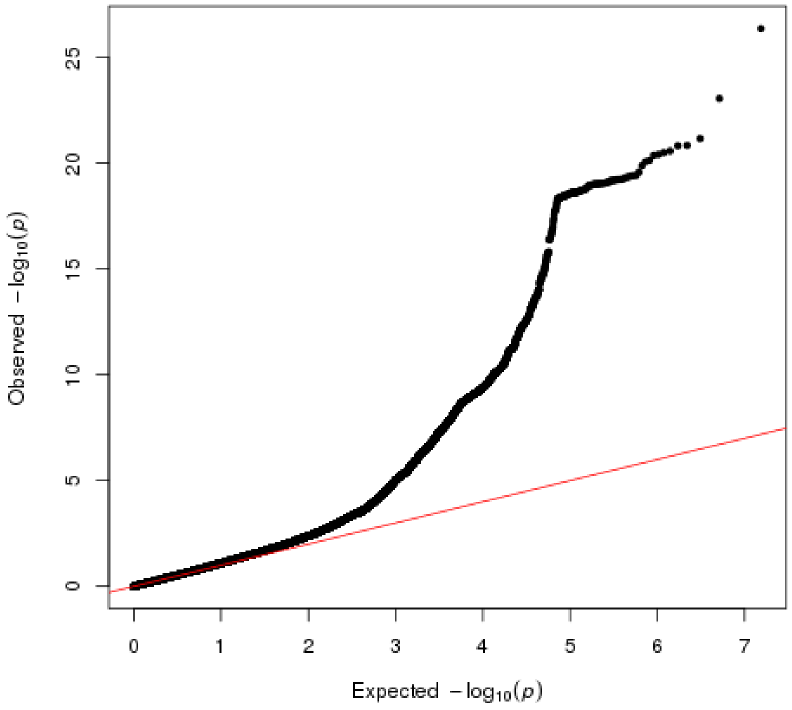

8068 ACO cases, 40360 controls, 7,693,381 SNPS. Intercept value from univariate LD score regression = 1.018. Standard errors and p-values in UK Biobank are corrected for this value.

e-Figure 2: Flowchart detailing the signal selection process in the UK Biobank discovery analysis

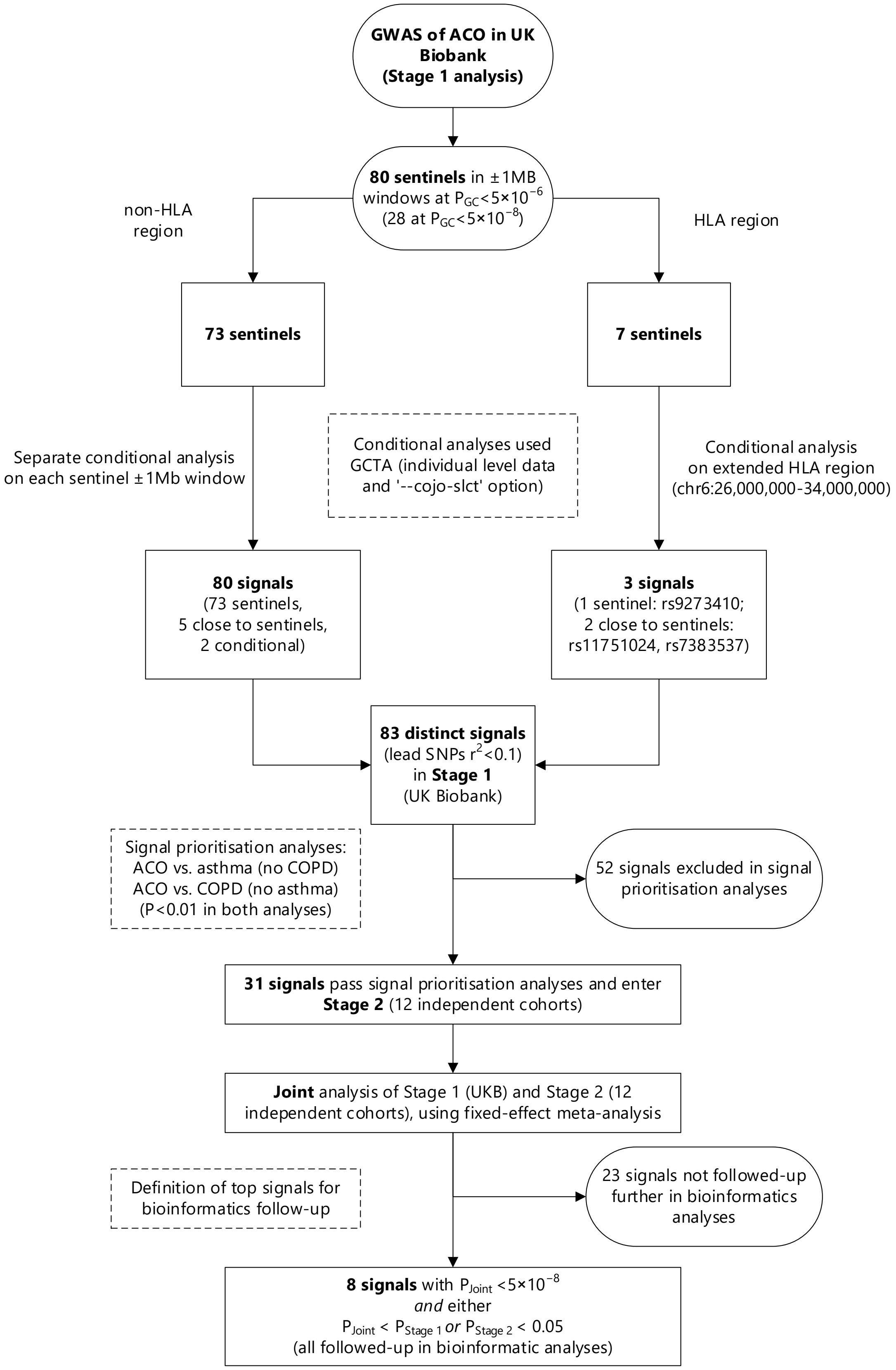

e-Figure 3 Scatterplots comparing effects in 31 signals taken forward for replication, with results when ACO cases are split into those with adult- (>25 years) and child-onset (<12 years) asthma

Comparison of effect sizes from analysis with ACO cases restricted to asthma onset >25 years (3755 cases) and analysis with ACO cases restricted to asthma onset <12 years (2903 cases).

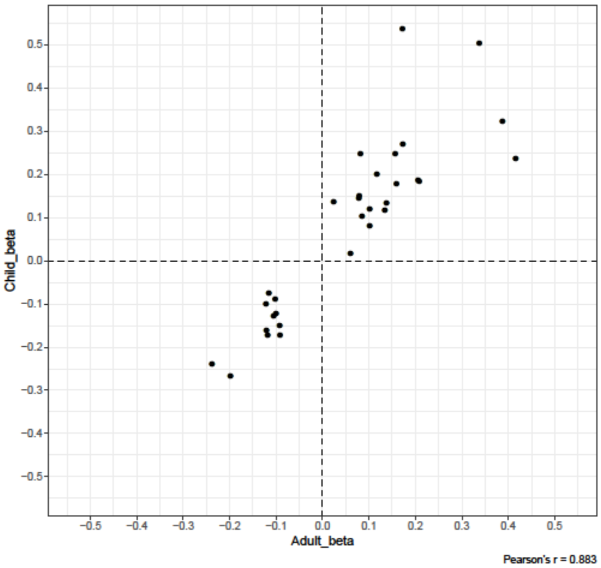

Comparison of effect sizes from main GWAS (8068 ACO, 40360 controls) and analysis with ACO cases restricted to asthma onset <12 years (2903 cases).

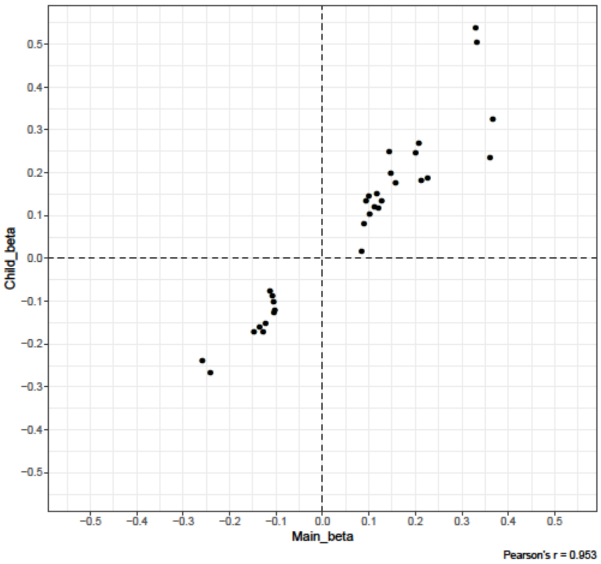

Comparison of effect sizes from main GWAS (8068 ACO, 40360 controls) and analysis with ACO cases restricted to asthma onset >25 years (3755 cases).

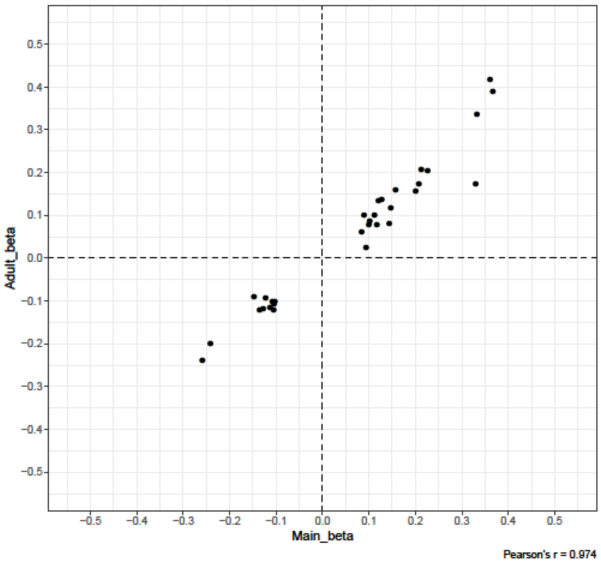

From left to right: comparison of effects (betas) of child-onset asthma ACO vs adult-onset asthma ACO cases; comparison of effects in child-onset ACO versus all ACO (e.g. main analysis); comparison of effects in adult-onset ACO versus all ACO (e.g. main analysis). Both subgroup analyses used the same 40,360 controls as used in the main analysis.

e-Figure 4 Scatterplots comparing effects in 31 signals taken forward for replication, with smoking-stratified results (by ever- and never-smokers)

Comparison of effect sizes from analysis of ever−smokers (4367 ACO, 17316 controls), with analysis of never−smokers (3701 ACO, 23044 controls).

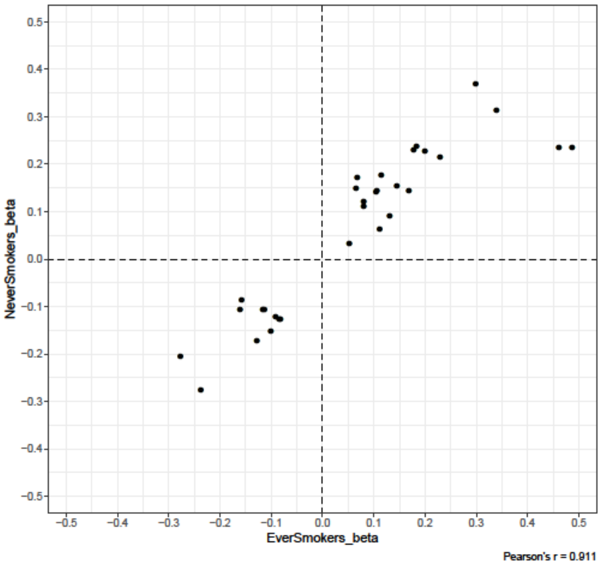

Comparison of effect sizes from main GWAS (8068 ACO, 40360 controls) and analysis of never−smokers (3701 ACO, 23044 controls).

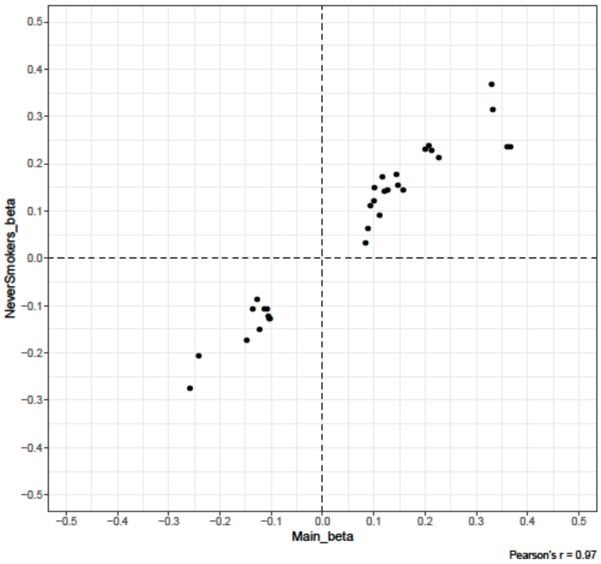

Comparison of effect sizes from main GWAS (8068 ACO, 40360 controls) and analysis of ever−smokers (4367 ACO, 17316 controls).

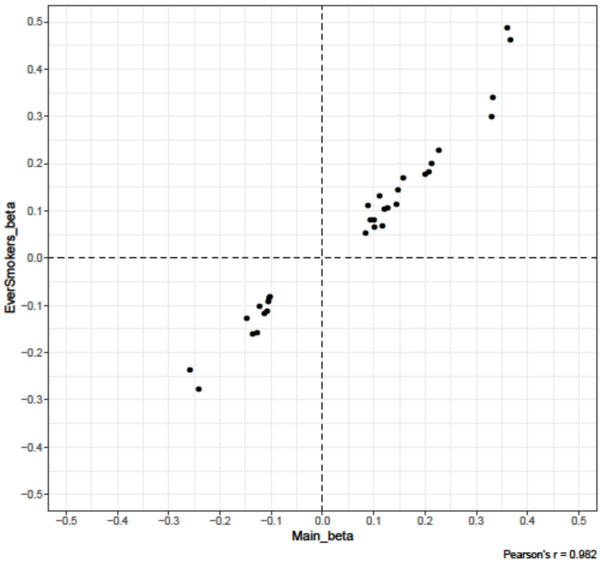

From left to right: comparison of effects (betas) of ACO versus controls (never-smokers) and ACO versus controls (ever-smokers); comparison of effects in ACO versus controls (never-smokers) versus all ACO (e.g. main analysis); comparison of effects in ACO versus controls (ever-smokers) versus all ACO (e.g. main analysis).

e-Figure 5 Region plots for eight signals taken forward for downstream analyses.

rs80101740

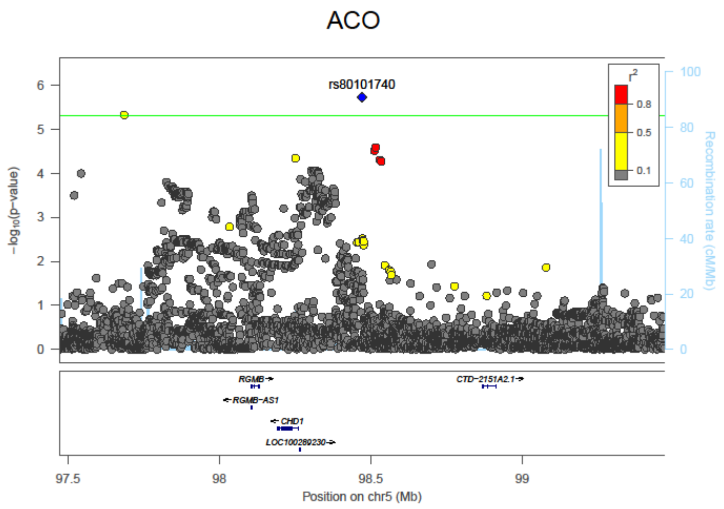

rs35570272

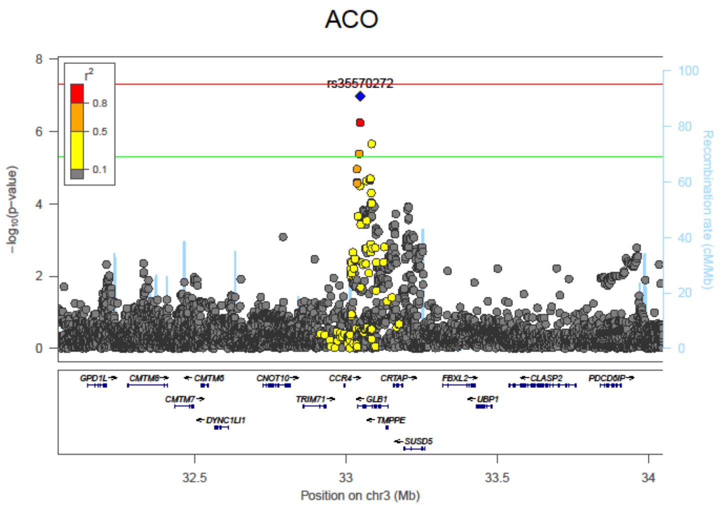

rs16903574

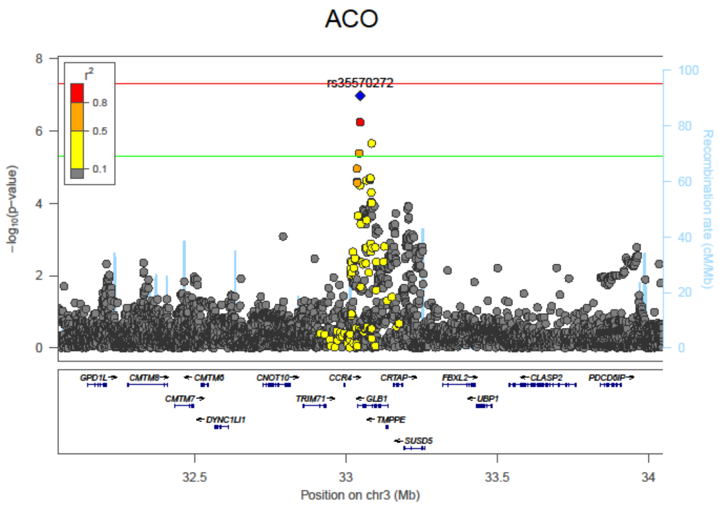

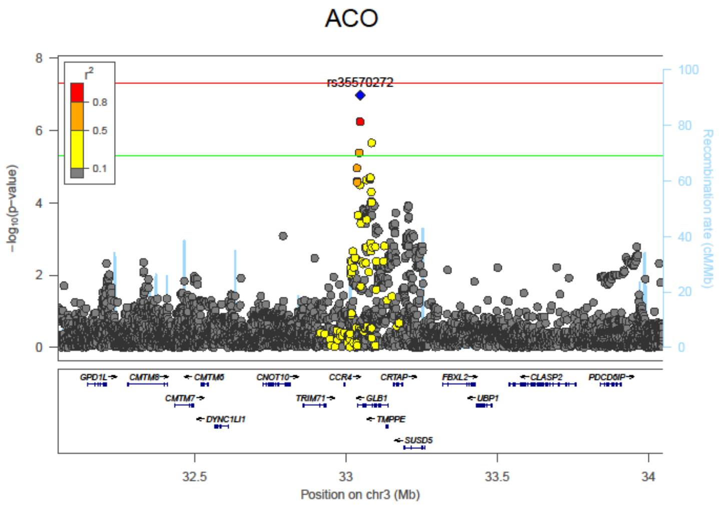

rs1837253

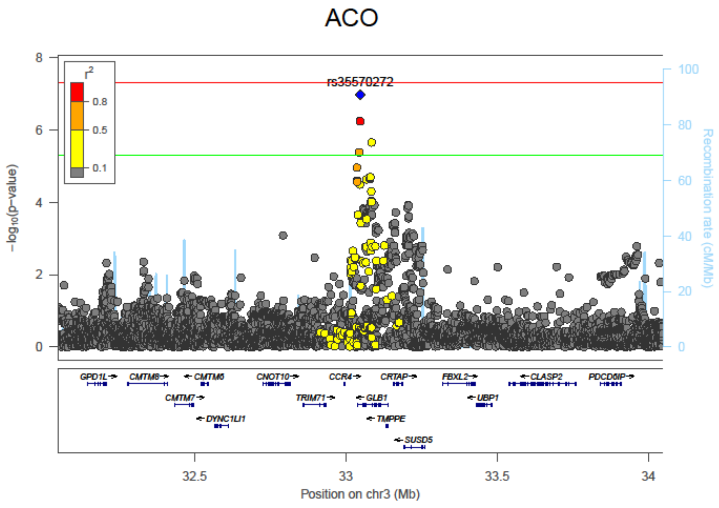

rs6787279

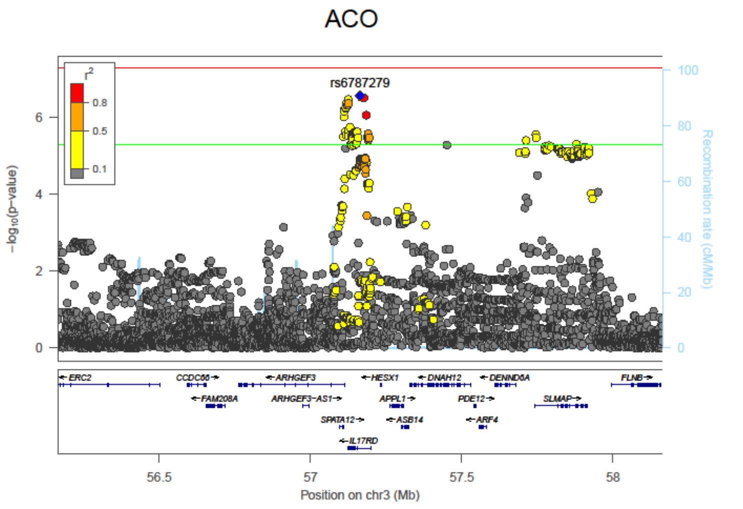

rs9273410

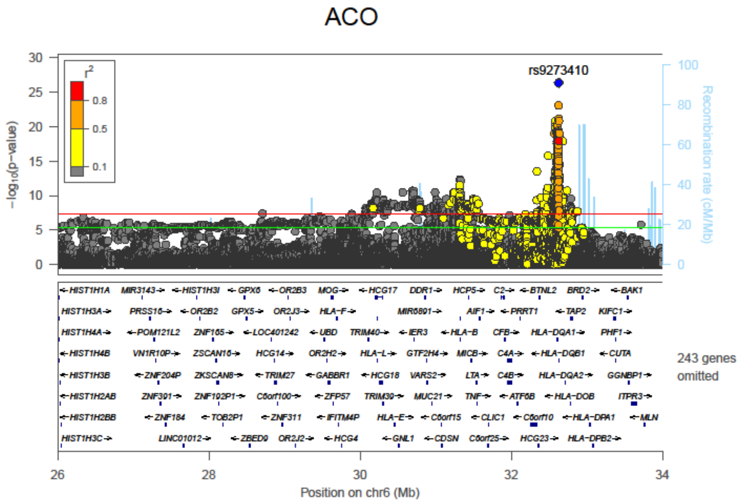

rs3749833

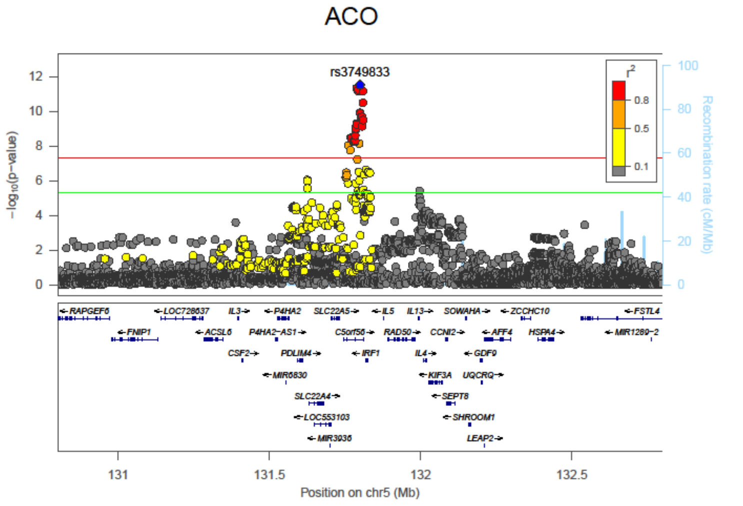

e-Figure 6 Forest plots for eight signals taken forward for downstream analyses (results from Stage 1 + Stage 2 joint analysis).

rs80101740

Meta−analysis of Stage 1 (UKB) + Stage 2 (12 studies) for variant rs80101740, (I^2=0%)

Cohort	OR	LCI	UCI	P	Cases	Controls
UKB	1.443	1.241	1.678	1.87e−06	8068	40360
COPDGeneNhw	1.557	0.901	2.690	0.113	591	1935
ECLIPSE	0.985	0.311	3.120	0.979	326	137
GenKOLS	1.169	0.550	2.486	0.685	425	707
SPIROMICS	1.348	0.517	3.517	0.541	197	433
LOVELACE						
FHS						
EPIC	1.485	0.810	2.723	0.201	281	10921
HUNT	1.627	0.941	2.812	0.081	1070	6788
CHS	1.332	0.447	3.966	0.607	89	1432
RS	2.470	0.909	6.715	0.076	114	4183
GS	0.997	0.558	1.783	0.993	374	11939
deCODE	1.213	0.629	2.338	0.564	631	5835
**Meta−Analysis**	**1.420**	**1.253**	**1.609**	**3.72e−08**	**12166**	**84670**

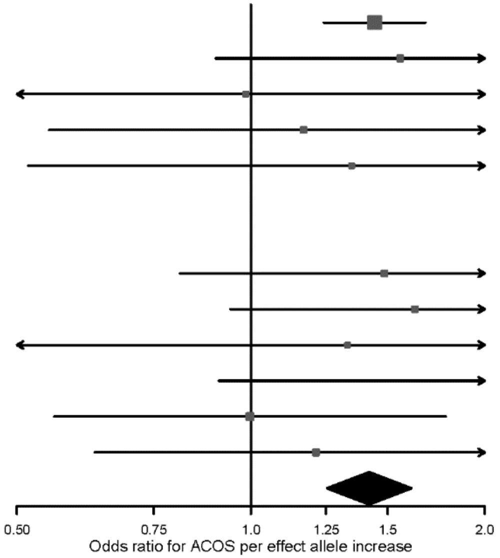

rs35570272

Meta−analysis of Stage 1 (UKB) + Stage 2 (12 studies) for variant rs35570272, (I^2=15.9%)

Cohort	OR	LCI	UCI	P	Cases	Controls
UKB	1.107	1.066	1.149	1.06e−07	8068	40360
COPDGeneNhw	1.082	0.943	1.241	0.261	591	1935
ECLIPSE	0.707	0.518	0.966	0.029	326	137
GenKOLS	1.202	0.984	1.468	0.071	425	707
SPIROMICS	0.927	0.728	1.180	0.536	197	433
LOVELACE	1.267	0.856	1.877	0.237	64	582
FHS	0.984	0.752	1.288	0.905	139	3707
EPIC	1.104	0.932	1.309	0.253	281	10921
HUNT	1.133	1.020	1.259	0.020	1070	6788
CHS	1.233	0.903	1.683	0.188	89	1432
RS	1.166	0.880	1.547	0.285	114	4183
GS	1.074	0.923	1.250	0.355	374	11939
deCODE	1.013	0.887	1.156	0.849	631	5835
**Meta−Analysis**	**1.097**	**1.064**	**1.130**	**2.44e−09**	**12369**	**88959**

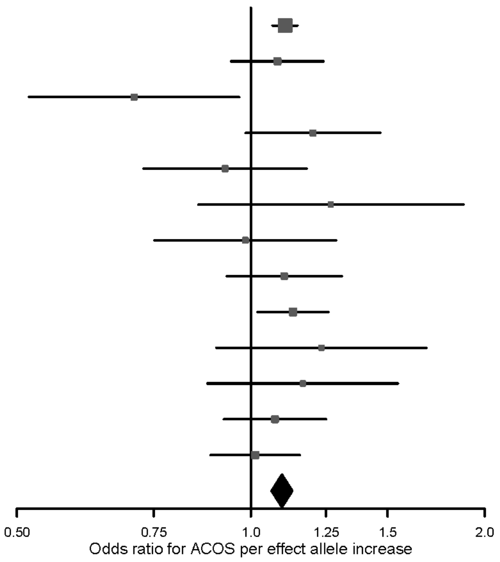

rs16903574

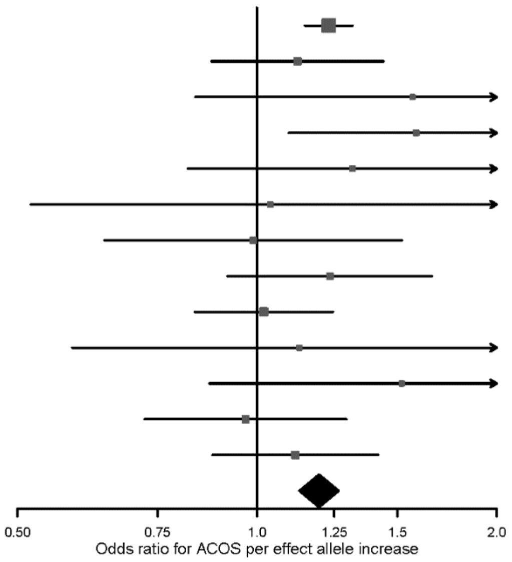

Cohort	OR	LCI	UCI	P	Cases	Controls
UKB	1.231	1.148	1.319	4.47e−09	8068	40360
COPDGeneNhw	1.124	0.877	1.442	0.356	591	1930
ECLIPSE	1.569	0.837	2.942	0.160	326	137
GenKOLS	1.585	1.096	2.292	0.014	425	707
SPIROMICS	1.318	0.819	2.122	0.256	197	433
LOVELACE	1.039	0.520	2.077	0.913	64	589
FHS	0.989	0.644	1.520	0.960	139	3707
EPIC	1.235	0.919	1.659	0.162	281	10921
HUNT	1.020	0.835	1.246	0.847	1070	6788
CHS	1.130	0.586	2.180	0.716	89	1432
RS	1.520	0.871	2.652	0.141	114	4183
GS	0.967	0.722	1.296	0.824	374	11939
deCODE	1.117	0.879	1.419	0.372	631	5835
**Meta−Analysis**	**1.197**	**1.131**	**1.266**	**3.8e−10**	**12369**	**88961**

rs2584662

Meta−analysis of Stage 1 (UKB) + Stage 2 (12 studies) for variant rs2584662, (I^2=39.6%)

Cohort	OR	LCI	UCI	P	Cases	Controls
UKB	0.901	0.868	0.935	3.2e−08	8068	40360
COPDGeneNhw	0.834	0.726	0.957	0.010	591	1935
ECLIPSE	1.294	0.953	1.757	0.098	326	137
GenKOLS	0.892	0.732	1.086	0.254	425	707
SPIROMICS	0.918	0.710	1.186	0.512	197	433
LOVELACE	1.035	0.690	1.552	0.868	64	589
FHS	0.858	0.645	1.143	0.296	139	3707
EPIC	0.835	0.700	0.996	0.045	281	10921
HUNT	0.926	0.836	1.026	0.143	1070	6788
CHS	1.080	0.790	1.476	0.631	89	1432
RS	1.155	0.876	1.522	0.306	114	4183
GS	1.069	0.920	1.242	0.383	374	11939
deCODE	1.025	0.899	1.169	0.706	631	5835
**Meta−Analysis**	**0.918**	**0.890**	**0.946**	**2.21e−08**	**12369**	**88966**

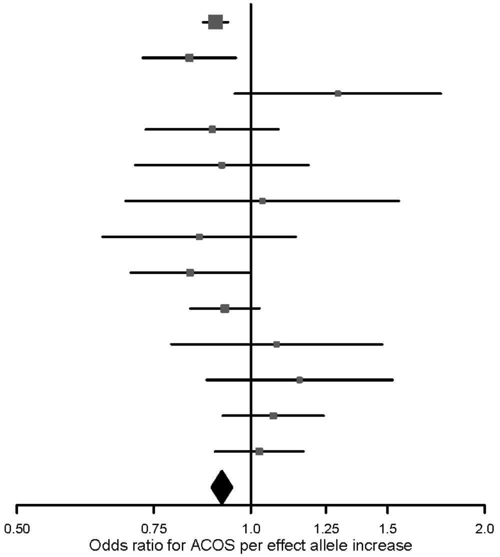

rs1837253

Meta−analysis of Stage 1 (UKB) + Stage 2 (12 studies) for variant rs1837253, (I^2=53.3%)

Cohort	OR	LCI	UCI	P	Cases	Controls
UKB	1.222	1.172	1.274	4.22e−21	8068	40360
COPDGeneNhw	1.046	0.898	1.218	0.564	591	1934
ECLIPSE	1.255	0.885	1.780	0.203	326	137
GenKOLS	1.057	0.856	1.306	0.607	425	707
SPIROMICS	0.880	0.651	1.189	0.406	197	433
LOVELACE	1.301	0.830	2.038	0.251	63	590
FHS	1.051	0.805	1.372	0.713	139	3707
EPIC	0.974	0.804	1.178	0.783	281	10921
HUNT	1.092	0.976	1.221	0.125	1070	6788
CHS	1.120	0.788	1.591	0.527	89	1432
RS	1.102	0.813	1.492	0.532	114	4183
GS	0.904	0.765	1.070	0.240	374	11939
deCODE	1.185	1.023	1.373	0.021	631	5835
**Meta−Analysis**	**1.162**	**1.124**	**1.202**	**1.53e−18**	**12368**	**88966**

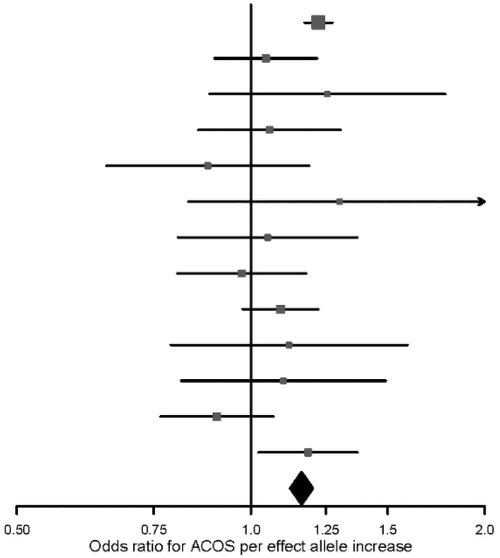

rs6787279

Meta−analysis of Stage 1 (UKB) + Stage 2 (12 studies) for variant rs6787279, (I^2=10.8%)

Cohort	OR	LCI	UCI	P	Cases	Controls
UKB	0.880	0.838	0.924	2.69e−07	8068	40360
COPDGeneNhw	0.854	0.710	1.027	0.094	591	1934
ECLIPSE	0.789	0.527	1.181	0.250	326	137
GenKOLS	0.933	0.725	1.201	0.588	425	707
SPIROMICS	0.780	0.562	1.084	0.139	197	433
LOVELACE	0.808	0.485	1.345	0.412	64	591
FHS	0.716	0.512	1.001	0.051	139	3707
EPIC	0.940	0.747	1.184	0.601	281	10921
HUNT	0.944	0.819	1.089	0.431	1070	6788
CHS	1.541	1.063	2.234	0.022	89	1432
RS	0.993	0.696	1.418	0.971	114	4183
GS	0.946	0.775	1.155	0.585	374	11939
deCODE	0.853	0.708	1.027	0.099	631	5835
**Meta−Analysis**	**0.889**	**0.855**	**0.926**	**7.87e−09**	**12369**	**88967**

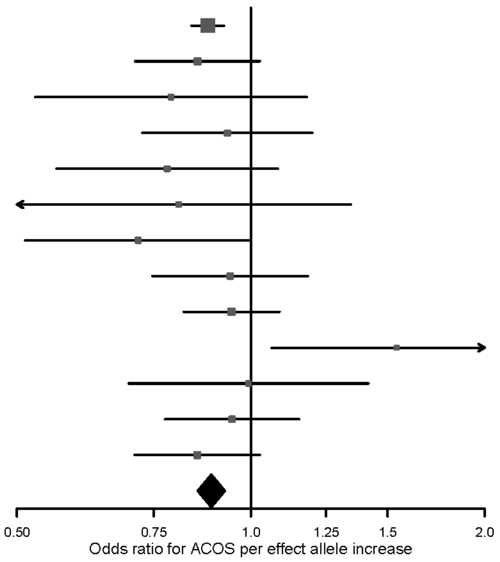

rs9273410

Meta−analysis of Stage 1 (UKB) + Stage 2 (12 studies) for variant rs9273410, (I^2=28.5%)

Cohort	OR	LCI	UCI	P	Cases	Controls
UKB	1.237	1.190	1.286	4.37e−27	8068	40360
COPDGeneNhw	0.973	0.812	1.167	0.772	591	1935
ECLIPSE	0.952	0.699	1.296	0.756	326	137
GenKOLS	1.152	0.873	1.519	0.318	425	707
SPIROMICS	1.228	0.960	1.569	0.102	197	433
LOVELACE	0.994	0.671	1.471	0.976	64	588
FHS	1.152	0.841	1.579	0.377	139	3707
EPIC	1.060	0.883	1.272	0.534	281	10921
HUNT	1.168	1.008	1.354	0.039	1070	6788
CHS	0.982	0.633	1.524	0.935	89	1432
RS	1.256	0.955	1.651	0.103	114	4183
GS	1.015	0.847	1.217	0.871	374	11939
deCODE	1.202	1.057	1.367	0.005	631	5835
**Meta−Analysis**	**1.199**	**1.161**	**1.239**	**9.19e−28**	**12369**	**88965**

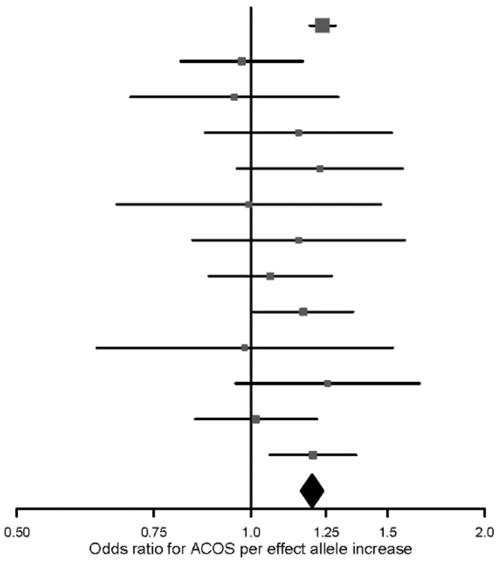

rs3749833

Meta−analysis of Stage 1 (UKB) + Stage 2 (12 studies) for variant rs3749833, (I^2=13.7%)

Cohort	OR	LCI	UCI	P	Cases	Controls
UKB	1.158	1.111	1.207	3.1e−12	8068	40360
COPDGeneNhw	1.064	0.913	1.242	0.426	591	1935
ECLIPSE	1.062	0.751	1.503	0.734	326	137
GenKOLS	1.050	0.848	1.300	0.652	425	707
SPIROMICS	0.953	0.718	1.265	0.737	197	433
LOVELACE	1.303	0.863	1.967	0.208	64	592
FHS	1.045	0.787	1.388	0.762	139	3707
EPIC	1.114	0.923	1.344	0.260	281	10921
HUNT	1.126	1.007	1.259	0.037	1070	6788
CHS	1.013	0.713	1.440	0.943	89	1432
RS	0.884	0.646	1.210	0.440	114	4183
GS	0.912	0.773	1.077	0.278	374	11939
deCODE	1.092	0.952	1.253	0.206	631	5835
**Meta−Analysis**	**1.123**	**1.086**	**1.160**	**9.37e−12**	**12369**	**88969**

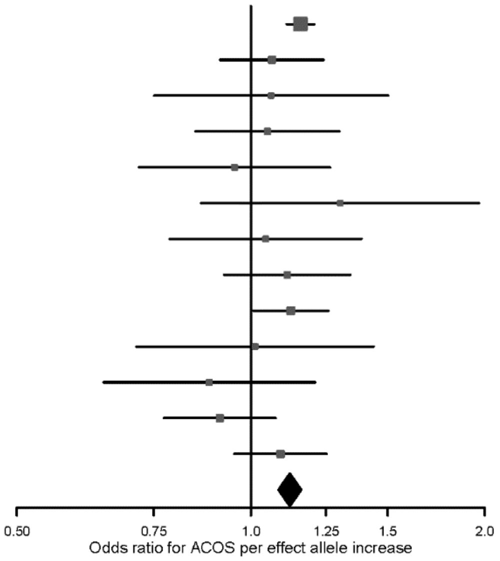

References

1. Moffatt MF, Kabesch M, Liang L, et al. Genetic variants regulating ORMDL3 expression contribute to the risk of childhood asthma. *Nature.* 2007;448(7152):470-473.
2. Himes BE, Hunninghake GM, Baurley JW, et al. Genome-wide association analysis identifies PDE4D as an asthma-susceptibility gene. *Am. J. Hum. Genet.* 2009;84(5):581-593.
3. Gudbjartsson DF, Bjornsdottir US, Halapi E, et al. Sequence variants affecting eosinophil numbers associate with asthma and myocardial infarction. *Nat. Genet.* 2009;41(3):342-347.
4. Moffatt MF, Gut IG, Demenais F, et al. A Large-Scale, Consortium-Based Genomewide Association Study of Asthma. *N. Engl. J. Med.* 2010;363(13):1211-1221.
5. Sleiman PM, Flory J, Imielinski M, et al. Variants of DENND1B associated with asthma in children. *N. Engl. J. Med.* 2010;362(1):36-44.
6. Ferreira MAR, Matheson MC, Duffy DL, et al. Identification of IL6R and chromosome 11q13.5 as risk loci for asthma. *The Lancet.* 2011;378(9795):1006-1014.
7. Hirota T, Takahashi A, Kubo M, et al. Genome-wide association study identifies three new susceptibility loci for adult asthma in the Japanese population. *Nat. Genet.* 2011;43(9):893-896.
8. Noguchi E, Sakamoto H, Hirota T, et al. Genome-wide association study identifies HLA-DP as a susceptibility gene for pediatric asthma in Asian populations. *PLoS Genet.* 2011;7(7):e1002170.
9. Torgerson DG, Ampleford EJ, Chiu GY, et al. Meta-analysis of genome-wide association studies of asthma in ethnically diverse North American populations. *Nat. Genet.* 2011;43(9):887-892.
10. Ramasamy A, Kuokkanen M, Vedantam S, et al. Genome-wide association studies of asthma in population-based cohorts confirm known and suggested loci and identify an additional association near HLA. *PLoS One.* 2012;7(9):e44008.
11. Wan YI, Shrine NRG, Soler Artigas M, et al. Genome-wide association study to identify genetic determinants of severe asthma. *Thorax.* 2012;67:762-768.
12. Bonnelykke K, Sleiman P, Nielsen K, et al. A genome-wide association study identifies CDHR3 as a susceptibility locus for early childhood asthma with severe exacerbations. *Nat. Genet.* 2014;46(1):51-55.
13. Ferreira MAR, Matheson MC, Tang CS, et al. Genome-wide association analysis identifies 11 risk variants associated with the asthma with hay fever phenotype. *J. Allergy Clin. Immunol.* 2014;133(6):1564-1571.
14. Almoguera B, Vazquez L, Mentch F, et al. Identification of Four Novel Loci in Asthma in European and African American Populations. *Am. J. Respir. Crit. Care Med.* 2016.
15. Pickrell JK, Berisa T, Liu JZ, Segurel L, Tung JY, Hinds DA. Detection and interpretation of shared genetic influences on 42 human traits. *Nat. Genet.* 2016;48(7):709-717.
16. Ferreira MA, Vonk JM, Baurecht H, et al. Shared genetic origin of asthma, hay fever and eczema elucidates allergic disease biology. *Nat. Genet.* 2017;49(12):1752-1757.
17. Demenais F, Margaritte-Jeannin P, Barnes KC, et al. Multiancestry association study identifies new asthma risk loci that colocalize with immune-cell enhancer marks. *Nat. Genet.* 2018;50(1):42-53.
18. Hayden LP, Cho MH, Raby BA, Beaty TH, Silverman EK, Hersh CP. Childhood asthma is associated with COPD and known asthma variants in COPDGene: a genome-wide association study. *Respir. Res.* 2018;19(1):209.
19. Zhu Z, Lee PH, Chaffin MD, et al. A genome-wide cross-trait analysis from UK Biobank highlights the shared genetic architecture of asthma and allergic diseases. *Nat. Genet.* 2018;50(6):857-864.
20. Dahlin A, Sordillo JE, Ziniti J, et al. Large-scale, multiethnic genome-wide association study identifies novel loci contributing to asthma susceptibility in adults. *J. Allergy Clin. Immunol.* 2019;143(4):1633-1635.

21. Pividori M, Schoettler N, Nicolae DL, Ober C, Im HK. Shared and distinct genetic risk factors for childhood-onset and adult-onset asthma: genome-wide and transcriptome-wide studies. *Lancet Respir. Med.* 2019;7(6):509-522.
22. Hancock DB, Eijgelsheim M, Wilk JB, et al. Meta-analyses of genome-wide association studies identify multiple loci associated with pulmonary function. *Nat. Genet.* 2010;42(1):45-52.
23. Repapi E, Sayers I, Wain LV, et al. Genome-wide association study identifies five loci associated with lung function. *Nat. Genet.* 2010;42(1):36-44.
24. Soler Artigas M, Loth DW, Wain LV, et al. Genome-wide association and large-scale follow up identifies 16 new loci influencing lung function. *Nat. Genet.* 2011;43(11):1082-1090.
25. Castaldi PJ, Cho MH, Litonjua AA, et al. The association of genome-wide significant spirometric loci with chronic obstructive pulmonary disease susceptibility. *Am. J. Respir. Cell Mol. Biol.* 2011;45(6):1147-1153.
26. Soler Artigas M, Wain LV, Repapi E, et al. Effect of five genetic variants associated with lung function on the risk of chronic obstructive lung disease, and their joint effects on lung function. *Am. J. Respir. Crit. Care Med.* 2011;184(7):786-795.
27. Wilk JB, Shrine NR, Loehr LR, et al. Genome-wide association studies identify CHRNA5/3 and HTR4 in the development of airflow obstruction. *Am. J. Respir. Crit. Care Med.* 2012;186(7):622-632.
28. Loth DW, Artigas MS, Gharib SA, et al. Genome-wide association analysis identifies six new loci associated with forced vital capacity. *Nat. Genet.* 2014;46(7):669-677.
29. Cho MH, McDonald ML, Zhou X, et al. Risk loci for chronic obstructive pulmonary disease: a genome-wide association study and meta-analysis. *Lancet Respir. Med.* 2014;2(3):214-225.
30. Wain LV, Shrine N, Miller S, et al. Novel insights into the genetics of smoking behaviour, lung function, and chronic obstructive pulmonary disease (UK BiLEVE): a genetic association study in UK Biobank. *Lancet Respir. Med.* 2015;3(10):769-781.
31. Lutz SM, Cho MH, Young K, et al. A genome-wide association study identifies risk loci for spirometric measures among smokers of European and African ancestry. *BMC Genet.* 2015;16(1):138.
32. Soler Artigas M, Wain LV, Miller S, et al. Sixteen new lung function signals identified through 1000 Genomes Project reference panel imputation. *Nat. Commun.* 2015;6:8658.
33. Hobbs BD, Parker MM, Chen H, et al. Exome Array Analysis Identifies a Common Variant in IL27 Associated with Chronic Obstructive Pulmonary Disease. *Am. J. Respir. Crit. Care Med.* 2016;194(1):48-57.
34. Hobbs BD, de Jong K, Lamontagne M, et al. Genetic loci associated with chronic obstructive pulmonary disease overlap with loci for lung function and pulmonary fibrosis. *Nat. Genet.* 2017;49(3):426-432.
35. Wain LV, Shrine N, Artigas MS, et al. Genome-wide association analyses for lung function and chronic obstructive pulmonary disease identify new loci and potential druggable targets. *Nat. Genet.* 2017;49(3):416-425.
36. Wyss AB, Sofer T, Lee MK, et al. Multiethnic meta-analysis identifies ancestry-specific and cross-ancestry loci for pulmonary function. *Nat. Commun.* 2018;9(1):2976.
37. Jackson VE, Latourelle JC, Wain LV, et al. Meta-analysis of exome array data identifies six novel genetic loci for lung function. *Wellcome Open Res.* 2018;3:4.
38. Shrine N, Portelli MA, John C, et al. Moderate-to-severe asthma in individuals of European ancestry: a genome-wide association study. *Lancet Respir. Med.* 2019;7(1):20-34.
39. Sakornsakolpat P, Prokopenko D, Lamontagne M, et al. Genetic landscape of chronic obstructive pulmonary disease identifies heterogeneous cell-type and phenotype associations. *Nat. Genet.* 2019;51(3):494-505.

40. Johansson A, Rask-Andersen M, Karlsson T, Ek WE. Genome-wide association analysis of 350 000 Caucasians from the UK Biobank identifies novel loci for asthma, hay fever and eczema. *Hum. Mol. Genet.* 2019;28(23):4022-4041.
41. Olafsdottir TA, Theodors F, Bjarnadottir K, et al. Eighty-eight variants highlight the role of T cell regulation and airway remodeling in asthma pathogenesis. *Nat. Commun.* 2020;11(1):393.
42. Fried LP, Borhani NO, Enright P, et al. The Cardiovascular Health Study: design and rationale. *Ann. Epidemiol.* 1991;1(3):263-276.
43. Regan EA, Hokanson JE, Murphy JR, et al. Genetic epidemiology of COPD (COPDGene) study design. *COPD.* 2010;7(1):32-43.
44. Gudbjartsson DF, Helgason H, Gudjonsson SA, et al. Large-scale whole-genome sequencing of the Icelandic population. *Nat. Genet.* 2015;47(5):435-444.
45. Gulcher JR, Kristjánsson K, Gudbjartsson H, Stefánsson K. Protection of privacy by third-party encryption in genetic research in Iceland. *Eur. J. Hum. Genet.* 2000;8(10):739-742.
46. Vestbo J, Anderson W, Coxson HO, et al. Evaluation of COPD Longitudinally to Identify Predictive Surrogate End-points (ECLIPSE). *Eur. Respir. J.* 2008;31(4):869-873.
47. Day N, Oakes S, Luben R, et al. EPIC-Norfolk: study design and characteristics of the cohort. European Prospective Investigation of Cancer. *Br. J. Cancer.* 1999;80 Suppl 1:95-103.
48. Henriksen AH, Langhammer A, Steinshamn S, Mai XM, Brumpton BM. The Prevalence and Symptom Profile of Asthma-COPD Overlap: The HUNT Study. *COPD.* 2018;15(1):27-35.
49. Smith BH, Campbell A, Linksted P, et al. Cohort Profile: Generation Scotland: Scottish Family Health Study (GS:SFHS). The study, its participants and their potential for genetic research on health and illness. *Int. J. Epidemiol.* 2013;42(3):689-700.
50. Sørheim IC, Johannessen A, Grydeland TB, Omenaas ER, Gulsvik A, Bakke PS. Case-control studies on risk factors for chronic obstructive pulmonary disease: how does the sampling of the cases and controls affect the results? *Clin. Respir. J.* 2010;4(2):89-96.
51. Banda Y, Kvale MN, Hoffmann TJ, et al. Characterizing Race/Ethnicity and Genetic Ancestry for 100,000 Subjects in the Genetic Epidemiology Research on Adult Health and Aging (GERA) Cohort. *Genetics.* 2015;200(4):1285-1295.
52. Åsvold BO, Langhammer A, Rehn TA, et al. Cohort Profile Update: The HUNT Study, Norway. *medRxiv.* 2021:2021.2010.2012.21264858.
53. Sood A, Stidley CA, Picchi MA, et al. Difference in airflow obstruction between Hispanic and non-Hispanic White female smokers. *COPD.* 2008;5(5):274-281.
54. Hofman A, Brusselle GG, Darwish Murad S, et al. The Rotterdam Study: 2016 objectives and design update. *Eur. J. Epidemiol.* 2015;30(8):661-708.
55. Kreiner-Møller E, Medina-Gomez C, Uitterlinden AG, Rivadeneira F, Estrada K. Improving accuracy of rare variant imputation with a two-step imputation approach. *Eur. J. Hum. Genet.* 2015;23(3):395-400.
56. Couper D, LaVange LM, Han M, et al. Design of the Subpopulations and Intermediate Outcomes in COPD Study (SPIROMICS). *Thorax.* 2014;69(5):491-494.
57. Li X, Ortega VE, Ampleford EJ, et al. Genome-wide association study of lung function and clinical implication in heavy smokers. *BMC Med. Genet.* 2018;19(1):134.
58. Shrine N, Guyatt AL, Erzurumluoglu AM, et al. New genetic signals for lung function highlight pathways and chronic obstructive pulmonary disease associations across multiple ancestries. *Nat. Genet.* 2019;51:481-493.
59. Bycroft C, Freeman C, Petkova D, et al. The UK Biobank resource with deep phenotyping and genomic data. *Nature.* 2018;562(7726):203-209.
60. John C, Reeve NF, Free RC, et al. Cohort Profile: Extended Cohort for E-health, Environment and DNA (EXCEED). *Int. J. Epidemiol.* 2019;48(3):678-679j.
61. Wakefield J. Reporting and interpretation in genome-wide association studies. *Int. J. Epidemiol.* 2008;37(3):641-653.

62. Wang K, Li M, Hakonarson H. ANNOVAR: Functional annotation of genetic variants from next-generation sequencing data. *Nucleic Acids Res.* 2010;38:e164.
63. Kumar P, Henikoff S, Ng PC. Predicting the effects of coding non-synonymous variants on protein function using the SIFT algorithm. *Nat. Protoc.* 2009;4(7):1073-1081.
64. Shihab HA, Gough J, Cooper DN, et al. Predicting the functional, molecular, and phenotypic consequences of amino acid substitutions using hidden Markov models. *Hum. Mutat.* 2013;34(1):57-65.
65. Adzhubei IA, Schmidt S, Peshkin L, et al. A method and server for predicting damaging missense mutations. *Nat. Methods.* 2010;7(4):248-249.
66. Rentzsch P, Witten D, Cooper GM, Shendure J, Kircher M. CADD: predicting the deleteriousness of variants throughout the human genome. *Nucleic Acids Res.* 2019;47(D1):D886-d894.
67. Hao K, Bosse Y, Nickle DC, et al. Lung eQTLs to help reveal the molecular underpinnings of asthma. *PLoS Genet.* 2012;8(11):e1003029.
68. Lamontagne M, Couture C, Postma DS, et al. Refining susceptibility loci of chronic obstructive pulmonary disease with lung eqtls. *PLoS One.* 2013;8(7):e70220.
69. Obeidat M, Miller S, Probert K, et al. GSTCD and INTS12 regulation and expression in the human lung. *PLoS One.* 2013;8(9):e74630.
70. GTEx Consortium. The Genotype-Tissue Expression (GTEx) project. *Nat. Genet.* 2013;45(6):580-585.
71. Giambartolomei C, Vukcevic D, Schadt EE, et al. Bayesian Test for Colocalisation between Pairs of Genetic Association Studies Using Summary Statistics. *PLoS Genet.* 2014;10(5):e1004383.
72. Lamontagne M, Berube JC, Obeidat M, et al. Leveraging lung tissue transcriptome to uncover candidate causal genes in COPD genetic associations. *Hum. Mol. Genet.* 2018;27(10):1819-1829.
73. Martin JS, Xu Z, Reiner AP, et al. HUGIn: Hi-C Unifying Genomic Interrogator. *Bioinformatics.* 2017;33(23):3793-3795.
74. Schmitt AD, Hu M, Jung I, et al. A Compendium of Chromatin Contact Maps Reveals Spatially Active Regions in the Human Genome. *Cell Rep.* 2016;17(8):2042-2059.
75. Ay F, Bailey TL, Noble WS. Statistical confidence estimation for Hi-C data reveals regulatory chromatin contacts. *Genome Res.* 2014;24(6):999-1011.
76. Staley JR, Blackshaw J, Kamat MA, et al. PhenoScanner: a database of human genotype-phenotype associations. *Bioinformatics.* 2016;32(20):3207-3209.

e-Table 1: Details of ACO case and control definitions, and covariate details in Stage 2 cohorts

y=years; L=litres; FEV_1=forced expiratory volume in 1 second; FEV_1/FVC=ratio of FEV_1 to forced vital capacity (FVC); Aa=African American; Nhw=non-Hispanic white

Ancestry & Analysis Group	Study	Subgroup	N	Females, %	Age range, y	Mean age, y (SD)	Ever smokers, %	Spirometry available, %	Mean FEV_1, L (SD)	Mean % predicted FEV_1 (SD)	Mean FEV_1/FVC (SD)
African-American	COPDGene (Aa)	Cases	297	55.9%	45-78	58.2 (8.2)	100.0%	100.0%	1.33 (0.58)	51.0 (17.6)	0.52 (0.12)
		Controls	1335	36.3%	40-80	52.7 (5.9)	100.0%	100.0%	2.88 (0.65)	99.0 (12.3)	0.80 (0.05)
		All	1632	39.8%	40-80	53.7 (6.78)	100.0%	100.0%	2.59 (0.87)	90.3 (22.9)	0.75 (0.13)
European (main meta-analysis only; COPD defined based on spirometry)	deCODE	Cases	631	63.4%	1900-1985**	1930** (12.3)	NA	100.0%	NA	60.0 (15.3)	0.56 (0.10)
		Controls	5835	56.7%	1900-2000	1950 (70.7)	NA	100.0%	NA	141.6 (46.1)	0.77 (0.17)
		All	6466	57.3%	NA	NA	NA	100.0%	NA	133.6 (50.3)	0.75 (0.18)
	HUNT	Cases	1070	43.7%	19-84	58.1 (13.5)	81.5%	100.0%	1.99 (0.79)	63.9 (15.8)	0.60 (0.10)
		Controls	6788	56.1%	19-89	43.2 (14.2)	56.7%	100.0%	3.48 (0.88)	100.1 (11.3)	0.81 (0.05)
		All	7858	54.4%	19-89	45.2 (14.99)	60.1%	100.0%	3.28 (1.01)	95.2 (17.2)	0.78 (0.09)
	Rotterdam Study (RS)	Cases	114	57.9%	50-95	69.8 (8.8)	72.8%	100.0%	1.73*	69.5*	0.63*
		Controls	4183	58.1%	48-103	71.5 (10.5)	63.2%	100.0%	2.77*	108.6*	0.79*
		All	4297	58.1%	48-103	71.5 (10.5)	63.4%	100.0%	2.74*	107.5*	0.78*
European (main and sensitivity meta-analyses; COPD defined based on spirometry)	CHS	Cases	89	64.0%	65-85	71.5 (4.7)	67.4%	100.0%	1.38 (0.54)	57.0 (16.7)	0.55 (0.10)
		Controls	1432	67.9%	65-92	71.8 (5.1)	42.3%	100.0%	2.36 (0.55)	101.5 (13.0)	0.76 (0.04)
		All	1521	67.7%	65-92	71.8 (5.1)	43.8%	100.0%	2.30 (0.60)	98.9 (16.9)	0.75 (0.07)
	COPDGene (Nhw)	Cases	591	55.2%	45-80	63.2 (8.3)	100.0%	100.0%	1.36 (0.61)	47.5 (17.8)	0.48 (0.13)
		Controls	1935	47.8%	45-80	59.5 (8.8)	100.0%	100.0%	3.01 (0.70)	97.4 (11.1)	0.78 (0.05)
		All	2526	49.5%	45-80	60.4 (8.8)	100.0%	100.0%	2.62 (0.98)	85.7 (24.8)	0.71 (0.15)
	ECLIPSE	Cases	326	44.2%	40-75	62.5 (7.7)	100.0%	100.0%	1.27 (0.50)	46.7 (15.7)	0.44 (0.12)
		Controls	137	37.2%	40-75	57.1 (9.5)	100.0%	100.0%	3.36 (0.80)	108.7 (13.7)	0.80 (0.06)
		All	463	42.1%	40-75	60.9 (8.6)	100.0%	100.0%	1.88 (1.13)	65.0 (32.1)	0.54 (0.19)

Ancestry & Analysis Group	Study	Subgroup	N	Females, %	Age range, y	Mean age, y (SD)	Ever smokers, %	Spirometry available, %	Mean FEV_1, L (SD)	Mean % predicted FEV_1 (SD)	Mean FEV_1/FVC (SD)
	EPIC-Norfolk	Cases	281	42.0%	43-78	62.7 (8.4)	66.5%	100.0%	1.59 (0.60)	53.9 (14.9)	0.59 (0.09)
		Controls	10921	53.8%	39-79	58.4 (9.0)	49.9%	100.0%	2.80 (0.66)	94.9 (10.1)	0.85 (0.08)
		All	11202	53.5%	39-79	58.5 (9.1)	50.3%	100.0%	2.77 (0.68)	93.9 (12.1)	0.84 (0.09)
	FHS	Cases	139	54.7%	29-80	56.6 (11.5)	72.7%	100.0%	2.01 (0.72)	64 (12)	0.58 (0.08)
		Controls	3707	53.4%	19-86	46.7 (12.8)	47.3%	100.0%	3.43 (0.83)	102 (11)	0.78 (0.05)
		All	3846	53.5%	19-86	47.1 (12.9)	48.2%	100.0%	3.38 (0.87)	100 (13)	0.78 (0.06)
	GenKOLS	Cases	425	49.6%	40-85	66.0 (9.9)	100.0%	100.0%	1.36 (0.61)	46.5 (16.7)	0.48 (0.13)
		Controls	707	47.7%	40-85	55.6 (9.7)	100.0%	100.0%	3.28 (0.73)	95.3 (9.2)	0.79 (0.04)
		All	1132	48.4%	40-85	59.5 (11.0)	100.0%	100.0%	2.56 (1.16)	77.0 (26.8)	0.68(0.17)
	Generation Scotland (GS)	Cases	374	61.5%	18-83	49.2 (15.2)	54.8%	100.0%	2.18 (0.68)	68.2 (11.2)	0.66 (0.12)
		Controls	11939	58.2%	18-94	45.8 (14.1)	43.7%	100.0%	3.22 (0.81)	96.6 (11.9)	0.8 (0.06)
		All	12313	58.3%	18-94	45.9 (14.2)	44.0%	100.0%	3.187 (0.83)	96.3 (12.9)	0.79 (0.07)
	LOVELACE	Cases	64	82.8%	41-75	60.2 (8.7)	100.0%	100.0%	1.65 (0.47)	61.1 (13.7)	0.57 (0.10)
		Controls	592	78.5%	40-75	54.3 (9.3)	100.0%	100.0%	2.88 (0.67)	97.5 (11.0)	0.79 (0.04)
		All	656	79.0%	40-75	54.9 (9.4)	100.0%	100.0%	2.76 (0.75)	93.9 (15.6)	0.76 (0.08)
	SPIROMICS	Cases	197	51.8%	41-80	64.5 (7.7)	100.0%	100.0%	2.86 (0.62)	51.9 (17.4)	0.48 (0.12)
		Controls	433	51.7%	40-80	63.2 (9.0)	100.0%	100.0%	2.94 (0.61)	98.2 (11.5)	0.77 (0.04)
		All	630	51.7%	40-80	63.6 (8.6)	100.0%	100.0%	2.91 (0.62)	83.7 (25.5)	0.68 (0.16)
European (sensitivity meta-analysis only; COPD defined based on spirometry and/or EHR)	deCODE	Cases	1888	62.7%	1895-1975	1931 (13.5)	NA	0.0%	NA	NA	NA
		Controls	5835	56.7%	1900-2000	1950 (70.7)	NA	100.0%	NA	141.6 (46.1)	0.77 (0.17)
		All	7723	58.1%	NA	NA	NA	NA	NA	NA	NA
	FINNCAD/Generisk	Cases	321	47.0%	34-75	58.8 (7.7)	100.0%	86.6%	1.60 (0.61)	49.2 (15.7)	0.59 (0.14)
		Controls	4948	63.7%	44-66	55.6 (5.8)	16.9%	0.0%	NA	NA	NA
		All	5269	62.7%	34-75	57.2 (7.2)	22.0%	NA	NA	NA	NA
	GERA	Cases	1237	66.9%	19-≥90	67.1 (12.3)	58.7%	0.0%	NA	NA	NA
		Controls	59457	59.0%	18-≥90	61.9 (13.2)	42.7%	0.0%	NA	NA	NA

Ancestry & Analysis Group	Study	Subgroup	N	Females, %	Age range, y	Mean age, y (SD)	Ever smokers, %	Spirometry available, %	Mean FEV_1, L (SD)	Mean % predicted FEV_1 (SD)	Mean FEV_1/FVC (SD)
		All	60694	59.1%	18-≥90	62.0 (13.2)	43.0%	0.0%	NA	NA	NA
	HUNT	Cases	2005	48.2%	19-90	58.8 (14.0)	79.9%	54.9%	2.01 (0.80)	64.8 (16.6)	0.61 (0.11)
		Controls	6788	56.1%	19-89	43.2 (14.2)	56.7%	100.0%	3.48 (0.88)	100.1 (11.3)	0.81 (0.05)
		All	8793	54.3%	19-90	46.7 (15.6)	62.0%	89.7%	3.28 (1.01)	95.2 (17.2)	0.78 (0.09)
	MGB Biobank	Cases	617	59.6%	25-97	67.2 (12.8)	62.6%	0.0%	NA	NA	NA
		Controls	7578	56.0%	19-102	58.9 (16.5)	42.9%	0.0%	NA	NA	NA
		All	8195	56.3%	19-102	59.5 (16.4)	44.4%	0.0%	NA	NA	NA
	Rotterdam Study (RS)	Cases	186	56.5%	50-98	69.6	74.7%	71.5%	1.93*	75.5*	0.65*
		Controls	4183	58.1%	48-103	71.5	63.2%	100.0%	2.77*	108.7*	0.79*
		All	4369	58.0%	48-103	71.5	58.0%	98.8%	2.74*	107.3*	0.78*

*Spirometry details from the Rotterdam Study (RS) in this table are from study visit RS3; all study visits (RS1, RS2, RS3) were used for identification of cases and controls.

**Year of birth given for deCODE study.

e-Table 2: Details of genotyping and imputation in Stage 2 cohorts

HWE=Hardy-Weinberg Equilibrium; MAF=Minor Allele Frequency; PCs=principal components; HRC=Haplotype Reference Consortium; GRM=genetic relatedness matrix; GEE=generalised estimating equations; Aa=African American; Nhw=non-Hispanic white

Study	Pre-imputation filtering metrics					Genotyping platform	Imputation		Analysis details			
	Individual call rate	SNP call rate	SNP HWE P-value filter	SNP MAF filter	Other filters		Software	Reference panel	Genotype data	Covariates	Relatedness	Association software
CHS	95%	97%	1.00E-05	NA	>2 duplicate errors or Mendelian inconsistencies, heterozygote frequency = 0, SNP not found in HapMap. Individuals additionally excluded if genotype was discordant with known sex or prior genotyping.	Illumina 370CNV BeadChip	Michigan imputation server	HRC 1.1	Dosages	Study clinic, 3 PCs	NA	R
COPDGene (Aa)	95%	95%	1.00E-03	0.05	AT/GC SNPs	Illumina Quad610	Umich Imputation Server	1000 Genomes v3	Dosages	Age, sex (all smokers).	All unrelated, pi-hat > 0.125.	R
COPDGene (Nhw)	95%	95%	1.00E-03	0.05	AT/GC SNPs	Illumina Quad610	Umich Imputation Server	HRC 1.1	Dosages	Age, sex (all smokers).	All unrelated, pi-hat > 0.125.	R
deCODE	98%	80%	1.00E-07	<0.1 / (2x sample count)	Imputation INFO greater than or equal to 0.8.	Illumina HumanHap300, HumanCNV370, HumanCNV340, HumanHap610, HumanHap1M, HumanHap660, Omni-1, Omni 2.5, Omni 5, Omni 23 or Omni Express	Software developed at deCODE genetics (Gudbjartsson, D.F. et al. Nat Genet 47, 435-44 (2015))	See footnote[1]	Dosages	Sex, age, county of origin	See footnote[2]	Software developed at deCODE genetics (Gudbjartsson, D.F. et al. Nat Genet 47, 435-44 (2015))
ECLIPSE	95%	95%	1.00E-03	0.05	AT/GC SNPs	Illumina HumanHap 550	Umich Imputation Server	HRC 1.1	Dosages	Age, sex (all smokers).	All unrelated, pi-hat > 0.125.	R
EPIC-Norfolk	95%	95%	1.00E-06	0	Clusters pass Affymetrix SNPolisher standard tests and thresholds. MAF not significantly affected by plate (exclusion threshold p<9.5e-8 and <20% difference in MAF), Y and MT chromosome removed;	Affymetrix Axiom UKBiobank	IMPUTE4 and Sanger Imputation Service	HRCv1 and UK10K+1000Gphase 3	Posterior probabilities	First 10 principal components	1st and 2nd degree related individuals were excluded	SNPTEST 2.5.4-beta3

Study	Pre-imputation filtering metrics					Genotyping platform	Imputation		Analysis details			
	Individual call rate	SNP call rate	SNP HWE P-value filter	SNP MAF filter	Other filters		Software	Reference panel	Genotype data	Covariates	Relatedness	Association software
					duplicates and unflippable SNPs removed.							
FHS	97%	96.9 %	1.00E-06	0.01	Remove if number of Mendelian errors >=1000; remove variants at locations that did not map to GRCh37.	Affymetrix 500K mapping array plus Affymetrix 50K supplemental array	HRC server	HRC	Dosages	2 principal components	Used GEE to account for family relatedness	geepack R package
FINNCAD / Generisk	95%	98%	1.00E-06	MAC >=3	Outliers greater than 4 SDs for PC1 and PC2	HumanCoreExome-24v1-0_A	Beagle 4.1	Finnish SISu v3 (dx.doi.org/10.17504/protocols.io.nmndc5e)	Dosages	Age, sex, smoking, 10 principal components	Kinship > 0.3 excluded	Plink 2.0
GenKOLS	95%	95%	1.00E-03	0.05	AT/GC SNPs	Illumina HumanHap 550	Umich Imputation Server	HRC 1.1	Dosages	Age, sex (all smokers).	All unrelated, pi-hat > 0.125.	R
GERA	95%	95%	1.00E-03	0.05	AT/GC SNPs	Affymetrix Axiom KP	Umich Imputation Server	HRC 1.1	Hard calls (INFO > 0.9)	Age, sex, ever smoking.	Unrelated	plink2
Generation Scotland (GS)	97%	98%	1.00E-06	0.01 for OMNI markers, 0.0001 for Exome Chip markers	NA	HumanOmniExpressExome8v1-2_A HumanOmniExpressExome-8v1_A	Shapeit v2.r873 + duohmm & PBWT (Sanger server)	HRC 1.1	Dosages	Age, sex, ever smoking.	Related individuals included in mixed model	SAIGE
HUNT	99%	99%	1.00E-04	0.01	The different arrays were harmonized by reducing to a set of overlapping variants and excluding variants that showed frequency differences >15% between data sets, or that were	Illumina HumanCoreExome arrays (HumanCoreExome12 v1.0, HumanCoreExome12 v1.1 and	Minimac3	HRC+WGS 2200 HUNT individuals	Dosages	Birth Year, Sex, 4 PCs, batch.	Generalised logistic mixed model with GRM.	SAIGE 29.1

Study	Pre-imputation filtering metrics					Genotyping platform	Imputation		Analysis details			
	Individual call rate	SNP call rate	SNP HWE P-value filter	SNP MAF filter	Other filters		Software	Reference panel	Genotype data	Covariates	Relatedness	Association software
					monomorphic in one and had MAF > 1% in another data set.	UM HUNT Biobank v1.0)						
LOVELACE	99%	99%	1.00E-08	0.008	Y and pseudo-autosomal region of X removed; filters for low heterozygosity, non-European ancestry, high relatedness.	Illumine HumanOmni2.5-4v1-H BeadChip	Michigan imputation server	HRC 1.1	Dosages	Age, sex (all smokers)	All unrelated, pi-hat > 0.125.	R
Mass General Brigham (MGB) Biobank	99%	90%	NA	0	Invalid alleles, duplicate, monomorphic, indel, allele mismatch, low call rate, SNPS not in HRC 1.1, discrepancies between predicted and reported sex.	3 Iterations of Multi-Ethnic Genotyping Array (Illumina)	Minimac4	HRC 1.1	Dosages	Age, sex, smoking, principal components.	Related individuals excluded; kinship threshold =0.0884	R
Rotterdam Study (RS) (I, II and III)	98%	98%	1.00E-06	0.01	Sample exclusion (missing DNA, gender mismatch, excess autosomal heterozygosity, duplicates, ethnic outliers)	Illumina	Michigan imputation server	HRC 1.1	Dosages	Age, sex, smoking and 4 PCs.	No related individuals included.	rvtest*
SPIROMICS	95%	95%	1.00E-06	0.01	Sex, ethnicity, relatedness.	Illumina HumanOmniExpressExome	Michigan Imputation Server	HRC 1.1	Threshold-assigned calls	Age, sex, current smoking status, smoking pack-years, 5 PCs.	No related individuals included.	PLINK1.9

[1] deCODE: The GWASs were performed with 32.5 million markers identified through whole-genome sequencing of 28,075 Icelanders to an average genome-wide coverage of 34X; imputed into 155,250 chip-typed individuals, and their first and second degree relatives.

[2] deCODE: Estimating the effective number of cases as 2 × Na × Nc / (Na+Nc)/GC, where Na and Nc are the number of cases and controls, respectively, and GC is the genomic adjustment factor, estimated using LD-score regression (Bulik-Sullivan, B.K., Nat Gen, 2015), we can calculate the effective sample size

e-Table 3 Proxy SNPs used in Stage 2 cohorts where lead SNP was missing

chr:pos=chromosome and position (genome build 37); other/coded (other and coded alleles). Details of proxies in LD (at r^2>0.3) with the lead SNP, along with rsIDs are given

rsid	chr:pos	other/coded	Studies in which SNP is missing and proxy with r2>0.3 available (rsID, r2)	Studies in which SNP is missing and NO proxy with r^2>0.3 available
rs159960	1:8476428	A/G	LOVELACE (rs301805, r2=0.862)	
rs41301991	1:15287168	C/T	LOVELACE (rs12563750, r2=0.314)	
rs34290285	2:242698640	G/A	LOVELACE (rs7563672, r2=0.553)	ECLIPSE, Rotterdam
rs35570272	3:33047662	G/T	LOVELACE (rs4678523, r2=0.581)	
rs6787279	3:57163751	T/C		
rs62326355	4:120141978	A/G	LOVELACE (rs17331353, r2=0.374)	
rs17720281	4:145543776	C/T	LOVELACE (rs17776795, r2=1.000)	
rs16903574	5:14610309	C/G		
rs80101740	5:98471135	A/C		LOVELACE
rs1837253	5:110401872	T/C		
rs3749833	5:131799626	T/C	LOVELACE (rs2548993, r2=0.929)	
rs7383537	6:30074872	C/T	LOVELACE, GERA (rs2240068, r2=0.784); Rotterdam, HUNT (rs12665401, r2=0.315)	
rs9273410	6:32627250	C/A	LOVELACE, ECLIPSE, Rotterdam (rs1063355, r2=0.560); FHS (rs9272346, r2=0.558)	GERA
rs115643887	6:109280933	G/T	LOVELACE (rs12195848, r2=0.995)	
rs1339248	9:78625640	T/C	LOVELACE (rs1416553, r2=0.996)	
rs1028655	9:101652106	A/G		
rs2076846	10:6063253	A/G		
rs2033149	10:60264741	A/T	LOVELACE (rs1658495, r2=0.898)	
rs55646091	11:76299431	G/A		LOVELACE
rs4936678	11:111625085	C/T	LOVELACE (rs4936682, r2=0.921)	
rs56136109	12:8211784	A/G		LOVELACE
rs7312770	12:56467587	C/T	LOVELACE (rs11171739, r2=0.754)	

rsid	chr:pos	other/coded	Studies in which SNP is missing and proxy with r2>0.3 available (rsID, r2)	Studies in which SNP is missing and NO proxy with r^2>0.3 available
rs17241080	12:94619773	C/G		LOVELACE
rs7995004	13:44478820	C/T	LOVELACE (rs1373904, r2=0.989)	
rs9513600	13:99996015	G/A	LOVELACE (rs2181502, r2=0.982)	
rs2180369	14:93516465	T/C		
rs118140944	14:103650919	A/G		LOVELACE
rs1031460	17:38072247	T/G	LOVELACE (rs12603332, r2=0.967)	
rs1029792	17:38808941	G/T	LOVELACE (rs7209735, r2=1.000)	
rs2584662	17:47470487	A/C	LOVELACE (rs2584663, r2=0.748)	
rs4804461	19:9959556	C/T	LOVELACE (rs10415033, r2=0.413)	

e-Table 4: 83 signals discovered in UK Biobank plus sensitivity analyses comparing ACO cases to asthma (not COPD) and COPD (not asthma) control sets

chr:pos_other_effect=chromosome, position (b37), other allele, effect allele; EAF=frequency of effect allele; Passes prioritisation?=meets P<0.01 threshold in signal prioritisation analyses; OR=odds ratio; LCI/UCI=lower/upper bound of 95% confidence interval; p=p-value. Stage 1 = UK Biobank; Stage 2 = independent follow-up cohorts. Variants taken forward to Stage 2 analyses are set to TRUE in the column 'Passes prioritisation?'

SNP information							UKB (8068 cases, 40360 controls)				UKB (8068 cases, 26815 controls with asthma but not COPD)				UKB (8068 cases, 16762 controls with COPD but not asthma)			
**rsid**	**chr:pos_other_effect**	**EAF**	**Gene**	**Location**	**Passes prioritisation?**	**Conditioned on**	**OR**	**LCI**	**UCI**	**P**	**OR**	**LCI**	**UCI**	**P**	**OR**	**LCI**	**UCI**	**p**
rs159960	1:8476428_A_G	0.559	*RERE*	intronic	TRUE		1.128	1.087	1.170	1.33E-10	1.098	1.056	1.141	2.00E-06	1.111	1.069	1.155	9.44E-08
rs41301991	1:15287168_C_T	0.022	*KAZN*	intronic	TRUE		1.394	1.232	1.578	1.42E-07	1.279	1.128	1.451	1.26E-04	1.279	1.128	1.451	1.31E-04
rs12123821	1:152179152_C_T	0.049	*HRNR*	intergenic	FALSE		1.280	1.177	1.392	8.56E-09	1.101	1.013	1.197	2.41E-02	1.255	1.150	1.369	3.42E-07
rs61816761	1:152285861_G_A	0.023	*FLG*	exonic	FALSE		1.483	1.307	1.681	8.47E-10	1.101	0.977	1.239	1.14E-01	1.320	1.163	1.498	1.67E-05
rs4090390	1:173146921_C_A	0.243	*TNFSF4*	intergenic	FALSE		1.110	1.064	1.158	1.53E-06	1.053	1.007	1.100	2.35E-02	1.065	1.018	1.113	5.78E-03
rs891058	2:8442547_G_A	0.293	*LINC00299*	ncRNA intronic	FALSE		0.888	0.854	0.925	6.93E-09	0.954	0.914	0.995	2.98E-02	0.910	0.872	0.949	1.10E-05
rs7563253	2:20391281_C_G	0.471	*SDC1*	intergenic	FALSE		0.915	0.882	0.949	2.11E-06	0.935	0.900	0.972	6.27E-04	0.970	0.933	1.008	1.22E-01
rs1420104	2:102948470_G_A	0.390	*IL1RL1*	intronic	FALSE		1.135	1.093	1.178	3.00E-11	1.032	0.993	1.073	1.11E-01	1.103	1.061	1.147	7.72E-07
rs6723099	2:229592415_T_A	0.081	*PID1*	intergenic	FALSE		0.834	0.779	0.893	1.84E-07	0.850	0.790	0.914	1.19E-05	0.965	0.895	1.041	3.54E-01
rs6747736	2:239891054_A_C	0.617	*FLJ43879*	intergenic	FALSE		1.099	1.058	1.140	8.31E-07	1.094	1.052	1.138	6.90E-06	1.022	0.982	1.063	2.92E-01
rs34290285	2:242698640_G_A	0.252	*D2HGDH*	intronic	TRUE		0.862	0.827	0.899	4.31E-12	0.928	0.887	0.970	1.06E-03	0.856	0.819	0.894	4.56E-12
rs2442776	3:11640601_G_A	0.863	*VGLL4*	intronic	FALSE		0.870	0.825	0.918	2.70E-07	0.884	0.836	0.934	1.04E-05	0.937	0.887	0.990	1.96E-02
rs35570272	3:33047662_G_T	0.398	*GLB1*	intronic	TRUE		1.107	1.066	1.149	1.06E-07	1.054	1.014	1.096	8.03E-03	1.086	1.044	1.129	3.60E-05
rs6787279	3:57163751_T_C	0.169	*IL17RD*	intronic	TRUE		0.880	0.838	0.924	2.69E-07	0.931	0.885	0.981	7.20E-03	0.921	0.874	0.970	1.88E-03
rs13874	3:66419956_C_T	0.436	*SLC25A26*	exonic	FALSE		1.098	1.058	1.139	7.04E-07	1.068	1.027	1.110	8.57E-04	1.047	1.008	1.088	1.92E-02
rs57699322	3:128065313_C_T	0.115	*EEFSEC*	intronic	FALSE		0.874	0.826	0.925	2.98E-06	0.918	0.864	0.975	5.33E-03	0.951	0.894	1.011	1.05E-01
rs1420472	3:168776326_G_T	0.438	*MECOM*	intergenic	FALSE		1.104	1.065	1.146	1.20E-07	1.065	1.025	1.107	1.27E-03	1.022	0.983	1.062	2.69E-01
rs7640550	3:188080248_T_C	0.503	*LPP*	intronic	FALSE		0.910	0.878	0.944	4.81E-07	0.957	0.920	0.994	2.37E-02	0.928	0.893	0.964	1.38E-04
rs73234989	4:38754888_G_A	0.158	*TLR10*	intergenic	FALSE		0.877	0.833	0.923	4.14E-07	0.932	0.883	0.984	1.07E-02	0.891	0.844	0.940	2.51E-05
rs34712979	4:106819053_G_A	0.259	*NPNT*	intronic	FALSE		1.210	1.161	1.262	2.20E-19	1.183	1.132	1.235	3.53E-14	1.010	0.968	1.054	6.44E-01
rs62326355	4:120141978_A_G	0.017	*USP53*	intronic	TRUE		1.434	1.238	1.660	1.44E-06	1.288	1.110	1.494	8.56E-04	1.353	1.164	1.573	8.53E-05
rs6842889	4:145479880_T_C	0.392	*HHIP-AS1*	intergenic	FALSE		0.842	0.811	0.874	1.58E-19	0.844	0.812	0.878	2.39E-17	0.983	0.944	1.023	4.01E-01
rs17720281	4:145543776_C_T	0.429	*HHIP-AS1*	intergenic	TRUE	rs6842889	1.088	1.050	1.127	3.77E-06	1.075	1.036	1.115	1.09E-04	1.080	1.039	1.124	1.12E-04
rs16903574	5:14610309_C_G	0.077	*FAM105A*	exonic	TRUE		1.231	1.148	1.319	4.47E-09	1.115	1.040	1.197	2.37E-03	1.185	1.103	1.273	3.21E-06
rs11742240	5:35881376_G_T	0.274	*IL7R*	intergenic	FALSE		0.906	0.870	0.944	2.08E-06	0.972	0.931	1.016	2.06E-01	0.946	0.906	0.988	1.17E-02
rs139785776	5:43716302_G_A	0.049	*NNT*	intergenic	FALSE		1.225	1.124	1.334	3.48E-06	1.113	1.021	1.214	1.54E-02	1.085	0.994	1.183	6.68E-02
rs80101740	5:98471135_A_C	0.015	*LOC100289230*	intergenic	TRUE		1.443	1.241	1.678	1.87E-06	1.318	1.130	1.538	4.37E-04	1.296	1.113	1.508	8.23E-04
rs1837253	5:110401872_T_C	0.739	*TSLP*	intergenic	TRUE		1.222	1.172	1.274	4.22E-21	1.080	1.032	1.129	7.96E-04	1.180	1.129	1.233	1.55E-13
rs13182369	5:122425832_G_T	0.375	*PRDM6*	exonic	FALSE		1.094	1.053	1.136	3.04E-06	1.075	1.034	1.119	3.00E-04	1.042	1.002	1.084	3.83E-02
rs3749833	5:131799626_T_C	0.263	*C5orf56*	ncRNA intronic	TRUE		1.158	1.111	1.207	3.10E-12	1.059	1.015	1.105	8.32E-03	1.108	1.062	1.157	2.40E-06
rs2069757	5:131998413_G_A	0.074	*IL13*	intergenic	FALSE	rs3749833	1.180	1.101	1.265	3.25E-06	1.030	0.965	1.098	3.78E-01	1.122	1.045	1.203	1.42E-03
rs249677	5:141539339_C_A	0.632	*NDFIP1*	intergenic	FALSE		1.095	1.055	1.138	2.37E-06	1.026	0.986	1.068	2.04E-01	1.062	1.020	1.105	3.20E-03
rs7733410	5:147856522_G_A	0.442	*HTR4*	intronic	FALSE		0.907	0.874	0.940	1.48E-07	0.933	0.897	0.969	4.05E-04	1.015	0.976	1.055	4.65E-01
rs11134789	5:156944199_C_A	0.341	*ADAM19*	intronic	FALSE		1.106	1.065	1.150	2.36E-07	1.082	1.040	1.126	1.16E-04	1.016	0.976	1.057	4.45E-01
rs12519165	5:170901586_A_T	0.625	*FGF18*	intergenic	FALSE		0.901	0.867	0.936	1.05E-07	0.899	0.863	0.936	2.68E-07	0.956	0.919	0.995	2.92E-02
rs2714335	6:7238069_T_C	0.627	*RREB1*	intronic	FALSE		1.097	1.056	1.139	1.65E-06	1.074	1.033	1.118	3.95E-04	1.026	0.986	1.068	2.12E-01
rs6905736	6:19843767_C_A	0.847	*ID4*	intergenic	FALSE		1.131	1.074	1.192	3.54E-06	1.096	1.037	1.158	1.08E-03	1.028	0.973	1.087	3.26E-01
rs7383537	6:30074872_C_T	0.372	*TRIM31-AS1*	ncRNA intronic	TRUE		0.893	0.859	0.928	6.96E-09	0.920	0.884	0.958	5.07E-05	0.930	0.893	0.969	4.56E-04

SNP information							UKB (8068 cases, 40360 controls)				UKB (8068 cases, 26815 controls with asthma but not COPD)				UKB (8068 cases, 16762 controls with COPD but not asthma)			
rsid	chr:pos_other_effect	EAF	Gene	Location	Passes prioritisation?	Conditioned on	OR	LCI	UCI	P	OR	LCI	UCI	P	OR	LCI	UCI	p
rs11751024	6:32586236_C_A	0.396	*HLA-DQA1*	intergenic	FALSE		0.867	0.835	0.900	6.62E-14	0.977	0.939	1.017	2.52E-01	0.851	0.818	0.885	9.19E-16
rs9273410	6:32627250_C_A	0.445	*HLA-DQB1*	UTR3	TRUE		1.237	1.190	1.286	4.37E-27	1.109	1.065	1.155	5.15E-07	1.136	1.091	1.182	5.62E-10
rs969577	6:90982387_T_C	0.350	*BACH2*	intronic	FALSE		0.898	0.865	0.933	3.74E-08	0.953	0.915	0.992	1.89E-02	0.919	0.882	0.957	3.90E-05
rs115643887	6:109280933_G_T	0.123	*ARMC2*	intronic	TRUE		0.873	0.825	0.923	1.55E-06	0.901	0.850	0.956	5.70E-04	0.910	0.857	0.965	1.80E-03
rs9482850	6:128293506_C_T	0.156	*PTPRK*	intronic	FALSE		0.879	0.836	0.924	4.97E-07	0.934	0.885	0.986	1.28E-02	0.918	0.870	0.969	1.90E-03
rs262126	6:142835364_A_C	0.312	*LOC153910*	intergenic	FALSE		0.881	0.847	0.916	2.29E-10	0.883	0.847	0.920	3.26E-09	1.029	0.986	1.073	1.90E-01
rs12700186	7:20416711_T_C	0.416	*ITGB8*	intronic	FALSE		0.895	0.862	0.928	3.35E-09	0.948	0.912	0.986	7.13E-03	0.954	0.917	0.992	1.76E-02
rs10245867	7:28142186_G_T	0.330	*JAZF1*	intronic	FALSE		1.113	1.071	1.157	6.41E-08	1.046	1.004	1.089	2.97E-02	1.091	1.048	1.136	2.68E-05
rs7837219	8:81283175_G_A	0.653	*ZBTB10*	intergenic	FALSE		0.913	0.879	0.949	3.44E-06	0.970	0.933	1.010	1.38E-01	0.910	0.874	0.947	3.84E-06
rs413382	9:6142948_C_A	0.822	*IL33*	intergenic	FALSE		1.174	1.119	1.231	4.63E-11	1.061	1.008	1.117	2.38E-02	1.157	1.100	1.217	1.61E-08
rs2381416	9:6193455_C_A	0.736	*IL33*	intergenic	FALSE		0.845	0.811	0.881	1.09E-15	0.954	0.915	0.996	3.02E-02	0.879	0.842	0.917	3.40E-09
rs1339248	9:78625640_T_C	0.142	*PCSK5*	intronic	TRUE		1.135	1.078	1.196	1.85E-06	1.123	1.064	1.185	2.73E-05	1.078	1.021	1.137	6.75E-03
rs1028655	9:101652106_A_G	0.620	*GALNT12*	intergenic	TRUE		0.899	0.866	0.934	3.06E-08	0.914	0.879	0.951	7.24E-06	0.948	0.912	0.986	8.05E-03
rs2076846	10:6063253_A_G	0.363	*IL2RA*	intronic	TRUE		1.105	1.064	1.148	2.57E-07	1.065	1.024	1.108	1.84E-03	1.079	1.037	1.123	1.78E-04
rs2197415	10:9062856_T_G	0.575	*LOC101928272*	intergenic	FALSE		1.127	1.086	1.169	1.92E-10	1.015	0.977	1.056	4.44E-01	1.078	1.037	1.121	1.58E-04
rs2033149	10:60264741_A_T	0.437	*BICC1*	intergenic	TRUE		1.093	1.053	1.134	2.44E-06	1.087	1.045	1.129	2.54E-05	1.082	1.041	1.125	7.41E-05
rs79860187	10:78630609_G_A	0.019	*KCNMA1*	UTR3	FALSE		1.371	1.199	1.568	4.08E-06	1.191	1.042	1.362	1.05E-02	1.186	1.037	1.355	1.24E-02
rs3127449	10:79269731_A_C	0.297	*KCNMA1*	intronic	FALSE		1.098	1.056	1.143	3.49E-06	1.090	1.046	1.137	4.17E-05	1.049	1.006	1.093	2.42E-02
rs7936323	11:76293758_G_A	0.480	*C11orf30*	intergenic	FALSE		1.150	1.109	1.193	6.73E-14	1.035	0.996	1.075	7.87E-02	1.135	1.092	1.179	9.37E-11
rs55646091	11:76299431_G_A	0.051	*C11orf30*	intergenic	TRUE		1.390	1.279	1.511	9.23E-15	1.136	1.047	1.233	2.25E-03	1.273	1.170	1.385	2.23E-08
rs12362635	11:86449598_T_C	0.152	*PRSS23*	intergenic	FALSE		1.126	1.071	1.184	4.13E-06	1.095	1.039	1.155	6.77E-04	1.019	0.968	1.074	4.66E-01
rs4936678	11:111625085_C_T	0.255	*PPP2R1B*	intronic	TRUE		0.897	0.860	0.936	4.26E-07	0.927	0.887	0.969	8.39E-04	0.909	0.869	0.950	2.63E-05
rs56136109	12:8211784_A_G	0.028	*C3AR1*	exonic	TRUE		0.772	0.691	0.862	4.07E-06	0.835	0.741	0.942	3.30E-03	0.846	0.749	0.955	6.97E-03
rs142146039	12:19896930_A_G	0.050	*AEBP2*	intergenic	FALSE		1.218	1.120	1.324	4.00E-06	1.216	1.115	1.327	1.12E-05	1.098	1.009	1.196	3.11E-02
rs7312770	12:56467587_C_T	0.495	*ERBB3*	intergenic	TRUE		0.884	0.853	0.918	5.67E-11	0.921	0.886	0.957	2.90E-05	0.908	0.874	0.944	8.78E-07
rs703817	12:57489828_C_T	0.518	*STAT6*	UTR3	FALSE		1.100	1.061	1.141	3.10E-07	1.041	1.002	1.081	4.16E-02	1.109	1.067	1.152	1.20E-07
rs17241080	12:94619773_C_G	0.058	*PLXNC1*	intronic	TRUE		1.254	1.157	1.359	3.25E-08	1.181	1.087	1.282	7.96E-05	1.237	1.138	1.345	5.84E-07
rs7995004	13:44478820_C_T	0.209	*LACC1*	intergenic	TRUE		1.118	1.069	1.169	1.16E-06	1.085	1.035	1.136	6.21E-04	1.103	1.053	1.156	3.94E-05
rs9513600	13:99996015_G_A	0.716	*MIR548AN*	ncRNA intronic	TRUE		1.123	1.079	1.170	1.63E-08	1.062	1.018	1.109	5.49E-03	1.100	1.054	1.148	1.16E-05
rs1950897	14:68760141_C_T	0.712	*RAD51B*	intronic	FALSE		0.898	0.862	0.935	1.57E-07	0.950	0.911	0.991	1.63E-02	0.930	0.892	0.970	7.54E-04
rs2180369	14:93516465_T_C	0.113	*ITPK1*	intronic	TRUE		1.170	1.105	1.239	7.68E-08	1.153	1.087	1.224	2.68E-06	1.099	1.036	1.167	1.74E-03
rs118140944	14:103650919_A_G	0.033	*LINC00605*	intergenic	TRUE		0.785	0.710	0.869	2.91E-06	0.797	0.715	0.888	4.06E-05	0.821	0.735	0.917	4.67E-04
rs12905602	15:61064698_G_A	0.133	*RORA*	intronic	FALSE		0.867	0.822	0.914	1.51E-07	0.929	0.877	0.984	1.22E-02	0.860	0.812	0.910	1.62E-07
rs56062135	15:67455630_C_T	0.237	*SMAD3*	intronic	FALSE		1.169	1.121	1.220	5.67E-13	1.025	0.981	1.070	2.72E-01	1.166	1.116	1.219	9.88E-12
rs4337253	15:71609306_G_C	0.334	*THSD4*	intronic	FALSE		1.127	1.084	1.171	1.27E-09	1.127	1.082	1.173	6.39E-09	0.994	0.955	1.035	7.87E-01
rs283789	15:76777577_G_A	0.675	*SCAPER*	intronic	FALSE		0.911	0.876	0.947	2.69E-06	0.958	0.921	0.998	3.92E-02	0.956	0.918	0.995	2.85E-02
rs77203805	15:84286544_G_A	0.112	*SH3GL3*	intronic	FALSE		0.860	0.811	0.911	3.30E-07	0.903	0.849	0.960	1.14E-03	0.928	0.871	0.988	1.90E-02
rs2241099	16:11225064_C_G	0.249	*CLEC16A*	intronic	FALSE		0.892	0.855	0.931	1.29E-07	0.961	0.919	1.005	8.01E-02	0.909	0.869	0.950	2.57E-05
rs1031460	17:38072247_T_G	0.504	*GSDMB*	intronic	TRUE		1.155	1.113	1.197	9.96E-15	1.065	1.025	1.106	1.25E-03	1.161	1.118	1.206	1.59E-14
rs1029792	17:38808941_G_T	0.694	*KRT222*	intergenic	TRUE		1.098	1.055	1.143	3.67E-06	1.074	1.030	1.119	8.50E-04	1.102	1.057	1.149	4.64E-06
rs2584662	17:47470487_A_C	0.420	*PHB*	intergenic	TRUE		0.901	0.868	0.935	3.20E-08	0.920	0.885	0.956	2.45E-05	0.934	0.899	0.971	6.25E-04
rs631126	18:8800723_T_C	0.751	*MTCL1*	intronic	FALSE		0.897	0.860	0.935	3.61E-07	0.941	0.901	0.983	6.42E-03	0.976	0.934	1.020	2.77E-01
rs4804461	19:9959556_C_T	0.249	*PIN1*	intronic	TRUE		0.902	0.865	0.941	1.73E-06	0.929	0.889	0.971	1.13E-03	0.925	0.885	0.967	6.27E-04
rs11672303	19:33726375_T_C	0.116	*SLC7A10*	intergenic	FALSE		0.871	0.823	0.922	1.97E-06	0.934	0.879	0.993	2.86E-02	0.869	0.819	0.923	4.14E-06
rs5992904	22:18415158_A_G	0.222	*MICAL3*	intronic	FALSE		0.887	0.849	0.927	9.12E-08	0.915	0.874	0.959	1.99E-04	0.951	0.907	0.997	3.57E-02

e-Table 5: Comparison of stage 1 results for 31 signals with stage 2 results, and joint analyses

chr:pos_other_effect=chromosome, position (b37), other allele, effect allele; EAF=frequency of effect allele; Novelty (LD)=is SNP in LD with another SNP previously associated with asthma, COPD or lung function; OR=odds ratio, LCI/UCI=lower/upper bound of 95% confidence interval; P=P-value. I^2 (%)=I-squared metric (%) for heterogeneity in meta-analysis. Stage 1 = UK Biobank; Stage 2 = independent follow-up cohorts. Previous asthma (or LF/COPD) SNP (PMID)=name of SNP and PMID where SNP in LD with sentinel at r^2>0.1 has previously been associated with asthma, or lung function/COPD.
For effect allele frequency, gene and location see e-Table 4. Note that the SNP marked with a * was conditioned on rs6842889.

SNP information					Stage 1 (UKB) results (8068 cases, 40360 controls)				Meta-analysis of Stage 2 cohorts (4301 cases, 48609 controls)					Joint analysis of Stage 1 and Stage 2					COPDGene African-American (AA) individuals (297 cases, 1335 controls)				
rsid	chr:pos_other_effect	Novelty (LD)	Previous asthma SNP (PMID)	Previous LF/COPD SNP (PMID)	OR	LCI	UCI	P	OR	LCI	UCI	P	I^2 (%)	OR	LCI	UCI	P	I^2 (%)	EAF	OR	LCI	UCI	P
rs159960	1:8476428_A_G	FALSE	rs301805 (29785011), rs301806 (29083406)	NA	1.128	1.087	1.170	1.33E-10	1.026	0.974	1.080	3.33E-01	0	1.093	1.060	1.126	6.65E-09	25	0.173	0.904	0.685	1.194	0.479
rs41301991	1:15287168_C_T	TRUE	NA	NA	1.394	1.232	1.578	1.42E-07	1.024	0.859	1.222	7.90E-01	0	1.259	1.138	1.394	8.14E-06	21	0.009	0.332	0.058	1.894	0.215
rs34290285	2:242698640_G_A	FALSE	rs34290285 (29083406, 27182965, 31036433, 29785011)	NA	0.862	0.827	0.899	4.31E-12	0.952	0.895	1.013	1.20E-01	39	0.890	0.859	0.921	4.02E-11	53	0.325	0.732	0.545	0.982	0.038
rs35570272	3:33047662_G_T	FALSE	rs35570272 (31036433), rs6776757 (29083406)	NA	1.107	1.066	1.149	1.06E-07	1.077	1.023	1.135	4.67E-03	19	1.097	1.064	1.130	2.44E-09	16	0.245	1.209	0.963	1.517	0.102
rs6787279	3:57163751_T_C	FALSE	NA	rs6445932 (30061609, 30804561)	0.880	0.838	0.924	2.69E-07	0.909	0.848	0.974	6.51E-03	15	0.889	0.855	0.926	7.87E-09	11	0.433	0.990	0.814	1.205	0.921
rs62326355	4:120141978_A_G	TRUE	NA	NA	1.434	1.238	1.660	1.44E-06	0.957	0.797	1.149	6.40E-01	0	1.224	1.092	1.372	5.26E-04	43	0.002	0.315	0.005	18.345	0.578
rs17720281*	4:145543776_C_T	FALSE	NA	rs13116999 (30804560)	1.088	1.050	1.127	3.77E-06	1.010	0.961	1.063	6.88E-01	28	1.062	1.031	1.093	6.03E-05	42	0.248	1.076	0.861	1.346	0.518
rs16903574	5:14610309_C_G	FALSE	rs16903574 (29083406, 31036433)	NA	1.231	1.148	1.319	4.47E-09	1.134	1.031	1.248	9.96E-03	0	1.197	1.131	1.266	3.80E-10	0	0.014	2.201	1.102	4.396	0.025
rs80101740	5:98471135_A_C	TRUE	NA	NA	1.443	1.241	1.678	1.87E-06	1.371	1.097	1.714	5.49E-03	0	1.420	1.253	1.609	3.72E-08	0	0.003	0.830	0.135	5.094	0.840
rs1837253	5:110401872_T_C	FALSE	rs1837253 (24388013, 29083406, 21804548, 27182965, 31036433, 21804549, 29785011), rs10455025 (29273806)	NA	1.222	1.172	1.274	4.22E-21	1.060	1.001	1.122	4.44E-02	0	1.162	1.124	1.202	1.53E-18	53	0.711	1.106	0.898	1.361	0.344
rs3749833	5:131799626_T_C	FALSE	rs17622378 (31036433), rs3749833 (29083406), rs2548992 (29785011)	rs3843503 (28166213)	1.158	1.111	1.207	3.10E-12	1.060	1.002	1.121	4.21E-02	0	1.123	1.086	1.160	9.37E-12	14	0.106	1.040	0.755	1.432	0.809
rs7383537	6:30074872_C_T	FALSE	rs1056241 (29083406), rs1117490 (31036433)	NA	0.893	0.859	0.928	6.96E-09	0.979	0.928	1.033	4.39E-01	0	0.921	0.893	0.950	2.43E-07	33	0.547	0.947	0.781	1.149	0.584
rs9273410	6:32627250_C_A	FALSE	rs1117490 (31036433), rs9272346 (27182965, 29273806), rs17843604 (30578877), rs28407950 (31036433), rs34004019 (29083406), rs1063355 (24388013, 29785011)	rs9274247 (26423011)	1.237	1.190	1.286	4.37E-27	1.111	1.046	1.181	6.42E-04	0	1.199	1.161	1.239	9.19E-28	29	0.415	0.974	0.761	1.246	0.833

rs115643887	6:109280933_G_T	TRUE	NA	NA	0.873	0.825	0.923	1.55E-06	0.948	0.877	1.025	1.84E-01	1	0.897	0.858	0.939	2.82E-06	14	0.118	1.181	0.888	1.570	0.254
rs1339248	9:78625640_T_C	TRUE	NA	NA	1.135	1.078	1.196	1.85E-06	1.023	0.948	1.103	5.62E-01	22	1.098	1.052	1.146	2.09E-05	37	0.147	0.769	0.572	1.032	0.080
rs1028655	9:101652106_A_G	FALSE	NA	rs57649467 (30804560)	0.899	0.866	0.934	3.06E-08	0.998	0.947	1.052	9.42E-01	20	0.931	0.903	0.960	5.21E-06	49	0.786	0.772	0.616	0.967	0.024
rs2076846	10:6063253_A_G	FALSE	rs4747846 (29083406), rs61839660 (29083406, 29785011)	NA	1.105	1.064	1.148	2.57E-07	1.027	0.974	1.082	3.24E-01	51	1.078	1.045	1.111	2.01E-06	56	0.125	1.029	0.777	1.361	0.843
rs2033149	10:60264741_A_T	TRUE	NA	NA	1.093	1.053	1.134	2.44E-06	1.001	0.952	1.054	9.56E-01	0	1.060	1.029	1.092	1.23E-04	30	0.747	1.062	0.838	1.346	0.618
rs55646091	11:76299431_G_A	FALSE	rs61894547 (31036433), rs55646091 (29083406)	NA	1.390	1.279	1.511	9.23E-15	0.986	0.871	1.115	8.19E-01	0	1.248	1.165	1.338	3.12E-10	60	0.007	1.456	0.438	4.847	0.540
rs4936678	11:111625085_C_T	FALSE	rs659529 (29785011), rs7130753 (29083406)	NA	0.897	0.860	0.936	4.26E-07	0.944	0.890	1.000	5.13E-02	30	0.913	0.882	0.944	1.57E-07	32	0.070	1.003	0.666	1.513	0.987
rs56136109	12:8211784_A_G	TRUE	NA	NA	0.772	0.691	0.862	4.07E-06	1.003	0.841	1.195	9.75E-01	17	0.831	0.757	0.912	1.02E-04	39	0.006	0.185	0.023	1.458	0.109
rs7312770	12:56467587_C_T	FALSE	rs705699 (31036433), rs10876864 (29083406, 29785011), rs1701704 (21804548)	rs1689510 (30061609)	0.884	0.853	0.918	5.67E-11	0.960	0.913	1.010	1.18E-01	17	0.910	0.883	0.937	4.91E-10	40	0.482	1.096	0.904	1.328	0.351
rs17241080	12:94619773_C_G	TRUE	NA	NA	1.254	1.157	1.359	3.25E-08	1.073	0.961	1.199	2.08E-01	0	1.188	1.114	1.268	1.90E-07	26	0.008	1.706	0.453	6.419	0.429
rs7995004	13:44478820_C_T	FALSE	rs9316059 (31361310)	NA	1.118	1.069	1.169	1.16E-06	1.013	0.950	1.079	6.95E-01	42	1.082	1.043	1.122	2.67E-05	52	0.306	1.053	0.854	1.298	0.631
rs9513600	13:99996015_G_A	FALSE	rs1887704 (31036433)	NA	1.123	1.079	1.170	1.63E-08	1.042	0.985	1.102	1.56E-01	0	1.095	1.060	1.132	6.05E-08	18	0.370	1.110	0.912	1.352	0.297
rs2180369	14:93516465_T_C	TRUE	NA	NA	1.170	1.105	1.239	7.68E-08	1.064	0.985	1.149	1.17E-01	35	1.131	1.080	1.184	1.50E-07	42	0.174	1.002	0.776	1.294	0.990
rs118140944	14:103650919_A_G	TRUE	NA	NA	0.785	0.710	0.869	2.91E-06	0.930	0.805	1.073	3.19E-01	0	0.830	0.764	0.902	1.10E-05	19	0.006	0.445	0.059	3.329	0.430
rs1031460	17:38072247_T_G	FALSE	rs2952156 (29273806), rs11655198 (27182965), rs4795399 (31036433), rs2305480 (24241537, 20860503), rs11078927 (21804549), rs869402 (29785011), rs921650 (29083406), rs7216389 (17611496), rs4794820 (22561531), rs3894194 (20860503), rs7212938 (24388013)	rs8069451 (30061609), rs62065216 (30804561)	1.155	1.113	1.197	9.96E-15	1.048	0.996	1.102	6.88E-02	47	1.117	1.085	1.151	2.06E-13	60	0.814	1.020	0.796	1.305	0.878
rs1029792	17:38808941_G_T	FALSE	rs757411 (29083406), rs9911533 (29785011)	NA	1.098	1.055	1.143	3.67E-06	0.966	0.915	1.020	2.09E-01	40	1.050	1.017	1.084	2.84E-03	63	0.828	1.090	0.839	1.417	0.518
rs2584662	17:47470487_A_C	FALSE	rs9889262 (29083406), rs28406364 (31036433), rs17637472 (29273806)	NA	0.901	0.868	0.935	3.20E-08	0.951	0.904	1.002	5.89E-02	35	0.918	0.890	0.946	2.21E-08	40	0.604	0.949	0.772	1.167	0.622
rs4804461	19:9959556_C_T	TRUE	NA	NA	0.902	0.865	0.941	1.73E-06	1.020	0.959	1.085	5.30E-01	17	0.938	0.906	0.971	3.28E-04	49	0.149	0.816	0.606	1.097	0.179

e-Table 6: Comparison of Stage 1 results for 31 signals with Stage 2 cohorts (sensitivity analysis), and corresponding joint analyses

chr:pos_other_effect=chromosome, position (b37), other allele, effect allele; Novelty (LD)=is SNP in LD with another SNP previously associated with asthma, COPD or lung function; OR=odds ratio, LCI/UCI=lower/upper bound of 95% confidence interval; P=P-value. I^2 (%)=I-squared metric (%) for heterogeneity in meta-analysis. Stage 1 = UK Biobank; Stage 2 = independent follow-up cohorts. Previous asthma (or LF/COPD) SNP (PMID)=name of SNP and PMID where SNP in LD with sentinel at r^2>0.1 has previously been associated with asthma, or lung function/COPD.

For effect allele frequency, gene and location see e-Table 4. Note that the SNP marked with a * was conditioned on rs6842889.

SNP information					Stage 1 (UKB) results (8068 cases, 40360 controls)				Meta-analysis of Stage 2 cohorts (sensitivity analysis) (9638 cases and 128273 controls)					Sensitivity analysis (Joint analysis of Stage 1 and Stage 2)				
rsid	chr:pos_other_effect	Novelty (LD)	Previous asthma SNP (PMID)	Previous LF/COPD SNP (PMID)	OR	LCI	UCI	P	OR	LCI	UCI	P	I^2 (%)	OR	LCI	UCI	P	I^2 (%)
rs159960	1:8476428_A_G	FALSE	rs301805 (29785011), rs301806 (29083406)	NA	1.128	1.087	1.170	1.33E-10	1.018	0.983	1.054	3.25E-01	0	1.069	1.042	1.097	2.51E-07	35
rs41301991	1:15287168_C_T	TRUE	NA	NA	1.394	1.232	1.578	1.42E-07	1.004	0.889	1.133	9.55E-01	0	1.179	1.081	1.286	1.95E-04	43
rs34290285	2:242698640_G_A	FALSE	rs34290285 (29083406, 27182965, 31036433, 29785011)	NA	0.862	0.827	0.899	4.31E-12	0.950	0.910	0.991	1.76E-02	19	0.904	0.878	0.932	4.12E-11	48
rs35570272	3:33047662_G_T	FALSE	rs35570272 (31036433), rs6776757 (29083406)	NA	1.107	1.066	1.149	1.06E-07	1.025	0.990	1.062	1.60E-01	46	1.063	1.036	1.090	3.11E-06	57
rs6787279	3:57163751_T_C	FALSE	NA	rs6445932 (30061609, 30804561)	0.880	0.838	0.924	2.69E-07	0.966	0.922	1.012	1.41E-01	42	0.924	0.894	0.956	4.01E-06	52
rs62326355	4:120141978_A_G	TRUE	NA	NA	1.434	1.238	1.660	1.44E-06	0.974	0.858	1.106	6.86E-01	0	1.149	1.044	1.265	4.47E-03	44
rs17720281*	4:145543776_C_T	FALSE	NA	rs13116999 (30804560)	1.088	1.050	1.127	3.77E-06	0.983	0.950	1.018	3.38E-01	18	1.033	1.007	1.059	1.11E-02	54
rs16903574	5:14610309_C_G	FALSE	rs16903574 (29083406, 31036433)	NA	1.231	1.148	1.319	4.47E-09	1.091	1.021	1.165	9.93E-03	0	1.155	1.101	1.212	3.40E-09	25
rs80101740	5:98471135_A_C	TRUE	NA	NA	1.443	1.241	1.678	1.87E-06	1.189	1.017	1.390	3.04E-02	12	1.314	1.179	1.465	8.05E-07	22
rs1837253	5:110401872_T_C	FALSE	rs1837253 (24388013, 29083406, 21804548, 27182965, 31036433, 21804549, 29785011), rs10455025 (29273806)	NA	1.222	1.172	1.274	4.22E-21	1.035	0.996	1.076	8.31E-02	0	1.118	1.087	1.150	1.34E-14	64
rs3749833	5:131799626_T_C	FALSE	rs17622378 (31036433), rs3749833 (29083406), rs2548992 (29785011)	rs3843503 (28166213)	1.158	1.111	1.207	3.10E-12	1.036	0.997	1.077	7.10E-02	0	1.092	1.061	1.123	1.14E-09	38
rs7383537	6:30074872_C_T	FALSE	rs1056241 (29083406), rs1117490 (31036433)	NA	0.893	0.859	0.928	6.96E-09	0.972	0.938	1.007	1.20E-01	0	0.934	0.910	0.959	3.66E-07	25
rs9273410	6:32627250_C_A	FALSE	rs1117490 (31036433), rs9272346 (27182965, 29273806), rs17843604 (30578877), rs28407950 (31036433), rs34004019 (29083406), rs1063355 (24388013, 29785011)	rs9274247 (26423011)	1.237	1.190	1.286	4.37E-27	1.103	1.055	1.153	1.55E-05	0	1.178	1.144	1.213	5.26E-28	32
rs115643887	6:109280933_G_T	TRUE	NA	NA	0.873	0.825	0.923	1.55E-06	0.985	0.934	1.038	5.71E-01	0	0.930	0.895	0.966	2.01E-04	24
rs1339248	9:78625640_T_C	TRUE	NA	NA	1.135	1.078	1.196	1.85E-06	1.024	0.972	1.079	3.68E-01	26	1.078	1.039	1.118	6.35E-05	43

rs1028655	9:101652106_A_G	FALSE	NA	rs57649467 (30804560)	0.899	0.866	0.934	3.06E-08	0.994	0.959	1.030	7.45E-01	3	0.948	0.924	0.973	4.92E-05	48
rs2076846	10:6063253_A_G	FALSE	rs4747846 (29083406), rs61839660 (29083406, 29785011)	NA	1.105	1.064	1.148	2.57E-07	1.003	0.968	1.040	8.67E-01	52	1.050	1.023	1.078	2.56E-04	65
rs2033149	10:60264741_A_T	TRUE	NA	NA	1.093	1.053	1.134	2.44E-06	1.010	0.976	1.046	5.71E-01	0	1.048	1.022	1.075	2.82E-04	19
rs55646091	11:76299431_G_A	FALSE	rs61894547 (31036433), rs55646091 (29083406)	NA	1.390	1.279	1.511	9.23E-15	1.063	0.980	1.153	1.39E-01	0	1.211	1.143	1.284	1.01E-10	51
rs4936678	11:111625085_C_T	FALSE	rs659529 (29785011), rs7130753 (29083406)	NA	0.897	0.860	0.936	4.26E-07	0.967	0.929	1.007	1.02E-01	16	0.933	0.907	0.961	3.00E-06	35
rs56136109	12:8211784_A_G	TRUE	NA	NA	0.772	0.691	0.862	4.07E-06	0.984	0.882	1.098	7.74E-01	26	0.872	0.806	0.942	5.42E-04	48
rs7312770	12:56467587_C_T	FALSE	rs705699 (31036433), rs10876864 (29083406, 29785011), rs1701704 (21804548)	rs1689510 (30061609)	0.884	0.853	0.918	5.67E-11	0.983	0.950	1.018	3.47E-01	0	0.936	0.912	0.959	2.19E-07	47
rs17241080	12:94619773_C_G	TRUE	NA	NA	1.254	1.157	1.359	3.25E-08	1.054	0.965	1.150	2.41E-01	0	1.158	1.092	1.229	1.14E-06	24
rs7995004	13:44478820_C_T	FALSE	rs9316059 (31361310)	NA	1.118	1.069	1.169	1.16E-06	1.000	0.958	1.043	9.95E-01	0	1.054	1.022	1.087	8.37E-04	39
rs9513600	13:99996015_G_A	FALSE	rs1887704 (31036433)	NA	1.123	1.079	1.170	1.63E-08	1.033	0.995	1.073	9.07E-02	0	1.075	1.045	1.105	3.35E-07	0
rs2180369	14:93516465_T_C	TRUE	NA	NA	1.170	1.105	1.239	7.68E-08	1.019	0.966	1.076	4.87E-01	0	1.088	1.046	1.132	2.63E-05	41
rs118140944	14:103650919_A_G	TRUE	NA	NA	0.785	0.710	0.869	2.91E-06	0.971	0.875	1.077	5.76E-01	0	0.871	0.810	0.936	1.86E-04	30
rs1031460	17:38072247_T_G	FALSE	rs2952156 (29273806), rs11655198 (27182965), rs4795399 (31036433), rs2305480 (24241537, 20860503), rs11078927 (21804549), rs869402 (29785011), rs921650 (29083406), rs7216389 (17611496), rs4794820 (22561531), rs3894194 (20860503), rs7212938 (24388013)	rs8069451 (30061609), rs62065216 (30804561)	1.155	1.113	1.197	9.96E-15	1.048	1.013	1.085	7.12E-03	33	1.097	1.070	1.125	3.46E-13	57
rs1029792	17:38808941_G_T	FALSE	rs757411 (29083406), rs9911533 (29785011)	NA	1.098	1.055	1.143	3.67E-06	0.982	0.947	1.019	3.31E-01	3	1.034	1.006	1.062	1.53E-02	51
rs2584662	17:47470487_A_C	FALSE	rs9889262 (29083406), rs28406364 (31036433), rs17637472 (29273806)	NA	0.901	0.868	0.935	3.20E-08	0.965	0.932	1.000	4.69E-02	36	0.934	0.911	0.958	1.60E-07	48
rs4804461	19:9959556_C_T	TRUE	NA	NA	0.902	0.865	0.941	1.73E-06	1.004	0.963	1.046	8.62E-01	11	0.953	0.925	0.981	1.31E-03	47

e-Table 7: Results of analyses stratified by age of asthma onset and by smoking status

chr:pos (other/effect)=chromosome, position (b37), other allele, effect allele; EAF=frequency of effect allele; OR=odds ratio, LCI/UCI=lower/upper bound of 95% confidence interval; p=p-value.

SNP information				Stage 1 (UKB)				ACO (cases divided by age of asthma onset)								Smoking-stratified ACO GWAS							
				(8068 cases, 40360 controls)				ACO (adult-onset asthma): 3755 cases vs 40360 controls				ACO (child-onset asthma): 2903 cases vs 40360 controls				ACO vs controls (all ever-smokers): 4367 cases vs 17216 controls				ACO vs controls (all never-smokers): 3701 cases, 23044 controls			
**rsid**	**chr:pos (other/effect)**	**EAF**	**gene**	**OR**	**LCI**	**UCI**	**P**	**OR**	**LCI**	**UCI**	**P**	**OR**	**LCI**	**UCI**	**P**	**OR**	**LCI**	**UCI**	**P**	**OR**	**LCI**	**UCI**	**P**
rs159960	1:8476428_A_G	0.559	*RERE*	1.128	1.087	1.170	1.33E-10	1.144	1.087	1.203	1.80E-07	1.124	1.063	1.189	4.05E-05	1.110	1.056	1.166	3.83E-05	1.152	1.092	1.216	2.27E-07
rs41301991	1:15287168_C_T	0.022	*KAZN*	1.394	1.232	1.578	1.42E-07	1.401	1.178	1.665	1.33E-04	1.655	1.364	2.008	3.25E-07	1.404	1.188	1.660	6.90E-05	1.369	1.143	1.640	6.37E-04
rs34290285	2:242698640_G_A	0.252	*D2HGDH*	0.862	0.827	0.899	4.31E-12	0.912	0.862	0.966	1.67E-03	0.843	0.791	0.898	1.30E-07	0.880	0.831	0.931	9.02E-06	0.841	0.791	0.894	3.14E-08
rs35570272	3:33047662_G_T	0.398	*GLB1*	1.107	1.066	1.149	1.06E-07	1.089	1.035	1.146	1.10E-03	1.108	1.047	1.172	4.12E-04	1.068	1.015	1.123	1.13E-02	1.160	1.099	1.225	8.60E-08
rs6787279	3:57163751_T_C	0.169	*IL17RD*	0.880	0.838	0.924	2.69E-07	0.888	0.831	0.949	4.75E-04	0.842	0.783	0.907	5.03E-06	0.853	0.799	0.911	2.31E-06	0.917	0.854	0.985	1.69E-02
rs62326355	4:120141978_A_G	0.017	*USP53*	1.434	1.238	1.660	1.44E-06	1.516	1.236	1.858	6.27E-05	1.267	1.007	1.594	4.37E-02	1.627	1.335	1.982	1.36E-06	1.265	1.019	1.570	3.29E-02
rs17720281*	4:145543776_C_T	0.429	*HHIP-AS1*	1.088	1.050	1.127	3.77E-06	1.062	1.010	1.116	1.87E-02	1.016	0.962	1.074	5.62E-01	1.053	1.003	1.107	3.94E-02	1.033	0.979	1.089	2.38E-01
rs16903574	5:14610309_C_G	0.077	*FAM105A*	1.231	1.148	1.319	4.47E-09	1.188	1.079	1.308	4.45E-04	1.310	1.179	1.456	5.61E-07	1.201	1.094	1.319	1.23E-04	1.268	1.146	1.404	4.55E-06
rs80101740	5:98471135_A_C	0.015	*LOC100289230*	1.443	1.241	1.678	1.87E-06	1.474	1.196	1.815	2.66E-04	1.382	1.092	1.749	7.12E-03	1.586	1.299	1.935	5.64E-06	1.264	1.009	1.585	4.17E-02
rs1837253	5:110401872_T_C	0.739	*TSLP*	1.222	1.172	1.274	4.22E-21	1.169	1.105	1.238	6.68E-08	1.281	1.203	1.364	1.12E-14	1.194	1.129	1.263	6.38E-10	1.259	1.185	1.338	1.02E-13
rs3749833	5:131799626_T_C	0.263	*C5orf56*	1.158	1.111	1.207	3.10E-12	1.124	1.062	1.190	5.44E-05	1.221	1.147	1.300	4.62E-10	1.156	1.093	1.222	3.55E-07	1.166	1.098	1.239	6.60E-07
rs7383537	6:30074872_C_T	0.372	*TRIM31-AS1*	0.893	0.859	0.928	6.96E-09	0.891	0.845	0.939	1.47E-05	0.927	0.875	0.983	1.07E-02	0.890	0.845	0.937	8.84E-06	0.899	0.850	0.951	2.23E-04
rs9273410	6:32627250_C_A	0.445	*HLA-DQB1*	1.237	1.190	1.286	4.37E-27	1.231	1.168	1.299	1.72E-14	1.201	1.132	1.273	9.37E-10	1.221	1.159	1.287	8.61E-14	1.256	1.187	1.328	2.14E-15
rs115643887	6:109280933_G_T	0.123	*ARMC2*	0.873	0.825	0.923	1.55E-06	0.886	0.821	0.956	1.74E-03	0.851	0.782	0.925	1.66E-04	0.851	0.789	0.917	2.53E-05	0.899	0.829	0.975	1.05E-02
rs1339248	9:78625640_T_C	0.142	*PCSK5*	1.135	1.078	1.196	1.85E-06	1.147	1.068	1.232	1.76E-04	1.144	1.056	1.239	9.41E-04	1.111	1.036	1.193	3.24E-03	1.156	1.071	1.247	2.06E-04
rs1028655	9:101652106_A_G	0.620	*GALNT12*	0.899	0.866	0.934	3.06E-08	0.885	0.841	0.932	3.12E-06	0.904	0.854	0.957	4.80E-04	0.912	0.867	0.960	3.81E-04	0.886	0.838	0.935	1.33E-05
rs2076846	10:6063253_A_G	0.363	*IL2RA*	1.105	1.064	1.148	2.57E-07	1.081	1.026	1.139	3.33E-03	1.155	1.090	1.224	1.03E-06	1.083	1.029	1.141	2.26E-03	1.130	1.069	1.194	1.56E-05
rs2033149	10:60264741_A_T	0.437	*BICC1*	1.093	1.053	1.134	2.44E-06	1.107	1.052	1.164	9.16E-05	1.085	1.026	1.148	4.21E-03	1.118	1.064	1.175	1.19E-05	1.064	1.008	1.123	2.42E-02

rs55646091	11:76299431_G_A	0.051	*C11orf30*	1.390	1.279	1.511	9.23E-15	1.188	1.058	1.334	3.66E-03	1.711	1.507	1.943	1.02E-16	1.348	1.204	1.509	2.12E-07	1.446	1.281	1.633	2.72E-09
rs4936678	11:111625085_C_T	0.255	*PPP2R1B*	0.897	0.860	0.936	4.26E-07	0.903	0.852	0.956	4.61E-04	0.916	0.859	0.976	6.43E-03	0.893	0.843	0.945	9.69E-05	0.899	0.846	0.955	6.20E-04
rs56136109	12:8211784_A_G	0.028	*C3AR1*	0.772	0.691	0.862	4.07E-06	0.788	0.678	0.915	1.84E-03	0.787	0.669	0.927	4.15E-03	0.788	0.679	0.914	1.68E-03	0.759	0.646	0.892	8.42E-04
rs7312770	12:56467587_C_T	0.495	*ERBB3*	0.884	0.853	0.918	5.67E-11	0.911	0.867	0.958	2.82E-04	0.860	0.813	0.909	1.04E-07	0.904	0.860	0.950	6.52E-05	0.860	0.815	0.907	3.42E-08
rs17241080	12:94619773_C_G	0.058	*PLXNC1*	1.254	1.157	1.359	3.25E-08	1.228	1.099	1.373	2.98E-04	1.206	1.067	1.364	2.82E-03	1.258	1.126	1.405	4.89E-05	1.238	1.104	1.389	2.67E-04
rs7995004	13:44478820_C_T	0.209	*LACC1*	1.118	1.069	1.169	1.16E-06	1.106	1.040	1.176	1.32E-03	1.127	1.053	1.207	5.92E-04	1.139	1.073	1.210	2.33E-05	1.096	1.026	1.170	6.42E-03
rs9513600	13:99996015_G_A	0.716	*MIR548AN*	1.123	1.079	1.170	1.63E-08	1.082	1.024	1.144	4.81E-03	1.163	1.094	1.237	1.36E-06	1.070	1.013	1.129	1.45E-02	1.188	1.120	1.261	1.37E-08
rs2180369	14:93516465_T_C	0.113	*ITPK1*	1.170	1.105	1.239	7.68E-08	1.173	1.084	1.269	6.80E-05	1.193	1.093	1.303	8.17E-05	1.183	1.095	1.279	1.96E-05	1.155	1.062	1.256	7.35E-04
rs118140944	14:103650919_A_G	0.033	*LINC00605*	0.785	0.710	0.869	2.91E-06	0.820	0.716	0.940	4.28E-03	0.765	0.658	0.889	4.84E-04	0.758	0.660	0.870	8.17E-05	0.814	0.703	0.943	6.05E-03
rs1031460	17:38072247_T_G	0.504	*GSDMB*	1.155	1.113	1.197	9.96E-15	1.085	1.032	1.140	1.40E-03	1.282	1.213	1.354	1.11E-18	1.121	1.068	1.178	4.89E-06	1.194	1.132	1.259	7.71E-11
rs1029792	17:38808941_G_T	0.694	*KRT222*	1.098	1.055	1.143	3.67E-06	1.024	0.970	1.081	3.89E-01	1.145	1.078	1.216	1.09E-05	1.084	1.027	1.143	3.47E-03	1.119	1.056	1.185	1.40E-04
rs2584662	17:47470487_A_C	0.420	*PHB*	0.901	0.868	0.935	3.20E-08	0.900	0.855	0.946	4.30E-05	0.881	0.833	0.932	1.01E-05	0.919	0.874	0.966	9.25E-04	0.881	0.835	0.930	4.36E-06
rs4804461	19:9959556_C_T	0.249	*PIN1*	0.902	0.865	0.941	1.73E-06	0.904	0.853	0.958	6.55E-04	0.886	0.831	0.945	2.08E-04	0.921	0.870	0.975	4.89E-03	0.881	0.828	0.937	5.76E-05

e-Table 8: 99% credible sets for eight signals followed up in downstream analyses, with gene annotations

Bold SNPs had exonic or UTR annotation and were taken forward for variant effect prediction analysis; postprob=posterior probability

sentinel	SNP	BP	A1	A2	A1 freq	Beta	SE	P	postprob	location	gene	distance	NSNPs in credible set
rs35570272	rs7631407	3:33036218	C	G	0.293	0.091	0.021	9.27E-06	0.008	intergenic	*CCR4;GLB1*	dist=39815;dist=1882	
rs35570272	rs9828592	3:33044339	C	T	0.531	0.086	0.018	3.43E-06	0.018	intronic	*GLB1*		
rs35570272	rs7646283	3:33046480	T	C	0.368	0.097	0.019	4.40E-07	0.133	intronic	*GLB1*		
rs35570272	rs34064757	3:33047283	A	G	0.368	0.097	0.019	4.61E-07	0.127	intronic	*GLB1*		
rs35570272	rs35570272	3:33047662	T	G	0.398	0.101	0.019	8.20E-08	0.655	intronic	*GLB1*		
rs35570272	rs73057087	3:33068268	G	A	0.381	0.081	0.019	1.98E-05	0.004	intronic	*GLB1*		
rs35570272	rs4547648	3:33080256	G	A	0.380	0.081	0.019	1.94E-05	0.004	intronic	*GLB1*		
rs35570272	rs4277638	3:33080356	A	G	0.380	0.082	0.019	1.77E-05	0.004	intronic	*GLB1*		
rs35570272	rs4274702	3:33080722	T	C	0.380	0.082	0.019	1.72E-05	0.004	intronic	*GLB1*		
rs35570272	rs6800001	3:33081102	A	G	0.380	0.082	0.019	1.71E-05	0.004	intronic	*GLB1*		
rs35570272	rs28752078	3:33084861	T	C	0.411	0.090	0.019	1.81E-06	0.034	intronic	*GLB1*		11
rs6787279	rs74932439	3:57115567	T	C	0.162	-0.119	0.025	1.90E-06	0.015	intergenic	*ARHGEF3;IL17RD*	dist=2231;dist=8443	
rs6787279	rs74999629	3:57117542	A	G	0.161	-0.118	0.025	2.33E-06	0.013	intergenic	*ARHGEF3;IL17RD*	dist=4206;dist=6468	
rs6787279	rs59527464	3:57122780	T	C	0.198	-0.118	0.023	3.67E-07	0.070	intergenic	*ARHGEF3;IL17RD*	dist=9444;dist=1230	
**rs6787279**	**rs2035656**	**3:57125101**	**C**	**A**	**0.161**	**-0.119**	**0.025**	**1.82E-06**	**0.016**	**UTR3**	**IL17RD**		
**rs6787279**	**rs2035655**	**3:57125202**	**G**	**T**	**0.198**	**-0.118**	**0.023**	**3.37E-07**	**0.075**	**UTR3**	**IL17RD**		
**rs6787279**	**rs17289035**	**3:57125424**	**A**	**G**	**0.198**	**-0.118**	**0.023**	**3.37E-07**	**0.075**	**UTR3**	**IL17RD**		
**rs6787279**	**rs12486855**	**3:57125864**	**G**	**A**	**0.161**	**-0.118**	**0.025**	**2.48E-06**	**0.012**	**UTR3**	**IL17RD**		
**rs6787279**	**rs17057718**	**3:57136585**	**T**	**C**	**0.161**	**-0.117**	**0.025**	**2.51E-06**	**0.012**	**exonic**	**IL17RD**		
rs6787279	rs1075398	3:57137596	C	A	0.161	-0.118	0.025	2.32E-06	0.013	intronic	*IL17RD*		
rs6787279	rs1077034	3:57138228	A	T	0.161	-0.118	0.025	2.25E-06	0.013	intronic	*IL17RD*		
rs6787279	rs76645245	3:57140794	G	A	0.161	-0.118	0.025	2.11E-06	0.014	intronic	*IL17RD*		
rs6787279	rs78345417	3:57143942	T	C	0.161	-0.119	0.025	1.97E-06	0.015	intronic	*IL17RD*		
rs6787279	rs79932559	3:57145726	T	C	0.161	-0.119	0.025	2.00E-06	0.015	intronic	*IL17RD*		
rs6787279	rs77543785	3:57148271	T	C	0.161	-0.119	0.025	2.01E-06	0.015	intronic	*IL17RD*		
rs6787279	rs6792236	3:57149315	G	T	0.161	-0.119	0.025	1.98E-06	0.015	intronic	*IL17RD*		
rs6787279	rs80095764	3:57149793	T	C	0.161	-0.119	0.025	1.95E-06	0.015	intronic	*IL17RD*		
rs6787279	rs1488123	3:57151812	A	G	0.161	-0.119	0.025	1.98E-06	0.015	intronic	*IL17RD*		
rs6787279	rs1386832	3:57152006	G	A	0.161	-0.119	0.025	1.99E-06	0.015	intronic	*IL17RD*		
rs6787279	rs1386833	3:57152041	T	C	0.162	-0.115	0.025	3.92E-06	0.008	intronic	*IL17RD*		
rs6787279	rs747089	3:57153869	T	C	0.161	-0.119	0.025	1.90E-06	0.015	intronic	*IL17RD*		
rs6787279	rs1872942	3:57154878	T	C	0.161	-0.119	0.025	1.93E-06	0.015	intronic	*IL17RD*		
rs6787279	rs111888204	3:57157978	T	C	0.161	-0.117	0.025	2.77E-06	0.011	intronic	*IL17RD*		
rs6787279	rs6768368	3:57162476	T	C	0.137	-0.116	0.027	1.49E-05	0.002	intronic	*IL17RD*		
rs6787279	rs6787279	3:57163751	C	T	0.169	-0.128	0.025	2.11E-07	0.123	intronic	*IL17RD*		
rs6787279	rs6772089	3:57163765	T	C	0.137	-0.116	0.027	1.41E-05	0.002	intronic	*IL17RD*		
rs6787279	rs6796041	3:57163887	T	G	0.137	-0.118	0.027	1.00E-05	0.003	intronic	*IL17RD*		
rs6787279	rs6775080	3:57164362	T	C	0.137	-0.116	0.027	1.48E-05	0.002	intronic	*IL17RD*		

rs6787279	rs6787775	3:57164408	T	A	0.137	-0.116	0.027	1.49E-05	0.002	intronic	*IL17RD*		
rs6787279	rs34313207	3:57164929	A	G	0.137	-0.118	0.027	9.89E-06	0.003	intronic	*IL17RD*		
rs6787279	rs4681957	3:57165310	A	C	0.137	-0.116	0.027	1.47E-05	0.002	intronic	*IL17RD*		
rs6787279	rs6803905	3:57168273	C	T	0.169	-0.127	0.025	2.31E-07	0.112	intronic	*IL17RD*		
rs6787279	rs6788981	3:57168341	T	C	0.137	-0.118	0.027	1.00E-05	0.003	intronic	*IL17RD*		
rs6787279	rs56164953	3:57168793	A	G	0.137	-0.118	0.027	9.95E-06	0.003	intronic	*IL17RD*		
rs6787279	rs6807593	3:57169648	C	T	0.137	-0.116	0.027	1.55E-05	0.002	intronic	*IL17RD*		
rs6787279	rs1488121	3:57170495	C	G	0.137	-0.118	0.027	9.75E-06	0.003	intronic	*IL17RD*		
rs6787279	rs6774301	3:57171554	C	G	0.137	-0.116	0.027	1.55E-05	0.002	intronic	*IL17RD*		
rs6787279	rs6445858	3:57171902	C	T	0.137	-0.118	0.027	9.98E-06	0.003	intronic	*IL17RD*		
rs6787279	rs79625814	3:57173021	T	A	0.137	-0.118	0.027	9.75E-06	0.003	intronic	*IL17RD*		
rs6787279	rs1906504	3:57175454	G	A	0.137	-0.116	0.027	1.47E-05	0.002	intronic	*IL17RD*		
rs6787279	rs1386828	3:57176197	A	G	0.137	-0.118	0.027	9.84E-06	0.003	intronic	*IL17RD*		
rs6787279	rs1386829	3:57176505	G	A	0.137	-0.118	0.027	9.81E-06	0.003	intronic	*IL17RD*		
rs6787279	rs12496376	3:57177171	G	A	0.137	-0.116	0.027	1.50E-05	0.002	intronic	*IL17RD*		
rs6787279	rs6801574	3:57177195	T	C	0.169	-0.127	0.025	2.41E-07	0.108	intronic	*IL17RD*		
rs6787279	rs76208275	3:57177716	C	T	0.137	-0.116	0.027	1.51E-05	0.002	intronic	*IL17RD*		
rs6787279	rs12494525	3:57178293	T	C	0.137	-0.115	0.027	1.66E-05	0.002	intronic	*IL17RD*		
rs6787279	rs6808185	3:57179261	G	C	0.137	-0.118	0.027	1.02E-05	0.003	intronic	*IL17RD*		
rs6787279	rs17057784	3:57180402	A	C	0.138	-0.118	0.027	9.37E-06	0.004	intronic	*IL17RD*		
rs6787279	rs9823843	3:57181631	C	T	0.137	-0.118	0.027	1.01E-05	0.003	intronic	*IL17RD*		
rs6787279	rs17216900	3:57183877	C	T	0.170	-0.122	0.025	6.90E-07	0.040	intronic	*IL17RD*		
rs6787279	rs4475007	3:57186159	G	A	0.232	-0.096	0.022	1.25E-05	0.002	intronic	*IL17RD*		
rs6787279	rs3968279	3:57187078	C	T	0.120	-0.134	0.028	2.66E-06	0.012	intronic	*IL17RD*		
rs6787279	rs9823220	3:57190211	T	G	0.119	-0.135	0.029	2.16E-06	0.015	intronic	*IL17RD*		
rs6787279	rs7431253	3:57190452	G	A	0.119	-0.133	0.028	3.23E-06	0.010	intronic	*IL17RD*		
rs6787279	rs35424480	3:57190811	C	G	0.120	-0.133	0.028	2.86E-06	0.011	intronic	*IL17RD*		
rs6787279	rs1906505	3:57194272	T	C	0.119	-0.134	0.029	2.75E-06	0.012	intronic	*IL17RD*		55
**rs16903574**	**rs16903574**	**5:14610309**	**G**	**C**	**0.077**	**0.208**	**0.035**	**3.27E-09**	**0.999**	**exonic**	**FAM105A**		1
rs80101740	rs80101740	5:98471135	C	A	0.015	0.367	0.076	1.52E-06	0.774	intergenic	*LOC100289230;LINC02113*	dist=204422;dist=398559	
rs80101740	rs138827081	5:98511774	A	G	0.013	0.347	0.083	2.61E-05	0.065	intergenic	*LOC100289230;LINC02113*	dist=245061;dist=357920	
rs80101740	rs183720747	5:98516580	G	T	0.013	0.350	0.083	2.20E-05	0.075	intergenic	*LOC100289230;LINC02113*	dist=249867;dist=353114	
rs80101740	rs144009750	5:98530592	T	C	0.013	0.334	0.082	4.30E-05	0.044	intergenic	*LOC100289230;LINC02113*	dist=263879;dist=339102	
rs80101740	rs149035101	5:98534794	G	A	0.013	0.333	0.082	4.65E-05	0.041	intergenic	*LOC100289230;LINC02113*	dist=268081;dist=334900	5
rs1837253	rs1837253	5:110401872	C	T	0.739	0.200	0.021	1.91E-21	1.000	intergenic	*SLC25A46;TSLP*	dist=300998;dist=5518	1
rs3749833	rs59041913	5:131788135	T	C	0.232	0.137	0.022	3.51E-10	0.002	ncRNA_intronic	*C5orf56*		
rs3749833	rs7713065	5:131788334	C	A	0.737	-0.146	0.021	3.01E-12	0.199	ncRNA_intronic	*C5orf56*		
rs3749833	rs10069798	5:131788421	A	T	0.232	0.136	0.022	4.10E-10	0.002	ncRNA_intronic	*C5orf56*		
rs3749833	rs1023518	5:131793772	T	G	0.263	0.145	0.021	4.49E-12	0.135	ncRNA_intronic	*C5orf56*		

rs3749833	rs3857440	5:131794069	A	G	0.263	0.145	0.021	3.73E-12	0.161	ncRNA_intronic	*C5orf56*		
rs3749833	rs3749833	5:131799626	C	T	0.263	0.147	0.021	2.00E-12	0.295	ncRNA_intronic	*C5orf56*		
rs3749833	rs11748326	5:131799959	T	C	0.237	0.140	0.022	8.39E-11	0.008	ncRNA_intronic	*C5orf56*		
rs3749833	rs10077785	5:131801158	T	C	0.237	0.140	0.022	9.28E-11	0.007	ncRNA_intronic	*C5orf56*		
rs3749833	rs10067603	5:131803868	G	A	0.232	0.139	0.022	1.60E-10	0.004	ncRNA_intronic	*C5orf56*		
rs3749833	rs10060626	5:131803967	G	T	0.240	0.139	0.022	1.49E-10	0.005	ncRNA_intronic	*C5orf56*		
rs3749833	rs10039559	5:131804507	C	T	0.232	0.139	0.022	1.67E-10	0.004	ncRNA_intronic	*C5orf56*		
rs3749833	rs3846729	5:131806768	C	G	0.232	0.139	0.022	1.49E-10	0.005	ncRNA_intronic	*C5orf56*		
rs3749833	rs2548993	5:131808869	A	G	0.722	-0.138	0.021	2.24E-11	0.028	ncRNA_intronic	*C5orf56*		
rs3749833	rs2057656	5:131809305	C	T	0.732	-0.144	0.021	4.51E-12	0.134	ncRNA_intronic	*C5orf56*		
rs3749833	rs2057657	5:131809316	G	A	0.232	0.138	0.022	2.26E-10	0.003	ncRNA_intronic	*C5orf56*		15
**rs9273410**	**rs9273410**	**6:32627250**	**A**	**C**	**0.445**	**0.213**	**0.020**	**1.55E-27**	**0.999**	**UTR3**	**HLA-DQB1**		1
rs2584662	rs2671654	17:47468011	G	A	0.420	-0.104	0.019	2.84E-08	0.146	intergenic	*LOC102724596;PHB*	dist=10555;dist=13399	
rs2584662	rs2584663	17:47468147	A	G	0.351	-0.105	0.019	5.55E-08	0.078	intergenic	*LOC102724596;PHB*	dist=10691;dist=13263	
rs2584662	rs2412099	17:47469186	A	G	0.420	-0.104	0.019	2.74E-08	0.151	intergenic	*LOC102724596;PHB*	dist=11730;dist=12224	
rs2584662	rs9916130	17:47469229	G	C	0.352	-0.105	0.019	5.66E-08	0.077	intergenic	*LOC102724596;PHB*	dist=11773;dist=12181	
rs2584662	rs2584662	17:47470487	C	A	0.420	-0.104	0.019	2.42E-08	0.170	intergenic	*LOC102724596;PHB*	dist=13031;dist=10923	
rs2584662	rs2584661	17:47470534	A	G	0.347	-0.108	0.019	3.09E-08	0.138	intergenic	*LOC102724596;PHB*	dist=13078;dist=10876	
rs2584662	rs2671657	17:47473616	A	G	0.420	-0.103	0.019	3.38E-08	0.123	intergenic	*LOC102724596;PHB*	dist=16160;dist=7794	
rs2584662	rs2671659	17:47474529	A	G	0.347	-0.107	0.019	3.66E-08	0.117	intergenic	*LOC102724596;PHB*	dist=17073;dist=6881	8

e-Table 9: Results of eQTL analysis for SNPs in 99% credible set at 5% FDR level

eqtl_p=p-value in eQTL analysis; eqtl_coded=coded allele in eQTL analysis; expr_sign_eqtl_coded=expression in terms of eQTL coded allele (+=increased, -=decreased); expr_sign_A1 (expression change in terms of A1 (+=increased, -=decreased))

sentinel	SNP	CHR	BP	A1	A2	A1FREQ	BETA	SE	P	postprob	tissue	gene	eqtl_p	eqtl_coded	expr_sign_eqtl_coded	expr_sign_A1
rs35570272	rs7646283	3	33046480	T	C	0.368	0.097	0.019	4.40E-07	0.133	Lung	*CRTAP*	0.000455	C	+	-
rs35570272	rs34064757	3	33047283	A	G	0.368	0.097	0.019	4.61E-07	0.127	Lung	*CRTAP*	0.000455	G	+	-
rs6787279	rs74999629	3	57117542	A	G	0.161	-0.118	0.025	2.33E-06	0.013	GTEx_lung	*IL17RD*	2.27E-09	A	+	+
rs6787279	rs74932439	3	57115567	T	C	0.162	-0.119	0.025	1.90E-06	0.015	GTEx_lung	*IL17RD*	3.15E-09	T	+	+
rs6787279	rs59527464	3	57122780	T	C	0.198	-0.118	0.023	3.67E-07	0.070	GTEx_lung	*IL17RD*	1.44E-08	T	+	+
rs6787279	rs59527464	3	57122780	T	C	0.198	-0.118	0.023	3.67E-07	0.070	Lung	*IL17RD*	1.40E-05	C	-	+
rs6787279	rs74932439	3	57115567	T	C	0.162	-0.119	0.025	1.90E-06	0.015	Lung	*IL17RD*	1.95E-04	C	-	+
rs6787279	rs74999629	3	57117542	A	G	0.161	-0.118	0.025	2.33E-06	0.013	Lung	*IL17RD*	0.000195	G	-	+
rs6787279	rs12486855	3	57125864	G	A	0.161	-0.118	0.025	2.48E-06	0.012	GTEx_lung	*IL17RD*	3.11E-09	G	+	+
rs6787279	rs2035655	3	57125202	G	T	0.198	-0.118	0.023	3.37E-07	0.075	GTEx_lung	*IL17RD*	1.29E-08	G	+	+
rs6787279	rs17289035	3	57125424	A	G	0.198	-0.118	0.023	3.37E-07	0.075	GTEx_lung	*IL17RD*	1.29E-08	A	+	+
rs6787279	rs2035656	3	57125101	C	A	0.161	-0.119	0.025	1.82E-06	0.016	GTEx_lung	*IL17RD*	1.39E-08	C	+	+
rs6787279	rs2035655	3	57125202	G	T	0.198	-0.118	0.023	3.37E-07	0.075	Lung	*IL17RD*	1.40E-05	T	-	+
rs6787279	rs17289035	3	57125424	A	G	0.198	-0.118	0.023	3.37E-07	0.075	Lung	*IL17RD*	1.40E-05	G	-	+
rs6787279	rs2035656	3	57125101	C	A	0.161	-0.119	0.025	1.82E-06	0.016	Lung	*IL17RD*	1.95E-04	A	-	+
rs6787279	rs12486855	3	57125864	G	A	0.161	-0.118	0.025	2.48E-06	0.012	Lung	*IL17RD*	0.000195	A	-	+
rs6787279	rs17057718	3	57136585	T	C	0.161	-0.117	0.025	2.51E-06	0.012	GTEx_lung	*IL17RD*	4.63E-09	T	+	+
rs6787279	rs1075398	3	57137596	C	A	0.161	-0.118	0.025	2.32E-06	0.013	GTEx_lung	*IL17RD*	4.63E-09	C	+	+
rs6787279	rs1077034	3	57138228	A	T	0.161	-0.118	0.025	2.25E-06	0.013	GTEx_lung	*IL17RD*	4.63E-09	A	+	+
rs6787279	rs76645245	3	57140794	G	A	0.161	-0.118	0.025	2.11E-06	0.014	GTEx_lung	*IL17RD*	4.63E-09	G	+	+
rs6787279	rs78345417	3	57143942	T	C	0.161	-0.119	0.025	1.97E-06	0.015	GTEx_lung	*IL17RD*	1.70E-08	T	+	+
rs6787279	rs1077034	3	57138228	A	T	0.161	-0.118	0.025	2.25E-06	0.013	Lung	*IL17RD*	9.37E-05	T	-	+
rs6787279	rs76645245	3	57140794	G	A	0.161	-0.118	0.025	2.11E-06	0.014	Lung	*IL17RD*	9.37E-05	A	-	+
rs6787279	rs78345417	3	57143942	T	C	0.161	-0.119	0.025	1.97E-06	0.015	Lung	*IL17RD*	9.37E-05	C	-	+
rs6787279	rs17057718	3	57136585	T	C	0.161	-0.117	0.025	2.51E-06	0.012	Lung	*IL17RD*	1.38E-04	C	-	+
rs6787279	rs1075398	3	57137596	C	A	0.161	-0.118	0.025	2.32E-06	0.013	Lung	*IL17RD*	0.000138	A	-	+
rs6787279	rs79932559	3	57145726	T	C	0.161	-0.119	0.025	2.00E-06	0.015	GTEx_lung	*IL17RD*	4.63E-09	T	+	+
rs6787279	rs1488123	3	57151812	A	G	0.161	-0.119	0.025	1.98E-06	0.015	GTEx_lung	*IL17RD*	4.63E-09	A	+	+
rs6787279	rs1386832	3	57152006	G	A	0.161	-0.119	0.025	1.99E-06	0.015	GTEx_lung	*IL17RD*	4.63E-09	G	+	+
rs6787279	rs1386833	3	57152041	T	C	0.162	-0.115	0.025	3.92E-06	0.008	GTEx_lung	*IL17RD*	4.63E-09	T	+	+
rs6787279	rs747089	3	57153869	T	C	0.161	-0.119	0.025	1.90E-06	0.015	GTEx_lung	*IL17RD*	4.63E-09	T	+	+
rs6787279	rs1872942	3	57154878	T	C	0.161	-0.119	0.025	1.93E-06	0.015	GTEx_lung	*IL17RD*	4.63E-09	T	+	+
rs6787279	rs77543785	3	57148271	T	C	0.161	-0.119	0.025	2.01E-06	0.015	GTEx_lung	*IL17RD*	3.95E-08	T	+	+
rs6787279	rs6792236	3	57149315	G	T	0.161	-0.119	0.025	1.98E-06	0.015	GTEx_lung	*IL17RD*	3.95E-08	G	+	+
rs6787279	rs80095764	3	57149793	T	C	0.161	-0.119	0.025	1.95E-06	0.015	GTEx_lung	*IL17RD*	3.95E-08	T	+	+
rs6787279	rs79932559	3	57145726	T	C	0.161	-0.119	0.025	2.00E-06	0.015	Lung	*IL17RD*	9.37E-05	C	-	+

sentinel	SNP	CHR	BP	A1	A2	A1FREQ	BETA	SE	P	postprob	tissue	gene	eqtl_p	eqtl_coded	expr_sign_eqtl_coded	expr_sign_A1
rs6787279	rs77543785	3	57148271	T	C	0.161	-0.119	0.025	2.01E-06	0.015	Lung	*IL17RD*	9.37E-05	C	-	+
rs6787279	rs80095764	3	57149793	T	C	0.161	-0.119	0.025	1.95E-06	0.015	Lung	*IL17RD*	9.37E-05	C	-	+
rs6787279	rs1488123	3	57151812	A	G	0.161	-0.119	0.025	1.98E-06	0.015	Lung	*IL17RD*	9.37E-05	G	-	+
rs6787279	rs1386832	3	57152006	G	A	0.161	-0.119	0.025	1.99E-06	0.015	Lung	*IL17RD*	9.37E-05	A	-	+
rs6787279	rs1386833	3	57152041	T	C	0.162	-0.115	0.025	3.92E-06	0.008	Lung	*IL17RD*	9.37E-05	C	-	+
rs6787279	rs747089	3	57153869	T	C	0.161	-0.119	0.025	1.90E-06	0.015	Lung	*IL17RD*	9.37E-05	C	-	+
rs6787279	rs6792236	3	57149315	G	T	0.161	-0.119	0.025	1.98E-06	0.015	Lung	*IL17RD*	1.38E-04	T	-	+
rs6787279	rs1872942	3	57154878	T	C	0.161	-0.119	0.025	1.93E-06	0.015	Lung	*IL17RD*	0.000138	C	-	+
rs6787279	rs111888204	3	57157978	T	C	0.161	-0.117	0.025	2.77E-06	0.011	GTEx_lung	*IL17RD*	7.85E-09	T	+	+
rs6787279	rs6768368	3	57162476	T	C	0.137	-0.116	0.027	1.49E-05	0.002	GTEx_lung	*IL17RD*	7.45E-08	T	+	+
rs6787279	rs6772089	3	57163765	T	C	0.137	-0.116	0.027	1.41E-05	0.002	GTEx_lung	*IL17RD*	1.35E-07	T	+	+
rs6787279	rs6775080	3	57164362	T	C	0.137	-0.116	0.027	1.48E-05	0.002	GTEx_lung	*IL17RD*	1.35E-07	T	+	+
rs6787279	rs6787775	3	57164408	T	A	0.137	-0.116	0.027	1.49E-05	0.002	GTEx_lung	*IL17RD*	1.35E-07	T	+	+
rs6787279	rs6787279	3	57163751	C	T	0.169	-0.128	0.025	2.11E-07	0.123	Lung	*IL17RD*	4.07E-07	T	-	+
rs6787279	rs6787279	3	57163751	C	T	0.169	-0.128	0.025	2.11E-07	0.123	GTEx_lung	*IL17RD*	1.82E-06	C	+	+
rs6787279	rs34313207	3	57164929	A	G	0.137	-0.118	0.027	9.89E-06	0.003	Lung	*IL17RD*	2.31E-06	G	-	+
rs6787279	rs6768368	3	57162476	T	C	0.137	-0.116	0.027	1.49E-05	0.002	Lung	*IL17RD*	3.19E-06	C	-	+
rs6787279	rs6772089	3	57163765	T	C	0.137	-0.116	0.027	1.41E-05	0.002	Lung	*IL17RD*	3.19E-06	C	-	+
rs6787279	rs6796041	3	57163887	T	G	0.137	-0.118	0.027	1.00E-05	0.003	Lung	*IL17RD*	3.19E-06	G	-	+
rs6787279	rs6787775	3	57164408	T	A	0.137	-0.116	0.027	1.49E-05	0.002	Lung	*IL17RD*	3.19E-06	A	-	+
rs6787279	rs6796041	3	57163887	T	G	0.137	-0.118	0.027	1.00E-05	0.003	GTEx_lung	*IL17RD*	3.47E-06	T	+	+
rs6787279	rs34313207	3	57164929	A	G	0.137	-0.118	0.027	9.89E-06	0.003	GTEx_lung	*IL17RD*	3.47E-06	A	+	+
rs6787279	rs6775080	3	57164362	T	C	0.137	-0.116	0.027	1.48E-05	0.002	Lung	*IL17RD*	5.34E-06	C	-	+
rs6787279	rs111888204	3	57157978	T	C	0.161	-0.117	0.025	2.77E-06	0.011	Lung	*IL17RD*	1.27E-04	C	-	+
rs6787279	rs4681957	3	57165310	A	C	0.137	-0.116	0.027	1.47E-05	0.002	GTEx_lung	*IL17RD*	1.35E-07	A	+	+
rs6787279	rs6803905	3	57168273	C	T	0.169	-0.127	0.025	2.31E-07	0.112	Lung	*IL17RD*	3.00E-07	T	-	+
rs6787279	rs6807593	3	57169648	C	T	0.137	-0.116	0.027	1.55E-05	0.002	GTEx_lung	*IL17RD*	1.32E-06	C	+	+
rs6787279	rs6803905	3	57168273	C	T	0.169	-0.127	0.025	2.31E-07	0.112	GTEx_lung	*IL17RD*	1.82E-06	C	+	+
rs6787279	rs6774301	3	57171554	C	G	0.137	-0.116	0.027	1.55E-05	0.002	GTEx_lung	*IL17RD*	1.92E-06	C	+	+
rs6787279	rs6788981	3	57168341	T	C	0.137	-0.118	0.027	1.00E-05	0.003	Lung	*IL17RD*	2.31E-06	C	-	+
rs6787279	rs56164953	3	57168793	A	G	0.137	-0.118	0.027	9.95E-06	0.003	Lung	*IL17RD*	2.31E-06	G	-	+
rs6787279	rs1488121	3	57170495	C	G	0.137	-0.118	0.027	9.75E-06	0.003	Lung	*IL17RD*	2.31E-06	G	-	+
rs6787279	rs6774301	3	57171554	C	G	0.137	-0.116	0.027	1.55E-05	0.002	Lung	*IL17RD*	2.31E-06	G	-	+
rs6787279	rs79625814	3	57173021	T	A	0.137	-0.118	0.027	9.75E-06	0.003	Lung	*IL17RD*	2.31E-06	A	-	+
rs6787279	rs1488121	3	57170495	C	G	0.137	-0.118	0.027	9.75E-06	0.003	GTEx_lung	*IL17RD*	2.81E-06	C	+	+
rs6787279	rs6445858	3	57171902	C	T	0.137	-0.118	0.027	9.98E-06	0.003	GTEx_lung	*IL17RD*	2.81E-06	C	+	+
rs6787279	rs6788981	3	57168341	T	C	0.137	-0.118	0.027	1.00E-05	0.003	GTEx_lung	*IL17RD*	3.47E-06	T	+	+
rs6787279	rs4681957	3	57165310	A	C	0.137	-0.116	0.027	1.47E-05	0.002	Lung	*IL17RD*	3.91E-06	C	-	+
rs6787279	rs6807593	3	57169648	C	T	0.137	-0.116	0.027	1.55E-05	0.002	Lung	*IL17RD*	3.91E-06	T	-	+
rs6787279	rs6445858	3	57171902	C	T	0.137	-0.118	0.027	9.98E-06	0.003	Lung	*IL17RD*	3.91E-06	T	-	+

sentinel	SNP	CHR	BP	A1	A2	A1FREQ	BETA	SE	P	postprob	tissue	gene	eqtl_p	eqtl_coded	expr_sign_eqtl_coded	expr_sign_A1
rs6787279	rs56164953	3	57168793	A	G	0.137	-0.118	0.027	9.95E-06	0.003	GTEx_lung	*IL17RD*	6.67E-06	A	+	+
rs6787279	rs79625814	3	57173021	T	A	0.137	-0.118	0.027	9.75E-06	0.003	GTEx_lung	*IL17RD*	7.70E-06	T	+	+
rs6787279	rs12494525	3	57178293	T	C	0.137	-0.115	0.027	1.66E-05	0.002	GTEx_lung	*IL17RD*	9.49E-08	T	+	+
rs6787279	rs1906504	3	57175454	G	A	0.137	-0.116	0.027	1.47E-05	0.002	GTEx_lung	*IL17RD*	1.70E-07	G	+	+
rs6787279	rs12496376	3	57177171	G	A	0.137	-0.116	0.027	1.50E-05	0.002	GTEx_lung	*IL17RD*	1.70E-07	G	+	+
rs6787279	rs6801574	3	57177195	T	C	0.169	-0.127	0.025	2.41E-07	0.108	Lung	*IL17RD*	3.00E-07	C	-	+
rs6787279	rs76208275	3	57177716	C	T	0.137	-0.116	0.027	1.51E-05	0.002	GTEx_lung	*IL17RD*	3.42E-07	C	+	+
rs6787279	rs17057784	3	57180402	A	C	0.138	-0.118	0.027	9.37E-06	0.004	GTEx_lung	*IL17RD*	7.99E-07	A	+	+
rs6787279	rs17216900	3	57183877	C	T	0.170	-0.122	0.025	6.90E-07	0.040	Lung	*IL17RD*	1.30E-06	T	-	+
rs6787279	rs6801574	3	57177195	T	C	0.169	-0.127	0.025	2.41E-07	0.108	GTEx_lung	*IL17RD*	1.48E-06	T	+	+
rs6787279	rs9823843	3	57181631	C	T	0.137	-0.118	0.027	1.01E-05	0.003	GTEx_lung	*IL17RD*	1.92E-06	C	+	+
rs6787279	rs1386828	3	57176197	A	G	0.137	-0.118	0.027	9.84E-06	0.003	Lung	*IL17RD*	2.31E-06	G	-	+
rs6787279	rs1386829	3	57176505	G	A	0.137	-0.118	0.027	9.81E-06	0.003	Lung	*IL17RD*	2.31E-06	A	-	+
rs6787279	rs76208275	3	57177716	C	T	0.137	-0.116	0.027	1.51E-05	0.002	Lung	*IL17RD*	2.31E-06	T	-	+
rs6787279	rs12494525	3	57178293	T	C	0.137	-0.115	0.027	1.66E-05	0.002	Lung	*IL17RD*	2.31E-06	C	-	+
rs6787279	rs6808185	3	57179261	G	C	0.137	-0.118	0.027	1.02E-05	0.003	Lung	*IL17RD*	2.31E-06	C	-	+
rs6787279	rs17216900	3	57183877	C	T	0.170	-0.122	0.025	6.90E-07	0.040	GTEx_lung	*IL17RD*	2.54E-06	C	+	+
rs6787279	rs12496376	3	57177171	G	A	0.137	-0.116	0.027	1.50E-05	0.002	Lung	*IL17RD*	2.62E-06	A	-	+
rs6787279	rs1386828	3	57176197	A	G	0.137	-0.118	0.027	9.84E-06	0.003	GTEx_lung	*IL17RD*	2.73E-06	A	+	+
rs6787279	rs1386829	3	57176505	G	A	0.137	-0.118	0.027	9.81E-06	0.003	GTEx_lung	*IL17RD*	2.73E-06	G	+	+
rs6787279	rs6808185	3	57179261	G	C	0.137	-0.118	0.027	1.02E-05	0.003	GTEx_lung	*IL17RD*	2.81E-06	G	+	+
rs6787279	rs1906504	3	57175454	G	A	0.137	-0.116	0.027	1.47E-05	0.002	Lung	*IL17RD*	3.91E-06	A	-	+
rs6787279	rs17057784	3	57180402	A	C	0.138	-0.118	0.027	9.37E-06	0.004	Lung	*IL17RD*	4.89E-06	C	-	+
rs6787279	rs9823843	3	57181631	C	T	0.137	-0.118	0.027	1.01E-05	0.003	Lung	*IL17RD*	8.08E-06	T	-	+
rs6787279	rs1906505	3	57194272	T	C	0.119	-0.134	0.029	2.75E-06	0.012	GTEx_lung	*IL17RD*	5.22E-08	T	+	+
rs6787279	rs35424480	3	57190811	C	G	0.120	-0.133	0.028	2.86E-06	0.011	GTEx_lung	*IL17RD*	5.75E-08	C	+	+
rs6787279	rs3968279	3	57187078	C	T	0.120	-0.134	0.028	2.66E-06	0.012	GTEx_lung	*IL17RD*	9.77E-08	C	+	+
rs6787279	rs9823220	3	57190211	T	G	0.119	-0.135	0.029	2.16E-06	0.015	GTEx_lung	*IL17RD*	1.29E-07	T	+	+
rs6787279	rs7431253	3	57190452	G	A	0.119	-0.133	0.028	3.23E-06	0.010	GTEx_lung	*IL17RD*	1.29E-07	G	+	+
rs6787279	rs9823220	3	57190211	T	G	0.119	-0.135	0.029	2.16E-06	0.015	Lung	*IL17RD*	1.36E-06	G	-	+
rs6787279	rs7431253	3	57190452	G	A	0.119	-0.133	0.028	3.23E-06	0.010	Lung	*IL17RD*	1.36E-06	A	-	+
rs6787279	rs1906505	3	57194272	T	C	0.119	-0.134	0.029	2.75E-06	0.012	Lung	*IL17RD*	1.36E-06	C	-	+
rs6787279	rs35424480	3	57190811	C	G	0.120	-0.133	0.028	2.86E-06	0.011	Lung	*IL17RD*	2.03E-06	G	-	+
rs6787279	rs3968279	3	57187078	C	T	0.120	-0.134	0.028	2.66E-06	0.012	Lung	*IL17RD*	2.29E-06	T	-	+
rs3749833	rs2057656	5	131809305	C	T	0.732	-0.144	0.021	4.51E-12	0.134	GTEx_whole_blood	*SLC22A5*	5.19E-17	C	-	-
rs3749833	rs2548993	5	131808869	A	G	0.722	-0.138	0.021	2.24E-11	0.028	GTEx_whole_blood	*SLC22A5*	6.41E-17	A	-	-
rs3749833	rs3749833	5	131799626	C	T	0.263	0.147	0.021	2.00E-12	0.295	GTEx_whole_blood	*SLC22A5*	1.59E-15	C	+	+
rs3749833	rs7713065	5	131788334	C	A	0.737	-0.146	0.021	3.01E-12	0.199	GTEx_whole_blood	*SLC22A5*	2.83E-15	C	-	-

sentinel	SNP	CHR	BP	A1	A2	A1FREQ	BETA	SE	P	postprob	tissue	gene	eqtl_p	eqtl_coded	expr_sign_eqtl_coded	expr_sign_A1
rs3749833	rs3857440	5	131794069	A	G	0.263	0.145	0.021	3.73E-12	0.161	GTEx_whole_blood	*SLC22A5*	3.66E-15	A	+	+
rs3749833	rs1023518	5	131793772	T	G	0.263	0.145	0.021	4.49E-12	0.135	GTEx_whole_blood	*SLC22A5*	6.88E-15	T	+	+
rs3749833	rs10039559	5	131804507	C	T	0.232	0.139	0.022	1.67E-10	0.004	GTEx_whole_blood	*SLC22A5*	6.45E-11	C	+	+
rs3749833	rs10069798	5	131788421	A	T	0.232	0.136	0.022	4.10E-10	0.002	GTEx_whole_blood	*SLC22A5*	9.66E-11	A	+	+
rs3749833	rs10067603	5	131803868	G	A	0.232	0.139	0.022	1.60E-10	0.004	GTEx_whole_blood	*SLC22A5*	1.50E-10	G	+	+
rs3749833	rs11748326	5	131799959	T	C	0.237	0.140	0.022	8.39E-11	0.008	GTEx_whole_blood	*SLC22A5*	1.64E-10	T	+	+
rs3749833	rs10077785	5	131801158	T	C	0.237	0.140	0.022	9.28E-11	0.007	GTEx_whole_blood	*SLC22A5*	1.64E-10	T	+	+
rs3749833	rs59041913	5	131788135	T	C	0.232	0.137	0.022	3.51E-10	0.002	GTEx_whole_blood	*SLC22A5*	1.97E-10	T	+	+
rs3749833	rs3846729	5	131806768	C	G	0.232	0.139	0.022	1.49E-10	0.005	GTEx_whole_blood	*SLC22A5*	2.12E-10	C	+	+
rs3749833	rs2057657	5	131809316	G	A	0.232	0.138	0.022	2.26E-10	0.003	GTEx_whole_blood	*SLC22A5*	2.12E-10	G	+	+
rs3749833	rs2548993	5	131808869	A	G	0.722	-0.138	0.021	2.24E-11	0.028	Lung	*SLC22A5*	3.63E-09	G	+	-
rs3749833	rs3749833	5	131799626	C	T	0.263	0.147	0.021	2.00E-12	0.295	Lung	*SLC22A5*	8.59E-09	T	-	+
rs3749833	rs1023518	5	131793772	T	G	0.263	0.145	0.021	4.49E-12	0.135	Lung	*SLC22A5*	1.53E-08	G	-	+
rs3749833	rs3857440	5	131794069	A	G	0.263	0.145	0.021	3.73E-12	0.161	Lung	*SLC22A5*	1.53E-08	G	-	+
rs3749833	rs7713065	5	131788334	C	A	0.737	-0.146	0.021	3.01E-12	0.199	Lung	*SLC22A5*	2.07E-08	A	+	-
rs3749833	rs2057656	5	131809305	C	T	0.732	-0.144	0.021	4.51E-12	0.134	Lung	*SLC22A5*	3.27E-08	T	+	-
rs3749833	rs3749833	5	131799626	C	T	0.263	0.147	0.021	2.00E-12	0.295	Lung	Unknown mRNA	5.32E-08	T	-	+
rs3749833	rs1023518	5	131793772	T	G	0.263	0.145	0.021	4.49E-12	0.135	Lung	Unknown mRNA	5.37E-08	G	-	+
rs3749833	rs3857440	5	131794069	A	G	0.263	0.145	0.021	3.73E-12	0.161	Lung	Unknown mRNA	5.37E-08	G	-	+
rs3749833	rs2057656	5	131809305	C	T	0.732	-0.144	0.021	4.51E-12	0.134	Lung	Unknown mRNA	9.23E-08	T	+	-
rs3749833	rs7713065	5	131788334	C	A	0.737	-0.146	0.021	3.01E-12	0.199	Lung	Unknown mRNA	9.26E-08	A	+	-
rs3749833	rs2057656	5	131809305	C	T	0.732	-0.144	0.021	4.51E-12	0.134	GTEx_whole_blood	*AC116366.6*	1.15E-07	C	+	+
rs3749833	rs2548993	5	131808869	A	G	0.722	-0.138	0.021	2.24E-11	0.028	GTEx_whole_blood	*AC116366.6*	2.58E-07	A	+	+
rs3749833	rs3857440	5	131794069	A	G	0.263	0.145	0.021	3.73E-12	0.161	GTEx_whole_blood	*AC116366.6*	5.99E-07	A	-	-

sentinel	SNP	CHR	BP	A1	A2	A1FREQ	BETA	SE	P	postprob	tissue	gene	eqtl_p	eqtl_coded	expr_sign_eqtl_coded	expr_sign_A1
rs3749833	rs1023518	5	131793772	T	G	0.263	0.145	0.021	4.49E-12	0.135	GTEx_whole_blood	*AC116366.6*	7.55E-07	T	-	-
rs3749833	rs2548993	5	131808869	A	G	0.722	-0.138	0.021	2.24E-11	0.028	Lung	Unknown mRNA	7.91E-07	G	+	-
rs3749833	rs10060626	5	131803967	G	T	0.240	0.139	0.022	1.49E-10	0.005	Lung	*SLC22A5*	1.91E-06	T	-	+
rs3749833	rs3749833	5	131799626	C	T	0.263	0.147	0.021	2.00E-12	0.295	GTEx_whole_blood	*AC116366.6*	2.12E-06	C	-	-
rs3749833	rs7713065	5	131788334	C	A	0.737	-0.146	0.021	3.01E-12	0.199	GTEx_whole_blood	*AC116366.6*	2.61E-06	C	+	+
rs3749833	rs10060626	5	131803967	G	T	0.240	0.139	0.022	1.49E-10	0.005	Lung	Unknown mRNA	2.84E-06	T	-	+
rs3749833	rs11748326	5	131799959	T	C	0.237	0.140	0.022	8.39E-11	0.008	Lung	Unknown mRNA	2.89E-06	C	-	+
rs3749833	rs10077785	5	131801158	T	C	0.237	0.140	0.022	9.28E-11	0.007	Lung	Unknown mRNA	2.89E-06	C	-	+
rs3749833	rs10067603	5	131803868	G	A	0.232	0.139	0.022	1.60E-10	0.004	Lung	*SLC22A5*	2.91E-06	A	-	+
rs3749833	rs10039559	5	131804507	C	T	0.232	0.139	0.022	1.67E-10	0.004	Lung	*SLC22A5*	2.91E-06	T	-	+
rs3749833	rs3846729	5	131806768	C	G	0.232	0.139	0.022	1.49E-10	0.005	Lung	*SLC22A5*	2.91E-06	G	-	+
rs3749833	rs59041913	5	131788135	T	C	0.232	0.137	0.022	3.51E-10	0.002	Lung	*SLC22A5*	3.14E-06	C	-	+
rs3749833	rs10069798	5	131788421	A	T	0.232	0.136	0.022	4.10E-10	0.002	Lung	*SLC22A5*	3.14E-06	T	-	+
rs3749833	rs10067603	5	131803868	G	A	0.232	0.139	0.022	1.60E-10	0.004	Lung	Unknown mRNA	3.59E-06	A	-	+
rs3749833	rs10039559	5	131804507	C	T	0.232	0.139	0.022	1.67E-10	0.004	Lung	Unknown mRNA	3.59E-06	T	-	+
rs3749833	rs3846729	5	131806768	C	G	0.232	0.139	0.022	1.49E-10	0.005	Lung	Unknown mRNA	3.59E-06	G	-	+
rs3749833	rs11748326	5	131799959	T	C	0.237	0.140	0.022	8.39E-11	0.008	Lung	*SLC22A5*	3.63E-06	C	-	+
rs3749833	rs10077785	5	131801158	T	C	0.237	0.140	0.022	9.28E-11	0.007	Lung	*SLC22A5*	3.63E-06	C	-	+
rs3749833	rs2057657	5	131809316	G	A	0.232	0.138	0.022	2.26E-10	0.003	Lung	Unknown mRNA	4.26E-06	A	-	+
rs3749833	rs2057657	5	131809316	G	A	0.232	0.138	0.022	2.26E-10	0.003	Lung	*SLC22A5*	6.40E-06	A	-	+
rs3749833	rs59041913	5	131788135	T	C	0.232	0.137	0.022	3.51E-10	0.002	Lung	Unknown mRNA	8.65E-06	C	-	+
rs3749833	rs10069798	5	131788421	A	T	0.232	0.136	0.022	4.10E-10	0.002	Lung	Unknown mRNA	8.65E-06	T	-	+
rs3749833	rs3749833	5	131799626	C	T	0.263	0.147	0.021	2.00E-12	0.295	GTEx_lung	*SLC22A5*	2.03E-05	C	+	+
rs3749833	rs11748326	5	131799959	T	C	0.237	0.140	0.022	8.39E-11	0.008	GTEx_whole_blood	*AC116366.6*	2.09E-05	T	-	-
rs3749833	rs10077785	5	131801158	T	C	0.237	0.140	0.022	9.28E-11	0.007	GTEx_whole_blood	*AC116366.6*	2.09E-05	T	-	-
rs3749833	rs2548993	5	131808869	A	G	0.722	-0.138	0.021	2.24E-11	0.028	GTEx_lung	*SLC22A5*	2.42E-05	A	-	-

sentinel	SNP	CHR	BP	A1	A2	A1FREQ	BETA	SE	P	postprob	tissue	gene	eqtl_p	eqtl_coded	expr_sign_eqtl_coded	expr_sign_A1
rs3749833	rs3846729	5	131806768	C	G	0.232	0.139	0.022	1.49E-10	0.005	GTEx_whole_blood	*AC116366.6*	3.16E-05	C	-	-
rs3749833	rs2057657	5	131809316	G	A	0.232	0.138	0.022	2.26E-10	0.003	GTEx_whole_blood	*AC116366.6*	3.16E-05	G	-	-
rs3749833	rs10039559	5	131804507	C	T	0.232	0.139	0.022	1.67E-10	0.004	GTEx_whole_blood	*AC116366.6*	3.22E-05	C	-	-
rs3749833	rs3857440	5	131794069	A	G	0.263	0.145	0.021	3.73E-12	0.161	GTEx_lung	*SLC22A5*	3.31E-05	A	+	+
rs3749833	rs10067603	5	131803868	G	A	0.232	0.139	0.022	1.60E-10	0.004	GTEx_whole_blood	*AC116366.6*	3.57E-05	G	-	-
rs3749833	rs7713065	5	131788334	C	A	0.737	-0.146	0.021	3.01E-12	0.199	GTEx_lung	*SLC22A5*	3.60E-05	C	-	-
rs3749833	rs10069798	5	131788421	A	T	0.232	0.136	0.022	4.10E-10	0.002	GTEx_whole_blood	*AC116366.6*	4.07E-05	A	-	-
rs3749833	rs1023518	5	131793772	T	G	0.263	0.145	0.021	4.49E-12	0.135	GTEx_lung	*SLC22A5*	5.12E-05	T	+	+
rs3749833	rs2548993	5	131808869	A	G	0.722	-0.138	0.021	2.24E-11	0.028	Lung	*RAD50*	5.61E-05	G	+	-
rs3749833	rs2057656	5	131809305	C	T	0.732	-0.144	0.021	4.51E-12	0.134	GTEx_lung	*SLC22A5*	6.02E-05	C	-	-
rs3749833	rs59041913	5	131788135	T	C	0.232	0.137	0.022	3.51E-10	0.002	GTEx_whole_blood	*AC116366.6*	6.51E-05	T	-	-
rs3749833	rs11748326	5	131799959	T	C	0.237	0.140	0.022	8.39E-11	0.008	GTEx_lung	*Y_RNA*	7.33E-05	T	+	+
rs3749833	rs10077785	5	131801158	T	C	0.237	0.140	0.022	9.28E-11	0.007	GTEx_lung	*Y_RNA*	7.33E-05	T	+	+
rs3749833	rs2548993	5	131808869	A	G	0.722	-0.138	0.021	2.24E-11	0.028	GTEx_whole_blood	*Y_RNA*	7.70E-05	A	-	-
rs3749833	rs10067603	5	131803868	G	A	0.232	0.139	0.022	1.60E-10	0.004	GTEx_lung	*Y_RNA*	1.34E-04	G	+	+
rs3749833	rs3846729	5	131806768	C	G	0.232	0.139	0.022	1.49E-10	0.005	GTEx_lung	*Y_RNA*	0.000137	C	+	+
rs3749833	rs2057657	5	131809316	G	A	0.232	0.138	0.022	2.26E-10	0.003	GTEx_lung	*Y_RNA*	0.000137	G	+	+
rs3749833	rs2057656	5	131809305	C	T	0.732	-0.144	0.021	4.51E-12	0.134	GTEx_whole_blood	*Y_RNA*	0.000144	C	-	-
rs3749833	rs10039559	5	131804507	C	T	0.232	0.139	0.022	1.67E-10	0.004	GTEx_lung	*Y_RNA*	0.000167	C	+	+
rs3749833	rs10067603	5	131803868	G	A	0.232	0.139	0.022	1.60E-10	0.004	GTEx_lung	*SLC22A5*	0.000167	G	+	+
rs3749833	rs2057656	5	131809305	C	T	0.732	-0.144	0.021	4.51E-12	0.134	Lung	*RAD50*	0.000246	T	+	-
rs3749833	rs1023518	5	131793772	T	G	0.263	0.145	0.021	4.49E-12	0.135	Lung	*RAD50*	0.000663	G	-	+
rs3749833	rs3857440	5	131794069	A	G	0.263	0.145	0.021	3.73E-12	0.161	Lung	*RAD50*	0.000663	G	-	+
rs3749833	rs7713065	5	131788334	C	A	0.737	-0.146	0.021	3.01E-12	0.199	Lung	*RAD50*	0.000694	A	+	-
rs9273410	rs9273410	6	32627250	A	C	0.445	0.213	0.020	1.55E-27	0.999	GTEx_whole_blood	*HLA-DQB1*	########	A	-	-
rs9273410	rs9273410	6	32627250	A	C	0.445	0.213	0.020	1.55E-27	0.999	GTEx_whole_blood	*HLA-DQB2*	########	A	+	+
rs9273410	rs9273410	6	32627250	A	C	0.445	0.213	0.020	1.55E-27	0.999	GTEx_lung	*HLA-DQB2*	########	A	+	+
rs9273410	rs9273410	6	32627250	A	C	0.445	0.213	0.020	1.55E-27	0.999	GTEx_whole_blood	*HLA-DQA2*	7.65E-64	A	+	+
rs9273410	rs9273410	6	32627250	A	C	0.445	0.213	0.020	1.55E-27	0.999	GTEx_lung	*HLA-DQB1*	3.98E-55	A	-	-
rs9273410	rs9273410	6	32627250	A	C	0.445	0.213	0.020	1.55E-27	0.999	GTEx_lung	*HLA-DQA1*	9.50E-50	A	-	-

sentinel	SNP	CHR	BP	A1	A2	A1FREQ	BETA	SE	P	postprob	tissue	gene	eqtl_p	eqtl_coded	expr_sign_eqtl_coded	expr_sign_A1
rs9273410	rs9273410	6	32627250	A	C	0.445	0.213	0.020	1.55E-27	0.999	GTEx_lung	*HLA-DQA2*	9.05E-47	A	+	+
rs9273410	rs9273410	6	32627250	A	C	0.445	0.213	0.020	1.55E-27	0.999	GTEx_whole_blood	*HLA-DQA1*	4.95E-44	A	-	-
rs9273410	rs9273410	6	32627250	A	C	0.445	0.213	0.020	1.55E-27	0.999	Lung	*HLA-DRB5*	5.35E-42	C	+	-
rs9273410	rs9273410	6	32627250	A	C	0.445	0.213	0.020	1.55E-27	0.999	Lung	*HLA-DRB6*	5.67E-41	C	-	+
rs9273410	rs9273410	6	32627250	A	C	0.445	0.213	0.020	1.55E-27	0.999	GTEx_whole_blood	*HLA-DQB1-AS1*	2.09E-37	A	-	-
rs9273410	rs9273410	6	32627250	A	C	0.445	0.213	0.020	1.55E-27	0.999	Lung	*HLA-DQB2*	3.71E-30	C	+	-
rs9273410	rs9273410	6	32627250	A	C	0.445	0.213	0.020	1.55E-27	0.999	GTEx_whole_blood	*HLA-DRB6*	4.57E-25	A	+	+
rs9273410	rs9273410	6	32627250	A	C	0.445	0.213	0.020	1.55E-27	0.999	Lung	*HLA-DQB1*	2.09E-23	C	+	-
rs9273410	rs9273410	6	32627250	A	C	0.445	0.213	0.020	1.55E-27	0.999	Lung	*HLA-DQB1*	6.06E-23	C	+	-
rs9273410	rs9273410	6	32627250	A	C	0.445	0.213	0.020	1.55E-27	0.999	Lung	*AGPAT1*	6.39E-23	C	+	-
rs9273410	rs9273410	6	32627250	A	C	0.445	0.213	0.020	1.55E-27	0.999	Lung	*HLA-DQB1*	3.21E-22	C	+	-
rs9273410	rs9273410	6	32627250	A	C	0.445	0.213	0.020	1.55E-27	0.999	GTEx_lung	*HLA-DQB1-AS1*	5.69E-22	A	-	-
rs9273410	rs9273410	6	32627250	A	C	0.445	0.213	0.020	1.55E-27	0.999	GTEx_lung	*HLA-DRB6*	7.01E-22	A	+	+
rs9273410	rs9273410	6	32627250	A	C	0.445	0.213	0.020	1.55E-27	0.999	GTEx_lung	*HLA-DRB9*	2.71E-17	A	+	+
rs9273410	rs9273410	6	32627250	A	C	0.445	0.213	0.020	1.55E-27	0.999	GTEx_whole_blood	*HLA-DRB9*	5.47E-17	A	+	+
rs9273410	rs9273410	6	32627250	A	C	0.445	0.213	0.020	1.55E-27	0.999	Lung	*TAP2*	3.62E-13	C	+	-
rs9273410	rs9273410	6	32627250	A	C	0.445	0.213	0.020	1.55E-27	0.999	Lung	*AGPAT1*	1.27E-12	C	+	-
rs9273410	rs9273410	6	32627250	A	C	0.445	0.213	0.020	1.55E-27	0.999	GTEx_lung	*HLA-DRB1*	1.01E-11	A	-	-
rs9273410	rs9273410	6	32627250	A	C	0.445	0.213	0.020	1.55E-27	0.999	GTEx_lung	*HLA-DRB5*	7.48E-10	A	-	-
rs9273410	rs9273410	6	32627250	A	C	0.445	0.213	0.020	1.55E-27	0.999	Lung	*HLA-DQB2*	4.44E-09	C	+	-
rs9273410	rs9273410	6	32627250	A	C	0.445	0.213	0.020	1.55E-27	0.999	GTEx_whole_blood	*HLA-DRB5*	1.48E-08	A	-	-
rs9273410	rs9273410	6	32627250	A	C	0.445	0.213	0.020	1.55E-27	0.999	GTEx_lung	*C4A*	4.51E-06	A	-	-
rs9273410	rs9273410	6	32627250	A	C	0.445	0.213	0.020	1.55E-27	0.999	GTEx_whole_blood	*C4A*	1.28E-05	A	-	-
rs9273410	rs9273410	6	32627250	A	C	0.445	0.213	0.020	1.55E-27	0.999	Lung	*HLA-DMA*	2.09E-05	C	+	-
rs9273410	rs9273410	6	32627250	A	C	0.445	0.213	0.020	1.55E-27	0.999	GTEx_lung	*HLA-DOB*	2.56E-05	A	+	+
rs9273410	rs9273410	6	32627250	A	C	0.445	0.213	0.020	1.55E-27	0.999	Lung	*HLA-DPA1*	4.73E-05	C	+	-
rs9273410	rs9273410	6	32627250	A	C	0.445	0.213	0.020	1.55E-27	0.999	GTEx_whole_blood	*C4B*	5.81E-05	A	+	+
rs9273410	rs9273410	6	32627250	A	C	0.445	0.213	0.020	1.55E-27	0.999	GTEx_whole_blood	*CYP21A2*	1.20E-04	A	+	+

e-Table 10: Results of colocalisation analyses for rs80101740

coloc_snp_eqtl = beta and p-value for association of SNP with expression; coloc_snp_gwas = beta and p-value for association of SNP with ACO in stage 1 (UK Biobank); PP.HX.abf = posterior probability of hypothesis X (H0: neither trait [expression and ACO] has a genetic association in the region; H1: only trait 1 has a genetic association in the region; H2: only trait 2 has a genetic association in the region; H3: both traits associated but different causal variants; H4: both traits associated with a shared single causal variant)

Probe/coloc SNP	nsnps	Gene symbol	start:end	Chr:pos	coded/ non-coded	coloc_snp_eqtl	coloc_snp_gwas	PP.H0.abf	PP.H1.abf	PP.H2.abf	PP.H3.abf	PP.H4.abf
100123669_TGI_at	223		100239113:100241470	5:99392151	C/T	-0.098 (P=0.0056)	0.029 (P=0.31)	9.73E-01	1.62E-02	9.56E-03	1.58E-04	7.28E-04
100124460_TGI_at	24	*RIOK2*	96498640:96518944	5:97499614	C/T	-0.11 (P=0.14)	-0.14 (P=0.097)	9.97E-01	1.72E-03	9.67E-04	1.59E-06	7.87E-05
100126091_TGI_at	845	*FAM174A*	99871123:99922441	5:99427131	C/T	0.15 (P=0.001)	-0.04 (P=0.42)	8.87E-01	8.32E-02	2.44E-02	2.28E-03	3.44E-03
100129953_TGI_at	2572	*RGMB*	98104998:98129765	5:98471135	C/A	-0.014 (P=0.82)	0.37 (P=1.9e-06)	1.68E-01	6.20E-02	5.33E-01	1.97E-01	4.03E-02
100133549_TGI_at	2572		98128699:98129220	5:98471135	C/A	-0.015 (P=0.73)	0.37 (P=1.9e-06)	1.98E-01	3.21E-02	6.29E-01	1.02E-01	3.92E-02
100135322_TGI_at	534	*ST8SIA4*	100142903:100238970	5:99338518	A/C	0.13 (P=0.17)	-0.16 (P=0.053)	9.52E-01	2.82E-02	1.79E-02	5.30E-04	1.21E-03
100140190_TGI_at	9	*LIX1*	96430557:96478294	5:97477608	C/T	-0.072 (P=0.082)	0.057 (P=0.34)	9.99E-01	7.53E-04	2.64E-04	1.74E-07	2.50E-05
100140572_TGI_at	2544	*RGMB-AS1*	98105322:98108788	5:98108758	G/C	-0.066 (P=6.2e-10)	-0.066 (P=0.00042)	7.99E-06	1.42E-01	2.54E-05	4.50E-01	4.08E-01
100140772_TGI_at	2665		98852906:98853305	5:98471135	C/A	-0.031 (P=0.59)	0.37 (P=1.9e-06)	2.29E-01	3.92E-02	5.97E-01	1.02E-01	3.18E-02
100146169_TGI_at	525	*ST8SIA4*	100145359:100238987	5:99338518	A/C	0.14 (P=0.19)	-0.16 (P=0.053)	9.50E-01	3.01E-02	1.78E-02	5.62E-04	1.20E-03
100152307_TGI_at	10	*LIX1*	96427573:96478520	5:97471327	G/C	-0.18 (P=0.051)	0.058 (P=0.49)	9.99E-01	1.08E-03	2.77E-04	2.69E-07	2.96E-05
100154459_TGI_at	3003	*LOC100289230*	98264891:98266713	5:98312447	A/C	-0.12 (P=8.1e-05)	-0.079 (P=8.7e-05)	3.89E-02	8.53E-02	1.24E-01	2.72E-01	4.80E-01
100159234_TGI_at	2575	*RGMB*	98104999:98132196	5:98130968	A/G	0.66 (P=2.9e-170)	-0.048 (P=0.058)	3.84E-149	2.35E-01	1.22E-148	7.47E-01	1.73E-02
100160906_TGI_at	2443		98015482:98016692	5:98471135	C/A	0.044 (P=0.59)	0.37 (P=1.9e-06)	2.05E-01	2.63E-02	6.49E-01	8.34E-02	3.70E-02
100162289_TGI_at	2990	*CHD1*	98191448:98262240	5:98471135	C/A	-0.032 (P=0.68)	0.37 (P=1.9e-06)	1.85E-01	4.23E-02	5.91E-01	1.35E-01	4.67E-02
100301011_TGI_at	247	*ST8SIA4*	100221014:100238970	5:99338518	A/C	0.24 (P=0.022)	-0.16 (P=0.053)	9.69E-01	1.97E-02	1.03E-02	2.08E-04	1.10E-03
100301033_TGI_at	2547		98104999:98116889	5:98471135	C/A	0.013 (P=0.85)	0.37 (P=1.9e-06)	1.94E-01	3.73E-02	6.14E-01	1.18E-01	3.63E-02
100303038_TGI_at	2990	*CHD1*	98190908:98262238	5:98471135	C/A	0.021 (P=0.72)	0.37 (P=1.9e-06)	1.90E-01	3.74E-02	6.06E-01	1.19E-01	4.74E-02
100305010_TGI_at	523	*ST8SIA4*	100145679:100146257	5:99338518	A/C	0.13 (P=0.27)	-0.16 (P=0.053)	9.54E-01	2.65E-02	1.78E-02	4.93E-04	1.11E-03
100307991_TGI_at	845	*FAM174A*	99871050:99921974	5:99079537	C/T	0.031 (P=0.62)	0.14 (P=0.014)	9.29E-01	4.30E-02	2.55E-02	1.18E-03	1.67E-03
100312416_TGI_at	2865		98204849:98208136	5:98471135	C/A	0.034 (P=0.7)	0.37 (P=1.9e-06)	1.92E-01	3.75E-02	6.12E-01	1.19E-01	3.82E-02

e-Table 11: Results of PhenoScanner lookup for SNPs in 99% credible set at 1% FDR level

sentinel=sentinel variant in ACO GWAS; SNP=SNP in 99% credible set; chr=chromosome; pos=position (hg19); eff=effect allele; other=other allele; efo=EFO ontology term for trait or phenotype; FDR = false discovery rate (calculated by authors); pmid=PubMed ID (UKBB = Neale UK Biobank GWAS effort). For studies with binary outcomes, number of controls can be calculated as "n" – "n_cases". See also http://www.phenoscanner.medschl.cam.ac.uk/information/

sentinel	SNP	chr_bp_eff_other	trait	efo	pmid	ancestry	beta	se	p	n	n_cases	n_studies	unit	FDR
rs35570272	rs7631407	3:33036218_C_G	Neutrophil percentage of granulocytes	EFO_0004586	27863252	European	-0.027	0.004	1.34E-11	173480	0	2	IVNT	1.36E-08
rs35570272	rs7631407	3:33036218_C_G	Sum eosinophil basophil counts	EFO_0004586	27863252	European	0.025	0.004	1.11E-10	173480	0	2	IVNT	8.99E-08
rs35570272	rs7631407	3:33036218_C_G	Eosinophil percentage of granulocytes	EFO_0004586	27863252	European	0.024	0.004	4.26E-10	173480	0	2	IVNT	3.16E-07
rs35570272	rs7631407	3:33036218_C_G	Eosinophil percentage of white cells	EFO_0004586	27863252	European	0.024	0.004	4.52E-10	173480	0	2	IVNT	3.33E-07
rs35570272	rs7631407	3:33036218_C_G	Eosinophil count	EFO_0004586	27863252	European	0.023	0.004	2.41E-09	173480	0	2	IVNT	1.41E-06
rs35570272	rs7631407	3:33036218_C_G	No blood clot, bronchitis, emphysema, asthma, rhinitis, eczema or allergy diagnosed by doctor	-	UKBB	European	-0.006	0.001	3.32E-07	336782	228530	1	risk diff	1.23E-04
rs35570272	rs7631407	3:33036218_C_G	Allergic disease	EFO_0003785	29083406	European	0.029	0.006	3.29E-06	360838	180129	13	log OR	9.62E-04
rs35570272	rs7631407	3:33036218_C_G	Self-reported asthma	EFO_0000270	UKBB	European	0.004	0.001	4.96E-06	337159	39049	1	risk diff	1.39E-03
rs35570272	rs7631407	3:33036218_C_G	Asthma	EFO_0000270	UKBB	European	0.004	0.001	1.61E-05	336782	38791	1	risk diff	3.90E-03
rs35570272	rs7631407	3:33036218_C_G	Hayfever, allergic rhinitis or eczema	EFO_0003956; EFO_0005854; EFO_0000274	UKBB	European	0.005	0.001	1.84E-05	336782	77891	1	risk diff	4.40E-03
rs35570272	rs9828592	3:33044339_C_T	Self-reported asthma	EFO_0000270	UKBB	European	0.005	0.001	7.32E-11	337159	39049	1	risk diff	6.30E-08
rs35570272	rs9828592	3:33044339_C_T	No blood clot, bronchitis, emphysema, asthma, rhinitis, eczema or allergy diagnosed by doctor	-	UKBB	European	-0.007	0.001	1.94E-10	336782	228530	1	risk diff	1.52E-07
rs35570272	rs9828592	3:33044339_C_T	Asthma	EFO_0000270	UKBB	European	0.005	0.001	4.69E-10	336782	38791	1	risk diff	3.44E-07
rs35570272	rs9828592	3:33044339_C_T	Neutrophil percentage of granulocytes	EFO_0004586	27863252	European	-0.019	0.004	2.39E-07	173480	0	2	IVNT	9.12E-05
rs35570272	rs9828592	3:33044339_C_T	Sum eosinophil basophil counts	EFO_0004586	27863252	European	0.018	0.004	5.65E-07	173480	0	2	IVNT	2.00E-04
rs35570272	rs9828592	3:33044339_C_T	Allergic disease	EFO_0003785	29083406	European	0.028	0.006	1.05E-06	360838	180129	13	log OR	3.43E-04
rs35570272	rs9828592	3:33044339_C_T	Eosinophil percentage of granulocytes	EFO_0004586	27863252	European	0.017	0.004	1.50E-06	173480	0	2	IVNT	4.70E-04
rs35570272	rs9828592	3:33044339_C_T	Hayfever, allergic rhinitis or eczema	EFO_0003956; EFO_0005854; EFO_0000274	UKBB	European	0.005	0.001	1.93E-06	336782	77891	1	risk diff	5.96E-04
rs35570272	rs9828592	3:33044339_C_T	Eosinophil percentage of white cells	EFO_0004586	27863252	European	0.017	0.004	3.59E-06	173480	0	2	IVNT	1.04E-03
rs35570272	rs9828592	3:33044339_C_T	Eosinophil count	EFO_0004586	27863252	European	0.017	0.004	4.05E-06	173480	0	2	IVNT	1.16E-03
rs35570272	rs9828592	3:33044339_C_T	Wheeze or whistling in the chest in last year	EFO_0009715	UKBB	European	0.004	0.001	2.03E-05	331257	68531	1	risk diff	4.81E-03
rs35570272	rs9828592	3:33044339_C_T	Neuroticism	EFO_0007660; EFO_0004257	29292387	European	-0.010	0.003	3.94E-05	168105	0	2	SD	8.48E-03
rs35570272	rs7646283	3:33046480_T_C	Neutrophil percentage of granulocytes	EFO_0004586	27863252	European	0.030	0.004	3.87E-15	173480	0	2	IVNT	1.01E-11
rs35570272	rs7646283	3:33046480_T_C	Sum eosinophil basophil counts	EFO_0004586	27863252	European	-0.029	0.004	2.96E-14	173480	0	2	IVNT	6.18E-11
rs35570272	rs7646283	3:33046480_T_C	Eosinophil percentage of white cells	EFO_0004586	27863252	European	-0.028	0.004	7.59E-14	173480	0	2	IVNT	1.42E-10
rs35570272	rs7646283	3:33046480_T_C	Eosinophil percentage of granulocytes	EFO_0004586	27863252	European	-0.028	0.004	8.39E-14	173480	0	2	IVNT	1.53E-10
rs35570272	rs7646283	3:33046480_T_C	Eosinophil count	EFO_0004586	27863252	European	-0.027	0.004	4.34E-13	173480	0	2	IVNT	6.49E-10
rs35570272	rs7646283	3:33046480_T_C	No blood clot, bronchitis, emphysema, asthma, rhinitis, eczema or allergy diagnosed by doctor	-	UKBB	European	0.009	0.001	7.78E-13	336782	228530	1	risk diff	1.09E-09
rs35570272	rs7646283	3:33046480_T_C	Self-reported asthma	EFO_0000270	UKBB	European	-0.005	0.001	4.41E-11	337159	39049	1	risk diff	3.99E-08
rs35570272	rs7646283	3:33046480_T_C	Asthma	EFO_0000270	UKBB	European	-0.005	0.001	1.13E-10	336782	38791	1	risk diff	9.15E-08

sentinel	SNP	chr_bp_eff_other	trait	efo	pmid	ancestry	beta	se	p	n	n_cases	n_studies	unit	FDR
rs35570272	rs7646283	3:33046480_T_C	Hayfever, allergic rhinitis or eczema	EFO_0003956; EFO_0005854; EFO_0000274	UKBB	European	-0.006	0.001	2.32E-08	336782	77891	1	risk diff	1.11E-05
rs35570272	rs7646283	3:33046480_T_C	Allergic disease	EFO_0003785	29083406	European	-0.032	0.006	6.13E-08	360838	180129	13	log OR	2.67E-05
rs35570272	rs34064757	3:33047283_A_G	Neutrophil percentage of granulocytes	EFO_0004586	27863252	European	-0.030	0.004	2.72E-15	173480	0	2	IVNT	7.71E-12
rs35570272	rs34064757	3:33047283_A_G	Neutrophil percentage of granulocytes	EFO_0007994	27863252	European	0.030	0.004	3.00E-15	-	-	-	unit decrease	8.21E-12
rs35570272	rs34064757	3:33047283_A_G	Sum eosinophil basophil counts	EFO_0004586	27863252	European	0.029	0.004	1.94E-14	173480	0	2	IVNT	4.24E-11
rs35570272	rs34064757	3:33047283_A_G	Sum eosinophil basophil counts	EFO_0005090; EFO_0004842	27863252	European	0.029	0.004	2.00E-14	-	-	-	unit increase	4.33E-11
rs35570272	rs34064757	3:33047283_A_G	Eosinophil percentage of granulocytes	EFO_0007996	27863252	European	0.028	0.004	6.00E-14	-	-	-	unit increase	1.17E-10
rs35570272	rs34064757	3:33047283_A_G	Eosinophil percentage of white cells	EFO_0007991	27863252	European	0.028	0.004	6.00E-14	-	-	-	unit increase	1.17E-10
rs35570272	rs34064757	3:33047283_A_G	Eosinophil percentage of white cells	EFO_0004586	27863252	European	0.028	0.004	6.32E-14	173480	0	2	IVNT	1.22E-10
rs35570272	rs34064757	3:33047283_A_G	Eosinophil percentage of granulocytes	EFO_0004586	27863252	European	0.028	0.004	6.38E-14	173480	0	2	IVNT	1.23E-10
rs35570272	rs34064757	3:33047283_A_G	Eosinophil counts	EFO_0004842	27863252	European	0.027	0.004	3.00E-13	-	-	-	unit increase	4.80E-10
rs35570272	rs34064757	3:33047283_A_G	Eosinophil count	EFO_0004586	27863252	European	0.027	0.004	3.33E-13	173480	0	2	IVNT	5.19E-10
rs35570272	rs34064757	3:33047283_A_G	No blood clot, bronchitis, emphysema, asthma, rhinitis, eczema or allergy diagnosed by doctor	-	UKBB	European	-0.008	0.001	9.06E-13	336782	228530	1	risk diff	1.25E-09
rs35570272	rs34064757	3:33047283_A_G	Self-reported asthma	EFO_0000270	UKBB	European	0.005	0.001	4.22E-11	337159	39049	1	risk diff	3.85E-08
rs35570272	rs34064757	3:33047283_A_G	Asthma	EFO_0000270	UKBB	European	0.005	0.001	1.09E-10	336782	38791	1	risk diff	8.91E-08
rs35570272	rs34064757	3:33047283_A_G	Hayfever, allergic rhinitis or eczema	EFO_0003956; EFO_0005854; EFO_0000274	UKBB	European	0.006	0.001	2.42E-08	336782	77891	1	risk diff	1.15E-05
rs35570272	rs34064757	3:33047283_A_G	Allergic disease	EFO_0003785	29083406	European	0.032	0.006	6.92E-08	360838	180129	13	log OR	2.98E-05
rs35570272	rs35570272	3:33047662_T_G	Self-reported asthma	EFO_0000270	UKBB	European	-0.006	0.001	7.45E-13	337159	39049	1	risk diff	1.06E-09
rs35570272	rs35570272	3:33047662_T_G	No blood clot, bronchitis, emphysema, asthma, rhinitis, eczema or allergy diagnosed by doctor	-	UKBB	European	0.008	0.001	1.01E-12	336782	228530	1	risk diff	1.37E-09
rs35570272	rs35570272	3:33047662_T_G	Neutrophil percentage of granulocytes	EFO_0004586	27863252	European	0.026	0.004	1.89E-12	173480	0	2	IVNT	2.26E-09
rs35570272	rs35570272	3:33047662_T_G	Sum eosinophil basophil counts	EFO_0004586	27863252	European	-0.026	0.004	2.56E-12	173480	0	2	IVNT	2.98E-09
rs35570272	rs35570272	3:33047662_T_G	Asthma	EFO_0000270	UKBB	European	-0.006	0.001	2.83E-12	336782	38791	1	risk diff	3.27E-09
rs35570272	rs35570272	3:33047662_T_G	Eosinophil percentage of granulocytes	EFO_0004586	27863252	European	-0.024	0.004	4.92E-11	173480	0	2	IVNT	4.41E-08
rs35570272	rs35570272	3:33047662_T_G	Eosinophil count	EFO_0004586	27863252	European	-0.024	0.004	5.13E-11	173480	0	2	IVNT	4.59E-08
rs35570272	rs35570272	3:33047662_T_G	Eosinophil percentage of white cells	EFO_0004586	27863252	European	-0.024	0.004	7.39E-11	173480	0	2	IVNT	6.31E-08
rs35570272	rs35570272	3:33047662_T_G	Allergic disease	EFO_0003785	29083406	European	-0.031	0.006	1.83E-07	360838	180129	13	log OR	7.09E-05
rs35570272	rs35570272	3:33047662_T_G	Hayfever, allergic rhinitis or eczema	EFO_0003956; EFO_0005854; EFO_0000274	UKBB	European	-0.005	0.001	2.25E-07	336782	77891	1	risk diff	8.60E-05
rs35570272	rs35570272	3:33047662_T_G	Wheeze or whistling in the chest in last year	EFO_0009715	UKBB	European	-0.005	0.001	8.76E-06	331257	68531	1	risk diff	2.31E-03
rs35570272	rs73057087	3:33068268_G_A	No blood clot, bronchitis, emphysema, asthma, rhinitis, eczema or allergy diagnosed by doctor	-	UKBB	European	0.009	0.001	2.42E-15	336782	228530	1	risk diff	6.99E-12
rs35570272	rs73057087	3:33068268_G_A	Hayfever, allergic rhinitis or eczema	EFO_0003956; EFO_0005854; EFO_0000274	UKBB	European	-0.007	0.001	4.02E-11	336782	77891	1	risk diff	3.69E-08
rs35570272	rs73057087	3:33068268_G_A	Sum eosinophil basophil counts	EFO_0004586	27863252	European	-0.023	0.004	1.22E-09	173480	0	2	IVNT	7.84E-07
rs35570272	rs73057087	3:33068268_G_A	Neutrophil percentage of granulocytes	EFO_0004586	27863252	European	0.022	0.004	2.76E-09	173480	0	2	IVNT	1.60E-06
rs35570272	rs73057087	3:33068268_G_A	Eosinophil count	EFO_0004586	27863252	European	-0.022	0.004	2.87E-09	173480	0	2	IVNT	1.66E-06

sentinel	SNP	chr_bp_eff_other	trait	efo	pmid	ancestry	beta	se	p	n	n_cases	n_studies	unit	FDR
rs35570272	rs73057087	3:33068268_G_A	Eosinophil percentage of white cells	EFO_0004586	27863252	European	-0.022	0.004	4.84E-09	173480	0	2	IVNT	2.58E-06
rs35570272	rs73057087	3:33068268_G_A	Eosinophil percentage of granulocytes	EFO_0004586	27863252	European	-0.021	0.004	7.93E-09	173480	0	2	IVNT	4.07E-06
rs35570272	rs73057087	3:33068268_G_A	Allergic disease	EFO_0003785	29083406	European	-0.032	0.006	4.51E-08	360838	180129	13	log OR	2.02E-05
rs35570272	rs73057087	3:33068268_G_A	Self-reported asthma	EFO_0000270	UKBB	European	-0.004	0.001	2.66E-07	337159	39049	1	risk diff	1.01E-04
rs35570272	rs73057087	3:33068268_G_A	Asthma	EFO_0000270	UKBB	European	-0.004	0.001	5.43E-07	336782	38791	1	risk diff	1.93E-04
rs35570272	rs4547648	3:33080256_G_A	No blood clot, bronchitis, emphysema, asthma, rhinitis, eczema or allergy diagnosed by doctor	-	UKBB	European	0.009	0.001	5.23E-15	336782	228530	1	risk diff	1.32E-11
rs35570272	rs4547648	3:33080256_G_A	Hayfever, allergic rhinitis or eczema	EFO_0003956; EFO_0005854; EFO_0000274	UKBB	European	-0.007	0.001	7.29E-11	336782	77891	1	risk diff	6.29E-08
rs35570272	rs4547648	3:33080256_G_A	Sum eosinophil basophil counts	EFO_0004586	27863252	European	-0.023	0.004	1.12E-09	173480	0	2	IVNT	7.23E-07
rs35570272	rs4547648	3:33080256_G_A	Neutrophil percentage of granulocytes	EFO_0004586	27863252	European	0.022	0.004	3.38E-09	173480	0	2	IVNT	1.93E-06
rs35570272	rs4547648	3:33080256_G_A	Eosinophil count	EFO_0004586	27863252	European	-0.022	0.004	3.48E-09	173480	0	2	IVNT	1.97E-06
rs35570272	rs4547648	3:33080256_G_A	Eosinophil percentage of white cells	EFO_0004586	27863252	European	-0.021	0.004	6.73E-09	173480	0	2	IVNT	3.50E-06
rs35570272	rs4547648	3:33080256_G_A	Eosinophil percentage of granulocytes	EFO_0004586	27863252	European	-0.021	0.004	1.32E-08	173480	0	2	IVNT	6.55E-06
rs35570272	rs4547648	3:33080256_G_A	Allergic disease	EFO_0003785	29083406	European	-0.033	0.006	3.06E-08	360838	180129	13	log OR	1.43E-05
rs35570272	rs4547648	3:33080256_G_A	Self-reported asthma	EFO_0000270	UKBB	European	-0.004	0.001	3.24E-07	337159	39049	1	risk diff	1.21E-04
rs35570272	rs4547648	3:33080256_G_A	Asthma	EFO_0000270	UKBB	European	-0.004	0.001	6.74E-07	336782	38791	1	risk diff	2.32E-04
rs35570272	rs4547648	3:33080256_G_A	Wheeze or whistling in the chest in last year	EFO_0009715	UKBB	European	-0.004	0.001	3.68E-05	331257	68531	1	risk diff	7.98E-03
rs35570272	rs4277638	3:33080356_A_G	No blood clot, bronchitis, emphysema, asthma, rhinitis, eczema or allergy diagnosed by doctor	-	UKBB	European	-0.009	0.001	4.02E-15	336782	228530	1	risk diff	1.02E-11
rs35570272	rs4277638	3:33080356_A_G	Hayfever, allergic rhinitis or eczema	EFO_0003956; EFO_0005854; EFO_0000274	UKBB	European	0.007	0.001	6.07E-11	336782	77891	1	risk diff	5.27E-08
rs35570272	rs4277638	3:33080356_A_G	Sum eosinophil basophil counts	EFO_0004586	27863252	European	0.023	0.004	1.06E-09	173480	0	2	IVNT	6.93E-07
rs35570272	rs4277638	3:33080356_A_G	Neutrophil percentage of granulocytes	EFO_0004586	27863252	European	-0.022	0.004	3.01E-09	173480	0	2	IVNT	1.74E-06
rs35570272	rs4277638	3:33080356_A_G	Eosinophil count	EFO_0004586	27863252	European	0.022	0.004	3.33E-09	173480	0	2	IVNT	1.91E-06
rs35570272	rs4277638	3:33080356_A_G	Eosinophil percentage of white cells	EFO_0004586	27863252	European	0.021	0.004	6.23E-09	173480	0	2	IVNT	3.28E-06
rs35570272	rs4277638	3:33080356_A_G	Eosinophil percentage of granulocytes	EFO_0004586	27863252	European	0.021	0.004	1.17E-08	173480	0	2	IVNT	5.90E-06
rs35570272	rs4277638	3:33080356_A_G	Allergic disease	EFO_0003785	29083406	European	0.033	0.006	3.22E-08	360838	180129	13	log OR	1.50E-05
rs35570272	rs4277638	3:33080356_A_G	Self-reported asthma	EFO_0000270	UKBB	European	0.004	0.001	2.93E-07	337159	39049	1	risk diff	1.10E-04
rs35570272	rs4277638	3:33080356_A_G	Asthma	EFO_0000270	UKBB	European	0.004	0.001	6.12E-07	336782	38791	1	risk diff	2.14E-04
rs35570272	rs4277638	3:33080356_A_G	Wheeze or whistling in the chest in last year	EFO_0009715	UKBB	European	0.004	0.001	3.39E-05	331257	68531	1	risk diff	7.45E-03
rs35570272	rs4274702	3:33080722_T_C	No blood clot, bronchitis, emphysema, asthma, rhinitis, eczema or allergy diagnosed by doctor	-	UKBB	European	0.009	0.001	3.85E-15	336782	228530	1	risk diff	1.01E-11
rs35570272	rs4274702	3:33080722_T_C	Hayfever, allergic rhinitis or eczema	EFO_0003956; EFO_0005854; EFO_0000274	UKBB	European	-0.007	0.001	5.56E-11	336782	77891	1	risk diff	4.92E-08
rs35570272	rs4274702	3:33080722_T_C	Sum eosinophil basophil counts	EFO_0004586	27863252	European	-0.022	0.004	1.43E-09	173480	0	2	IVNT	8.83E-07
rs35570272	rs4274702	3:33080722_T_C	Neutrophil percentage of granulocytes	EFO_0004586	27863252	European	0.022	0.004	3.89E-09	173480	0	2	IVNT	2.14E-06
rs35570272	rs4274702	3:33080722_T_C	Eosinophil count	EFO_0004586	27863252	European	-0.022	0.004	4.45E-09	173480	0	2	IVNT	2.41E-06
rs35570272	rs4274702	3:33080722_T_C	Eosinophil percentage of white cells	EFO_0004586	27863252	European	-0.021	0.004	7.99E-09	173480	0	2	IVNT	4.10E-06
rs35570272	rs4274702	3:33080722_T_C	Eosinophil percentage of granulocytes	EFO_0004586	27863252	European	-0.021	0.004	1.52E-08	173480	0	2	IVNT	7.47E-06
rs35570272	rs4274702	3:33080722_T_C	Allergic disease	EFO_0003785	29083406	European	-0.033	0.006	2.41E-08	360838	180129	13	log OR	1.15E-05
rs35570272	rs4274702	3:33080722_T_C	Self-reported asthma	EFO_0000270	UKBB	European	-0.004	0.001	3.09E-07	337159	39049	1	risk diff	1.16E-04
rs35570272	rs4274702	3:33080722_T_C	Asthma	EFO_0000270	UKBB	European	-0.004	0.001	6.46E-07	336782	38791	1	risk diff	2.25E-04
rs35570272	rs4274702	3:33080722_T_C	Wheeze or whistling in the chest in last year	EFO_0009715	UKBB	European	-0.004	0.001	3.46E-05	331257	68531	1	risk diff	7.57E-03
rs35570272	rs6800001	3:33081102_A_G	No blood clot, bronchitis, emphysema, asthma, rhinitis, eczema or allergy diagnosed by doctor	-	UKBB	European	-0.009	0.001	3.88E-15	336782	228530	1	risk diff	1.01E-11

sentinel	SNP	chr_bp_eff_other	trait	efo	pmid	ancestry	beta	se	p	n	n_cases	n_studies	unit	FDR
rs35570272	rs6800001	3:33081102_A_G	Hayfever, allergic rhinitis or eczema	EFO_0003956; EFO_0005854; EFO_0000274	UKBB	European	0.007	0.001	5.52E-11	336782	77891	1	risk diff	4.90E-08
rs35570272	rs6800001	3:33081102_A_G	Sum eosinophil basophil counts	EFO_0004586	27863252	European	0.022	0.004	1.33E-09	173480	0	2	IVNT	8.36E-07
rs35570272	rs6800001	3:33081102_A_G	Eosinophil count	EFO_0004586	27863252	European	0.022	0.004	3.12E-09	173480	0	2	IVNT	1.80E-06
rs35570272	rs6800001	3:33081102_A_G	Neutrophil percentage of granulocytes	EFO_0004586	27863252	European	-0.022	0.004	3.65E-09	173480	0	2	IVNT	2.04E-06
rs35570272	rs6800001	3:33081102_A_G	Eosinophil percentage of white cells	EFO_0004586	27863252	European	0.022	0.004	5.76E-09	173480	0	2	IVNT	3.04E-06
rs35570272	rs6800001	3:33081102_A_G	Eosinophil percentage of granulocytes	EFO_0004586	27863252	European	0.021	0.004	1.09E-08	173480	0	2	IVNT	5.49E-06
rs35570272	rs6800001	3:33081102_A_G	Allergic disease	EFO_0003785	29083406	European	0.033	0.006	2.28E-08	360838	180129	13	log OR	1.09E-05
rs35570272	rs6800001	3:33081102_A_G	Self-reported asthma	EFO_0000270	UKBB	European	0.004	0.001	3.24E-07	337159	39049	1	risk diff	1.21E-04
rs35570272	rs6800001	3:33081102_A_G	Asthma	EFO_0000270	UKBB	European	0.004	0.001	6.51E-07	336782	38791	1	risk diff	2.26E-04
rs35570272	rs6800001	3:33081102_A_G	Wheeze or whistling in the chest in last year	EFO_0009715	UKBB	European	0.004	0.001	3.40E-05	331257	68531	1	risk diff	7.45E-03
rs35570272	rs28752078	3:33084861_T_C	No blood clot, bronchitis, emphysema, asthma, rhinitis, eczema or allergy diagnosed by doctor	-	UKBB	European	0.009	0.001	1.07E-14	336782	228530	1	risk diff	2.43E-11
rs35570272	rs28752078	3:33084861_T_C	Hayfever, allergic rhinitis or eczema	EFO_0003956; EFO_0005854; EFO_0000274	UKBB	European	-0.006	0.001	6.97E-10	336782	77891	1	risk diff	4.92E-07
rs35570272	rs28752078	3:33084861_T_C	Self-reported asthma	EFO_0000270	UKBB	European	-0.004	0.001	2.66E-08	337159	39049	1	risk diff	1.25E-05
rs35570272	rs28752078	3:33084861_T_C	Sum eosinophil basophil counts	EFO_0004586	27863252	European	-0.020	0.004	8.56E-08	173480	0	2	IVNT	3.55E-05
rs35570272	rs28752078	3:33084861_T_C	Asthma	EFO_0000270	UKBB	European	-0.004	0.001	8.75E-08	336782	38791	1	risk diff	3.62E-05
rs35570272	rs28752078	3:33084861_T_C	Allergic disease	EFO_0003785	29083406	European	-0.031	0.006	1.24E-07	360838	180129	13	log OR	4.96E-05
rs35570272	rs28752078	3:33084861_T_C	Eosinophil count	EFO_0004586	27863252	European	-0.019	0.004	2.37E-07	173480	0	2	IVNT	9.06E-05
rs35570272	rs28752078	3:33084861_T_C	Neutrophil percentage of granulocytes	EFO_0004586	27863252	European	0.018	0.004	4.60E-07	173480	0	2	IVNT	1.66E-04
rs35570272	rs28752078	3:33084861_T_C	Eosinophil percentage of white cells	EFO_0004586	27863252	European	-0.018	0.004	1.16E-06	173480	0	2	IVNT	3.73E-04
rs35570272	rs28752078	3:33084861_T_C	Eosinophil percentage of granulocytes	EFO_0004586	27863252	European	-0.018	0.004	1.54E-06	173480	0	2	IVNT	4.80E-04
rs35570272	rs28752078	3:33084861_T_C	Wheeze or whistling in the chest in last year	EFO_0009715	UKBB	European	-0.005	0.001	7.18E-06	331257	68531	1	risk diff	1.95E-03
rs6787279	rs74932439	3:57115567_T_C	Forced expiratory volume in 1-second	EFO_0004314	UKBB	European	-0.014	0.003	5.81E-07	307638	0	1	IVNT	2.05E-04
rs6787279	rs74932439	3:57115567_T_C	Average weekly beer plus cider intake	EFO_0004329	UKBB	European	0.012	0.003	1.37E-05	241447	0	1	-	3.38E-03
rs6787279	rs74932439	3:57115567_T_C	Hand grip strength right	EFO_0006941	UKBB	European	-0.010	0.002	1.52E-05	335842	0	1	IVNT	3.69E-03
rs6787279	rs74999629	3:57117542_A_G	Forced expiratory volume in 1-second	EFO_0004314	UKBB	European	0.014	0.003	6.18E-07	307638	0	1	IVNT	2.16E-04
rs6787279	rs74999629	3:57117542_A_G	Average weekly beer plus cider intake	EFO_0004329	UKBB	European	-0.012	0.003	1.34E-05	241447	0	1	-	3.32E-03
rs6787279	rs74999629	3:57117542_A_G	Hand grip strength right	EFO_0006941	UKBB	European	0.010	0.002	1.42E-05	335842	0	1	IVNT	3.48E-03
rs6787279	rs59527464	3:57122780_T_C	Forced expiratory volume in 1-second	EFO_0004314	UKBB	European	-0.012	0.003	6.11E-06	307638	0	1	IVNT	1.68E-03
rs6787279	rs2035656	3:57125101_C_A	Forced expiratory volume in 1-second	EFO_0004314	UKBB	European	0.014	0.003	6.53E-07	307638	0	1	IVNT	2.26E-04
rs6787279	rs2035656	3:57125101_C_A	Hand grip strength right	EFO_0006941	UKBB	European	0.010	0.002	1.49E-05	335842	0	1	IVNT	3.63E-03
rs6787279	rs2035656	3:57125101_C_A	Average weekly beer plus cider intake	EFO_0004329	UKBB	European	-0.012	0.003	1.55E-05	241447	0	1	-	3.76E-03
rs6787279	rs2035655	3:57125202_G_T	Forced expiratory volume in 1-second	EFO_0004314	UKBB	European	-0.012	0.003	5.99E-06	307638	0	1	IVNT	1.66E-03
rs6787279	rs17289035	3:57125424_A_G	Forced expiratory volume in 1-second	EFO_0004314	UKBB	European	0.012	0.003	6.39E-06	307638	0	1	IVNT	1.76E-03
rs6787279	rs12486855	3:57125864_G_A	Forced expiratory volume in 1-second	EFO_0004314	UKBB	European	-0.014	0.003	8.23E-07	307638	0	1	IVNT	2.79E-04
rs6787279	rs12486855	3:57125864_G_A	Average weekly beer plus cider intake	EFO_0004329	UKBB	European	0.012	0.003	1.30E-05	241447	0	1	-	3.24E-03
rs6787279	rs12486855	3:57125864_G_A	Hand grip strength right	EFO_0006941	UKBB	European	-0.010	0.002	1.41E-05	335842	0	1	IVNT	3.46E-03
rs6787279	rs17057718	3:57136585_T_C	Cerebrospinal fluid biomarker levels	EFO_0006794	28031287	East Asian	NA	NA	1.00E-11	-	-	-	-	1.05E-08
rs6787279	rs17057718	3:57136585_T_C	Forced expiratory volume in 1-second	EFO_0004314	UKBB	European	-0.014	0.003	1.14E-06	307638	0	1	IVNT	3.66E-04
rs6787279	rs17057718	3:57136585_T_C	Hand grip strength right	EFO_0006941	UKBB	European	-0.011	0.002	1.20E-05	335842	0	1	IVNT	3.00E-03
rs6787279	rs17057718	3:57136585_T_C	Average weekly beer plus cider intake	EFO_0004329	UKBB	European	0.012	0.003	2.10E-05	241447	0	1	-	4.94E-03
rs6787279	rs1075398	3:57137596_C_A	Forced expiratory volume in 1-second	EFO_0004314	UKBB	European	-0.014	0.003	8.64E-07	307638	0	1	IVNT	2.91E-04
rs6787279	rs1075398	3:57137596_C_A	Hand grip strength right	EFO_0006941	UKBB	European	-0.011	0.002	1.05E-05	335842	0	1	IVNT	2.68E-03
rs6787279	rs1075398	3:57137596_C_A	Average weekly beer plus cider intake	EFO_0004329	UKBB	European	0.012	0.003	2.08E-05	241447	0	1	-	4.91E-03
rs6787279	rs1077034	3:57138228_A_T	Forced expiratory volume in 1-second	EFO_0004314	UKBB	European	-0.014	0.003	8.57E-07	307638	0	1	IVNT	2.89E-04

sentinel	SNP	chr_bp_eff_other	trait	efo	pmid	ancestry	beta	se	p	n	n_cases	n_studies	unit	FDR
rs6787279	rs1077034	3:57138228_A_T	Hand grip strength right	EFO_0006941	UKBB	European	-0.011	0.002	1.03E-05	335842	0	1	IVNT	2.64E-03
rs6787279	rs1077034	3:57138228_A_T	Average weekly beer plus cider intake	EFO_0004329	UKBB	European	0.012	0.003	1.93E-05	241447	0	1	-	4.59E-03
rs6787279	rs76645245	3:57140794_G_A	Forced expiratory volume in 1-second	EFO_0004314	UKBB	European	-0.014	0.003	8.54E-07	307638	0	1	IVNT	2.89E-04
rs6787279	rs76645245	3:57140794_G_A	Hand grip strength right	EFO_0006941	UKBB	European	-0.011	0.002	1.17E-05	335842	0	1	IVNT	2.95E-03
rs6787279	rs76645245	3:57140794_G_A	Average weekly beer plus cider intake	EFO_0004329	UKBB	European	0.012	0.003	2.15E-05	241447	0	1	-	5.06E-03
rs6787279	rs78345417	3:57143942_T_C	Forced expiratory volume in 1-second	EFO_0004314	UKBB	European	-0.014	0.003	9.18E-07	307638	0	1	IVNT	3.05E-04
rs6787279	rs78345417	3:57143942_T_C	Hand grip strength right	EFO_0006941	UKBB	European	-0.011	0.002	1.15E-05	335842	0	1	IVNT	2.91E-03
rs6787279	rs78345417	3:57143942_T_C	Average weekly beer plus cider intake	EFO_0004329	UKBB	European	0.012	0.003	2.32E-05	241447	0	1	-	5.41E-03
rs6787279	rs79932559	3:57145726_T_C	Forced expiratory volume in 1-second	EFO_0004314	UKBB	European	-0.014	0.003	9.15E-07	307638	0	1	IVNT	3.05E-04
rs6787279	rs79932559	3:57145726_T_C	Hand grip strength right	EFO_0006941	UKBB	European	-0.011	0.002	1.19E-05	335842	0	1	IVNT	2.99E-03
rs6787279	rs79932559	3:57145726_T_C	Average weekly beer plus cider intake	EFO_0004329	UKBB	European	0.012	0.003	2.24E-05	241447	0	1	-	5.24E-03
rs6787279	rs77543785	3:57148271_T_C	Forced expiratory volume in 1-second	EFO_0004314	UKBB	European	-0.014	0.003	9.57E-07	307638	0	1	IVNT	3.15E-04
rs6787279	rs77543785	3:57148271_T_C	Hand grip strength right	EFO_0006941	UKBB	European	-0.010	0.002	1.32E-05	335842	0	1	IVNT	3.28E-03
rs6787279	rs77543785	3:57148271_T_C	Average weekly beer plus cider intake	EFO_0004329	UKBB	European	0.012	0.003	2.15E-05	241447	0	1	-	5.06E-03
rs6787279	rs6792236	3:57149315_G_T	Forced expiratory volume in 1-second	EFO_0004314	UKBB	European	0.014	0.003	9.57E-07	307638	0	1	IVNT	3.15E-04
rs6787279	rs6792236	3:57149315_G_T	Hand grip strength right	EFO_0006941	UKBB	European	0.010	0.002	1.26E-05	335842	0	1	IVNT	3.16E-03
rs6787279	rs6792236	3:57149315_G_T	Average weekly beer plus cider intake	EFO_0004329	UKBB	European	-0.012	0.003	2.04E-05	241447	0	1	-	4.82E-03
rs6787279	rs80095764	3:57149793_T_C	Forced expiratory volume in 1-second	EFO_0004314	UKBB	European	0.014	0.003	9.31E-07	307638	0	1	IVNT	3.09E-04
rs6787279	rs80095764	3:57149793_T_C	Hand grip strength right	EFO_0006941	UKBB	European	0.010	0.002	1.40E-05	335842	0	1	IVNT	3.43E-03
rs6787279	rs80095764	3:57149793_T_C	Average weekly beer plus cider intake	EFO_0004329	UKBB	European	-0.012	0.003	2.21E-05	241447	0	1	-	5.19E-03
rs6787279	rs1488123	3:57151812_A_G	Forced expiratory volume in 1-second	EFO_0004314	UKBB	European	0.014	0.003	9.04E-07	307638	0	1	IVNT	3.03E-04
rs6787279	rs1488123	3:57151812_A_G	Hand grip strength right	EFO_0006941	UKBB	European	0.011	0.002	1.18E-05	335842	0	1	IVNT	2.97E-03
rs6787279	rs1488123	3:57151812_A_G	Average weekly beer plus cider intake	EFO_0004329	UKBB	European	-0.012	0.003	2.31E-05	241447	0	1	-	5.39E-03
rs6787279	rs1386832	3:57152006_G_A	Forced expiratory volume in 1-second	EFO_0004314	UKBB	European	-0.014	0.003	9.09E-07	307638	0	1	IVNT	3.04E-04
rs6787279	rs1386832	3:57152006_G_A	Hand grip strength right	EFO_0006941	UKBB	European	-0.011	0.002	1.16E-05	335842	0	1	IVNT	2.94E-03
rs6787279	rs1386832	3:57152006_G_A	Average weekly beer plus cider intake	EFO_0004329	UKBB	European	0.012	0.003	2.32E-05	241447	0	1	-	5.40E-03
rs6787279	rs1386833	3:57152041_T_C	Forced expiratory volume in 1-second	EFO_0004314	UKBB	European	-0.013	0.003	3.87E-06	307638	0	1	IVNT	1.11E-03
rs6787279	rs1386833	3:57152041_T_C	Hand grip strength right	EFO_0006941	UKBB	European	-0.011	0.002	1.09E-05	335842	0	1	IVNT	2.76E-03
rs6787279	rs1386833	3:57152041_T_C	Average weekly beer plus cider intake	EFO_0004329	UKBB	European	0.012	0.003	2.40E-05	241447	0	1	-	5.56E-03
rs6787279	rs747089	3:57153869_T_C	Forced expiratory volume in 1-second	EFO_0004314	UKBB	European	-0.014	0.003	1.03E-06	307638	0	1	IVNT	3.37E-04
rs6787279	rs747089	3:57153869_T_C	Hand grip strength right	EFO_0006941	UKBB	European	-0.011	0.002	1.04E-05	335842	0	1	IVNT	2.67E-03
rs6787279	rs747089	3:57153869_T_C	Average weekly beer plus cider intake	EFO_0004329	UKBB	European	0.011	0.003	2.77E-05	241447	0	1	-	6.31E-03
rs6787279	rs1872942	3:57154878_T_C	Forced expiratory volume in 1-second	EFO_0004314	UKBB	European	0.014	0.003	1.13E-06	307638	0	1	IVNT	3.65E-04
rs6787279	rs1872942	3:57154878_T_C	Hand grip strength right	EFO_0006941	UKBB	European	0.011	0.002	9.17E-06	335842	0	1	IVNT	2.40E-03
rs6787279	rs1872942	3:57154878_T_C	Average weekly beer plus cider intake	EFO_0004329	UKBB	European	-0.011	0.003	2.87E-05	241447	0	1	-	6.49E-03
rs6787279	rs111888204	3:57157978_T_C	Forced expiratory volume in 1-second	EFO_0004314	UKBB	European	-0.014	0.003	1.45E-06	307638	0	1	IVNT	4.55E-04
rs6787279	rs111888204	3:57157978_T_C	Hand grip strength right	EFO_0006941	UKBB	European	-0.011	0.002	1.22E-05	335842	0	1	IVNT	3.05E-03
rs6787279	rs111888204	3:57157978_T_C	Average weekly beer plus cider intake	EFO_0004329	UKBB	European	0.011	0.003	3.06E-05	241447	0	1	-	6.82E-03
rs6787279	rs6768368	3:57162476_T_C	Forced expiratory volume in 1-second	EFO_0004314	UKBB	European	-0.018	0.003	1.36E-09	307638	0	1	IVNT	8.46E-07
rs6787279	rs6768368	3:57162476_T_C	Forced expiratory volume in 1-second, best measure	EFO_0004314	UKBB	European	-0.018	0.003	1.05E-07	255492	0	1	IVNT	4.30E-05
rs6787279	rs6768368	3:57162476_T_C	Forced vital capacity	EFO_0004312	UKBB	European	-0.014	0.003	2.10E-06	307638	0	1	IVNT	6.44E-04
rs6787279	rs6768368	3:57162476_T_C	Forced vital capacity, best measure	EFO_0004312	UKBB	European	-0.014	0.003	1.18E-05	255492	0	1	IVNT	2.98E-03
rs6787279	rs6787279	3:57163751_C_T	Forced expiratory volume in 1-second	EFO_0004314	UKBB	European	0.017	0.003	3.74E-09	307638	0	1	IVNT	2.07E-06
rs6787279	rs6787279	3:57163751_C_T	Forced expiratory volume in 1-second, best measure	EFO_0004314	UKBB	European	0.017	0.003	1.33E-08	255492	0	1	IVNT	6.56E-06
rs6787279	rs6787279	3:57163751_C_T	Forced expiratory volume in 1-second, predicted percentage	EFO_0004314	UKBB	European	0.027	0.006	2.37E-06	110423	0	1	IVNT	7.16E-04
rs6787279	rs6787279	3:57163751_C_T	Forced vital capacity	EFO_0004312	UKBB	European	0.011	0.003	1.65E-05	307638	0	1	IVNT	3.98E-03

sentinel	SNP	chr_bp_eff_other	trait	efo	pmid	ancestry	beta	se	p	n	n_cases	n_studies	unit	FDR
rs6787279	rs6787279	3:57163751_C_T	Forced vital capacity, best measure	EFO_0004312	UKBB	European	0.012	0.003	2.04E-05	255492	0	1	IVNT	4.83E-03
rs6787279	rs6772089	3:57163765_T_C	Forced expiratory volume in 1-second	EFO_0004314	UKBB	European	-0.018	0.003	1.41E-09	307638	0	1	IVNT	8.71E-07
rs6787279	rs6772089	3:57163765_T_C	Forced expiratory volume in 1-second, best measure	EFO_0004314	UKBB	European	-0.018	0.003	1.05E-07	255492	0	1	IVNT	4.28E-05
rs6787279	rs6772089	3:57163765_T_C	Forced vital capacity	EFO_0004312	UKBB	European	-0.014	0.003	2.19E-06	307638	0	1	IVNT	6.66E-04
rs6787279	rs6772089	3:57163765_T_C	Forced vital capacity, best measure	EFO_0004312	UKBB	European	-0.014	0.003	1.20E-05	255492	0	1	IVNT	3.00E-03
rs6787279	rs6796041	3:57163887_T_G	Forced expiratory volume in 1-second	EFO_0004314	UKBB	European	-0.019	0.003	8.72E-10	307638	0	1	IVNT	5.97E-07
rs6787279	rs6796041	3:57163887_T_G	Forced expiratory volume in 1-second, best measure	EFO_0004314	UKBB	European	-0.018	0.003	7.16E-08	255492	0	1	IVNT	3.07E-05
rs6787279	rs6796041	3:57163887_T_G	Forced vital capacity	EFO_0004312	UKBB	European	-0.014	0.003	1.49E-06	307638	0	1	IVNT	4.68E-04
rs6787279	rs6796041	3:57163887_T_G	Forced vital capacity, best measure	EFO_0004312	UKBB	European	-0.014	0.003	8.30E-06	255492	0	1	IVNT	2.20E-03
rs6787279	rs6775080	3:57164362_T_C	Forced expiratory volume in 1-second	EFO_0004314	UKBB	European	-0.018	0.003	1.38E-09	307638	0	1	IVNT	8.57E-07
rs6787279	rs6775080	3:57164362_T_C	Forced expiratory volume in 1-second, best measure	EFO_0004314	UKBB	European	-0.018	0.003	1.04E-07	255492	0	1	IVNT	4.26E-05
rs6787279	rs6775080	3:57164362_T_C	Forced vital capacity	EFO_0004312	UKBB	European	-0.014	0.003	2.14E-06	307638	0	1	IVNT	6.54E-04
rs6787279	rs6775080	3:57164362_T_C	Forced vital capacity, best measure	EFO_0004312	UKBB	European	-0.014	0.003	1.19E-05	255492	0	1	IVNT	2.99E-03
rs6787279	rs6787775	3:57164408_T_A	Forced expiratory volume in 1-second	EFO_0004314	UKBB	European	-0.018	0.003	1.38E-09	307638	0	1	IVNT	8.59E-07
rs6787279	rs6787775	3:57164408_T_A	Forced expiratory volume in 1-second, best measure	EFO_0004314	UKBB	European	-0.018	0.003	1.07E-07	255492	0	1	IVNT	4.35E-05
rs6787279	rs6787775	3:57164408_T_A	Forced vital capacity	EFO_0004312	UKBB	European	-0.014	0.003	2.15E-06	307638	0	1	IVNT	6.56E-04
rs6787279	rs6787775	3:57164408_T_A	Forced vital capacity, best measure	EFO_0004312	UKBB	European	-0.014	0.003	1.22E-05	255492	0	1	IVNT	3.05E-03
rs6787279	rs34313207	3:57164929_A_G	Forced expiratory volume in 1-second	EFO_0004314	UKBB	European	0.019	0.003	7.96E-10	307638	0	1	IVNT	5.51E-07
rs6787279	rs34313207	3:57164929_A_G	Forced expiratory volume in 1-second, best measure	EFO_0004314	UKBB	European	0.018	0.003	6.64E-08	255492	0	1	IVNT	2.87E-05
rs6787279	rs34313207	3:57164929_A_G	Forced vital capacity	EFO_0004312	UKBB	European	0.014	0.003	1.35E-06	307638	0	1	IVNT	4.30E-04
rs6787279	rs34313207	3:57164929_A_G	Forced vital capacity, best measure	EFO_0004312	UKBB	European	0.014	0.003	7.56E-06	255492	0	1	IVNT	2.04E-03
rs6787279	rs4681957	3:57165310_A_C	Forced expiratory volume in 1-second	EFO_0004314	UKBB	European	0.019	0.003	1.27E-09	307638	0	1	IVNT	8.02E-07
rs6787279	rs4681957	3:57165310_A_C	Forced expiratory volume in 1-second, best measure	EFO_0004314	UKBB	European	0.018	0.003	9.62E-08	255492	0	1	IVNT	3.96E-05
rs6787279	rs4681957	3:57165310_A_C	Forced vital capacity	EFO_0004312	UKBB	European	0.014	0.003	1.96E-06	307638	0	1	IVNT	6.02E-04
rs6787279	rs4681957	3:57165310_A_C	Forced vital capacity, best measure	EFO_0004312	UKBB	European	0.014	0.003	1.09E-05	255492	0	1	IVNT	2.76E-03
rs6787279	rs6803905	3:57168273_C_T	Forced expiratory volume in 1-second	EFO_0004314	UKBB	European	0.017	0.003	3.75E-09	307638	0	1	IVNT	2.07E-06
rs6787279	rs6803905	3:57168273_C_T	Forced expiratory volume in 1-second, best measure	EFO_0004314	UKBB	European	0.017	0.003	1.33E-08	255492	0	1	IVNT	6.56E-06
rs6787279	rs6803905	3:57168273_C_T	Forced expiratory volume in 1-second, predicted percentage	EFO_0004314	UKBB	European	0.027	0.006	2.41E-06	110423	0	1	IVNT	7.28E-04
rs6787279	rs6803905	3:57168273_C_T	Forced vital capacity	EFO_0004312	UKBB	European	0.011	0.003	1.65E-05	307638	0	1	IVNT	3.98E-03
rs6787279	rs6803905	3:57168273_C_T	Forced vital capacity, best measure	EFO_0004312	UKBB	European	0.012	0.003	2.02E-05	255492	0	1	IVNT	4.80E-03
rs6787279	rs6788981	3:57168341_T_C	Forced expiratory volume in 1-second	EFO_0004314	UKBB	European	-0.019	0.003	8.24E-10	307638	0	1	IVNT	5.67E-07
rs6787279	rs6788981	3:57168341_T_C	Forced expiratory volume in 1-second, best measure	EFO_0004314	UKBB	European	-0.018	0.003	6.83E-08	255492	0	1	IVNT	2.94E-05
rs6787279	rs6788981	3:57168341_T_C	Forced vital capacity	EFO_0004312	UKBB	European	-0.014	0.003	1.41E-06	307638	0	1	IVNT	4.46E-04
rs6787279	rs6788981	3:57168341_T_C	Forced vital capacity, best measure	EFO_0004312	UKBB	European	-0.014	0.003	7.90E-06	255492	0	1	IVNT	2.10E-03
rs6787279	rs56164953	3:57168793_A_G	Forced expiratory volume in 1-second	EFO_0004314	UKBB	European	0.019	0.003	8.09E-10	307638	0	1	IVNT	5.58E-07
rs6787279	rs56164953	3:57168793_A_G	Forced expiratory volume in 1-second, best measure	EFO_0004314	UKBB	European	0.018	0.003	6.60E-08	255492	0	1	IVNT	2.86E-05
rs6787279	rs56164953	3:57168793_A_G	Forced vital capacity	EFO_0004312	UKBB	European	0.014	0.003	1.41E-06	307638	0	1	IVNT	4.46E-04
rs6787279	rs56164953	3:57168793_A_G	Forced vital capacity, best measure	EFO_0004312	UKBB	European	0.014	0.003	7.80E-06	255492	0	1	IVNT	2.08E-03
rs6787279	rs6807593	3:57169648_C_T	Forced expiratory volume in 1-second	EFO_0004314	UKBB	European	0.018	0.003	1.58E-09	307638	0	1	IVNT	9.60E-07

sentinel	SNP	chr_bp_eff_other	trait	efo	pmid	ancestry	beta	se	p	n	n_cases	n_studies	unit	FDR
rs6787279	rs6807593	3:57169648_C_T	Forced expiratory volume in 1-second, best measure	EFO_0004314	UKBB	European	0.018	0.003	1.09E-07	255492	0	1	IVNT	4.44E-05
rs6787279	rs6807593	3:57169648_C_T	Forced vital capacity	EFO_0004312	UKBB	European	0.014	0.003	2.73E-06	307638	0	1	IVNT	8.18E-04
rs6787279	rs6807593	3:57169648_C_T	Forced vital capacity, best measure	EFO_0004312	UKBB	European	0.014	0.003	1.28E-05	255492	0	1	IVNT	3.20E-03
rs6787279	rs1488121	3:57170495_C_G	Forced expiratory volume in 1-second	EFO_0004314	UKBB	European	0.019	0.003	9.97E-10	307638	0	1	IVNT	6.62E-07
rs6787279	rs1488121	3:57170495_C_G	Forced expiratory volume in 1-second, best measure	EFO_0004314	UKBB	European	0.018	0.003	8.19E-08	255492	0	1	IVNT	3.42E-05
rs6787279	rs1488121	3:57170495_C_G	Forced vital capacity	EFO_0004312	UKBB	European	0.014	0.003	1.81E-06	307638	0	1	IVNT	5.60E-04
rs6787279	rs1488121	3:57170495_C_G	Forced vital capacity, best measure	EFO_0004312	UKBB	European	0.014	0.003	9.18E-06	255492	0	1	IVNT	2.40E-03
rs6787279	rs6774301	3:57171554_C_G	Forced expiratory volume in 1-second	EFO_0004314	UKBB	European	0.018	0.003	1.59E-09	307638	0	1	IVNT	9.65E-07
rs6787279	rs6774301	3:57171554_C_G	Forced expiratory volume in 1-second, best measure	EFO_0004314	UKBB	European	0.018	0.003	1.15E-07	255492	0	1	IVNT	4.64E-05
rs6787279	rs6774301	3:57171554_C_G	Forced vital capacity	EFO_0004312	UKBB	European	0.014	0.003	2.74E-06	307638	0	1	IVNT	8.18E-04
rs6787279	rs6774301	3:57171554_C_G	Forced vital capacity, best measure	EFO_0004312	UKBB	European	0.014	0.003	1.32E-05	255492	0	1	IVNT	3.26E-03
rs6787279	rs6445858	3:57171902_C_T	Forced expiratory volume in 1-second	EFO_0004314	UKBB	European	0.019	0.003	9.81E-10	307638	0	1	IVNT	6.55E-07
rs6787279	rs6445858	3:57171902_C_T	Forced expiratory volume in 1-second, best measure	EFO_0004314	UKBB	European	0.018	0.003	7.61E-08	255492	0	1	IVNT	3.22E-05
rs6787279	rs6445858	3:57171902_C_T	Forced vital capacity	EFO_0004312	UKBB	European	0.014	0.003	1.82E-06	307638	0	1	IVNT	5.63E-04
rs6787279	rs6445858	3:57171902_C_T	Forced vital capacity, best measure	EFO_0004312	UKBB	European	0.014	0.003	8.70E-06	255492	0	1	IVNT	2.30E-03
rs6787279	rs79625814	3:57173021_T_A	Forced expiratory volume in 1-second	EFO_0004314	UKBB	European	-0.019	0.003	9.39E-10	307638	0	1	IVNT	6.31E-07
rs6787279	rs79625814	3:57173021_T_A	Forced expiratory volume in 1-second, best measure	EFO_0004314	UKBB	European	-0.018	0.003	7.19E-08	255492	0	1	IVNT	3.07E-05
rs6787279	rs79625814	3:57173021_T_A	Forced vital capacity	EFO_0004312	UKBB	European	-0.014	0.003	1.64E-06	307638	0	1	IVNT	5.12E-04
rs6787279	rs79625814	3:57173021_T_A	Forced vital capacity, best measure	EFO_0004312	UKBB	European	-0.014	0.003	8.42E-06	255492	0	1	IVNT	2.23E-03
rs6787279	rs1906504	3:57175454_G_A	Forced expiratory volume in 1-second	EFO_0004314	UKBB	European	-0.018	0.003	1.57E-09	307638	0	1	IVNT	9.59E-07
rs6787279	rs1906504	3:57175454_G_A	Forced expiratory volume in 1-second, best measure	EFO_0004314	UKBB	European	-0.018	0.003	1.16E-07	255492	0	1	IVNT	4.68E-05
rs6787279	rs1906504	3:57175454_G_A	Forced vital capacity	EFO_0004312	UKBB	European	-0.014	0.003	2.60E-06	307638	0	1	IVNT	7.81E-04
rs6787279	rs1906504	3:57175454_G_A	Forced vital capacity, best measure	EFO_0004312	UKBB	European	-0.014	0.003	1.29E-05	255492	0	1	IVNT	3.22E-03
rs6787279	rs1386828	3:57176197_A_G	Forced expiratory volume in 1-second	EFO_0004314	UKBB	European	0.019	0.003	9.85E-10	307638	0	1	IVNT	6.55E-07
rs6787279	rs1386828	3:57176197_A_G	Forced expiratory volume in 1-second, best measure	EFO_0004314	UKBB	European	0.018	0.003	8.20E-08	255492	0	1	IVNT	3.42E-05
rs6787279	rs1386828	3:57176197_A_G	Forced vital capacity	EFO_0004312	UKBB	European	0.014	0.003	1.81E-06	307638	0	1	IVNT	5.59E-04
rs6787279	rs1386828	3:57176197_A_G	Forced vital capacity, best measure	EFO_0004312	UKBB	European	0.014	0.003	9.27E-06	255492	0	1	IVNT	2.42E-03
rs6787279	rs1386829	3:57176505_G_A	Forced expiratory volume in 1-second	EFO_0004314	UKBB	European	-0.019	0.003	9.83E-10	307638	0	1	IVNT	6.55E-07
rs6787279	rs1386829	3:57176505_G_A	Forced expiratory volume in 1-second, best measure	EFO_0004314	UKBB	European	-0.018	0.003	8.19E-08	255492	0	1	IVNT	3.42E-05
rs6787279	rs1386829	3:57176505_G_A	Forced vital capacity	EFO_0004312	UKBB	European	-0.014	0.003	1.81E-06	307638	0	1	IVNT	5.59E-04
rs6787279	rs1386829	3:57176505_G_A	Forced vital capacity, best measure	EFO_0004312	UKBB	European	-0.014	0.003	9.29E-06	255492	0	1	IVNT	2.42E-03
rs6787279	rs12496376	3:57177171_G_A	Forced expiratory volume in 1-second	EFO_0004314	UKBB	European	-0.018	0.003	1.68E-09	307638	0	1	IVNT	1.00E-06
rs6787279	rs12496376	3:57177171_G_A	Forced expiratory volume in 1-second, best measure	EFO_0004314	UKBB	European	-0.018	0.003	1.23E-07	255492	0	1	IVNT	4.94E-05
rs6787279	rs12496376	3:57177171_G_A	Forced vital capacity	EFO_0004312	UKBB	European	-0.014	0.003	2.70E-06	307638	0	1	IVNT	8.10E-04
rs6787279	rs12496376	3:57177171_G_A	Forced vital capacity, best measure	EFO_0004312	UKBB	European	-0.014	0.003	1.37E-05	255492	0	1	IVNT	3.38E-03
rs6787279	rs6801574	3:57177195_T_C	Forced expiratory volume in 1-second	EFO_0004314	UKBB	European	-0.016	0.003	4.37E-09	307638	0	1	IVNT	2.37E-06
rs6787279	rs6801574	3:57177195_T_C	Forced expiratory volume in 1-second, best measure	EFO_0004314	UKBB	European	-0.017	0.003	1.60E-08	255492	0	1	IVNT	7.82E-06
rs6787279	rs6801574	3:57177195_T_C	Forced expiratory volume in 1-second, predicted percentage	EFO_0004314	UKBB	European	-0.027	0.006	3.42E-06	110423	0	1	IVNT	9.92E-04

sentinel	SNP	chr_bp_eff_other	trait	efo	pmid	ancestry	beta	se	p	n	n_cases	n_studies	unit	FDR
rs6787279	rs6801574	3:57177195_T_C	Forced vital capacity	EFO_0004312	UKBB	European	-0.011	0.003	1.99E-05	307638	0	1	IVNT	4.72E-03
rs6787279	rs6801574	3:57177195_T_C	Forced vital capacity, best measure	EFO_0004312	UKBB	European	-0.012	0.003	2.33E-05	255492	0	1	IVNT	5.42E-03
rs6787279	rs76208275	3:57177716_C_T	Forced expiratory volume in 1-second	EFO_0004314	UKBB	European	0.018	0.003	1.52E-09	307638	0	1	IVNT	9.32E-07
rs6787279	rs76208275	3:57177716_C_T	Forced expiratory volume in 1-second, best measure	EFO_0004314	UKBB	European	0.018	0.003	1.12E-07	255492	0	1	IVNT	4.53E-05
rs6787279	rs76208275	3:57177716_C_T	Forced vital capacity	EFO_0004312	UKBB	European	0.014	0.003	2.55E-06	307638	0	1	IVNT	7.67E-04
rs6787279	rs76208275	3:57177716_C_T	Forced vital capacity, best measure	EFO_0004312	UKBB	European	0.014	0.003	1.28E-05	255492	0	1	IVNT	3.20E-03
rs6787279	rs12494525	3:57178293_T_C	Forced expiratory volume in 1-second	EFO_0004314	UKBB	European	-0.018	0.003	1.64E-09	307638	0	1	IVNT	9.80E-07
rs6787279	rs12494525	3:57178293_T_C	Forced expiratory volume in 1-second, best measure	EFO_0004314	UKBB	European	-0.018	0.003	1.17E-07	255492	0	1	IVNT	4.70E-05
rs6787279	rs12494525	3:57178293_T_C	Forced vital capacity	EFO_0004312	UKBB	European	-0.014	0.003	2.50E-06	307638	0	1	IVNT	7.54E-04
rs6787279	rs12494525	3:57178293_T_C	Forced vital capacity, best measure	EFO_0004312	UKBB	European	-0.014	0.003	1.37E-05	255492	0	1	IVNT	3.37E-03
rs6787279	rs6808185	3:57179261_G_C	Forced expiratory volume in 1-second	EFO_0004314	UKBB	European	-0.019	0.003	8.48E-10	307638	0	1	IVNT	5.82E-07
rs6787279	rs6808185	3:57179261_G_C	Forced expiratory volume in 1-second, best measure	EFO_0004314	UKBB	European	-0.018	0.003	7.21E-08	255492	0	1	IVNT	3.08E-05
rs6787279	rs6808185	3:57179261_G_C	Forced vital capacity	EFO_0004312	UKBB	European	-0.014	0.003	1.44E-06	307638	0	1	IVNT	4.54E-04
rs6787279	rs6808185	3:57179261_G_C	Forced vital capacity, best measure	EFO_0004312	UKBB	European	-0.014	0.003	8.31E-06	255492	0	1	IVNT	2.20E-03
rs6787279	rs17057784	3:57180402_A_C	Forced expiratory volume in 1-second	EFO_0004314	UKBB	European	0.019	0.003	1.09E-09	307638	0	1	IVNT	7.11E-07
rs6787279	rs17057784	3:57180402_A_C	Forced expiratory volume in 1-second, best measure	EFO_0004314	UKBB	European	0.018	0.003	6.93E-08	255492	0	1	IVNT	2.98E-05
rs6787279	rs17057784	3:57180402_A_C	Forced vital capacity	EFO_0004312	UKBB	European	0.014	0.003	1.74E-06	307638	0	1	IVNT	5.40E-04
rs6787279	rs17057784	3:57180402_A_C	Forced vital capacity, best measure	EFO_0004312	UKBB	European	0.014	0.003	7.41E-06	255492	0	1	IVNT	2.00E-03
rs6787279	rs9823843	3:57181631_C_T	Forced expiratory volume in 1-second	EFO_0004314	UKBB	European	0.019	0.003	1.05E-09	307638	0	1	IVNT	6.89E-07
rs6787279	rs9823843	3:57181631_C_T	Forced expiratory volume in 1-second, best measure	EFO_0004314	UKBB	European	0.018	0.003	8.07E-08	255492	0	1	IVNT	3.38E-05
rs6787279	rs9823843	3:57181631_C_T	Forced vital capacity	EFO_0004312	UKBB	European	0.014	0.003	1.96E-06	307638	0	1	IVNT	6.04E-04
rs6787279	rs9823843	3:57181631_C_T	Forced vital capacity, best measure	EFO_0004312	UKBB	European	0.014	0.003	9.24E-06	255492	0	1	IVNT	2.42E-03
rs6787279	rs17216900	3:57183877_C_T	Forced expiratory volume in 1-second	EFO_0004314	UKBB	European	0.017	0.003	3.63E-09	307638	0	1	IVNT	2.03E-06
rs6787279	rs17216900	3:57183877_C_T	Forced expiratory volume in 1-second, best measure	EFO_0004314	UKBB	European	0.018	0.003	1.01E-08	255492	0	1	IVNT	5.15E-06
rs6787279	rs17216900	3:57183877_C_T	Forced expiratory volume in 1-second, predicted percentage	EFO_0004314	UKBB	European	0.026	0.006	5.08E-06	110423	0	1	IVNT	1.42E-03
rs6787279	rs17216900	3:57183877_C_T	Forced vital capacity, best measure	EFO_0004312	UKBB	European	0.013	0.003	1.57E-05	255492	0	1	IVNT	3.82E-03
rs6787279	rs17216900	3:57183877_C_T	Forced vital capacity	EFO_0004312	UKBB	European	0.011	0.003	1.64E-05	307638	0	1	IVNT	3.95E-03
rs6787279	rs4475007	3:57186159_G_A	Forced expiratory volume in 1-second, best measure	EFO_0004314	UKBB	European	-0.012	0.003	5.56E-06	255492	0	1	IVNT	1.54E-03
rs6787279	rs3968279	3:57187078_C_T	Forced expiratory volume in 1-second	EFO_0004314	UKBB	European	-0.018	0.003	1.50E-08	307638	0	1	IVNT	7.38E-06
rs6787279	rs3968279	3:57187078_C_T	Forced expiratory volume in 1-second, best measure	EFO_0004314	UKBB	European	-0.019	0.004	7.99E-08	255492	0	1	IVNT	3.35E-05
rs6787279	rs3968279	3:57187078_C_T	Forced expiratory volume in 1-second, predicted percentage	EFO_0004314	UKBB	European	-0.031	0.007	2.40E-06	110423	0	1	IVNT	7.26E-04
rs6787279	rs9823220	3:57190211_T_G	Forced expiratory volume in 1-second	EFO_0004314	UKBB	European	-0.018	0.003	3.23E-08	307638	0	1	IVNT	1.50E-05
rs6787279	rs9823220	3:57190211_T_G	Forced expiratory volume in 1-second, best measure	EFO_0004314	UKBB	European	-0.018	0.004	1.76E-07	255492	0	1	IVNT	6.85E-05
rs6787279	rs9823220	3:57190211_T_G	Forced expiratory volume in 1-second, predicted percentage	EFO_0004314	UKBB	European	-0.031	0.007	2.92E-06	110423	0	1	IVNT	8.67E-04
rs6787279	rs7431253	3:57190452_G_A	Forced expiratory volume in 1-second	EFO_0004314	UKBB	European	-0.018	0.003	2.46E-08	307638	0	1	IVNT	1.17E-05
rs6787279	rs7431253	3:57190452_G_A	Forced expiratory volume in 1-second, best measure	EFO_0004314	UKBB	European	-0.019	0.004	1.35E-07	255492	0	1	IVNT	5.37E-05

sentinel	SNP	chr_bp_eff_other	trait	efo	pmid	ancestry	beta	se	p	n	n_cases	n_studies	unit	FDR
rs6787279	rs7431253	3:57190452_G_A	Forced expiratory volume in 1-second, predicted percentage	EFO_0004314	UKBB	European	-0.031	0.007	3.01E-06	110423	0	1	IVNT	8.89E-04
rs6787279	rs35424480	3:57190811_C_G	Forced expiratory volume in 1-second	EFO_0004314	UKBB	European	0.018	0.003	2.51E-08	307638	0	1	IVNT	1.19E-05
rs6787279	rs35424480	3:57190811_C_G	Forced expiratory volume in 1-second, best measure	EFO_0004314	UKBB	European	0.019	0.004	1.55E-07	255492	0	1	IVNT	6.11E-05
rs6787279	rs35424480	3:57190811_C_G	Forced expiratory volume in 1-second, predicted percentage	EFO_0004314	UKBB	European	0.031	0.007	2.94E-06	110423	0	1	IVNT	8.73E-04
rs6787279	rs1906505	3:57194272_T_C	Forced expiratory volume in 1-second	EFO_0004314	UKBB	European	-0.018	0.003	3.71E-08	307638	0	1	IVNT	1.70E-05
rs6787279	rs1906505	3:57194272_T_C	Forced expiratory volume in 1-second, best measure	EFO_0004314	UKBB	European	-0.018	0.004	2.04E-07	255492	0	1	IVNT	7.90E-05
rs6787279	rs1906505	3:57194272_T_C	Forced expiratory volume in 1-second, predicted percentage	EFO_0004314	UKBB	European	-0.031	0.007	3.77E-06	110423	0	1	IVNT	1.09E-03
rs16903574	rs16903574	5:14610309_G_C	Self-reported asthma	EFO_0000270	UKBB	European	-0.011	0.001	1.52E-13	337159	39049	1	risk diff	2.68E-10
rs16903574	rs16903574	5:14610309_G_C	Asthma	EFO_0000270	UKBB	European	-0.011	0.001	4.86E-13	336782	38791	1	risk diff	7.12E-10
rs16903574	rs16903574	5:14610309_G_C	Allergic disease asthma hay fever or eczema	EFO_0003785	29083406	European	-0.069	0.010	1.00E-12	-	-	-	log OR	1.36E-09
rs16903574	rs16903574	5:14610309_G_C	No blood clot, bronchitis, emphysema, asthma, rhinitis, eczema or allergy diagnosed by doctor	-	UKBB	European	0.014	0.002	3.35E-11	336782	228530	1	risk diff	3.09E-08
rs16903574	rs16903574	5:14610309_G_C	Allergic disease	EFO_0003785	29083406	European	-0.070	0.011	1.68E-10	360838	180129	13	log OR	1.33E-07
rs16903574	rs16903574	5:14610309_G_C	Doctor diagnosed asthma	EFO_0000270	UKBB	European	-0.019	0.003	3.42E-09	83529	10589	1	risk diff	1.94E-06
rs16903574	rs16903574	5:14610309_G_C	Eosinophil percentage of white cells	EFO_0004586	27863252	European	-0.036	0.007	5.98E-08	173480	0	2	IVNT	2.62E-05
rs16903574	rs16903574	5:14610309_G_C	Eosinophil percentage of granulocytes	EFO_0004586	27863252	European	-0.034	0.007	4.37E-07	173480	0	2	IVNT	1.58E-04
rs16903574	rs16903574	5:14610309_G_C	Eosinophil count	EFO_0004586	27863252	European	-0.034	0.007	5.26E-07	173480	0	2	IVNT	1.88E-04
rs16903574	rs16903574	5:14610309_G_C	Hayfever, allergic rhinitis or eczema	EFO_0003956; EFO_0005854; EFO_0000274	UKBB	European	-0.009	0.002	4.90E-06	336782	77891	1	risk diff	1.38E-03
rs16903574	rs16903574	5:14610309_G_C	Neutrophil percentage of granulocytes	EFO_0004586	27863252	European	0.030	0.007	9.77E-06	173480	0	2	IVNT	2.53E-03
rs16903574	rs16903574	5:14610309_G_C	Sum eosinophil basophil counts	EFO_0004586	27863252	European	-0.029	0.007	1.42E-05	173480	0	2	IVNT	3.48E-03
rs16903574	rs16903574	5:14610309_G_C	Wheeze or whistling in the chest in last year	EFO_0009715	UKBB	European	-0.008	0.002	2.84E-05	331257	68531	1	risk diff	6.44E-03
rs16903574	rs16903574	5:14610309_G_C	Mineral and other dietary supplements: calcium	EFO_0008111	UKBB	European	0.005	0.001	4.53E-05	336314	22047	1	risk diff	9.60E-03
rs80101740	rs80101740	5:98471135_C_A	Postprocedural disorders of eye and adnexa	EFO_0009546	UKBB	European	-0.001	0.000	1.36E-07	337199	60	1	risk diff	5.39E-05
rs80101740	rs80101740	5:98471135_C_A	Treatment with phenoxymethylpenicillin	EFO_0007056	UKBB	European	-0.001	0.000	8.51E-07	337159	68	1	risk diff	2.88E-04
rs80101740	rs80101740	5:98471135_C_A	Cause of death: peripheral vascular disease, unspecified	EFO_0003875	UKBB	European	-0.008	0.002	3.41E-06	7637	5	1	risk diff	9.91E-04
rs80101740	rs138827081	5:98511774_A_G	Postprocedural disorders of eye and adnexa	EFO_0009546	UKBB	European	0.001	0.000	1.00E-06	337199	60	1	risk diff	3.29E-04
rs80101740	rs138827081	5:98511774_A_G	Treatment with phenoxymethylpenicillin	EFO_0007056	UKBB	European	0.001	0.000	1.37E-06	337159	68	1	risk diff	4.33E-04
rs80101740	rs138827081	5:98511774_A_G	Cause of death: peripheral vascular disease, unspecified	EFO_0003875	UKBB	European	0.008	0.002	9.34E-06	7637	5	1	risk diff	2.43E-03
rs80101740	rs138827081	5:98511774_A_G	Malignant neoplasm of cervix uteri	EFO_0001061	UKBB	European	0.001	0.000	1.36E-05	337199	145	1	risk diff	3.36E-03
rs80101740	rs138827081	5:98511774_A_G	Self-reported vaginal prolapse or uterine prolapse	EFO_1001864	UKBB	European	0.004	0.001	1.67E-05	337159	1870	1	risk diff	4.01E-03
rs80101740	rs183720747	5:98516580_G_T	Postprocedural disorders of eye and adnexa	EFO_0009546	UKBB	European	0.001	0.000	1.07E-06	337199	60	1	risk diff	3.46E-04
rs80101740	rs183720747	5:98516580_G_T	Treatment with phenoxymethylpenicillin	EFO_0007056	UKBB	European	0.001	0.000	1.36E-06	337159	68	1	risk diff	4.32E-04
rs80101740	rs183720747	5:98516580_G_T	Cause of death: peripheral vascular disease, unspecified	EFO_0003875	UKBB	European	0.008	0.002	9.18E-06	7637	5	1	risk diff	2.40E-03
rs80101740	rs183720747	5:98516580_G_T	Malignant neoplasm of cervix uteri	EFO_0001061	UKBB	European	0.001	0.000	1.29E-05	337199	145	1	risk diff	3.21E-03
rs80101740	rs183720747	5:98516580_G_T	Self-reported vaginal prolapse or uterine prolapse	EFO_1001864	UKBB	European	0.003	0.001	2.18E-05	337159	1870	1	risk diff	5.13E-03
rs80101740	rs144009750	5:98530592_T_C	Treatment with phenoxymethylpenicillin	EFO_0007056	UKBB	European	-0.001	0.000	2.31E-06	337159	68	1	risk diff	7.03E-04
rs80101740	rs144009750	5:98530592_T_C	Postprocedural disorders of eye and adnexa	EFO_0009546	UKBB	European	-0.001	0.000	3.90E-06	337199	60	1	risk diff	1.12E-03

sentinel	SNP	chr_bp_eff_other	trait	efo	pmid	ancestry	beta	se	p	n	n_cases	n_studies	unit	FDR
rs80101740	rs144009750	5:98530592_T_C	Cause of death: aortic stenosis	EFO_0000266	UKBB	European	-0.016	0.004	2.68E-05	7637	21	1	risk diff	6.14E-03
rs80101740	rs144009750	5:98530592_T_C	Malignant neoplasm of cervix uteri	EFO_0001061	UKBB	European	-0.001	0.000	4.39E-05	337199	145	1	risk diff	9.36E-03
rs80101740	rs144009750	5:98530592_T_C	Self-reported vaginal prolapse or uterine prolapse	EFO_1001864	UKBB	European	-0.003	0.001	4.47E-05	337159	1870	1	risk diff	9.51E-03
rs80101740	rs149035101	5:98534794_G_A	Treatment with phenoxymethylpenicillin	EFO_0007056	UKBB	European	-0.001	0.000	2.33E-06	337159	68	1	risk diff	7.09E-04
rs80101740	rs149035101	5:98534794_G_A	Postprocedural disorders of eye and adnexa	EFO_0009546	UKBB	European	-0.001	0.000	5.02E-06	337199	60	1	risk diff	1.41E-03
rs80101740	rs149035101	5:98534794_G_A	Cause of death: aortic stenosis	EFO_0000266	UKBB	European	-0.015	0.004	3.68E-05	7637	21	1	risk diff	7.98E-03
rs1837253	rs1837253	5:110401872_C_T	Self-reported asthma	EFO_0000270	UKBB	European	0.012	0.001	8.10E-40	337159	39049	1	risk diff	2.60E-35
rs1837253	rs1837253	5:110401872_C_T	Asthma	EFO_0000270	UKBB	European	0.011	0.001	4.20E-38	336782	38791	1	risk diff	1.08E-33
rs1837253	rs1837253	5:110401872_C_T	Allergic disease asthma hay fever or eczema	EFO_0003785	29083406	European	NA	NA	2.00E-31	-	-	-	log OR	4.68E-27
rs1837253	rs1837253	5:110401872_C_T	Asthma	EFO_0000270	27182965	European	NA	NA	3.00E-31	-	-	-	-	6.43E-27
rs1837253	rs1837253	5:110401872_C_T	Asthma	EFO_0000270	29273806	Mixed	0.146	0.014	2.03E-25	142486	23948	66	log OR	2.61E-21
rs1837253	rs1837253	5:110401872_C_T	No blood clot, bronchitis, emphysema, asthma, rhinitis, eczema or allergy diagnosed by doctor	-	UKBB	European	-0.013	0.001	1.45E-24	336782	228530	1	risk diff	1.62E-20
rs1837253	rs1837253	5:110401872_C_T	Eosinophil count	EFO_0004586	27863252	European	0.040	0.004	1.88E-22	173480	0	2	IVNT	1.61E-18
rs1837253	rs1837253	5:110401872_C_T	Eosinophil counts	EFO_0004842	27863252	European	0.040	0.004	2.00E-22	-	-	-	unit increase	1.66E-18
rs1837253	rs1837253	5:110401872_C_T	Doctor diagnosed asthma	EFO_0000270	UKBB	European	0.018	0.002	8.13E-22	83529	10589	1	risk diff	6.16E-18
rs1837253	rs1837253	5:110401872_C_T	Asthma	EFO_0000270	29273806	European	0.145	0.015	1.06E-21	127669	19954	56	log OR	7.79E-18
rs1837253	rs1837253	5:110401872_C_T	Eosinophil percentage of white cells	EFO_0007991	27863252	European	0.038	0.004	4.00E-21	-	-	-	unit increase	2.64E-17
rs1837253	rs1837253	5:110401872_C_T	Eosinophil percentage of white cells	EFO_0004586	27863252	European	0.038	0.004	4.49E-21	173480	0	2	IVNT	2.89E-17
rs1837253	rs1837253	5:110401872_C_T	Allergic disease	EFO_0003785	29083406	European	0.059	0.007	1.81E-19	360838	180129	13	log OR	9.32E-16
rs1837253	rs1837253	5:110401872_C_T	Sum eosinophil basophil counts	EFO_0004586	27863252	European	0.036	0.004	5.28E-19	173480	0	2	IVNT	2.34E-15
rs1837253	rs1837253	5:110401872_C_T	Eosinophil percentage of granulocytes	EFO_0007996	27863252	European	0.036	0.004	2.00E-18	-	-	-	unit increase	8.58E-15
rs1837253	rs1837253	5:110401872_C_T	Eosinophil percentage of granulocytes	EFO_0004586	27863252	European	0.036	0.004	2.32E-18	173480	0	2	IVNT	9.64E-15
rs1837253	rs1837253	5:110401872_C_T	Asthma	EFO_0000270	21804548	East Asian	0.157	0.019	1.00E-16	-	-	-	log OR	3.22E-13
rs1837253	rs1837253	5:110401872_C_T	Asthma	EFO_0000270	21804548	East Asian	NA	NA	1.00E-16	-	-	-	-	3.22E-13
rs1837253	rs1837253	5:110401872_C_T	Adult asthma	EFO_1002011	21804548	East Asian	NA	NA	1.24E-16	4836	-	-	-	3.94E-13
rs1837253	rs1837253	5:110401872_C_T	Wheeze or whistling in the chest in last year	EFO_0009715	UKBB	European	0.009	0.001	1.18E-15	331257	68531	1	risk diff	3.50E-12
rs1837253	rs1837253	5:110401872_C_T	Treatment with ventolin 100micrograms inhaler	EFO_0007056	UKBB	European	0.004	0.000	1.58E-15	337159	9712	1	risk diff	4.62E-12
rs1837253	rs1837253	5:110401872_C_T	Neutrophil percentage of granulocytes	EFO_0004586	27863252	European	-0.032	0.004	2.73E-15	173480	0	2	IVNT	7.71E-12
rs1837253	rs1837253	5:110401872_C_T	Neutrophil percentage of granulocytes	EFO_0007994	27863252	European	0.032	0.004	3.00E-15	-	-	-	unit decrease	8.21E-12
rs1837253	rs1837253	5:110401872_C_T	Nasal polyp	EFO_1000391	UKBB	European	0.002	0.000	7.06E-15	337199	1637	1	risk diff	1.73E-11
rs1837253	rs1837253	5:110401872_C_T	Asthma	EFO_0000270	21804549	Mixed	NA	NA	1.00E-14	5388	-	-	-	2.32E-11
rs1837253	rs1837253	5:110401872_C_T	Asthma	EFO_0000270	21804549	Mixed	NA	NA	1.00E-14	-	-	-	-	2.32E-11
rs1837253	rs1837253	5:110401872_C_T	Asthma	EFO_0000270	21804549	Mixed	NA	NA	1.00E-14	-	-	-	-	2.32E-11
rs1837253	rs1837253	5:110401872_C_T	Hayfever, allergic rhinitis or eczema	EFO_0003956; EFO_0005854; EFO_0000274	UKBB	European	0.009	0.001	1.15E-14	336782	77891	1	risk diff	2.58E-11
rs1837253	rs1837253	5:110401872_C_T	Self-reported hayfever or allergic rhinitis	EFO_0005854	UKBB	European	0.005	0.001	1.58E-13	337159	19320	1	risk diff	2.74E-10
rs1837253	rs1837253	5:110401872_C_T	Asthma childhood later and unknown onset and severe asthma	-	20860503	European	NA	NA	1.37E-10	26475	-	-	-	1.10E-07
rs1837253	rs1837253	5:110401872_C_T	Asthma	EFO_0000270	20860503	European	0.148	0.023	3.03E-10	26475	10365	24	log OR	2.31E-07
rs1837253	rs1837253	5:110401872_C_T	Asthma childhood later and unknown onset and severe and industrial asthma	-	20860503	European	NA	NA	3.03E-10	26475	-	-	-	2.31E-07

sentinel	SNP	chr_bp_eff_other	trait	efo	pmid	ancestry	beta	se	p	n	n_cases	n_studies	unit	FDR
rs1837253	rs1837253	5:110401872_C_T	Asthma and hay fever	EFO_0003956; EFO_0000270	24388013	European	0.157	0.026	1.00E-09	-	-	-	log OR	6.62E-07
rs1837253	rs1837253	5:110401872_C_T	Self-reported nasal polyps	EFO_1000391	UKBB	European	0.001	0.000	4.64E-09	337159	1489	1	risk diff	2.50E-06
rs1837253	rs1837253	5:110401872_C_T	Doctor diagnosed hayfever or allergic rhinitis	EFO_0005854	UKBB	European	0.014	0.002	6.08E-09	83529	18934	1	risk diff	3.21E-06
rs1837253	rs1837253	5:110401872_C_T	Asthma	EFO_0000270	27611488	Mixed	NA	NA	7.00E-08	-	-	-	-	3.00E-05
rs1837253	rs1837253	5:110401872_C_T	Asthma childhood and later onset	-	20860503	European	NA	NA	7.52E-08	26475	-	-	-	3.19E-05
rs1837253	rs1837253	5:110401872_C_T	Asthma	EFO_0000270	27611488	Mixed	NA	NA	4.00E-07	-	-	-	-	1.47E-04
rs1837253	rs1837253	5:110401872_C_T	Number of self-reported non-cancer illnesses	EFO_0009801	UKBB	European	0.011	0.002	2.55E-06	337159	0	1	-	7.67E-04
rs1837253	rs1837253	5:110401872_C_T	Severe asthma	EFO_0000270	20860503	European	NA	NA	3.00E-06	26475	-	-	-	8.87E-04
rs1837253	rs1837253	5:110401872_C_T	Taking other prescription medications	EFO_0007056	UKBB	European	0.006	0.001	3.65E-06	336330	158113	1	risk diff	1.05E-03
rs1837253	rs1837253	5:110401872_C_T	Asthma severe asthma	EFO_0000270	22561531	European	NA	NA	5.52E-06	4279	-	-	-	1.53E-03
rs1837253	rs1837253	5:110401872_C_T	Asthma childhood onset	EFO_0004591	20860503	European	NA	NA	7.76E-06	26475	-	-	-	2.08E-03
rs3749833	rs59041913	5:131788135_T_C	Self-reported asthma	EFO_0000270	UKBB	European	-0.008	0.001	2.68E-19	337159	39049	1	risk diff	1.28E-15
rs3749833	rs59041913	5:131788135_T_C	Asthma	EFO_0000270	UKBB	European	-0.008	0.001	1.01E-17	336782	38791	1	risk diff	3.75E-14
rs3749833	rs59041913	5:131788135_T_C	Sum eosinophil basophil counts	EFO_0004586	27863252	European	-0.031	0.004	3.51E-14	173480	0	2	IVNT	7.23E-11
rs3749833	rs59041913	5:131788135_T_C	Crohns disease	EFO_0000384	26192919	European	0.217	0.030	1.97E-13	20883	5956	7	log OR	3.33E-10
rs3749833	rs59041913	5:131788135_T_C	No blood clot, bronchitis, emphysema, asthma, rhinitis, eczema or allergy diagnosed by doctor	-	UKBB	European	0.010	0.001	7.72E-13	336782	228530	1	risk diff	1.09E-09
rs3749833	rs59041913	5:131788135_T_C	Eosinophil count	EFO_0004586	27863252	European	-0.029	0.004	9.28E-13	173480	0	2	IVNT	1.27E-09
rs3749833	rs59041913	5:131788135_T_C	Height	EFO_0004339	UKBB	European	0.014	0.002	2.50E-11	336474	0	1	IVNT	2.42E-08
rs3749833	rs59041913	5:131788135_T_C	Mean platelet volume	EFO_0004586	27863252	European	0.027	0.004	2.60E-11	173480	0	2	IVNT	2.51E-08
rs3749833	rs59041913	5:131788135_T_C	Myeloid white cell count	EFO_0004586	27863252	European	-0.027	0.004	4.74E-11	173480	0	2	IVNT	4.28E-08
rs3749833	rs59041913	5:131788135_T_C	Granulocyte count	EFO_0004586	27863252	European	-0.026	0.004	1.44E-10	173480	0	2	IVNT	1.15E-07
rs3749833	rs59041913	5:131788135_T_C	Sum neutrophil eosinophil counts	EFO_0004586	27863252	European	-0.025	0.004	4.81E-10	173480	0	2	IVNT	3.52E-07
rs3749833	rs59041913	5:131788135_T_C	Inflammatory bowel disease	EFO_0003767	26192919	European	0.129	0.021	8.94E-10	34652	12882	15	log OR	6.09E-07
rs3749833	rs59041913	5:131788135_T_C	Sum basophil neutrophil counts	EFO_0004586	27863252	European	-0.023	0.004	6.32E-09	173480	0	2	IVNT	3.32E-06
rs3749833	rs59041913	5:131788135_T_C	White blood cell count	EFO_0004586	27863252	European	-0.023	0.004	1.30E-08	173480	0	2	IVNT	6.44E-06
rs3749833	rs59041913	5:131788135_T_C	Neutrophil count	EFO_0004586	27863252	European	-0.023	0.004	1.70E-08	173480	0	2	IVNT	8.27E-06
rs3749833	rs59041913	5:131788135_T_C	Eosinophil percentage of white cells	EFO_0004586	27863252	European	-0.022	0.004	7.82E-08	173480	0	2	IVNT	3.29E-05
rs3749833	rs59041913	5:131788135_T_C	Lymphocyte percentage of white cells	EFO_0004586	27863252	European	0.020	0.004	3.58E-07	173480	0	2	IVNT	1.32E-04
rs3749833	rs59041913	5:131788135_T_C	Allergic disease	EFO_0003785	29083406	European	-0.032	0.007	9.14E-07	360838	180129	13	log OR	3.05E-04
rs3749833	rs59041913	5:131788135_T_C	Comparative height size at age 10	-	UKBB	European	0.009	0.002	1.45E-06	332021	0	1	-	4.56E-04
rs3749833	rs59041913	5:131788135_T_C	Hayfever, allergic rhinitis or eczema	EFO_0003956; EFO_0005854; EFO_0000274	UKBB	European	-0.006	0.001	1.66E-06	336782	77891	1	risk diff	5.14E-04
rs3749833	rs59041913	5:131788135_T_C	Monocyte count	EFO_0004586	27863252	European	-0.018	0.004	1.07E-05	173480	0	2	IVNT	2.74E-03
rs3749833	rs59041913	5:131788135_T_C	Treatment with vitamin a+d capsule	EFO_0007056	UKBB	European	0.000	0.000	1.08E-05	337159	53	1	risk diff	2.74E-03
rs3749833	rs59041913	5:131788135_T_C	Self-reported nasal polyps	EFO_1000391	UKBB	European	-0.001	0.000	1.90E-05	337159	1489	1	risk diff	4.53E-03
rs3749833	rs59041913	5:131788135_T_C	Neutrophil percentage of granulocytes	EFO_0004586	27863252	European	0.017	0.004	2.38E-05	173480	0	2	IVNT	5.51E-03
rs3749833	rs59041913	5:131788135_T_C	Platelet count	EFO_0004586	27863252	European	-0.017	0.004	2.49E-05	173480	0	2	IVNT	5.74E-03
rs3749833	rs59041913	5:131788135_T_C	Doctor diagnosed asthma	EFO_0000270	UKBB	European	-0.008	0.002	3.26E-05	83529	10589	1	risk diff	7.19E-03
rs3749833	rs59041913	5:131788135_T_C	Eosinophil percentage of granulocytes	EFO_0004586	27863252	European	-0.016	0.004	4.30E-05	173480	0	2	IVNT	9.19E-03
rs3749833	rs7713065	5:131788334_C_A	Self-reported asthma	EFO_0000270	UKBB	European	0.009	0.001	2.53E-25	337159	39049	1	risk diff	3.10E-21
rs3749833	rs7713065	5:131788334_C_A	Asthma	EFO_0000270	UKBB	European	0.009	0.001	2.92E-24	336782	38791	1	risk diff	2.89E-20
rs3749833	rs7713065	5:131788334_C_A	Sum eosinophil basophil counts	EFO_0004586	27863252	European	0.037	0.004	3.69E-21	173480	0	2	IVNT	2.50E-17
rs3749833	rs7713065	5:131788334_C_A	Eosinophil count	EFO_0004586	27863252	European	0.035	0.004	1.01E-19	173480	0	2	IVNT	5.63E-16
rs3749833	rs7713065	5:131788334_C_A	Height	EFO_0004339	UKBB	European	-0.017	0.002	2.23E-17	336474	0	1	IVNT	7.97E-14
rs3749833	rs7713065	5:131788334_C_A	Crohns disease	EFO_0000384	26192919	European	-0.218	0.028	5.59E-15	20883	5956	7	log OR	1.40E-11

sentinel	SNP	chr_bp_eff_other	trait	efo	pmid	ancestry	beta	se	p	n	n_cases	n_studies	unit	FDR
rs3749833	rs7713065	5:131788334_C_A	No blood clot, bronchitis, emphysema, asthma, rhinitis, eczema or allergy diagnosed by doctor	-	UKBB	European	-0.010	0.001	1.64E-14	336782	228530	1	risk diff	3.60E-11
rs3749833	rs7713065	5:131788334_C_A	Eosinophil percentage of white cells	EFO_0004586	27863252	European	0.028	0.004	4.30E-13	173480	0	2	IVNT	6.47E-10
rs3749833	rs7713065	5:131788334_C_A	Mean platelet volume	EFO_0004586	27863252	European	-0.028	0.004	7.27E-13	173480	0	2	IVNT	1.04E-09
rs3749833	rs7713065	5:131788334_C_A	Myeloid white cell count	EFO_0004586	27863252	European	0.028	0.004	1.27E-12	173480	0	2	IVNT	1.62E-09
rs3749833	rs7713065	5:131788334_C_A	Granulocyte count	EFO_0004586	27863252	European	0.027	0.004	3.54E-12	173480	0	2	IVNT	3.98E-09
rs3749833	rs7713065	5:131788334_C_A	Sum neutrophil eosinophil counts	EFO_0004586	27863252	European	0.026	0.004	1.22E-11	173480	0	2	IVNT	1.27E-08
rs3749833	rs7713065	5:131788334_C_A	Lung function FEV1FVC	EFO_0004713	28166213	European	-0.029	0.004	3.00E-11	-	-	-	unit increase	2.84E-08
rs3749833	rs7713065	5:131788334_C_A	Inflammatory bowel disease	EFO_0003767	26192919	European	-0.133	0.020	3.24E-11	34652	12882	15	log OR	3.04E-08
rs3749833	rs7713065	5:131788334_C_A	White blood cell count	EFO_0004586	27863252	European	0.025	0.004	3.45E-10	173480	0	2	IVNT	2.59E-07
rs3749833	rs7713065	5:131788334_C_A	Sum basophil neutrophil counts	EFO_0004586	27863252	European	0.024	0.004	5.12E-10	173480	0	2	IVNT	3.72E-07
rs3749833	rs7713065	5:131788334_C_A	Neutrophil count	EFO_0004586	27863252	European	0.024	0.004	1.58E-09	173480	0	2	IVNT	9.60E-07
rs3749833	rs7713065	5:131788334_C_A	Comparative height size at age 10	-	UKBB	European	-0.011	0.002	3.38E-09	332021	0	1	-	1.93E-06
rs3749833	rs7713065	5:131788334_C_A	Neutrophil percentage of granulocytes	EFO_0004586	27863252	European	-0.023	0.004	4.68E-09	173480	0	2	IVNT	2.52E-06
rs3749833	rs7713065	5:131788334_C_A	Eosinophil percentage of granulocytes	EFO_0004586	27863252	European	0.023	0.004	5.28E-09	173480	0	2	IVNT	2.80E-06
rs3749833	rs7713065	5:131788334_C_A	Allergic disease	EFO_0003785	29083406	European	0.036	0.006	6.44E-09	360838	180129	13	log OR	3.37E-06
rs3749833	rs7713065	5:131788334_C_A	Leg predicted mass left	-	UKBB	European	-0.010	0.002	2.51E-08	331253	0	1	IVNT	1.19E-05
rs3749833	rs7713065	5:131788334_C_A	Leg fat-free mass left	-	UKBB	European	-0.010	0.002	2.58E-08	331258	0	1	IVNT	1.21E-05
rs3749833	rs7713065	5:131788334_C_A	Leg fat-free mass right	-	UKBB	European	-0.010	0.002	2.92E-08	331285	0	1	IVNT	1.37E-05
rs3749833	rs7713065	5:131788334_C_A	Leg predicted mass right	-	UKBB	European	-0.010	0.002	3.01E-08	331285	0	1	IVNT	1.41E-05
rs3749833	rs7713065	5:131788334_C_A	Sitting height	EFO_0004339	UKBB	European	-0.012	0.002	6.39E-08	336172	0	1	IVNT	2.77E-05
rs3749833	rs7713065	5:131788334_C_A	Whole body water mass	-	UKBB	European	-0.009	0.002	1.01E-07	331315	0	1	IVNT	4.14E-05
rs3749833	rs7713065	5:131788334_C_A	Whole body fat-free mass	-	UKBB	European	-0.009	0.002	1.05E-07	331291	0	1	IVNT	4.28E-05
rs3749833	rs7713065	5:131788334_C_A	Lymphocyte percentage of white cells	EFO_0004586	27863252	European	-0.020	0.004	1.71E-07	173480	0	2	IVNT	6.68E-05
rs3749833	rs7713065	5:131788334_C_A	Basal metabolic rate	EFO_0007777	UKBB	European	-0.010	0.002	1.82E-07	331307	0	1	IVNT	7.09E-05
rs3749833	rs7713065	5:131788334_C_A	Forced expiratory volume in 1-second, best measure	EFO_0004314	UKBB	European	-0.013	0.003	2.86E-07	255492	0	1	IVNT	1.08E-04
rs3749833	rs7713065	5:131788334_C_A	Trunk fat-free mass	-	UKBB	European	-0.009	0.002	4.88E-07	331030	0	1	IVNT	1.75E-04
rs3749833	rs7713065	5:131788334_C_A	Trunk predicted mass	-	UKBB	European	-0.009	0.002	5.91E-07	330995	0	1	IVNT	2.08E-04
rs3749833	rs7713065	5:131788334_C_A	Doctor diagnosed asthma	EFO_0000270	UKBB	European	0.009	0.002	8.12E-07	83529	10589	1	risk diff	2.76E-04
rs3749833	rs7713065	5:131788334_C_A	Hayfever, allergic rhinitis or eczema	EFO_0003956; EFO_0005854; EFO_0000274	UKBB	European	0.006	0.001	1.27E-06	336782	77891	1	risk diff	4.08E-04
rs3749833	rs7713065	5:131788334_C_A	Treatment with seretide 50 evohaler	EFO_0007056	UKBB	European	0.001	0.000	1.32E-06	337159	3961	1	risk diff	4.19E-04
rs3749833	rs7713065	5:131788334_C_A	Monocyte count	EFO_0004586	27863252	European	0.018	0.004	4.67E-06	173480	0	2	IVNT	1.32E-03
rs3749833	rs7713065	5:131788334_C_A	Forced expiratory volume in 1-second	EFO_0004314	UKBB	European	-0.011	0.002	5.26E-06	307638	0	1	IVNT	1.47E-03
rs3749833	rs7713065	5:131788334_C_A	Arm predicted mass left	-	UKBB	European	-0.008	0.002	6.61E-06	331146	0	1	IVNT	1.81E-03
rs3749833	rs7713065	5:131788334_C_A	Self-reported nasal polyps	EFO_1000391	UKBB	European	0.001	0.000	6.69E-06	337159	1489	1	risk diff	1.82E-03
rs3749833	rs7713065	5:131788334_C_A	Hand grip strength right	EFO_0006941	UKBB	European	-0.009	0.002	7.48E-06	335842	0	1	IVNT	2.02E-03
rs3749833	rs7713065	5:131788334_C_A	Arm fat-free mass left	-	UKBB	European	-0.008	0.002	7.81E-06	331159	0	1	IVNT	2.08E-03
rs3749833	rs7713065	5:131788334_C_A	Wheeze or whistling in the chest in last year	EFO_0009715	UKBB	European	0.005	0.001	1.62E-05	331257	68531	1	risk diff	3.93E-03
rs3749833	rs7713065	5:131788334_C_A	Basophil count	EFO_0004586	27863252	European	0.016	0.004	1.84E-05	173480	0	2	IVNT	4.41E-03
rs3749833	rs7713065	5:131788334_C_A	Platelet count	EFO_0004586	27863252	European	0.017	0.004	1.85E-05	173480	0	2	IVNT	4.41E-03
rs3749833	rs7713065	5:131788334_C_A	Weight	EFO_0004338	UKBB	European	-0.010	0.002	2.11E-05	336227	0	1	IVNT	4.96E-03
rs3749833	rs7713065	5:131788334_C_A	Hand grip strength left	EFO_0006941	UKBB	European	-0.008	0.002	2.46E-05	335821	0	1	IVNT	5.69E-03
rs3749833	rs7713065	5:131788334_C_A	Treatment with vitamin a+d capsule	EFO_0007056	UKBB	European	0.000	0.000	2.90E-05	337159	53	1	risk diff	6.54E-03
rs3749833	rs10069798	5:131788421_A_T	Self-reported asthma	EFO_0000270	UKBB	European	0.008	0.001	2.75E-19	337159	39049	1	risk diff	1.28E-15
rs3749833	rs10069798	5:131788421_A_T	Asthma	EFO_0000270	UKBB	European	0.008	0.001	1.03E-17	336782	38791	1	risk diff	3.78E-14

sentinel	SNP	chr_bp_eff_other	trait	efo	pmid	ancestry	beta	se	p	n	n_cases	n_studies	unit	FDR
rs3749833	rs10069798	5:131788421_A_T	Sum eosinophil basophil counts	EFO_0004586	27863252	European	0.031	0.004	3.84E-14	173480	0	2	IVNT	7.85E-11
rs3749833	rs10069798	5:131788421_A_T	Crohns disease	EFO_0000384	26192919	European	-0.217	0.030	1.84E-13	20883	5956	7	log OR	3.16E-10
rs3749833	rs10069798	5:131788421_A_T	No blood clot, bronchitis, emphysema, asthma, rhinitis, eczema or allergy diagnosed by doctor	-	UKBB	European	-0.010	0.001	5.86E-13	336782	228530	1	risk diff	8.53E-10
rs3749833	rs10069798	5:131788421_A_T	Eosinophil count	EFO_0004586	27863252	European	0.029	0.004	8.78E-13	173480	0	2	IVNT	1.22E-09
rs3749833	rs10069798	5:131788421_A_T	Height	EFO_0004339	UKBB	European	-0.014	0.002	2.26E-11	336474	0	1	IVNT	2.20E-08
rs3749833	rs10069798	5:131788421_A_T	Mean platelet volume	EFO_0004586	27863252	European	-0.027	0.004	3.09E-11	173480	0	2	IVNT	2.91E-08
rs3749833	rs10069798	5:131788421_A_T	Myeloid white cell count	EFO_0004586	27863252	European	0.027	0.004	4.78E-11	173480	0	2	IVNT	4.30E-08
rs3749833	rs10069798	5:131788421_A_T	Granulocyte count	EFO_0004586	27863252	European	0.026	0.004	1.46E-10	173480	0	2	IVNT	1.16E-07
rs3749833	rs10069798	5:131788421_A_T	Sum neutrophil eosinophil counts	EFO_0004586	27863252	European	0.025	0.004	4.63E-10	173480	0	2	IVNT	3.41E-07
rs3749833	rs10069798	5:131788421_A_T	Inflammatory bowel disease	EFO_0003767	26192919	European	-0.129	0.021	9.16E-10	34652	12882	15	log OR	6.19E-07
rs3749833	rs10069798	5:131788421_A_T	Sum basophil neutrophil counts	EFO_0004586	27863252	European	0.024	0.004	6.47E-09	173480	0	2	IVNT	3.38E-06
rs3749833	rs10069798	5:131788421_A_T	White blood cell count	EFO_0004586	27863252	European	0.023	0.004	1.13E-08	173480	0	2	IVNT	5.68E-06
rs3749833	rs10069798	5:131788421_A_T	Neutrophil count	EFO_0004586	27863252	European	0.023	0.004	1.66E-08	173480	0	2	IVNT	8.10E-06
rs3749833	rs10069798	5:131788421_A_T	Eosinophil percentage of white cells	EFO_0004586	27863252	European	0.022	0.004	7.87E-08	173480	0	2	IVNT	3.31E-05
rs3749833	rs10069798	5:131788421_A_T	Lymphocyte percentage of white cells	EFO_0004586	27863252	European	-0.021	0.004	3.53E-07	173480	0	2	IVNT	1.31E-04
rs3749833	rs10069798	5:131788421_A_T	Allergic disease	EFO_0003785	29083406	European	0.032	0.007	1.04E-06	360838	180129	13	log OR	3.38E-04
rs3749833	rs10069798	5:131788421_A_T	Comparative height size at age 10	-	UKBB	European	-0.009	0.002	1.40E-06	332021	0	1	-	4.42E-04
rs3749833	rs10069798	5:131788421_A_T	Hayfever, allergic rhinitis or eczema	EFO_0003956; EFO_0005854; EFO_0000274	UKBB	European	0.006	0.001	1.42E-06	336782	77891	1	risk diff	4.46E-04
rs3749833	rs10069798	5:131788421_A_T	Treatment with vitamin a+d capsule	EFO_0007056	UKBB	European	0.000	0.000	1.08E-05	337159	53	1	risk diff	2.74E-03
rs3749833	rs10069798	5:131788421_A_T	Monocyte count	EFO_0004586	27863252	European	0.018	0.004	1.17E-05	173480	0	2	IVNT	2.95E-03
rs3749833	rs10069798	5:131788421_A_T	Self-reported nasal polyps	EFO_1000391	UKBB	European	0.001	0.000	2.28E-05	337159	1489	1	risk diff	5.33E-03
rs3749833	rs10069798	5:131788421_A_T	Platelet count	EFO_0004586	27863252	European	0.017	0.004	2.36E-05	173480	0	2	IVNT	5.48E-03
rs3749833	rs10069798	5:131788421_A_T	Neutrophil percentage of granulocytes	EFO_0004586	27863252	European	-0.017	0.004	2.65E-05	173480	0	2	IVNT	6.07E-03
rs3749833	rs10069798	5:131788421_A_T	Doctor diagnosed asthma	EFO_0000270	UKBB	European	0.008	0.002	3.64E-05	83529	10589	1	risk diff	7.93E-03
rs3749833	rs10069798	5:131788421_A_T	Height	EFO_0004339	25282103	European	-0.014	0.003	4.20E-05	253280	0	79	Z-score	9.00E-03
rs3749833	rs10069798	5:131788421_A_T	Eosinophil percentage of granulocytes	EFO_0004586	27863252	European	0.017	0.004	4.23E-05	173480	0	2	IVNT	9.06E-03
rs3749833	rs1023518	5:131793772_T_G	Self-reported asthma	EFO_0000270	UKBB	European	-0.009	0.001	1.87E-25	337159	39049	1	risk diff	2.54E-21
rs3749833	rs1023518	5:131793772_T_G	Asthma	EFO_0000270	UKBB	European	-0.009	0.001	2.22E-24	336782	38791	1	risk diff	2.29E-20
rs3749833	rs1023518	5:131793772_T_G	Sum eosinophil basophil counts	EFO_0004586	27863252	European	-0.037	0.004	2.55E-21	173480	0	2	IVNT	1.77E-17
rs3749833	rs1023518	5:131793772_T_G	Eosinophil count	EFO_0004586	27863252	European	-0.035	0.004	7.15E-20	173480	0	2	IVNT	4.09E-16
rs3749833	rs1023518	5:131793772_T_G	Height	EFO_0004339	UKBB	European	0.017	0.002	2.37E-17	336474	0	1	IVNT	8.25E-14
rs3749833	rs1023518	5:131793772_T_G	Crohns disease	EFO_0000384	23128233	European	NA	NA	3.20E-15	14342	6299	7	log OR	8.67E-12
rs3749833	rs1023518	5:131793772_T_G	Crohns disease	EFO_0000384	26192919	European	0.217	0.028	8.65E-15	20883	5956	7	log OR	2.10E-11
rs3749833	rs1023518	5:131793772_T_G	No blood clot, bronchitis, emphysema, asthma, rhinitis, eczema or allergy diagnosed by doctor	-	UKBB	European	0.010	0.001	1.61E-14	336782	228530	1	risk diff	3.58E-11
rs3749833	rs1023518	5:131793772_T_G	Eosinophil percentage of white cells	EFO_0004586	27863252	European	-0.028	0.004	3.36E-13	173480	0	2	IVNT	5.21E-10
rs3749833	rs1023518	5:131793772_T_G	Mean platelet volume	EFO_0004586	27863252	European	0.028	0.004	8.68E-13	173480	0	2	IVNT	1.21E-09
rs3749833	rs1023518	5:131793772_T_G	Myeloid white cell count	EFO_0004586	27863252	European	-0.028	0.004	1.34E-12	173480	0	2	IVNT	1.68E-09
rs3749833	rs1023518	5:131793772_T_G	Granulocyte count	EFO_0004586	27863252	European	-0.027	0.004	3.73E-12	173480	0	2	IVNT	4.18E-09
rs3749833	rs1023518	5:131793772_T_G	Sum neutrophil eosinophil counts	EFO_0004586	27863252	European	-0.026	0.004	1.29E-11	173480	0	2	IVNT	1.33E-08
rs3749833	rs1023518	5:131793772_T_G	Inflammatory bowel disease	EFO_0003767	26192919	European	0.130	0.020	1.03E-10	34652	12882	15	log OR	8.46E-08
rs3749833	rs1023518	5:131793772_T_G	White blood cell count	EFO_0004586	27863252	European	-0.025	0.004	3.46E-10	173480	0	2	IVNT	2.59E-07
rs3749833	rs1023518	5:131793772_T_G	Sum basophil neutrophil counts	EFO_0004586	27863252	European	-0.024	0.004	5.47E-10	173480	0	2	IVNT	3.96E-07
rs3749833	rs1023518	5:131793772_T_G	Asthma	EFO_0000270	29273806	European	-0.085	0.014	1.16E-09	127669	19954	56	log OR	7.46E-07
rs3749833	rs1023518	5:131793772_T_G	Asthma	EFO_0000270	29273806	Mixed	-0.079	0.013	1.61E-09	142486	23948	66	log OR	9.68E-07
rs3749833	rs1023518	5:131793772_T_G	Neutrophil count	EFO_0004586	27863252	European	-0.023	0.004	1.70E-09	173480	0	2	IVNT	1.01E-06

sentinel	SNP	chr_bp_eff_other	trait	efo	pmid	ancestry	beta	se	p	n	n_cases	n_studies	unit	FDR
rs3749833	rs1023518	5:131793772_T_G	Neutrophil percentage of granulocytes	EFO_0004586	27863252	European	0.023	0.004	3.51E-09	173480	0	2	IVNT	1.98E-06
rs3749833	rs1023518	5:131793772_T_G	Comparative height size at age 10	-	UKBB	European	0.011	0.002	3.88E-09	332021	0	1	-	2.14E-06
rs3749833	rs1023518	5:131793772_T_G	Eosinophil percentage of granulocytes	EFO_0004586	27863252	European	-0.023	0.004	4.25E-09	173480	0	2	IVNT	2.32E-06
rs3749833	rs1023518	5:131793772_T_G	Leg predicted mass left	-	UKBB	European	0.010	0.002	3.14E-08	331253	0	1	IVNT	1.46E-05
rs3749833	rs1023518	5:131793772_T_G	Leg fat-free mass left	-	UKBB	European	0.010	0.002	3.28E-08	331258	0	1	IVNT	1.52E-05
rs3749833	rs1023518	5:131793772_T_G	Leg fat-free mass right	-	UKBB	European	0.010	0.002	3.61E-08	331285	0	1	IVNT	1.65E-05
rs3749833	rs1023518	5:131793772_T_G	Leg predicted mass right	-	UKBB	European	0.010	0.002	3.75E-08	331285	0	1	IVNT	1.71E-05
rs3749833	rs1023518	5:131793772_T_G	Sitting height	EFO_0004339	UKBB	European	0.012	0.002	6.66E-08	336172	0	1	IVNT	2.88E-05
rs3749833	rs1023518	5:131793772_T_G	Whole body water mass	-	UKBB	European	0.009	0.002	1.23E-07	331315	0	1	IVNT	4.92E-05
rs3749833	rs1023518	5:131793772_T_G	Whole body fat-free mass	-	UKBB	European	0.009	0.002	1.28E-07	331291	0	1	IVNT	5.09E-05
rs3749833	rs1023518	5:131793772_T_G	Lymphocyte percentage of white cells	EFO_0004586	27863252	European	0.020	0.004	1.60E-07	173480	0	2	IVNT	6.28E-05
rs3749833	rs1023518	5:131793772_T_G	Allergic disease	EFO_0003785	29083406	European	-0.039	0.008	1.93E-07	360838	180129	13	log OR	7.48E-05
rs3749833	rs1023518	5:131793772_T_G	Basal metabolic rate	EFO_0007777	UKBB	European	0.009	0.002	2.20E-07	331307	0	1	IVNT	8.42E-05
rs3749833	rs1023518	5:131793772_T_G	Forced expiratory volume in 1-second, best measure	EFO_0004314	UKBB	European	0.013	0.003	2.77E-07	255492	0	1	IVNT	1.05E-04
rs3749833	rs1023518	5:131793772_T_G	Trunk fat-free mass	-	UKBB	European	0.009	0.002	5.92E-07	331030	0	1	IVNT	2.08E-04
rs3749833	rs1023518	5:131793772_T_G	Irritible bowel syndrome	EFO_0000555	18587394	European	NA	NA	6.12E-07	8059	-	-	-	2.14E-04
rs3749833	rs1023518	5:131793772_T_G	Doctor diagnosed asthma	EFO_0000270	UKBB	European	-0.009	0.002	6.78E-07	83529	10589	1	risk diff	2.33E-04
rs3749833	rs1023518	5:131793772_T_G	Trunk predicted mass	-	UKBB	European	0.009	0.002	7.16E-07	330995	0	1	IVNT	2.45E-04
rs3749833	rs1023518	5:131793772_T_G	Treatment with seretide 50 evohaler	EFO_0007056	UKBB	European	-0.001	0.000	1.07E-06	337159	3961	1	risk diff	3.47E-04
rs3749833	rs1023518	5:131793772_T_G	Hayfever, allergic rhinitis or eczema	EFO_0003956; EFO_0005854; EFO_0000274	UKBB	European	-0.006	0.001	1.24E-06	336782	77891	1	risk diff	3.98E-04
rs3749833	rs1023518	5:131793772_T_G	Height	EFO_0004339	25282103	European	0.016	0.003	2.10E-06	253280	0	79	Z-score	6.44E-04
rs3749833	rs1023518	5:131793772_T_G	Monocyte count	EFO_0004586	27863252	European	-0.018	0.004	3.92E-06	173480	0	2	IVNT	1.13E-03
rs3749833	rs1023518	5:131793772_T_G	Forced expiratory volume in 1-second	EFO_0004314	UKBB	European	0.011	0.002	5.24E-06	307638	0	1	IVNT	1.46E-03
rs3749833	rs1023518	5:131793772_T_G	Self-reported nasal polyps	EFO_1000391	UKBB	European	-0.001	0.000	6.55E-06	337159	1489	1	risk diff	1.79E-03
rs3749833	rs1023518	5:131793772_T_G	Arm predicted mass left	-	UKBB	European	0.008	0.002	7.38E-06	331146	0	1	IVNT	2.00E-03
rs3749833	rs1023518	5:131793772_T_G	Hand grip strength right	EFO_0006941	UKBB	European	0.009	0.002	7.46E-06	335842	0	1	IVNT	2.01E-03
rs3749833	rs1023518	5:131793772_T_G	Arm fat-free mass left	-	UKBB	European	0.008	0.002	9.11E-06	331159	0	1	IVNT	2.39E-03
rs3749833	rs1023518	5:131793772_T_G	Wheeze or whistling in the chest in last year	EFO_0009715	UKBB	European	-0.005	0.001	1.36E-05	331257	68531	1	risk diff	3.37E-03
rs3749833	rs1023518	5:131793772_T_G	Basophil count	EFO_0004586	27863252	European	-0.016	0.004	1.72E-05	173480	0	2	IVNT	4.12E-03
rs3749833	rs1023518	5:131793772_T_G	Platelet count	EFO_0004586	27863252	European	-0.017	0.004	1.87E-05	173480	0	2	IVNT	4.46E-03
rs3749833	rs1023518	5:131793772_T_G	Weight	EFO_0004338	UKBB	European	0.010	0.002	2.46E-05	336227	0	1	IVNT	5.68E-03
rs3749833	rs1023518	5:131793772_T_G	Asthma	EFO_0000270	20860503	European	-0.100	0.024	2.50E-05	26475	10365	24	log OR	5.76E-03
rs3749833	rs1023518	5:131793772_T_G	Asthma	EFO_0000270	20860503	European	NA	NA	2.50E-05	26475	-	-	-	5.76E-03
rs3749833	rs1023518	5:131793772_T_G	Hand grip strength left	EFO_0006941	UKBB	European	0.008	0.002	2.73E-05	335821	0	1	IVNT	6.23E-03
rs3749833	rs1023518	5:131793772_T_G	Treatment with vitamin a+d capsule	EFO_0007056	UKBB	European	0.000	0.000	2.93E-05	337159	53	1	risk diff	6.58E-03
rs3749833	rs3857440	5:131794069_A_G	Self-reported asthma	EFO_0000270	UKBB	European	0.009	0.001	2.67E-25	337159	39049	1	risk diff	3.12E-21
rs3749833	rs3857440	5:131794069_A_G	Asthma	EFO_0000270	UKBB	European	0.009	0.001	3.06E-24	336782	38791	1	risk diff	2.91E-20
rs3749833	rs3857440	5:131794069_A_G	Sum eosinophil basophil counts	EFO_0004586	27863252	European	0.037	0.004	2.46E-21	173480	0	2	IVNT	1.76E-17
rs3749833	rs3857440	5:131794069_A_G	Eosinophil count	EFO_0004586	27863252	European	0.036	0.004	6.17E-20	173480	0	2	IVNT	3.61E-16
rs3749833	rs3857440	5:131794069_A_G	Height	EFO_0004339	UKBB	European	-0.017	0.002	2.34E-17	336474	0	1	IVNT	8.25E-14
rs3749833	rs3857440	5:131794069_A_G	Crohns disease	EFO_0000384	23128233	European	NA	NA	4.00E-15	14342	6299	7	log OR	1.02E-11
rs3749833	rs3857440	5:131794069_A_G	Crohns disease	EFO_0000384	26192919	European	-0.216	0.028	9.04E-15	20883	5956	7	log OR	2.17E-11
rs3749833	rs3857440	5:131794069_A_G	No blood clot, bronchitis, emphysema, asthma, rhinitis, eczema or allergy diagnosed by doctor	-	UKBB	European	-0.010	0.001	2.06E-14	336782	228530	1	risk diff	4.39E-11
rs3749833	rs3857440	5:131794069_A_G	Eosinophil percentage of white cells	EFO_0004586	27863252	European	0.028	0.004	3.23E-13	173480	0	2	IVNT	5.06E-10
rs3749833	rs3857440	5:131794069_A_G	Mean platelet volume	EFO_0004586	27863252	European	-0.028	0.004	1.23E-12	173480	0	2	IVNT	1.59E-09

sentinel	SNP	chr_bp_eff_other	trait	efo	pmid	ancestry	beta	se	p	n	n_cases	n_studies	unit	FDR
rs3749833	rs3857440	5:131794069_A_G	Myeloid white cell count	EFO_0004586	27863252	European	0.028	0.004	1.24E-12	173480	0	2	IVNT	1.60E-09
rs3749833	rs3857440	5:131794069_A_G	Granulocyte count	EFO_0004586	27863252	European	0.027	0.004	3.04E-12	173480	0	2	IVNT	3.46E-09
rs3749833	rs3857440	5:131794069_A_G	Sum neutrophil eosinophil counts	EFO_0004586	27863252	European	0.027	0.004	1.05E-11	173480	0	2	IVNT	1.10E-08
rs3749833	rs3857440	5:131794069_A_G	Inflammatory bowel disease	EFO_0003767	26192919	European	-0.132	0.020	3.32E-11	34652	12882	15	log OR	3.07E-08
rs3749833	rs3857440	5:131794069_A_G	White blood cell count	EFO_0004586	27863252	European	0.025	0.004	2.83E-10	173480	0	2	IVNT	2.17E-07
rs3749833	rs3857440	5:131794069_A_G	Sum basophil neutrophil counts	EFO_0004586	27863252	European	0.024	0.004	4.49E-10	173480	0	2	IVNT	3.32E-07
rs3749833	rs3857440	5:131794069_A_G	Asthma	EFO_0000270	29273806	Mixed	0.080	0.013	1.04E-09	142486	23948	66	log OR	6.85E-07
rs3749833	rs3857440	5:131794069_A_G	Neutrophil count	EFO_0004586	27863252	European	0.024	0.004	1.40E-09	173480	0	2	IVNT	8.69E-07
rs3749833	rs3857440	5:131794069_A_G	Asthma	EFO_0000270	29273806	European	0.085	0.014	1.61E-09	127669	19954	56	log OR	9.68E-07
rs3749833	rs3857440	5:131794069_A_G	Neutrophil percentage of granulocytes	EFO_0004586	27863252	European	-0.023	0.004	3.51E-09	173480	0	2	IVNT	1.98E-06
rs3749833	rs3857440	5:131794069_A_G	Comparative height size at age 10	-	UKBB	European	-0.011	0.002	3.53E-09	332021	0	1	-	1.99E-06
rs3749833	rs3857440	5:131794069_A_G	Eosinophil percentage of granulocytes	EFO_0004586	27863252	European	0.023	0.004	4.15E-09	173480	0	2	IVNT	2.27E-06
rs3749833	rs3857440	5:131794069_A_G	Allergic disease	EFO_0003785	29083406	European	0.037	0.006	5.23E-09	360838	180129	13	log OR	2.78E-06
rs3749833	rs3857440	5:131794069_A_G	Leg predicted mass left	-	UKBB	European	-0.010	0.002	3.00E-08	331253	0	1	IVNT	1.40E-05
rs3749833	rs3857440	5:131794069_A_G	Leg fat-free mass left	-	UKBB	European	-0.010	0.002	3.10E-08	331258	0	1	IVNT	1.45E-05
rs3749833	rs3857440	5:131794069_A_G	Leg fat-free mass right	-	UKBB	European	-0.010	0.002	3.43E-08	331285	0	1	IVNT	1.58E-05
rs3749833	rs3857440	5:131794069_A_G	Leg predicted mass right	-	UKBB	European	-0.010	0.002	3.54E-08	331285	0	1	IVNT	1.62E-05
rs3749833	rs3857440	5:131794069_A_G	Sitting height	EFO_0004339	UKBB	European	-0.012	0.002	5.93E-08	336172	0	1	IVNT	2.60E-05
rs3749833	rs3857440	5:131794069_A_G	Whole body water mass	-	UKBB	European	-0.009	0.002	1.16E-07	331315	0	1	IVNT	4.67E-05
rs3749833	rs3857440	5:131794069_A_G	Whole body fat-free mass	-	UKBB	European	-0.009	0.002	1.20E-07	331291	0	1	IVNT	4.82E-05
rs3749833	rs3857440	5:131794069_A_G	Lymphocyte percentage of white cells	EFO_0004586	27863252	European	-0.020	0.004	1.65E-07	173480	0	2	IVNT	6.47E-05
rs3749833	rs3857440	5:131794069_A_G	Basal metabolic rate	EFO_0007777	UKBB	European	-0.010	0.002	2.05E-07	331307	0	1	IVNT	7.92E-05
rs3749833	rs3857440	5:131794069_A_G	Forced expiratory volume in 1-second, best measure	EFO_0004314	UKBB	European	-0.013	0.003	2.73E-07	255492	0	1	IVNT	1.03E-04
rs3749833	rs3857440	5:131794069_A_G	Trunk fat-free mass	-	UKBB	European	-0.009	0.002	5.64E-07	331030	0	1	IVNT	2.00E-04
rs3749833	rs3857440	5:131794069_A_G	Trunk predicted mass	-	UKBB	European	-0.009	0.002	6.80E-07	330995	0	1	IVNT	2.34E-04
rs3749833	rs3857440	5:131794069_A_G	Doctor diagnosed asthma	EFO_0000270	UKBB	European	0.009	0.002	7.40E-07	83529	10589	1	risk diff	2.52E-04
rs3749833	rs3857440	5:131794069_A_G	Height	EFO_0004339	25282103	European	-0.016	0.003	1.30E-06	253280	0	79	Z-score	4.16E-04
rs3749833	rs3857440	5:131794069_A_G	Treatment with seretide 50 evohaler	EFO_0007056	UKBB	European	0.001	0.000	1.31E-06	337159	3961	1	risk diff	4.19E-04
rs3749833	rs3857440	5:131794069_A_G	Hayfever, allergic rhinitis or eczema	EFO_0003956; EFO_0005854; EFO_0000274	UKBB	European	0.006	0.001	1.46E-06	336782	77891	1	risk diff	4.59E-04
rs3749833	rs3857440	5:131794069_A_G	Monocyte count	EFO_0004586	27863252	European	0.018	0.004	3.40E-06	173480	0	2	IVNT	9.90E-04
rs3749833	rs3857440	5:131794069_A_G	Forced expiratory volume in 1-second	EFO_0004314	UKBB	European	-0.011	0.002	5.24E-06	307638	0	1	IVNT	1.46E-03
rs3749833	rs3857440	5:131794069_A_G	Self-reported nasal polyps	EFO_1000391	UKBB	European	0.001	0.000	6.42E-06	337159	1489	1	risk diff	1.76E-03
rs3749833	rs3857440	5:131794069_A_G	Arm predicted mass left	-	UKBB	European	-0.008	0.002	6.77E-06	331146	0	1	IVNT	1.84E-03
rs3749833	rs3857440	5:131794069_A_G	Hand grip strength right	EFO_0006941	UKBB	European	-0.009	0.002	6.93E-06	335842	0	1	IVNT	1.89E-03
rs3749833	rs3857440	5:131794069_A_G	Arm fat-free mass left	-	UKBB	European	-0.008	0.002	8.29E-06	331159	0	1	IVNT	2.20E-03
rs3749833	rs3857440	5:131794069_A_G	Wheeze or whistling in the chest in last year	EFO_0009715	UKBB	European	0.005	0.001	1.48E-05	331257	68531	1	risk diff	3.61E-03
rs3749833	rs3857440	5:131794069_A_G	Basophil count	EFO_0004586	27863252	European	0.016	0.004	1.65E-05	173480	0	2	IVNT	3.98E-03
rs3749833	rs3857440	5:131794069_A_G	Platelet count	EFO_0004586	27863252	European	0.017	0.004	2.25E-05	173480	0	2	IVNT	5.28E-03
rs3749833	rs3857440	5:131794069_A_G	Weight	EFO_0004338	UKBB	European	-0.010	0.002	2.26E-05	336227	0	1	IVNT	5.28E-03
rs3749833	rs3857440	5:131794069_A_G	Hand grip strength left	EFO_0006941	UKBB	European	-0.008	0.002	2.58E-05	335821	0	1	IVNT	5.93E-03
rs3749833	rs3857440	5:131794069_A_G	Treatment with vitamin a+d capsule	EFO_0007056	UKBB	European	0.000	0.000	2.91E-05	337159	53	1	risk diff	6.54E-03
rs3749833	rs3857440	5:131794069_A_G	Age at menopause	EFO_0004704; EFO_0004703	26414677	European	-0.100	0.020	3.00E-05	69360	0	33	years	6.71E-03
rs3749833	rs3749833	5:131799626_C_T	Self-reported asthma	EFO_0000270	UKBB	European	-0.009	0.001	1.88E-25	337159	39049	1	risk diff	2.54E-21
rs3749833	rs3749833	5:131799626_C_T	Asthma	EFO_0000270	UKBB	European	-0.009	0.001	2.20E-24	336782	38791	1	risk diff	2.29E-20
rs3749833	rs3749833	5:131799626_C_T	Sum eosinophil basophil counts	EFO_0004586	27863252	European	-0.036	0.004	1.33E-19	173480	0	2	IVNT	7.14E-16

sentinel	SNP	chr_bp_eff_other	trait	efo	pmid	ancestry	beta	se	p	n	n_cases	n_studies	unit	FDR
rs3749833	rs3749833	5:131799626_C_T	Eosinophil count	EFO_0004586	27863252	European	-0.035	0.004	2.15E-18	173480	0	2	IVNT	9.05E-15
rs3749833	rs3749833	5:131799626_C_T	Height	EFO_0004339	UKBB	European	0.016	0.002	3.82E-17	336474	0	1	IVNT	1.29E-13
rs3749833	rs3749833	5:131799626_C_T	Mean platelet volume	EFO_0004586	27863252	European	0.032	0.004	5.67E-15	173480	0	2	IVNT	1.40E-11
rs3749833	rs3749833	5:131799626_C_T	Crohns disease	EFO_0000384	26192919	European	0.217	0.028	1.04E-14	20883	5956	7	log OR	2.39E-11
rs3749833	rs3749833	5:131799626_C_T	No blood clot, bronchitis, emphysema, asthma, rhinitis, eczema or allergy diagnosed by doctor	-	UKBB	European	0.010	0.001	1.12E-14	336782	228530	1	risk diff	2.52E-11
rs3749833	rs3749833	5:131799626_C_T	Myeloid white cell count	EFO_0004586	27863252	European	-0.029	0.004	3.12E-13	173480	0	2	IVNT	4.93E-10
rs3749833	rs3749833	5:131799626_C_T	Granulocyte count	EFO_0004586	27863252	European	-0.029	0.004	9.12E-13	173480	0	2	IVNT	1.25E-09
rs3749833	rs3749833	5:131799626_C_T	Eosinophil percentage of white cells	EFO_0004586	27863252	European	-0.028	0.004	1.97E-12	173480	0	2	IVNT	2.35E-09
rs3749833	rs3749833	5:131799626_C_T	Sum neutrophil eosinophil counts	EFO_0004586	27863252	European	-0.028	0.004	2.62E-12	173480	0	2	IVNT	3.04E-09
rs3749833	rs3749833	5:131799626_C_T	Allergic disease asthma hay fever or eczema	EFO_0003785	29083406	European	-0.038	0.006	3.00E-11	-	-	-	log OR	2.84E-08
rs3749833	rs3749833	5:131799626_C_T	Sum basophil neutrophil counts	EFO_0004586	27863252	European	-0.026	0.004	9.52E-11	173480	0	2	IVNT	7.91E-08
rs3749833	rs3749833	5:131799626_C_T	Inflammatory bowel disease	EFO_0003767	26192919	European	0.130	0.020	1.05E-10	34652	12882	15	log OR	8.66E-08
rs3749833	rs3749833	5:131799626_C_T	Neutrophil count	EFO_0004586	27863252	European	-0.025	0.004	3.29E-10	173480	0	2	IVNT	2.49E-07
rs3749833	rs3749833	5:131799626_C_T	Lymphocyte percentage of white cells	EFO_0004586	27863252	European	0.025	0.004	3.56E-10	173480	0	2	IVNT	2.66E-07
rs3749833	rs3749833	5:131799626_C_T	White blood cell count	EFO_0004586	27863252	European	-0.024	0.004	2.10E-09	173480	0	2	IVNT	1.23E-06
rs3749833	rs3749833	5:131799626_C_T	Allergic disease	EFO_0003785	29083406	European	-0.038	0.006	3.62E-09	360838	180129	13	log OR	2.03E-06
rs3749833	rs3749833	5:131799626_C_T	Comparative height size at age 10	-	UKBB	European	0.011	0.002	3.67E-09	332021	0	1	-	2.04E-06
rs3749833	rs3749833	5:131799626_C_T	Eosinophil percentage of granulocytes	EFO_0004586	27863252	European	-0.022	0.004	3.41E-08	173480	0	2	IVNT	1.57E-05
rs3749833	rs3749833	5:131799626_C_T	Leg predicted mass left	-	UKBB	European	0.010	0.002	3.79E-08	331253	0	1	IVNT	1.73E-05
rs3749833	rs3749833	5:131799626_C_T	Leg fat-free mass left	-	UKBB	European	0.010	0.002	3.89E-08	331258	0	1	IVNT	1.77E-05
rs3749833	rs3749833	5:131799626_C_T	Leg fat-free mass right	-	UKBB	European	0.010	0.002	4.28E-08	331285	0	1	IVNT	1.93E-05
rs3749833	rs3749833	5:131799626_C_T	Leg predicted mass right	-	UKBB	European	0.010	0.002	4.44E-08	331285	0	1	IVNT	1.99E-05
rs3749833	rs3749833	5:131799626_C_T	Neutrophil percentage of granulocytes	EFO_0004586	27863252	European	0.022	0.004	6.15E-08	173480	0	2	IVNT	2.68E-05
rs3749833	rs3749833	5:131799626_C_T	Sitting height	EFO_0004339	UKBB	European	0.011	0.002	7.32E-08	336172	0	1	IVNT	3.11E-05
rs3749833	rs3749833	5:131799626_C_T	Whole body water mass	-	UKBB	European	0.009	0.002	1.40E-07	331315	0	1	IVNT	5.53E-05
rs3749833	rs3749833	5:131799626_C_T	Whole body fat-free mass	-	UKBB	European	0.009	0.002	1.52E-07	331291	0	1	IVNT	5.97E-05
rs3749833	rs3749833	5:131799626_C_T	Basal metabolic rate	EFO_0007777	UKBB	European	0.009	0.002	2.48E-07	331307	0	1	IVNT	9.45E-05
rs3749833	rs3749833	5:131799626_C_T	Forced expiratory volume in 1-second, best measure	EFO_0004314	UKBB	European	0.013	0.003	3.05E-07	255492	0	1	IVNT	1.15E-04
rs3749833	rs3749833	5:131799626_C_T	Trunk fat-free mass	-	UKBB	European	0.009	0.002	7.20E-07	331030	0	1	IVNT	2.46E-04
rs3749833	rs3749833	5:131799626_C_T	Trunk predicted mass	-	UKBB	European	0.009	0.002	8.65E-07	330995	0	1	IVNT	2.91E-04
rs3749833	rs3749833	5:131799626_C_T	Doctor diagnosed asthma	EFO_0000270	UKBB	European	-0.009	0.002	9.74E-07	83529	10589	1	risk diff	3.21E-04
rs3749833	rs3749833	5:131799626_C_T	Hayfever, allergic rhinitis or eczema	EFO_0003956; EFO_0005854; EFO_0000274	UKBB	European	-0.006	0.001	1.06E-06	336782	77891	1	risk diff	3.45E-04
rs3749833	rs3749833	5:131799626_C_T	Treatment with seretide 50 evohaler	EFO_0007056	UKBB	European	-0.001	0.000	1.40E-06	337159	3961	1	risk diff	4.42E-04
rs3749833	rs3749833	5:131799626_C_T	Monocyte count	EFO_0004586	27863252	European	-0.018	0.004	4.96E-06	173480	0	2	IVNT	1.39E-03
rs3749833	rs3749833	5:131799626_C_T	Forced expiratory volume in 1-second	EFO_0004314	UKBB	European	0.011	0.002	5.91E-06	307638	0	1	IVNT	1.64E-03
rs3749833	rs3749833	5:131799626_C_T	Platelet count	EFO_0004586	27863252	European	-0.019	0.004	6.13E-06	173480	0	2	IVNT	1.69E-03
rs3749833	rs3749833	5:131799626_C_T	Arm predicted mass left	-	UKBB	European	0.008	0.002	7.39E-06	331146	0	1	IVNT	2.00E-03
rs3749833	rs3749833	5:131799626_C_T	Hand grip strength right	EFO_0006941	UKBB	European	0.009	0.002	7.61E-06	335842	0	1	IVNT	2.05E-03
rs3749833	rs3749833	5:131799626_C_T	Self-reported nasal polyps	EFO_1000391	UKBB	European	-0.001	0.000	8.28E-06	337159	1489	1	risk diff	2.20E-03
rs3749833	rs3749833	5:131799626_C_T	Arm fat-free mass left	-	UKBB	European	0.008	0.002	9.14E-06	331159	0	1	IVNT	2.40E-03
rs3749833	rs3749833	5:131799626_C_T	Wheeze or whistling in the chest in last year	EFO_0009715	UKBB	European	-0.005	0.001	1.46E-05	331257	68531	1	risk diff	3.58E-03
rs3749833	rs3749833	5:131799626_C_T	Weight	EFO_0004338	UKBB	European	0.010	0.002	2.41E-05	336227	0	1	IVNT	5.57E-03
rs3749833	rs3749833	5:131799626_C_T	Hand grip strength left	EFO_0006941	UKBB	European	0.008	0.002	2.70E-05	335821	0	1	IVNT	6.18E-03
rs3749833	rs3749833	5:131799626_C_T	Treatment with vitamin a+d capsule	EFO_0007056	UKBB	European	0.000	0.000	2.81E-05	337159	53	1	risk diff	6.38E-03
rs3749833	rs11748326	5:131799959_T_C	Self-reported asthma	EFO_0000270	UKBB	European	-0.009	0.001	1.27E-20	337159	39049	1	risk diff	7.81E-17

sentinel	SNP	chr_bp_eff_other	trait	efo	pmid	ancestry	beta	se	p	n	n_cases	n_studies	unit	FDR
rs3749833	rs11748326	5:131799959_T_C	Asthma	EFO_0000270	UKBB	European	-0.008	0.001	5.03E-19	336782	38791	1	risk diff	2.28E-15
rs3749833	rs11748326	5:131799959_T_C	Mean platelet volume	EFO_0004586	27863252	European	0.032	0.004	6.51E-14	173480	0	2	IVNT	1.23E-10
rs3749833	rs11748326	5:131799959_T_C	Sum eosinophil basophil counts	EFO_0004586	27863252	European	-0.031	0.004	1.64E-13	173480	0	2	IVNT	2.83E-10
rs3749833	rs11748326	5:131799959_T_C	No blood clot, bronchitis, emphysema, asthma, rhinitis, eczema or allergy diagnosed by doctor	-	UKBB	European	0.010	0.001	1.90E-13	336782	228530	1	risk diff	3.24E-10
rs3749833	rs11748326	5:131799959_T_C	Crohns disease	EFO_0000384	26192919	European	0.214	0.030	3.93E-13	20883	5956	7	log OR	6.03E-10
rs3749833	rs11748326	5:131799959_T_C	Eosinophil count	EFO_0004586	27863252	European	-0.029	0.004	2.12E-12	173480	0	2	IVNT	2.48E-09
rs3749833	rs11748326	5:131799959_T_C	Height	EFO_0004339	UKBB	European	0.014	0.002	3.21E-12	336474	0	1	IVNT	3.64E-09
rs3749833	rs11748326	5:131799959_T_C	Myeloid white cell count	EFO_0004586	27863252	European	-0.028	0.004	3.27E-11	173480	0	2	IVNT	3.06E-08
rs3749833	rs11748326	5:131799959_T_C	Granulocyte count	EFO_0004586	27863252	European	-0.027	0.004	1.09E-10	173480	0	2	IVNT	8.91E-08
rs3749833	rs11748326	5:131799959_T_C	Sum neutrophil eosinophil counts	EFO_0004586	27863252	European	-0.026	0.004	3.29E-10	173480	0	2	IVNT	2.49E-07
rs3749833	rs11748326	5:131799959_T_C	Lymphocyte percentage of white cells	EFO_0004586	27863252	European	0.026	0.004	6.37E-10	173480	0	2	IVNT	4.57E-07
rs3749833	rs11748326	5:131799959_T_C	Inflammatory bowel disease	EFO_0003767	26192919	European	0.126	0.021	1.90E-09	34652	12882	15	log OR	1.12E-06
rs3749833	rs11748326	5:131799959_T_C	Sum basophil neutrophil counts	EFO_0004586	27863252	European	-0.025	0.004	3.09E-09	173480	0	2	IVNT	1.78E-06
rs3749833	rs11748326	5:131799959_T_C	Neutrophil count	EFO_0004586	27863252	European	-0.024	0.004	9.83E-09	173480	0	2	IVNT	5.01E-06
rs3749833	rs11748326	5:131799959_T_C	Eosinophil percentage of white cells	EFO_0004586	27863252	European	-0.023	0.004	4.86E-08	173480	0	2	IVNT	2.16E-05
rs3749833	rs11748326	5:131799959_T_C	White blood cell count	EFO_0004586	27863252	European	-0.022	0.004	1.36E-07	173480	0	2	IVNT	5.39E-05
rs3749833	rs11748326	5:131799959_T_C	Allergic disease	EFO_0003785	29083406	European	-0.035	0.007	2.88E-07	360838	180129	13	log OR	1.09E-04
rs3749833	rs11748326	5:131799959_T_C	Hayfever, allergic rhinitis or eczema	EFO_0003956; EFO_0005854; EFO_0000274	UKBB	European	-0.006	0.001	3.71E-07	336782	77891	1	risk diff	1.37E-04
rs3749833	rs11748326	5:131799959_T_C	Comparative height size at age 10	-	UKBB	European	0.010	0.002	4.25E-07	332021	0	1	-	1.55E-04
rs3749833	rs11748326	5:131799959_T_C	Platelet count	EFO_0004586	27863252	European	-0.020	0.004	4.33E-06	173480	0	2	IVNT	1.23E-03
rs3749833	rs11748326	5:131799959_T_C	Monocyte count	EFO_0004586	27863252	European	-0.019	0.004	9.77E-06	173480	0	2	IVNT	2.53E-03
rs3749833	rs11748326	5:131799959_T_C	Leg fat-free mass left	-	UKBB	European	0.008	0.002	9.93E-06	331258	0	1	IVNT	2.57E-03
rs3749833	rs11748326	5:131799959_T_C	Leg predicted mass left	-	UKBB	European	0.008	0.002	1.08E-05	331253	0	1	IVNT	2.75E-03
rs3749833	rs11748326	5:131799959_T_C	Self-reported nasal polyps	EFO_1000391	UKBB	European	-0.001	0.000	1.51E-05	337159	1489	1	risk diff	3.69E-03
rs3749833	rs11748326	5:131799959_T_C	Doctor diagnosed asthma	EFO_0000270	UKBB	European	-0.008	0.002	1.61E-05	83529	10589	1	risk diff	3.90E-03
rs3749833	rs11748326	5:131799959_T_C	Leg fat-free mass right	-	UKBB	European	0.008	0.002	1.66E-05	331285	0	1	IVNT	4.00E-03
rs3749833	rs11748326	5:131799959_T_C	Leg predicted mass right	-	UKBB	European	0.008	0.002	1.82E-05	331285	0	1	IVNT	4.36E-03
rs3749833	rs11748326	5:131799959_T_C	Treatment with vitamin a+d capsule	EFO_0007056	UKBB	European	0.000	0.000	2.04E-05	337159	53	1	risk diff	4.82E-03
rs3749833	rs11748326	5:131799959_T_C	Neutrophil percentage of white cells	EFO_0004586	27863252	European	-0.017	0.004	3.32E-05	173480	0	2	IVNT	7.30E-03
rs3749833	rs11748326	5:131799959_T_C	Whole body fat-free mass	-	UKBB	European	0.007	0.002	3.94E-05	331291	0	1	IVNT	8.48E-03
rs3749833	rs11748326	5:131799959_T_C	Whole body water mass	-	UKBB	European	0.007	0.002	3.98E-05	331315	0	1	IVNT	8.55E-03
rs3749833	rs11748326	5:131799959_T_C	Sitting height	EFO_0004339	UKBB	European	0.009	0.002	4.40E-05	336172	0	1	IVNT	9.36E-03
rs3749833	rs10077785	5:131801158_T_C	Self-reported asthma	EFO_0000270	UKBB	European	-0.009	0.001	1.28E-20	337159	39049	1	risk diff	7.81E-17
rs3749833	rs10077785	5:131801158_T_C	Asthma	EFO_0000270	UKBB	European	-0.008	0.001	5.06E-19	336782	38791	1	risk diff	2.28E-15
rs3749833	rs10077785	5:131801158_T_C	Mean platelet volume	EFO_0004586	27863252	European	0.033	0.004	9.99E-15	173480	0	2	IVNT	2.32E-11
rs3749833	rs10077785	5:131801158_T_C	Crohns disease	EFO_0000384	23128233	European	NA	NA	4.20E-14	14342	6299	7	log OR	8.45E-11
rs3749833	rs10077785	5:131801158_T_C	Sum eosinophil basophil counts	EFO_0004586	27863252	European	-0.031	0.004	2.02E-13	173480	0	2	IVNT	3.40E-10
rs3749833	rs10077785	5:131801158_T_C	No blood clot, bronchitis, emphysema, asthma, rhinitis, eczema or allergy diagnosed by doctor	-	UKBB	European	0.010	0.001	2.06E-13	336782	228530	1	risk diff	3.44E-10
rs3749833	rs10077785	5:131801158_T_C	Crohns disease	EFO_0000384	26192919	European	0.214	0.030	4.01E-13	20883	5956	7	log OR	6.10E-10
rs3749833	rs10077785	5:131801158_T_C	Eosinophil count	EFO_0004586	27863252	European	-0.029	0.004	2.08E-12	173480	0	2	IVNT	2.44E-09
rs3749833	rs10077785	5:131801158_T_C	Height	EFO_0004339	UKBB	European	0.014	0.002	2.89E-12	336474	0	1	IVNT	3.32E-09
rs3749833	rs10077785	5:131801158_T_C	Myeloid white cell count	EFO_0004586	27863252	European	-0.028	0.004	2.84E-11	173480	0	2	IVNT	2.71E-08
rs3749833	rs10077785	5:131801158_T_C	Granulocyte count	EFO_0004586	27863252	European	-0.027	0.004	8.70E-11	173480	0	2	IVNT	7.27E-08
rs3749833	rs10077785	5:131801158_T_C	Sum neutrophil eosinophil counts	EFO_0004586	27863252	European	-0.027	0.004	2.52E-10	173480	0	2	IVNT	1.95E-07
rs3749833	rs10077785	5:131801158_T_C	Lymphocyte percentage of white cells	EFO_0004586	27863252	European	0.026	0.004	7.74E-10	173480	0	2	IVNT	5.39E-07

sentinel	SNP	chr_bp_eff_other	trait	efo	pmid	ancestry	beta	se	p	n	n_cases	n_studies	unit	FDR
rs3749833	rs10077785	5:131801158_T_C	Inflammatory bowel disease	EFO_0003767	26192919	European	0.126	0.021	1.82E-09	34652	12882	15	log OR	1.08E-06
rs3749833	rs10077785	5:131801158_T_C	Crohns disease	NCIT_C2965	17554300	European	NA	NA	2.25E-09	4806	-	-	-	1.32E-06
rs3749833	rs10077785	5:131801158_T_C	Sum basophil neutrophil counts	EFO_0004586	27863252	European	-0.025	0.004	2.42E-09	173480	0	2	IVNT	1.41E-06
rs3749833	rs10077785	5:131801158_T_C	Neutrophil count	EFO_0004586	27863252	European	-0.024	0.004	7.48E-09	173480	0	2	IVNT	3.87E-06
rs3749833	rs10077785	5:131801158_T_C	Asthma	EFO_0000270	29273806	European	-0.082	0.015	2.35E-08	127669	19954	56	log OR	1.12E-05
rs3749833	rs10077785	5:131801158_T_C	Eosinophil percentage of white cells	EFO_0004586	27863252	European	-0.023	0.004	4.82E-08	173480	0	2	IVNT	2.16E-05
rs3749833	rs10077785	5:131801158_T_C	Asthma	EFO_0000270	29273806	Mixed	-0.077	0.014	5.21E-08	142486	23948	66	log OR	2.30E-05
rs3749833	rs10077785	5:131801158_T_C	White blood cell count	EFO_0004586	27863252	European	-0.022	0.004	1.13E-07	173480	0	2	IVNT	4.59E-05
rs3749833	rs10077785	5:131801158_T_C	Allergic disease	EFO_0003785	29083406	European	-0.035	0.007	3.24E-07	360838	180129	13	log OR	1.21E-04
rs3749833	rs10077785	5:131801158_T_C	Comparative height size at age 10	-	UKBB	European	0.010	0.002	3.84E-07	332021	0	1	-	1.41E-04
rs3749833	rs10077785	5:131801158_T_C	Hayfever, allergic rhinitis or eczema	EFO_0003956; EFO_0005854; EFO_0000274	UKBB	European	-0.006	0.001	4.01E-07	336782	77891	1	risk diff	1.47E-04
rs3749833	rs10077785	5:131801158_T_C	Irritible bowel syndrome	EFO_0000555	18587394	European	NA	NA	2.99E-06	8059	-	-	-	8.86E-04
rs3749833	rs10077785	5:131801158_T_C	Platelet count	EFO_0004586	27863252	European	-0.020	0.004	3.35E-06	173480	0	2	IVNT	9.77E-04
rs3749833	rs10077785	5:131801158_T_C	Leg fat-free mass left	-	UKBB	European	0.008	0.002	9.37E-06	331258	0	1	IVNT	2.43E-03
rs3749833	rs10077785	5:131801158_T_C	Leg predicted mass left	-	UKBB	European	0.008	0.002	1.01E-05	331253	0	1	IVNT	2.61E-03
rs3749833	rs10077785	5:131801158_T_C	Doctor diagnosed asthma	EFO_0000270	UKBB	European	-0.008	0.002	1.50E-05	83529	10589	1	risk diff	3.66E-03
rs3749833	rs10077785	5:131801158_T_C	Leg fat-free mass right	-	UKBB	European	0.008	0.002	1.61E-05	331285	0	1	IVNT	3.91E-03
rs3749833	rs10077785	5:131801158_T_C	Leg predicted mass right	-	UKBB	European	0.008	0.002	1.77E-05	331285	0	1	IVNT	4.26E-03
rs3749833	rs10077785	5:131801158_T_C	Self-reported nasal polyps	EFO_1000391	UKBB	European	-0.001	0.000	1.86E-05	337159	1489	1	risk diff	4.45E-03
rs3749833	rs10077785	5:131801158_T_C	Monocyte count	EFO_0004586	27863252	European	-0.018	0.004	1.96E-05	173480	0	2	IVNT	4.65E-03
rs3749833	rs10077785	5:131801158_T_C	Treatment with vitamin a+d capsule	EFO_0007056	UKBB	European	0.000	0.000	2.05E-05	337159	53	1	risk diff	4.84E-03
rs3749833	rs10077785	5:131801158_T_C	Neutrophil percentage of white cells	EFO_0004586	27863252	European	-0.018	0.004	2.75E-05	173480	0	2	IVNT	6.26E-03
rs3749833	rs10077785	5:131801158_T_C	Whole body fat-free mass	-	UKBB	European	0.007	0.002	3.77E-05	331291	0	1	IVNT	8.13E-03
rs3749833	rs10077785	5:131801158_T_C	Whole body water mass	-	UKBB	European	0.007	0.002	3.77E-05	331315	0	1	IVNT	8.13E-03
rs3749833	rs10077785	5:131801158_T_C	Sitting height	EFO_0004339	UKBB	European	0.009	0.002	4.27E-05	336172	0	1	IVNT	9.13E-03
rs3749833	rs10067603	5:131803868_G_A	Self-reported asthma	EFO_0000270	UKBB	European	-0.008	0.001	1.12E-19	337159	39049	1	risk diff	6.14E-16
rs3749833	rs10067603	5:131803868_G_A	Asthma	EFO_0000270	UKBB	European	-0.008	0.001	4.09E-18	336782	38791	1	risk diff	1.67E-14
rs3749833	rs10067603	5:131803868_G_A	Mean platelet volume	EFO_0004586	27863252	European	0.033	0.004	2.04E-14	173480	0	2	IVNT	4.37E-11
rs3749833	rs10067603	5:131803868_G_A	Crohns disease	EFO_0000384	26192919	European	0.215	0.030	3.13E-13	20883	5956	7	log OR	4.93E-10
rs3749833	rs10067603	5:131803868_G_A	No blood clot, bronchitis, emphysema, asthma, rhinitis, eczema or allergy diagnosed by doctor	-	UKBB	European	0.010	0.001	1.14E-12	336782	228530	1	risk diff	1.50E-09
rs3749833	rs10067603	5:131803868_G_A	Sum eosinophil basophil counts	EFO_0004586	27863252	European	-0.029	0.004	4.27E-12	173480	0	2	IVNT	4.72E-09
rs3749833	rs10067603	5:131803868_G_A	Height	EFO_0004339	UKBB	European	0.014	0.002	1.29E-11	336474	0	1	IVNT	1.33E-08
rs3749833	rs10067603	5:131803868_G_A	Eosinophil count	EFO_0004586	27863252	European	-0.028	0.004	5.98E-11	173480	0	2	IVNT	5.22E-08
rs3749833	rs10067603	5:131803868_G_A	Myeloid white cell count	EFO_0004586	27863252	European	-0.028	0.004	8.72E-11	173480	0	2	IVNT	7.27E-08
rs3749833	rs10067603	5:131803868_G_A	Granulocyte count	EFO_0004586	27863252	European	-0.027	0.004	3.04E-10	173480	0	2	IVNT	2.31E-07
rs3749833	rs10067603	5:131803868_G_A	Sum neutrophil eosinophil counts	EFO_0004586	27863252	European	-0.026	0.004	7.75E-10	173480	0	2	IVNT	5.39E-07
rs3749833	rs10067603	5:131803868_G_A	Inflammatory bowel disease	EFO_0003767	26192919	European	0.127	0.021	1.33E-09	34652	12882	15	log OR	8.36E-07
rs3749833	rs10067603	5:131803868_G_A	Lymphocyte percentage of white cells	EFO_0004586	27863252	European	0.025	0.004	1.70E-09	173480	0	2	IVNT	1.01E-06
rs3749833	rs10067603	5:131803868_G_A	Sum basophil neutrophil counts	EFO_0004586	27863252	European	-0.025	0.004	6.72E-09	173480	0	2	IVNT	3.50E-06
rs3749833	rs10067603	5:131803868_G_A	Neutrophil count	EFO_0004586	27863252	European	-0.024	0.004	1.84E-08	173480	0	2	IVNT	8.89E-06
rs3749833	rs10067603	5:131803868_G_A	Asthma	EFO_0000270	29273806	European	-0.082	0.015	2.25E-08	127669	19954	56	log OR	1.08E-05
rs3749833	rs10067603	5:131803868_G_A	Asthma	EFO_0000270	29273806	Mixed	-0.078	0.014	5.01E-08	142486	23948	66	log OR	2.22E-05
rs3749833	rs10067603	5:131803868_G_A	White blood cell count	EFO_0004586	27863252	European	-0.022	0.004	3.41E-07	173480	0	2	IVNT	1.27E-04
rs3749833	rs10067603	5:131803868_G_A	Eosinophil percentage of white cells	EFO_0004586	27863252	European	-0.021	0.004	5.21E-07	173480	0	2	IVNT	1.87E-04
rs3749833	rs10067603	5:131803868_G_A	Comparative height size at age 10	-	UKBB	European	0.010	0.002	1.06E-06	332021	0	1	-	3.45E-04
rs3749833	rs10067603	5:131803868_G_A	Allergic disease	EFO_0003785	29083406	European	-0.032	0.007	2.34E-06	360838	180129	13	log OR	7.11E-04

sentinel	SNP	chr_bp_eff_other	trait	efo	pmid	ancestry	beta	se	p	n	n_cases	n_studies	unit	FDR
rs3749833	rs10067603	5:131803868_G_A	Hayfever, allergic rhinitis or eczema	EFO_0003956; EFO_0005854; EFO_0000274	UKBB	European	-0.006	0.001	3.09E-06	336782	77891	1	risk diff	9.11E-04
rs3749833	rs10067603	5:131803868_G_A	Platelet count	EFO_0004586	27863252	European	-0.019	0.004	7.82E-06	173480	0	2	IVNT	2.08E-03
rs3749833	rs10067603	5:131803868_G_A	Treatment with vitamin a+d capsule	EFO_0007056	UKBB	European	0.000	0.000	1.03E-05	337159	53	1	risk diff	2.65E-03
rs3749833	rs10067603	5:131803868_G_A	Monocyte count	EFO_0004586	27863252	European	-0.018	0.004	2.28E-05	173480	0	2	IVNT	5.33E-03
rs3749833	rs10067603	5:131803868_G_A	Self-reported nasal polyps	EFO_1000391	UKBB	European	-0.001	0.000	2.90E-05	337159	1489	1	risk diff	6.54E-03
rs3749833	rs10067603	5:131803868_G_A	Leg fat-free mass left	-	UKBB	European	0.008	0.002	3.20E-05	331258	0	1	IVNT	7.07E-03
rs3749833	rs10067603	5:131803868_G_A	Neutrophil percentage of white cells	EFO_0004586	27863252	European	-0.018	0.004	3.32E-05	173480	0	2	IVNT	7.30E-03
rs3749833	rs10067603	5:131803868_G_A	Leg predicted mass left	-	UKBB	European	0.008	0.002	3.54E-05	331253	0	1	IVNT	7.74E-03
rs3749833	rs10067603	5:131803868_G_A	Doctor diagnosed asthma	EFO_0000270	UKBB	European	-0.008	0.002	3.74E-05	83529	10589	1	risk diff	8.10E-03
rs3749833	rs10067603	5:131803868_G_A	Leg fat-free mass right	-	UKBB	European	0.008	0.002	3.89E-05	331285	0	1	IVNT	8.37E-03
rs3749833	rs10067603	5:131803868_G_A	Leg predicted mass right	-	UKBB	European	0.008	0.002	4.37E-05	331285	0	1	IVNT	9.33E-03
rs3749833	rs10067603	5:131803868_G_A	Lung function ratio of forced expiratory volume in 1 second FEV1 to forced vital capacity FVC FEV1FVC	NCIT_C38084; NCIT_C111359	20010835	European	NA	NA	4.54E-05	20890	-	-	-	9.61E-03
rs3749833	rs10060626	5:131803967_G_T	Self-reported asthma	EFO_0000270	UKBB	European	0.008	0.001	1.99E-19	337159	39049	1	risk diff	9.85E-16
rs3749833	rs10060626	5:131803967_G_T	Asthma	EFO_0000270	UKBB	European	0.008	0.001	7.09E-18	336782	38791	1	risk diff	2.73E-14
rs3749833	rs10060626	5:131803967_G_T	Mean platelet volume	EFO_0004586	27863252	European	-0.032	0.004	4.43E-14	173480	0	2	IVNT	8.84E-11
rs3749833	rs10060626	5:131803967_G_T	Crohns disease	EFO_0000384	26192919	European	-0.213	0.030	6.97E-13	20883	5956	7	log OR	1.00E-09
rs3749833	rs10060626	5:131803967_G_T	No blood clot, bronchitis, emphysema, asthma, rhinitis, eczema or allergy diagnosed by doctor	-	UKBB	European	-0.009	0.001	1.40E-12	336782	228530	1	risk diff	1.74E-09
rs3749833	rs10060626	5:131803967_G_T	Height	EFO_0004339	UKBB	European	-0.014	0.002	1.88E-11	336474	0	1	IVNT	1.86E-08
rs3749833	rs10060626	5:131803967_G_T	Sum eosinophil basophil counts	EFO_0004586	27863252	European	0.028	0.004	7.90E-11	173480	0	2	IVNT	6.64E-08
rs3749833	rs10060626	5:131803967_G_T	Myeloid white cell count	EFO_0004586	27863252	European	0.028	0.004	9.62E-11	173480	0	2	IVNT	7.96E-08
rs3749833	rs10060626	5:131803967_G_T	Granulocyte count	EFO_0004586	27863252	European	0.027	0.004	2.64E-10	173480	0	2	IVNT	2.03E-07
rs3749833	rs10060626	5:131803967_G_T	Sum neutrophil eosinophil counts	EFO_0004586	27863252	European	0.026	0.004	6.72E-10	173480	0	2	IVNT	4.78E-07
rs3749833	rs10060626	5:131803967_G_T	Lymphocyte percentage of white cells	EFO_0004586	27863252	European	-0.026	0.004	7.19E-10	173480	0	2	IVNT	5.06E-07
rs3749833	rs10060626	5:131803967_G_T	Eosinophil count	EFO_0004586	27863252	European	0.026	0.004	1.15E-09	173480	0	2	IVNT	7.42E-07
rs3749833	rs10060626	5:131803967_G_T	Inflammatory bowel disease	EFO_0003767	26192919	European	-0.128	0.021	1.32E-09	34652	12882	15	log OR	8.30E-07
rs3749833	rs10060626	5:131803967_G_T	Sum basophil neutrophil counts	EFO_0004586	27863252	European	0.025	0.004	4.56E-09	173480	0	2	IVNT	2.46E-06
rs3749833	rs10060626	5:131803967_G_T	Neutrophil count	EFO_0004586	27863252	European	0.024	0.004	1.25E-08	173480	0	2	IVNT	6.25E-06
rs3749833	rs10060626	5:131803967_G_T	White blood cell count	EFO_0004586	27863252	European	0.021	0.004	5.35E-07	173480	0	2	IVNT	1.91E-04
rs3749833	rs10060626	5:131803967_G_T	Comparative height size at age 10	-	UKBB	European	-0.010	0.002	8.85E-07	332021	0	1	-	2.97E-04
rs3749833	rs10060626	5:131803967_G_T	Allergic disease	EFO_0003785	29083406	European	0.032	0.007	3.12E-06	360838	180129	13	log OR	9.19E-04
rs3749833	rs10060626	5:131803967_G_T	Hayfever, allergic rhinitis or eczema	EFO_0003956; EFO_0005854; EFO_0000274	UKBB	European	0.006	0.001	3.21E-06	336782	77891	1	risk diff	9.42E-04
rs3749833	rs10060626	5:131803967_G_T	Eosinophil percentage of white cells	EFO_0004586	27863252	European	0.019	0.004	4.89E-06	173480	0	2	IVNT	1.38E-03
rs3749833	rs10060626	5:131803967_G_T	Platelet count	EFO_0004586	27863252	European	0.019	0.004	6.70E-06	173480	0	2	IVNT	1.83E-03
rs3749833	rs10060626	5:131803967_G_T	Neutrophil percentage of white cells	EFO_0004586	27863252	European	0.019	0.004	1.11E-05	173480	0	2	IVNT	2.81E-03
rs3749833	rs10060626	5:131803967_G_T	Treatment with vitamin a+d capsule	EFO_0007056	UKBB	European	0.000	0.000	2.36E-05	337159	53	1	risk diff	5.49E-03
rs3749833	rs10060626	5:131803967_G_T	Leg fat-free mass left	-	UKBB	European	-0.008	0.002	4.45E-05	331258	0	1	IVNT	9.47E-03
rs3749833	rs10039559	5:131804507_C_T	Self-reported asthma	EFO_0000270	UKBB	European	0.008	0.001	1.88E-19	337159	39049	1	risk diff	9.48E-16
rs3749833	rs10039559	5:131804507_C_T	Asthma	EFO_0000270	UKBB	European	0.008	0.001	6.68E-18	336782	38791	1	risk diff	2.61E-14
rs3749833	rs10039559	5:131804507_C_T	Mean platelet volume	EFO_0004586	27863252	European	-0.033	0.004	2.91E-14	173480	0	2	IVNT	6.15E-11
rs3749833	rs10039559	5:131804507_C_T	Crohns disease	EFO_0000384	23128233	European	NA	NA	6.60E-14	14342	6299	7	log OR	1.24E-10
rs3749833	rs10039559	5:131804507_C_T	Crohns disease	EFO_0000384	26192919	European	-0.216	0.030	2.75E-13	20883	5956	7	log OR	4.49E-10

sentinel	SNP	chr_bp_eff_other	trait	efo	pmid	ancestry	beta	se	p	n	n_cases	n_studies	unit	FDR
rs3749833	rs10039559	5:131804507_C_T	No blood clot, bronchitis, emphysema, asthma, rhinitis, eczema or allergy diagnosed by doctor	-	UKBB	European	-0.010	0.001	1.32E-12	336782	228530	1	risk diff	1.67E-09
rs3749833	rs10039559	5:131804507_C_T	Sum eosinophil basophil counts	EFO_0004586	27863252	European	0.029	0.004	4.89E-12	173480	0	2	IVNT	5.37E-09
rs3749833	rs10039559	5:131804507_C_T	Height	EFO_0004339	UKBB	European	-0.014	0.002	1.68E-11	336474	0	1	IVNT	1.68E-08
rs3749833	rs10039559	5:131804507_C_T	Eosinophil count	EFO_0004586	27863252	European	0.027	0.004	7.75E-11	173480	0	2	IVNT	6.56E-08
rs3749833	rs10039559	5:131804507_C_T	Myeloid white cell count	EFO_0004586	27863252	European	0.027	0.004	1.09E-10	173480	0	2	IVNT	8.91E-08
rs3749833	rs10039559	5:131804507_C_T	Granulocyte count	EFO_0004586	27863252	European	0.027	0.004	3.56E-10	173480	0	2	IVNT	2.66E-07
rs3749833	rs10039559	5:131804507_C_T	Sum neutrophil eosinophil counts	EFO_0004586	27863252	European	0.026	0.004	9.08E-10	173480	0	2	IVNT	6.16E-07
rs3749833	rs10039559	5:131804507_C_T	Inflammatory bowel disease	EFO_0003767	26192919	European	-0.127	0.021	1.25E-09	34652	12882	15	log OR	7.97E-07
rs3749833	rs10039559	5:131804507_C_T	Lymphocyte percentage of white cells	EFO_0004586	27863252	European	-0.025	0.004	1.57E-09	173480	0	2	IVNT	9.60E-07
rs3749833	rs10039559	5:131804507_C_T	Sum basophil neutrophil counts	EFO_0004586	27863252	European	0.025	0.004	7.78E-09	173480	0	2	IVNT	4.00E-06
rs3749833	rs10039559	5:131804507_C_T	Asthma	EFO_0000270	29273806	European	0.083	0.015	1.82E-08	127669	19954	56	log OR	8.82E-06
rs3749833	rs10039559	5:131804507_C_T	Neutrophil count	EFO_0004586	27863252	European	0.024	0.004	2.12E-08	173480	0	2	IVNT	1.02E-05
rs3749833	rs10039559	5:131804507_C_T	Asthma	EFO_0000270	29273806	Mixed	0.078	0.014	4.16E-08	142486	23948	66	log OR	1.88E-05
rs3749833	rs10039559	5:131804507_C_T	White blood cell count	EFO_0004586	27863252	European	0.021	0.004	4.03E-07	173480	0	2	IVNT	1.47E-04
rs3749833	rs10039559	5:131804507_C_T	Eosinophil percentage of white cells	EFO_0004586	27863252	European	0.021	0.004	5.84E-07	173480	0	2	IVNT	2.06E-04
rs3749833	rs10039559	5:131804507_C_T	Comparative height size at age 10	-	UKBB	European	-0.010	0.002	1.01E-06	332021	0	1	-	3.30E-04
rs3749833	rs10039559	5:131804507_C_T	Allergic disease	EFO_0003785	29083406	European	0.032	0.007	3.26E-06	360838	180129	13	log OR	9.55E-04
rs3749833	rs10039559	5:131804507_C_T	Hayfever, allergic rhinitis or eczema	EFO_0003956; EFO_0005854; EFO_0000274	UKBB	European	0.006	0.001	3.26E-06	336782	77891	1	risk diff	9.56E-04
rs3749833	rs10039559	5:131804507_C_T	Platelet count	EFO_0004586	27863252	European	0.019	0.004	9.00E-06	173480	0	2	IVNT	2.37E-03
rs3749833	rs10039559	5:131804507_C_T	Asthma	EFO_0000270	20860503	European	0.104	0.024	9.62E-06	26475	10365	24	log OR	2.49E-03
rs3749833	rs10039559	5:131804507_C_T	Asthma	EFO_0000270	20860503	European	NA	NA	9.62E-06	26475	-	-	-	2.49E-03
rs3749833	rs10039559	5:131804507_C_T	Treatment with vitamin a+d capsule	EFO_0007056	UKBB	European	0.000	0.000	1.04E-05	337159	53	1	risk diff	2.67E-03
rs3749833	rs10039559	5:131804507_C_T	Monocyte count	EFO_0004586	27863252	European	0.018	0.004	2.91E-05	173480	0	2	IVNT	6.54E-03
rs3749833	rs10039559	5:131804507_C_T	Neutrophil percentage of white cells	EFO_0004586	27863252	European	0.018	0.004	3.12E-05	173480	0	2	IVNT	6.91E-03
rs3749833	rs10039559	5:131804507_C_T	Self-reported nasal polyps	EFO_1000391	UKBB	European	0.001	0.000	3.71E-05	337159	1489	1	risk diff	8.04E-03
rs3749833	rs10039559	5:131804507_C_T	Leg fat-free mass left	-	UKBB	European	-0.008	0.002	4.04E-05	331258	0	1	IVNT	8.67E-03
rs3749833	rs10039559	5:131804507_C_T	Leg predicted mass left	-	UKBB	European	-0.008	0.002	4.41E-05	331253	0	1	IVNT	9.39E-03
rs3749833	rs10039559	5:131804507_C_T	Lung function ratio of forced expiratory volume in 1 second FEV1 to forced vital capacity FVC FEV1FVC	NCIT_C38084; NCIT_C111359	20010835	European	NA	NA	4.48E-05	20890	-	-	-	9.51E-03
rs3749833	rs10039559	5:131804507_C_T	Doctor diagnosed asthma	EFO_0000270	UKBB	European	0.008	0.002	4.52E-05	83529	10589	1	risk diff	9.58E-03
rs3749833	rs3846729	5:131806768_C_G	Self-reported asthma	EFO_0000270	UKBB	European	0.008	0.001	1.58E-19	337159	39049	1	risk diff	8.32E-16
rs3749833	rs3846729	5:131806768_C_G	Asthma	EFO_0000270	UKBB	European	0.008	0.001	5.69E-18	336782	38791	1	risk diff	2.25E-14
rs3749833	rs3846729	5:131806768_C_G	Mean platelet volume	EFO_0004586	27863252	European	-0.032	0.004	4.08E-14	173480	0	2	IVNT	8.27E-11
rs3749833	rs3846729	5:131806768_C_G	Crohns disease	EFO_0000384	26192919	European	-0.217	0.030	2.62E-13	20883	5956	7	log OR	4.30E-10
rs3749833	rs3846729	5:131806768_C_G	No blood clot, bronchitis, emphysema, asthma, rhinitis, eczema or allergy diagnosed by doctor	-	UKBB	European	-0.010	0.001	1.59E-12	336782	228530	1	risk diff	1.93E-09
rs3749833	rs3846729	5:131806768_C_G	Sum eosinophil basophil counts	EFO_0004586	27863252	European	0.029	0.004	4.06E-12	173480	0	2	IVNT	4.51E-09
rs3749833	rs3846729	5:131806768_C_G	Height	EFO_0004339	UKBB	European	-0.014	0.002	1.37E-11	336474	0	1	IVNT	1.39E-08
rs3749833	rs3846729	5:131806768_C_G	Eosinophil count	EFO_0004586	27863252	European	0.028	0.004	5.69E-11	173480	0	2	IVNT	5.00E-08
rs3749833	rs3846729	5:131806768_C_G	Myeloid white cell count	EFO_0004586	27863252	European	0.028	0.004	7.85E-11	173480	0	2	IVNT	6.63E-08
rs3749833	rs3846729	5:131806768_C_G	Granulocyte count	EFO_0004586	27863252	European	0.027	0.004	2.52E-10	173480	0	2	IVNT	1.95E-07
rs3749833	rs3846729	5:131806768_C_G	Sum neutrophil eosinophil counts	EFO_0004586	27863252	European	0.026	0.004	6.44E-10	173480	0	2	IVNT	4.60E-07
rs3749833	rs3846729	5:131806768_C_G	Inflammatory bowel disease	EFO_0003767	26192919	European	-0.128	0.021	1.25E-09	34652	12882	15	log OR	7.97E-07
rs3749833	rs3846729	5:131806768_C_G	Lymphocyte percentage of white cells	EFO_0004586	27863252	European	-0.026	0.004	1.35E-09	173480	0	2	IVNT	8.45E-07
rs3749833	rs3846729	5:131806768_C_G	Sum basophil neutrophil counts	EFO_0004586	27863252	European	0.025	0.004	5.57E-09	173480	0	2	IVNT	2.95E-06

sentinel	SNP	chr_bp_eff_other	trait	efo	pmid	ancestry	beta	se	p	n	n_cases	n_studies	unit	FDR
rs3749833	rs3846729	5:131806768_C_G	Neutrophil count	EFO_0004586	27863252	European	0.024	0.004	1.53E-08	173480	0	2	IVNT	7.48E-06
rs3749833	rs3846729	5:131806768_C_G	White blood cell count	EFO_0004586	27863252	European	0.022	0.004	3.14E-07	173480	0	2	IVNT	1.18E-04
rs3749833	rs3846729	5:131806768_C_G	Eosinophil percentage of white cells	EFO_0004586	27863252	European	0.021	0.004	4.79E-07	173480	0	2	IVNT	1.72E-04
rs3749833	rs3846729	5:131806768_C_G	Comparative height size at age 10	-	UKBB	European	-0.010	0.002	9.53E-07	332021	0	1	-	3.15E-04
rs3749833	rs3846729	5:131806768_C_G	Allergic disease	EFO_0003785	29083406	European	0.032	0.007	2.12E-06	360838	180129	13	log OR	6.50E-04
rs3749833	rs3846729	5:131806768_C_G	Hayfever, allergic rhinitis or eczema	EFO_0003956; EFO_0005854; EFO_0000274	UKBB	European	0.006	0.001	3.97E-06	336782	77891	1	risk diff	1.14E-03
rs3749833	rs3846729	5:131806768_C_G	Platelet count	EFO_0004586	27863252	European	0.019	0.004	8.53E-06	173480	0	2	IVNT	2.26E-03
rs3749833	rs3846729	5:131806768_C_G	Treatment with vitamin a+d capsule	EFO_0007056	UKBB	European	0.000	0.000	1.04E-05	337159	53	1	risk diff	2.67E-03
rs3749833	rs3846729	5:131806768_C_G	Height	EFO_0004339	25282103	European	-0.015	0.004	2.50E-05	253280	0	79	Z-score	5.76E-03
rs3749833	rs3846729	5:131806768_C_G	Neutrophil percentage of white cells	EFO_0004586	27863252	European	0.018	0.004	2.53E-05	173480	0	2	IVNT	5.80E-03
rs3749833	rs3846729	5:131806768_C_G	Leg fat-free mass left	-	UKBB	European	-0.008	0.002	2.83E-05	331258	0	1	IVNT	6.43E-03
rs3749833	rs3846729	5:131806768_C_G	Self-reported nasal polyps	EFO_1000391	UKBB	European	0.001	0.000	2.99E-05	337159	1489	1	risk diff	6.70E-03
rs3749833	rs3846729	5:131806768_C_G	Leg predicted mass left	-	UKBB	European	-0.008	0.002	3.08E-05	331253	0	1	IVNT	6.87E-03
rs3749833	rs3846729	5:131806768_C_G	Monocyte count	EFO_0004586	27863252	European	0.018	0.004	3.29E-05	173480	0	2	IVNT	7.24E-03
rs3749833	rs3846729	5:131806768_C_G	Leg fat-free mass right	-	UKBB	European	-0.008	0.002	3.64E-05	331285	0	1	IVNT	7.92E-03
rs3749833	rs3846729	5:131806768_C_G	Leg predicted mass right	-	UKBB	European	-0.008	0.002	4.04E-05	331285	0	1	IVNT	8.67E-03
rs3749833	rs3846729	5:131806768_C_G	Doctor diagnosed asthma	EFO_0000270	UKBB	European	0.008	0.002	4.60E-05	83529	10589	1	risk diff	9.73E-03
rs3749833	rs2548993	5:131808869_A_G	Self-reported asthma	EFO_0000270	UKBB	European	-0.009	0.001	8.92E-23	337159	39049	1	risk diff	8.20E-19
rs3749833	rs2548993	5:131808869_A_G	Asthma	EFO_0000270	UKBB	European	-0.008	0.001	7.46E-22	336782	38791	1	risk diff	5.82E-18
rs3749833	rs2548993	5:131808869_A_G	Sum eosinophil basophil counts	EFO_0004586	27863252	European	-0.037	0.004	3.65E-20	173480	0	2	IVNT	2.19E-16
rs3749833	rs2548993	5:131808869_A_G	Eosinophil count	EFO_0004586	27863252	European	-0.035	0.004	8.76E-19	173480	0	2	IVNT	3.82E-15
rs3749833	rs2548993	5:131808869_A_G	Crohns disease	EFO_0000384	23128233	European	NA	NA	4.60E-17	14342	6299	7	log OR	1.54E-13
rs3749833	rs2548993	5:131808869_A_G	Height	EFO_0004339	UKBB	European	0.016	0.002	6.43E-17	336474	0	1	IVNT	2.12E-13
rs3749833	rs2548993	5:131808869_A_G	Crohns disease	EFO_0000384	26192919	European	0.227	0.028	4.67E-16	20883	5956	7	log OR	1.41E-12
rs3749833	rs2548993	5:131808869_A_G	Mean platelet volume	EFO_0004586	27863252	European	0.033	0.004	4.75E-16	173480	0	2	IVNT	1.42E-12
rs3749833	rs2548993	5:131808869_A_G	Myeloid white cell count	EFO_0004586	27863252	European	-0.030	0.004	5.86E-14	173480	0	2	IVNT	1.16E-10
rs3749833	rs2548993	5:131808869_A_G	Granulocyte count	EFO_0004586	27863252	European	-0.030	0.004	1.36E-13	173480	0	2	IVNT	2.41E-10
rs3749833	rs2548993	5:131808869_A_G	No blood clot, bronchitis, emphysema, asthma, rhinitis, eczema or allergy diagnosed by doctor	-	UKBB	European	0.009	0.001	2.94E-13	336782	228530	1	risk diff	4.73E-10
rs3749833	rs2548993	5:131808869_A_G	Sum neutrophil eosinophil counts	EFO_0004586	27863252	European	-0.029	0.004	4.87E-13	173480	0	2	IVNT	7.12E-10
rs3749833	rs2548993	5:131808869_A_G	Eosinophil percentage of white cells	EFO_0004586	27863252	European	-0.028	0.004	1.56E-12	173480	0	2	IVNT	1.90E-09
rs3749833	rs2548993	5:131808869_A_G	Sum basophil neutrophil counts	EFO_0004586	27863252	European	-0.027	0.004	1.80E-11	173480	0	2	IVNT	1.79E-08
rs3749833	rs2548993	5:131808869_A_G	Inflammatory bowel disease	EFO_0003767	26192919	European	0.133	0.020	2.39E-11	34652	12882	15	log OR	2.32E-08
rs3749833	rs2548993	5:131808869_A_G	Neutrophil count	EFO_0004586	27863252	European	-0.026	0.004	7.38E-11	173480	0	2	IVNT	6.31E-08
rs3749833	rs2548993	5:131808869_A_G	Lymphocyte percentage of white cells	EFO_0004586	27863252	European	0.026	0.004	1.18E-10	173480	0	2	IVNT	9.55E-08
rs3749833	rs2548993	5:131808869_A_G	White blood cell count	EFO_0004586	27863252	European	-0.025	0.004	5.77E-10	173480	0	2	IVNT	4.16E-07
rs3749833	rs2548993	5:131808869_A_G	Asthma	EFO_0000270	29273806	Mixed	-0.080	0.013	1.60E-09	142486	23948	66	log OR	9.67E-07
rs3749833	rs2548993	5:131808869_A_G	Asthma	EFO_0000270	29273806	European	-0.085	0.014	3.57E-09	127669	19954	56	log OR	2.01E-06
rs3749833	rs2548993	5:131808869_A_G	Comparative height size at age 10	-	UKBB	European	0.011	0.002	6.90E-09	332021	0	1	-	3.58E-06
rs3749833	rs2548993	5:131808869_A_G	Eosinophil percentage of granulocytes	EFO_0004586	27863252	European	-0.022	0.004	4.24E-08	173480	0	2	IVNT	1.92E-05
rs3749833	rs2548993	5:131808869_A_G	Sitting height	EFO_0004339	UKBB	European	0.011	0.002	4.84E-08	336172	0	1	IVNT	2.16E-05
rs3749833	rs2548993	5:131808869_A_G	Neutrophil percentage of granulocytes	EFO_0004586	27863252	European	0.022	0.004	6.02E-08	173480	0	2	IVNT	2.64E-05
rs3749833	rs2548993	5:131808869_A_G	Leg predicted mass right	-	UKBB	European	0.009	0.002	1.19E-07	331285	0	1	IVNT	4.78E-05
rs3749833	rs2548993	5:131808869_A_G	Leg fat-free mass right	-	UKBB	European	0.009	0.002	1.27E-07	331285	0	1	IVNT	5.08E-05
rs3749833	rs2548993	5:131808869_A_G	Leg predicted mass left	-	UKBB	European	0.009	0.002	1.43E-07	331253	0	1	IVNT	5.65E-05
rs3749833	rs2548993	5:131808869_A_G	Leg fat-free mass left	-	UKBB	European	0.009	0.002	1.46E-07	331258	0	1	IVNT	5.78E-05
rs3749833	rs2548993	5:131808869_A_G	Height	EFO_0004339	25282103	European	0.018	0.003	1.70E-07	253280	0	79	Z-score	6.65E-05

sentinel	SNP	chr_bp_eff_other	trait	efo	pmid	ancestry	beta	se	p	n	n_cases	n_studies	unit	FDR
rs3749833	rs2548993	5:131808869_A_G	Whole body water mass	-	UKBB	European	0.009	0.002	1.83E-07	331315	0	1	IVNT	7.09E-05
rs3749833	rs2548993	5:131808869_A_G	Whole body fat-free mass	-	UKBB	European	0.009	0.002	2.52E-07	331291	0	1	IVNT	9.57E-05
rs3749833	rs2548993	5:131808869_A_G	Allergic disease	EFO_0003785	29083406	European	-0.033	0.006	3.34E-07	360838	180129	13	log OR	1.24E-04
rs3749833	rs2548993	5:131808869_A_G	Basal metabolic rate	EFO_0007777	UKBB	European	0.009	0.002	4.18E-07	331307	0	1	IVNT	1.52E-04
rs3749833	rs2548993	5:131808869_A_G	Trunk fat-free mass	-	UKBB	European	0.009	0.002	5.60E-07	331030	0	1	IVNT	1.99E-04
rs3749833	rs2548993	5:131808869_A_G	Trunk predicted mass	-	UKBB	European	0.009	0.002	6.19E-07	330995	0	1	IVNT	2.16E-04
rs3749833	rs2548993	5:131808869_A_G	Doctor diagnosed asthma	EFO_0000270	UKBB	European	-0.009	0.002	6.36E-07	83529	10589	1	risk diff	2.22E-04
rs3749833	rs2548993	5:131808869_A_G	Platelet count	EFO_0004586	27863252	European	-0.020	0.004	7.63E-07	173480	0	2	IVNT	2.60E-04
rs3749833	rs2548993	5:131808869_A_G	Asthma	EFO_0000270	20860503	European	-0.109	0.022	9.54E-07	26475	10365	24	log OR	3.15E-04
rs3749833	rs2548993	5:131808869_A_G	Asthma	EFO_0000270	20860503	European	NA	NA	9.54E-07	26475	-	-	-	3.15E-04
rs3749833	rs2548993	5:131808869_A_G	Forced expiratory volume in 1-second, best measure	EFO_0004314	UKBB	European	0.012	0.003	3.37E-06	255492	0	1	IVNT	9.83E-04
rs3749833	rs2548993	5:131808869_A_G	Monocyte count	EFO_0004586	27863252	European	-0.018	0.004	4.30E-06	173480	0	2	IVNT	1.23E-03
rs3749833	rs2548993	5:131808869_A_G	Hayfever, allergic rhinitis or eczema	EFO_0003956; EFO_0005854; EFO_0000274	UKBB	European	-0.005	0.001	4.77E-06	336782	77891	1	risk diff	1.35E-03
rs3749833	rs2548993	5:131808869_A_G	Self-reported nasal polyps	EFO_1000391	UKBB	European	-0.001	0.000	5.22E-06	337159	1489	1	risk diff	1.46E-03
rs3749833	rs2548993	5:131808869_A_G	Arm fat-free mass left	-	UKBB	European	0.008	0.002	7.67E-06	331159	0	1	IVNT	2.06E-03
rs3749833	rs2548993	5:131808869_A_G	Arm predicted mass left	-	UKBB	European	0.008	0.002	7.67E-06	331146	0	1	IVNT	2.06E-03
rs3749833	rs2548993	5:131808869_A_G	Treatment with seretide 50 evohaler	EFO_0007056	UKBB	European	-0.001	0.000	1.09E-05	337159	3961	1	risk diff	2.76E-03
rs3749833	rs2548993	5:131808869_A_G	Neutrophil percentage of white cells	EFO_0004586	27863252	European	-0.016	0.004	3.59E-05	173480	0	2	IVNT	7.82E-03
rs3749833	rs2548993	5:131808869_A_G	Arm fat-free mass right	-	UKBB	European	0.007	0.002	3.76E-05	331221	0	1	IVNT	8.12E-03
rs3749833	rs2057657	5:131809316_G_A	Self-reported asthma	EFO_0000270	UKBB	European	-0.008	0.001	2.06E-19	337159	39049	1	risk diff	9.99E-16
rs3749833	rs2057657	5:131809316_G_A	Asthma	EFO_0000270	UKBB	European	-0.008	0.001	7.72E-18	336782	38791	1	risk diff	2.92E-14
rs3749833	rs2057657	5:131809316_G_A	Mean platelet volume	EFO_0004586	27863252	European	0.032	0.004	3.17E-14	173480	0	2	IVNT	6.59E-11
rs3749833	rs2057657	5:131809316_G_A	Crohns disease	EFO_0000384	26192919	European	0.217	0.030	2.61E-13	20883	5956	7	log OR	4.30E-10
rs3749833	rs2057657	5:131809316_G_A	No blood clot, bronchitis, emphysema, asthma, rhinitis, eczema or allergy diagnosed by doctor	-	UKBB	European	0.010	0.001	1.50E-12	336782	228530	1	risk diff	1.84E-09
rs3749833	rs2057657	5:131809316_G_A	Sum eosinophil basophil counts	EFO_0004586	27863252	European	-0.029	0.004	4.02E-12	173480	0	2	IVNT	4.48E-09
rs3749833	rs2057657	5:131809316_G_A	Height	EFO_0004339	UKBB	European	0.014	0.002	1.21E-11	336474	0	1	IVNT	1.26E-08
rs3749833	rs2057657	5:131809316_G_A	Eosinophil count	EFO_0004586	27863252	European	-0.028	0.004	5.75E-11	173480	0	2	IVNT	5.04E-08
rs3749833	rs2057657	5:131809316_G_A	Myeloid white cell count	EFO_0004586	27863252	European	-0.028	0.004	8.60E-11	173480	0	2	IVNT	7.21E-08
rs3749833	rs2057657	5:131809316_G_A	Granulocyte count	EFO_0004586	27863252	European	-0.027	0.004	2.86E-10	173480	0	2	IVNT	2.19E-07
rs3749833	rs2057657	5:131809316_G_A	Sum neutrophil eosinophil counts	EFO_0004586	27863252	European	-0.026	0.004	7.27E-10	173480	0	2	IVNT	5.10E-07
rs3749833	rs2057657	5:131809316_G_A	Inflammatory bowel disease	EFO_0003767	26192919	European	0.128	0.021	1.25E-09	34652	12882	15	log OR	7.97E-07
rs3749833	rs2057657	5:131809316_G_A	Lymphocyte percentage of white cells	EFO_0004586	27863252	European	0.026	0.004	1.32E-09	173480	0	2	IVNT	8.30E-07
rs3749833	rs2057657	5:131809316_G_A	Sum basophil neutrophil counts	EFO_0004586	27863252	European	-0.025	0.004	6.44E-09	173480	0	2	IVNT	3.37E-06
rs3749833	rs2057657	5:131809316_G_A	Neutrophil count	EFO_0004586	27863252	European	-0.024	0.004	1.76E-08	173480	0	2	IVNT	8.54E-06
rs3749833	rs2057657	5:131809316_G_A	White blood cell count	EFO_0004586	27863252	European	-0.022	0.004	3.53E-07	173480	0	2	IVNT	1.31E-04
rs3749833	rs2057657	5:131809316_G_A	Eosinophil percentage of white cells	EFO_0004586	27863252	European	-0.021	0.004	4.62E-07	173480	0	2	IVNT	1.67E-04
rs3749833	rs2057657	5:131809316_G_A	Asthma	EFO_0000270	29273806	European	-0.076	0.015	5.34E-07	127669	19954	56	log OR	1.90E-04
rs3749833	rs2057657	5:131809316_G_A	Comparative height size at age 10	-	UKBB	European	0.010	0.002	8.71E-07	332021	0	1	-	2.92E-04
rs3749833	rs2057657	5:131809316_G_A	Asthma	EFO_0000270	29273806	Mixed	-0.072	0.015	1.18E-06	142486	23948	66	log OR	3.80E-04
rs3749833	rs2057657	5:131809316_G_A	Allergic disease	EFO_0003785	29083406	European	-0.032	0.007	2.10E-06	360838	180129	13	log OR	6.44E-04
rs3749833	rs2057657	5:131809316_G_A	Hayfever, allergic rhinitis or eczema	EFO_0003956; EFO_0005854; EFO_0000274	UKBB	European	-0.006	0.001	3.02E-06	336782	77891	1	risk diff	8.93E-04
rs3749833	rs2057657	5:131809316_G_A	Platelet count	EFO_0004586	27863252	European	-0.019	0.004	8.95E-06	173480	0	2	IVNT	2.36E-03
rs3749833	rs2057657	5:131809316_G_A	Treatment with vitamin a+d capsule	EFO_0007056	UKBB	European	0.000	0.000	1.06E-05	337159	53	1	risk diff	2.70E-03

sentinel	SNP	chr_bp_eff_other	trait	efo	pmid	ancestry	beta	se	p	n	n_cases	n_studies	unit	FDR
rs3749833	rs2057657	5:131809316_G_A	Height	EFO_0004339	25282103	European	0.015	0.004	1.30E-05	253280	0	79	Z-score	3.23E-03
rs3749833	rs2057657	5:131809316_G_A	Neutrophil percentage of white cells	EFO_0004586	27863252	European	-0.018	0.004	2.70E-05	173480	0	2	IVNT	6.18E-03
rs3749833	rs2057657	5:131809316_G_A	Leg fat-free mass left	-	UKBB	European	0.008	0.002	2.84E-05	331258	0	1	IVNT	6.43E-03
rs3749833	rs2057657	5:131809316_G_A	Self-reported nasal polyps	EFO_1000391	UKBB	European	-0.001	0.000	3.03E-05	337159	1489	1	risk diff	6.77E-03
rs3749833	rs2057657	5:131809316_G_A	Monocyte count	EFO_0004586	27863252	European	-0.018	0.004	3.08E-05	173480	0	2	IVNT	6.86E-03
rs3749833	rs2057657	5:131809316_G_A	Leg predicted mass left	-	UKBB	European	0.008	0.002	3.09E-05	331253	0	1	IVNT	6.88E-03
rs3749833	rs2057657	5:131809316_G_A	Leg fat-free mass right	-	UKBB	European	0.008	0.002	3.52E-05	331285	0	1	IVNT	7.69E-03
rs3749833	rs2057657	5:131809316_G_A	Leg predicted mass right	-	UKBB	European	0.008	0.002	3.98E-05	331285	0	1	IVNT	8.55E-03
rs3749833	rs2057657	5:131809316_G_A	Doctor diagnosed asthma	EFO_0000270	UKBB	European	-0.008	0.002	4.30E-05	83529	10589	1	risk diff	9.19E-03
rs9273410	rs9273410	6:32627250_A_C	Self-reported malabsorption or coeliac disease	EFO_0001060	UKBB	European	0.006	0.000	5.60E-291	337159	1452	1	risk diff	1.44E-285
rs9273410	rs9273410	6:32627250_A_C	Intestinal malabsorption	EFO_0009554	UKBB	European	0.002	0.000	4.22E-102	337199	624	1	risk diff	5.43E-97
rs9273410	rs9273410	6:32627250_A_C	Started insulin within one year diagnosis of diabetes	EFO_0000400	UKBB	European	0.079	0.004	1.59E-93	15397	1872	1	risk diff	1.36E-88
rs9273410	rs9273410	6:32627250_A_C	Treatment with insulin product	EFO_0007056	UKBB	European	0.005	0.000	6.70E-83	337159	3319	1	risk diff	4.31E-78
rs9273410	rs9273410	6:32627250_A_C	Asthma	EFO_0000270	UKBB	European	0.013	0.001	5.61E-58	336782	38791	1	risk diff	2.89E-53
rs9273410	rs9273410	6:32627250_A_C	Self-reported asthma	EFO_0000270	UKBB	European	0.013	0.001	4.28E-57	337159	39049	1	risk diff	1.84E-52
rs9273410	rs9273410	6:32627250_A_C	Treatment with insulin	EFO_0007056	UKBB	European	0.004	0.000	1.15E-46	180203	1377	1	risk diff	4.23E-42
rs9273410	rs9273410	6:32627250_A_C	Medication for cholesterol, blood pressure or diabetes: insulin	EFO_0007056	UKBB	European	0.006	0.000	1.12E-39	154702	2116	1	risk diff	3.19E-35
rs9273410	rs9273410	6:32627250_A_C	Self-reported hypothyroidism or myxoedema	EFO_0004705; EFO_1001055	UKBB	European	0.006	0.001	3.98E-29	337159	16376	1	risk diff	7.88E-25
rs9273410	rs9273410	6:32627250_A_C	Insulin-dependent diabetes mellitus	EFO_0001359	UKBB	European	0.001	0.000	5.33E-28	337199	431	1	risk diff	9.80E-24
rs9273410	rs9273410	6:32627250_A_C	Self-reported type 1 diabetes	EFO_0001359	UKBB	European	0.001	0.000	7.65E-28	337159	286	1	risk diff	1.31E-23
rs9273410	rs9273410	6:32627250_A_C	Treatment with ventolin 100micrograms inhaler	EFO_0007056	UKBB	European	0.005	0.000	6.13E-27	337159	9712	1	risk diff	9.85E-23
rs9273410	rs9273410	6:32627250_A_C	Self-reported hyperthyroidism or thyrotoxicosis	EFO_0004705; EFO_0004283	UKBB	European	0.002	0.000	2.56E-26	337159	2547	1	risk diff	3.88E-22
rs9273410	rs9273410	6:32627250_A_C	Treatment with levothyroxine sodium	EFO_0007056	UKBB	European	0.005	0.001	9.80E-23	337159	13717	1	risk diff	8.69E-19
rs9273410	rs9273410	6:32627250_A_C	Diabetes diagnosed by doctor	EFO_0000400	UKBB	European	0.005	0.001	6.15E-22	336473	16183	1	risk diff	4.94E-18
rs9273410	rs9273410	6:32627250_A_C	No blood clot, bronchitis, emphysema, asthma, rhinitis, eczema or allergy diagnosed by doctor	-	UKBB	European	-0.010	0.001	5.69E-18	336782	228530	1	risk diff	2.25E-14
rs9273410	rs9273410	6:32627250_A_C	Doctor diagnosed asthma	EFO_0000270	UKBB	European	0.015	0.002	2.07E-17	83529	10589	1	risk diff	7.49E-14
rs9273410	rs9273410	6:32627250_A_C	Forced expiratory volume in 1-second, best measure	EFO_0004314	UKBB	European	-0.020	0.002	3.42E-17	255492	0	1	IVNT	1.17E-13
rs9273410	rs9273410	6:32627250_A_C	Long-standing illness, disability or infirmity	-	UKBB	European	0.010	0.001	1.31E-16	329663	107123	1	risk diff	4.11E-13
rs9273410	rs9273410	6:32627250_A_C	Forced expiratory volume in 1-second, predicted percentage	EFO_0004314	UKBB	European	-0.037	0.005	1.59E-16	110423	0	1	IVNT	4.93E-13
rs9273410	rs9273410	6:32627250_A_C	Forced expiratory volume in 1-second	EFO_0004314	UKBB	European	-0.018	0.002	2.91E-16	307638	0	1	IVNT	8.93E-13
rs9273410	rs9273410	6:32627250_A_C	Self-reported diabetes	EFO_0000400	UKBB	European	0.004	0.001	2.83E-15	337159	13243	1	risk diff	7.93E-12
rs9273410	rs9273410	6:32627250_A_C	Eye problems or disorders: diabetes related eye disease	EFO_0009486	UKBB	European	0.005	0.001	3.43E-15	108817	2072	1	risk diff	9.19E-12
rs9273410	rs9273410	6:32627250_A_C	Self-reported psoriasis	EFO_0000676	UKBB	European	-0.002	0.000	5.16E-12	337159	3871	1	risk diff	5.65E-09
rs9273410	rs9273410	6:32627250_A_C	Illnesses of siblings: diabetes	EFO_0000400	UKBB	European	0.005	0.001	1.50E-11	261075	21747	1	risk diff	1.51E-08
rs9273410	rs9273410	6:32627250_A_C	Taking other prescription medications	EFO_0007056	UKBB	European	0.009	0.001	3.30E-11	336330	158113	1	risk diff	3.07E-08
rs9273410	rs9273410	6:32627250_A_C	Other disorders of pancreatic internal secretion	EFO_0009605	UKBB	European	0.000	0.000	5.93E-10	337199	228	1	risk diff	4.26E-07
rs9273410	rs9273410	6:32627250_A_C	Treatment with thyroxine product	EFO_0007056	UKBB	European	0.002	0.000	8.77E-10	337159	3917	1	risk diff	5.99E-07
rs9273410	rs9273410	6:32627250_A_C	Number of self-reported non-cancer illnesses	EFO_0009801	UKBB	European	0.013	0.002	1.25E-09	337159	0	1	-	7.97E-07
rs9273410	rs9273410	6:32627250_A_C	Self-reported ulcerative colitis	EFO_0000729	UKBB	European	-0.001	0.000	1.70E-09	337159	1795	1	risk diff	1.01E-06
rs9273410	rs9273410	6:32627250_A_C	Wheeze or whistling in the chest in last year	EFO_0009715	UKBB	European	0.006	0.001	4.69E-09	331257	68531	1	risk diff	2.52E-06
rs9273410	rs9273410	6:32627250_A_C	Red blood cell count	EFO_0004586	27863252	European	-0.021	0.004	7.62E-09	173480	0	2	IVNT	3.93E-06

sentinel	SNP	chr_bp_eff_other	trait	efo	pmid	ancestry	beta	se	p	n	n_cases	n_studies	unit	FDR
rs9273410	rs9273410	6:32627250_A_C	Treatment with seretide 50 evohaler	EFO_0007056	UKBB	European	0.002	0.000	1.09E-08	337159	3961	1	risk diff	5.49E-06
rs9273410	rs9273410	6:32627250_A_C	Peak expiratory flow	EFO_0009718	UKBB	European	-0.013	0.002	4.36E-08	307638	0	1	IVNT	1.96E-05
rs9273410	rs9273410	6:32627250_A_C	Hemoglobin concentration	EFO_0004586	27863252	European	-0.019	0.004	8.28E-08	173480	0	2	IVNT	3.45E-05
rs9273410	rs9273410	6:32627250_A_C	Self-reported multiple sclerosis	EFO_0003885	UKBB	European	-0.001	0.000	1.63E-07	337159	1228	1	risk diff	6.40E-05
rs9273410	rs9273410	6:32627250_A_C	Reticulocyte count	EFO_0004586	27863252	European	-0.019	0.004	2.47E-07	173480	0	2	IVNT	9.41E-05
rs9273410	rs9273410	6:32627250_A_C	Treatment with fludrocortisone	EFO_0007056	UKBB	European	0.000	0.000	2.84E-07	337159	148	1	risk diff	1.07E-04
rs9273410	rs9273410	6:32627250_A_C	Ulcerative colitis	EFO_0000729	UKBB	European	-0.001	0.000	6.88E-07	337199	1579	1	risk diff	2.36E-04
rs9273410	rs9273410	6:32627250_A_C	Asthma	EFO_0000270	UKBB	European	0.001	0.000	8.36E-07	337199	1235	1	risk diff	2.83E-04
rs9273410	rs9273410	6:32627250_A_C	Treatment with carbimazole	EFO_0007056	UKBB	European	0.000	0.000	1.01E-06	337159	275	1	risk diff	3.30E-04
rs9273410	rs9273410	6:32627250_A_C	Self-reported adrenocortical insufficiency or addisons disease	EFO_0009491	UKBB	European	0.000	0.000	1.02E-06	337159	141	1	risk diff	3.32E-04
rs9273410	rs9273410	6:32627250_A_C	Mean platelet volume	EFO_0004586	27863252	European	0.018	0.004	1.36E-06	173480	0	2	IVNT	4.31E-04
rs9273410	rs9273410	6:32627250_A_C	Lymphocyte count	EFO_0004586	27863252	European	-0.017	0.004	2.30E-06	173480	0	2	IVNT	7.01E-04
rs9273410	rs9273410	6:32627250_A_C	Hayfever, allergic rhinitis or eczema	EFO_0003956; EFO_0005854; EFO_0000274	UKBB	European	0.005	0.001	2.53E-06	336782	77891	1	risk diff	7.62E-04
rs9273410	rs9273410	6:32627250_A_C	Neutrophil percentage of white cells	EFO_0004586	27863252	European	0.017	0.004	2.92E-06	173480	0	2	IVNT	8.67E-04
rs9273410	rs9273410	6:32627250_A_C	Unspecified diabetes mellitus	EFO_0000400	UKBB	European	0.000	0.000	3.10E-06	337199	208	1	risk diff	9.13E-04
rs9273410	rs9273410	6:32627250_A_C	Treatment with ferrous sulphate	EFO_0007056	UKBB	European	0.001	0.000	4.53E-06	337159	1630	1	risk diff	1.28E-03
rs9273410	rs9273410	6:32627250_A_C	Mineral and other dietary supplements: calcium	EFO_0008111	UKBB	European	0.003	0.001	5.29E-06	336314	22047	1	risk diff	1.47E-03
rs9273410	rs9273410	6:32627250_A_C	Number of treatments or medications taken	EFO_0007056	UKBB	European	0.009	0.002	5.59E-06	337159	0	1	-	1.55E-03
rs9273410	rs9273410	6:32627250_A_C	Other anaemias	-	UKBB	European	0.001	0.000	7.20E-06	337199	2367	1	risk diff	1.95E-03
rs9273410	rs9273410	6:32627250_A_C	Lymphocyte percentage of white cells	EFO_0004586	27863252	European	-0.016	0.004	7.84E-06	173480	0	2	IVNT	2.09E-03
rs9273410	rs9273410	6:32627250_A_C	Granulocyte percentage of myeloid white cells	EFO_0004586	27863252	European	0.016	0.004	1.28E-05	173480	0	2	IVNT	3.20E-03
rs9273410	rs9273410	6:32627250_A_C	Mean corpuscular volume	EFO_0004586	27863252	European	0.015	0.004	1.80E-05	173480	0	2	IVNT	4.32E-03
rs9273410	rs9273410	6:32627250_A_C	Nasal polyp	EFO_1000391	UKBB	European	0.001	0.000	2.00E-05	337199	1637	1	risk diff	4.74E-03
rs9273410	rs9273410	6:32627250_A_C	Mean corpuscular hemoglobin concentration	EFO_0004586	27863252	European	-0.015	0.003	2.51E-05	173480	0	2	IVNT	5.77E-03
rs9273410	rs9273410	6:32627250_A_C	Self-reported diabetic eye disease	EFO_0009486	UKBB	European	0.000	0.000	2.99E-05	337159	635	1	risk diff	6.70E-03
rs9273410	rs9273410	6:32627250_A_C	Treatment with salbutamol	EFO_0007056	UKBB	European	0.001	0.000	3.10E-05	337159	3972	1	risk diff	6.88E-03
rs9273410	rs9273410	6:32627250_A_C	Used an inhaler for chest within last hour	-	UKBB	European	0.001	0.000	3.48E-05	309688	2245	1	risk diff	7.62E-03
rs9273410	rs9273410	6:32627250_A_C	Iron deficiency anaemia	-	UKBB	European	0.001	0.000	3.68E-05	337199	1775	1	risk diff	7.98E-03
rs9273410	rs9273410	6:32627250_A_C	Thyrotoxicosis	EFO_0009190	UKBB	European	0.000	0.000	3.75E-05	337199	299	1	risk diff	8.11E-03
rs2584662	rs2671654	17:47468011_G_A	Eosinophil count	EFO_0004586	27863252	European	0.027	0.004	6.49E-14	173480	0	2	IVNT	1.23E-10
rs2584662	rs2671654	17:47468011_G_A	Asthma	EFO_0000270	UKBB	European	0.006	0.001	8.65E-14	336782	38791	1	risk diff	1.57E-10
rs2584662	rs2671654	17:47468011_G_A	Vascular or heart problems diagnosed by doctor: none of the above	-	UKBB	European	-0.008	0.001	2.89E-13	336683	236530	1	risk diff	4.68E-10
rs2584662	rs2671654	17:47468011_G_A	Sum eosinophil basophil counts	EFO_0004586	27863252	European	0.026	0.004	4.62E-13	173480	0	2	IVNT	6.87E-10
rs2584662	rs2671654	17:47468011_G_A	Impedance of whole body	-	UKBB	European	-0.013	0.002	1.11E-12	331284	0	1	IVNT	1.48E-09
rs2584662	rs2671654	17:47468011_G_A	No blood clot, bronchitis, emphysema, asthma, rhinitis, eczema or allergy diagnosed by doctor	-	UKBB	European	-0.008	0.001	1.28E-12	336782	228530	1	risk diff	1.63E-09
rs2584662	rs2671654	17:47468011_G_A	Self-reported asthma	EFO_0000270	UKBB	European	0.006	0.001	1.83E-12	337159	39049	1	risk diff	2.20E-09
rs2584662	rs2671654	17:47468011_G_A	Impedance of leg right	-	UKBB	European	-0.015	0.002	7.57E-12	331301	0	1	IVNT	8.18E-09
rs2584662	rs2671654	17:47468011_G_A	Impedance of leg left	-	UKBB	European	-0.015	0.002	8.38E-12	331296	0	1	IVNT	8.99E-09
rs2584662	rs2671654	17:47468011_G_A	Eosinophil percentage of white cells	EFO_0004586	27863252	European	0.024	0.004	2.63E-11	173480	0	2	IVNT	2.53E-08
rs2584662	rs2671654	17:47468011_G_A	Vascular or heart problems diagnosed by doctor: high blood pressure	EFO_0004325	UKBB	European	0.007	0.001	1.27E-10	336683	91033	1	risk diff	1.02E-07
rs2584662	rs2671654	17:47468011_G_A	Eosinophil percentage of granulocytes	EFO_0004586	27863252	European	0.023	0.004	5.01E-10	173480	0	2	IVNT	3.65E-07
rs2584662	rs2671654	17:47468011_G_A	Wheeze or whistling in the chest in last year	EFO_0009715	UKBB	European	0.006	0.001	1.06E-09	331257	68531	1	risk diff	6.92E-07
rs2584662	rs2671654	17:47468011_G_A	Impedance of arm left	-	UKBB	European	-0.011	0.002	1.60E-09	331292	0	1	IVNT	9.67E-07

sentinel	SNP	chr_bp_eff_other	trait	efo	pmid	ancestry	beta	se	p	n	n_cases	n_studies	unit	FDR
rs2584662	rs2671654	17:47468011_G_A	Self-reported hypertension	EFO_0000537	UKBB	European	0.006	0.001	3.30E-09	337159	87690	1	risk diff	1.90E-06
rs2584662	rs2671654	17:47468011_G_A	Neutrophil percentage of granulocytes	EFO_0004586	27863252	European	-0.021	0.004	4.82E-09	173480	0	2	IVNT	2.58E-06
rs2584662	rs2671654	17:47468011_G_A	Height	EFO_0004339	UKBB	European	-0.010	0.002	1.28E-08	336474	0	1	IVNT	6.37E-06
rs2584662	rs2671654	17:47468011_G_A	Medication for cholesterol, blood pressure or diabetes: blood pressure medication	EFO_0007056	UKBB	European	0.009	0.002	1.28E-08	154702	38548	1	risk diff	6.40E-06
rs2584662	rs2671654	17:47468011_G_A	Impedance of arm right	-	UKBB	European	-0.009	0.002	4.50E-08	331279	0	1	IVNT	2.02E-05
rs2584662	rs2671654	17:47468011_G_A	Taking other prescription medications	EFO_0007056	UKBB	European	0.007	0.001	4.94E-08	336330	158113	1	risk diff	2.20E-05
rs2584662	rs2671654	17:47468011_G_A	Number of treatments or medications taken	EFO_0007056	UKBB	European	0.011	0.002	6.24E-08	337159	0	1	-	2.71E-05
rs2584662	rs2671654	17:47468011_G_A	Hayfever, allergic rhinitis or eczema	EFO_0003956; EFO_0005854; EFO_0000274	UKBB	European	0.006	0.001	8.76E-08	336782	77891	1	risk diff	3.62E-05
rs2584662	rs2671654	17:47468011_G_A	Number of self-reported non-cancer illnesses	EFO_0009801	UKBB	European	0.011	0.002	2.00E-07	337159	0	1	-	7.74E-05
rs2584662	rs2671654	17:47468011_G_A	Comparative body size at age 10	-	UKBB	European	0.009	0.002	4.67E-07	331693	0	1	-	1.68E-04
rs2584662	rs2671654	17:47468011_G_A	Treatment with ventolin 100micrograms inhaler	EFO_0007056	UKBB	European	0.002	0.000	7.29E-07	337159	9712	1	risk diff	2.49E-04
rs2584662	rs2671654	17:47468011_G_A	Coronary artery disease	EFO_0000378; EFO_0001645	29212778	Mixed	0.028	0.006	1.11E-06	547261	122733	2	log OR	3.57E-04
rs2584662	rs2671654	17:47468011_G_A	Atopic dermatitis	EFO_0000274	26482879	Mixed	0.084	0.018	3.39E-06	103066	18900	22	log OR	9.87E-04
rs2584662	rs2671654	17:47468011_G_A	Medication for cholesterol, blood pressure or diabetes: none of the above	EFO_0007056	UKBB	European	-0.008	0.002	3.77E-06	154702	103004	1	risk diff	1.09E-03
rs2584662	rs2671654	17:47468011_G_A	Coronary artery disease	EFO_0000378; EFO_0001645	29212778	Mixed	0.033	0.007	3.80E-06	296525	34541	1	log OR	1.09E-03
rs2584662	rs2671654	17:47468011_G_A	Allergic disease	EFO_0003785	29083406	European	0.026	0.006	7.13E-06	360838	180129	13	log OR	1.94E-03
rs2584662	rs2671654	17:47468011_G_A	Doctor diagnosed asthma	EFO_0000270	UKBB	European	0.007	0.002	2.75E-05	83529	10589	1	risk diff	6.26E-03
rs2584662	rs2671654	17:47468011_G_A	Overall health rating	-	UKBB	European	0.007	0.002	2.89E-05	336020	0	1	-	6.52E-03
rs2584662	rs2671654	17:47468011_G_A	Long-standing illness, disability or infirmity	-	UKBB	European	0.005	0.001	4.65E-05	329663	107123	1	risk diff	9.83E-03
rs2584662	rs2584663	17:47468147_A_G	Asthma	EFO_0000270	UKBB	European	0.006	0.001	1.02E-12	336782	38791	1	risk diff	1.38E-09
rs2584662	rs2584663	17:47468147_A_G	Self-reported asthma	EFO_0000270	UKBB	European	0.006	0.001	6.41E-12	337159	39049	1	risk diff	6.99E-09
rs2584662	rs2584663	17:47468147_A_G	Eosinophil count	EFO_0004586	27863252	European	0.025	0.004	1.06E-11	173480	0	2	IVNT	1.10E-08
rs2584662	rs2584663	17:47468147_A_G	Sum eosinophil basophil counts	EFO_0004586	27863252	European	0.025	0.004	1.91E-11	173480	0	2	IVNT	1.88E-08
rs2584662	rs2584663	17:47468147_A_G	Eosinophil percentage of white cells	EFO_0004586	27863252	European	0.024	0.004	7.75E-11	173480	0	2	IVNT	6.56E-08
rs2584662	rs2584663	17:47468147_A_G	No blood clot, bronchitis, emphysema, asthma, rhinitis, eczema or allergy diagnosed by doctor	-	UKBB	European	-0.008	0.001	1.97E-10	336782	228530	1	risk diff	1.53E-07
rs2584662	rs2584663	17:47468147_A_G	Eosinophil percentage of granulocytes	EFO_0004586	27863252	European	0.023	0.004	6.80E-10	173480	0	2	IVNT	4.82E-07
rs2584662	rs2584663	17:47468147_A_G	Neutrophil percentage of granulocytes	EFO_0004586	27863252	European	-0.023	0.004	9.48E-10	173480	0	2	IVNT	6.36E-07
rs2584662	rs2584663	17:47468147_A_G	Asthma	EFO_0000270	29273806	European	0.082	0.014	3.72E-09	127669	19954	56	log OR	2.06E-06
rs2584662	rs2584663	17:47468147_A_G	Vascular or heart problems diagnosed by doctor: none of the above	-	UKBB	European	-0.007	0.001	2.02E-08	336683	236530	1	risk diff	9.73E-06
rs2584662	rs2584663	17:47468147_A_G	Wheeze or whistling in the chest in last year	EFO_0009715	UKBB	European	0.006	0.001	7.39E-08	331257	68531	1	risk diff	3.14E-05
rs2584662	rs2584663	17:47468147_A_G	Asthma	EFO_0000270	29273806	Mixed	0.071	0.013	1.06E-07	142486	23948	66	log OR	4.32E-05
rs2584662	rs2584663	17:47468147_A_G	Hayfever, allergic rhinitis or eczema	EFO_0003956; EFO_0005854; EFO_0000274	UKBB	European	0.005	0.001	4.05E-07	336782	77891	1	risk diff	1.48E-04
rs2584662	rs2584663	17:47468147_A_G	Atopic dermatitis	EFO_0000274	26482879	Mixed	0.095	0.019	4.28E-07	103066	18900	22	log OR	1.55E-04
rs2584662	rs2584663	17:47468147_A_G	Number of treatments or medications taken	EFO_0007056	UKBB	European	0.010	0.002	4.98E-07	337159	0	1	-	1.79E-04
rs2584662	rs2584663	17:47468147_A_G	Comparative body size at age 10	-	UKBB	European	0.009	0.002	6.08E-07	331693	0	1	-	2.13E-04
rs2584662	rs2584663	17:47468147_A_G	Coronary artery disease	EFO_0000378; EFO_0001645	29212778	Mixed	0.029	0.006	1.30E-06	547261	122733	2	log OR	4.15E-04
rs2584662	rs2584663	17:47468147_A_G	Impedance of whole body	-	UKBB	European	-0.009	0.002	1.66E-06	331284	0	1	IVNT	5.14E-04
rs2584662	rs2584663	17:47468147_A_G	Taking other prescription medications	EFO_0007056	UKBB	European	0.006	0.001	2.83E-06	336330	158113	1	risk diff	8.44E-04

sentinel	SNP	chr_bp_eff_other	trait	efo	pmid	ancestry	beta	se	p	n	n_cases	n_studies	unit	FDR
rs2584662	rs2584663	17:47468147_A_G	Impedance of leg left	-	UKBB	European	-0.011	0.002	3.36E-06	331296	0	1	IVNT	9.80E-04
rs2584662	rs2584663	17:47468147_A_G	Impedance of leg right	-	UKBB	European	-0.011	0.002	4.36E-06	331301	0	1	IVNT	1.24E-03
rs2584662	rs2584663	17:47468147_A_G	Vascular or heart problems diagnosed by doctor: high blood pressure	EFO_0004325	UKBB	European	0.005	0.001	4.81E-06	336683	91033	1	risk diff	1.36E-03
rs2584662	rs2584663	17:47468147_A_G	Coronary artery disease	EFO_0000378; EFO_0001645	29212778	Mixed	0.033	0.007	7.70E-06	296525	34541	1	log OR	2.06E-03
rs2584662	rs2584663	17:47468147_A_G	Number of self-reported non-cancer illnesses	EFO_0009801	UKBB	European	0.009	0.002	1.24E-05	337159	0	1	-	3.10E-03
rs2584662	rs2584663	17:47468147_A_G	Medication for cholesterol, blood pressure or diabetes: blood pressure medication	EFO_0007056	UKBB	European	0.007	0.002	1.53E-05	154702	38548	1	risk diff	3.71E-03
rs2584662	rs2584663	17:47468147_A_G	Self-reported hypertension	EFO_0000537	UKBB	European	0.005	0.001	2.34E-05	337159	87690	1	risk diff	5.45E-03
rs2584662	rs2584663	17:47468147_A_G	Allergic disease	EFO_0003785	29083406	European	0.025	0.006	2.75E-05	360838	180129	13	log OR	6.26E-03
rs2584662	rs2584663	17:47468147_A_G	Mitral annular calcification	EFO_0005262	23388002	Mixed	NA	NA	3.10E-05	3795	568	2	log OR	6.88E-03
rs2584662	rs2584663	17:47468147_A_G	Mitral annular calcium	EFO_0005262	23388002	Mixed	NA	NA	3.10E-05	6942	-	-	-	6.88E-03
rs2584662	rs2412099	17:47469186_A_G	Asthma	EFO_0000270	UKBB	European	-0.006	0.001	8.13E-14	336782	38791	1	risk diff	1.51E-10
rs2584662	rs2412099	17:47469186_A_G	Eosinophil count	EFO_0004586	27863252	European	-0.027	0.004	1.53E-13	173480	0	2	IVNT	2.68E-10
rs2584662	rs2412099	17:47469186_A_G	Vascular or heart problems diagnosed by doctor: none of the above	-	UKBB	European	0.008	0.001	3.81E-13	336683	236530	1	risk diff	5.87E-10
rs2584662	rs2412099	17:47469186_A_G	Sum eosinophil basophil counts	EFO_0004586	27863252	European	-0.026	0.004	1.11E-12	173480	0	2	IVNT	1.48E-09
rs2584662	rs2412099	17:47469186_A_G	Impedance of whole body	-	UKBB	European	0.013	0.002	1.15E-12	331284	0	1	IVNT	1.52E-09
rs2584662	rs2412099	17:47469186_A_G	No blood clot, bronchitis, emphysema, asthma, rhinitis, eczema or allergy diagnosed by doctor	-	UKBB	European	0.008	0.001	1.20E-12	336782	228530	1	risk diff	1.56E-09
rs2584662	rs2412099	17:47469186_A_G	Self-reported asthma	EFO_0000270	UKBB	European	-0.006	0.001	1.76E-12	337159	39049	1	risk diff	2.12E-09
rs2584662	rs2412099	17:47469186_A_G	Impedance of leg right	-	UKBB	European	0.015	0.002	8.06E-12	331301	0	1	IVNT	8.68E-09
rs2584662	rs2412099	17:47469186_A_G	Impedance of leg left	-	UKBB	European	0.015	0.002	9.06E-12	331296	0	1	IVNT	9.63E-09
rs2584662	rs2412099	17:47469186_A_G	Eosinophil percentage of white cells	EFO_0004586	27863252	European	-0.024	0.004	5.66E-11	173480	0	2	IVNT	4.99E-08
rs2584662	rs2412099	17:47469186_A_G	Vascular or heart problems diagnosed by doctor: high blood pressure	EFO_0004325	UKBB	European	-0.007	0.001	1.56E-10	336683	91033	1	risk diff	1.24E-07
rs2584662	rs2412099	17:47469186_A_G	Wheeze or whistling in the chest in last year	EFO_0009715	UKBB	European	-0.006	0.001	1.02E-09	331257	68531	1	risk diff	6.74E-07
rs2584662	rs2412099	17:47469186_A_G	Eosinophil percentage of granulocytes	EFO_0004586	27863252	European	-0.022	0.004	1.12E-09	173480	0	2	IVNT	7.25E-07
rs2584662	rs2412099	17:47469186_A_G	Impedance of arm left	-	UKBB	European	0.011	0.002	1.62E-09	331292	0	1	IVNT	9.73E-07
rs2584662	rs2412099	17:47469186_A_G	Self-reported hypertension	EFO_0000537	UKBB	European	-0.006	0.001	4.12E-09	337159	87690	1	risk diff	2.25E-06
rs2584662	rs2412099	17:47469186_A_G	Neutrophil percentage of granulocytes	EFO_0004586	27863252	European	0.021	0.004	1.03E-08	173480	0	2	IVNT	5.22E-06
rs2584662	rs2412099	17:47469186_A_G	Medication for cholesterol, blood pressure or diabetes: blood pressure medication	EFO_0007056	UKBB	European	-0.009	0.002	1.16E-08	154702	38548	1	risk diff	5.82E-06
rs2584662	rs2412099	17:47469186_A_G	Height	EFO_0004339	UKBB	European	0.010	0.002	1.29E-08	336474	0	1	IVNT	6.41E-06
rs2584662	rs2412099	17:47469186_A_G	Impedance of arm right	-	UKBB	European	0.009	0.002	4.30E-08	331279	0	1	IVNT	1.94E-05
rs2584662	rs2412099	17:47469186_A_G	Taking other prescription medications	EFO_0007056	UKBB	European	-0.007	0.001	5.38E-08	336330	158113	1	risk diff	2.37E-05
rs2584662	rs2412099	17:47469186_A_G	Number of treatments or medications taken	EFO_0007056	UKBB	European	-0.011	0.002	7.32E-08	337159	0	1	-	3.11E-05
rs2584662	rs2412099	17:47469186_A_G	Hayfever, allergic rhinitis or eczema	EFO_0003956; EFO_0005854; EFO_0000274	UKBB	European	-0.006	0.001	8.36E-08	336782	77891	1	risk diff	3.48E-05
rs2584662	rs2412099	17:47469186_A_G	Number of self-reported non-cancer illnesses	EFO_0009801	UKBB	European	-0.011	0.002	2.09E-07	337159	0	1	-	8.04E-05
rs2584662	rs2412099	17:47469186_A_G	Comparative body size at age 10	-	UKBB	European	-0.009	0.002	4.60E-07	331693	0	1	-	1.66E-04
rs2584662	rs2412099	17:47469186_A_G	Treatment with ventolin 100micrograms inhaler	EFO_0007056	UKBB	European	-0.002	0.000	6.74E-07	337159	9712	1	risk diff	2.32E-04
rs2584662	rs2412099	17:47469186_A_G	Coronary artery disease	EFO_0000378; EFO_0001645	29212778	Mixed	-0.028	0.006	1.25E-06	547261	122733	2	log OR	4.02E-04
rs2584662	rs2412099	17:47469186_A_G	Medication for cholesterol, blood pressure or diabetes: none of the above	EFO_0007056	UKBB	European	0.008	0.002	3.51E-06	154702	103004	1	risk diff	1.02E-03

sentinel	SNP	chr_bp_eff_other	trait	efo	pmid	ancestry	beta	se	p	n	n_cases	n_studies	unit	FDR
rs2584662	rs2412099	17:47469186_A_G	Coronary artery disease	EFO_0000378; EFO_0001645	29212778	Mixed	-0.032	0.007	4.20E-06	296525	34541	1	log OR	1.20E-03
rs2584662	rs2412099	17:47469186_A_G	Allergic disease	EFO_0003785	29083406	European	-0.027	0.006	4.48E-06	360838	180129	13	log OR	1.27E-03
rs2584662	rs2412099	17:47469186_A_G	Atopic dermatitis	EFO_0000274	26482879	Mixed	-0.079	0.018	1.08E-05	103066	18900	22	log OR	2.75E-03
rs2584662	rs2412099	17:47469186_A_G	Doctor diagnosed asthma	EFO_0000270	UKBB	European	-0.007	0.002	2.91E-05	83529	10589	1	risk diff	6.54E-03
rs2584662	rs2412099	17:47469186_A_G	Overall health rating	-	UKBB	European	-0.007	0.002	3.33E-05	336020	0	1	-	7.32E-03
rs2584662	rs2412099	17:47469186_A_G	Long-standing illness, disability or infirmity	-	UKBB	European	-0.005	0.001	4.64E-05	329663	107123	1	risk diff	9.80E-03
rs2584662	rs9916130	17:47469229_G_C	Asthma	EFO_0000270	UKBB	European	0.006	0.001	1.10E-12	336782	38791	1	risk diff	1.48E-09
rs2584662	rs9916130	17:47469229_G_C	Self-reported asthma	EFO_0000270	UKBB	European	0.006	0.001	6.94E-12	337159	39049	1	risk diff	7.54E-09
rs2584662	rs9916130	17:47469229_G_C	Eosinophil count	EFO_0004586	27863252	European	0.026	0.004	9.66E-12	173480	0	2	IVNT	1.02E-08
rs2584662	rs9916130	17:47469229_G_C	Sum eosinophil basophil counts	EFO_0004586	27863252	European	0.025	0.004	1.73E-11	173480	0	2	IVNT	1.72E-08
rs2584662	rs9916130	17:47469229_G_C	Eosinophil percentage of white cells	EFO_0004586	27863252	European	0.024	0.004	7.63E-11	173480	0	2	IVNT	6.50E-08
rs2584662	rs9916130	17:47469229_G_C	No blood clot, bronchitis, emphysema, asthma, rhinitis, eczema or allergy diagnosed by doctor	-	UKBB	European	-0.008	0.001	1.82E-10	336782	228530	1	risk diff	1.43E-07
rs2584662	rs9916130	17:47469229_G_C	Eosinophil percentage of granulocytes	EFO_0004586	27863252	European	0.023	0.004	6.52E-10	173480	0	2	IVNT	4.65E-07
rs2584662	rs9916130	17:47469229_G_C	Neutrophil percentage of granulocytes	EFO_0004586	27863252	European	-0.023	0.004	9.11E-10	173480	0	2	IVNT	6.17E-07
rs2584662	rs9916130	17:47469229_G_C	Vascular or heart problems diagnosed by doctor: none of the above	-	UKBB	European	-0.006	0.001	2.56E-08	336683	236530	1	risk diff	1.21E-05
rs2584662	rs9916130	17:47469229_G_C	Wheeze or whistling in the chest in last year	EFO_0009715	UKBB	European	0.006	0.001	7.74E-08	331257	68531	1	risk diff	3.26E-05
rs2584662	rs9916130	17:47469229_G_C	Hayfever, allergic rhinitis or eczema	EFO_0003956; EFO_0005854; EFO_0000274	UKBB	European	0.005	0.001	3.91E-07	336782	77891	1	risk diff	1.44E-04
rs2584662	rs9916130	17:47469229_G_C	Atopic dermatitis	EFO_0000274	26482879	Mixed	0.095	0.019	4.11E-07	103066	18900	22	log OR	1.50E-04
rs2584662	rs9916130	17:47469229_G_C	Number of treatments or medications taken	EFO_0007056	UKBB	European	0.010	0.002	5.24E-07	337159	0	1	-	1.88E-04
rs2584662	rs9916130	17:47469229_G_C	Comparative body size at age 10	-	UKBB	European	0.009	0.002	6.72E-07	331693	0	1	-	2.32E-04
rs2584662	rs9916130	17:47469229_G_C	Coronary artery disease	EFO_0000378; EFO_0001645	29212778	Mixed	0.029	0.006	1.31E-06	547261	122733	2	log OR	4.18E-04
rs2584662	rs9916130	17:47469229_G_C	Impedance of whole body	-	UKBB	European	-0.009	0.002	1.63E-06	331284	0	1	IVNT	5.09E-04
rs2584662	rs9916130	17:47469229_G_C	Taking other prescription medications	EFO_0007056	UKBB	European	0.006	0.001	2.74E-06	336330	158113	1	risk diff	8.19E-04
rs2584662	rs9916130	17:47469229_G_C	Impedance of leg left	-	UKBB	European	-0.011	0.002	3.46E-06	331296	0	1	IVNT	1.00E-03
rs2584662	rs9916130	17:47469229_G_C	Impedance of leg right	-	UKBB	European	-0.011	0.002	4.39E-06	331301	0	1	IVNT	1.25E-03
rs2584662	rs9916130	17:47469229_G_C	Vascular or heart problems diagnosed by doctor: high blood pressure	EFO_0004325	UKBB	European	0.005	0.001	6.25E-06	336683	91033	1	risk diff	1.72E-03
rs2584662	rs9916130	17:47469229_G_C	Coronary artery disease	EFO_0000378; EFO_0001645	29212778	Mixed	0.033	0.007	6.50E-06	296525	34541	1	log OR	1.78E-03
rs2584662	rs9916130	17:47469229_G_C	Number of self-reported non-cancer illnesses	EFO_0009801	UKBB	European	0.009	0.002	1.38E-05	337159	0	1	-	3.39E-03
rs2584662	rs9916130	17:47469229_G_C	Medication for cholesterol, blood pressure or diabetes: blood pressure medication	EFO_0007056	UKBB	European	0.007	0.002	1.42E-05	154702	38548	1	risk diff	3.48E-03
rs2584662	rs9916130	17:47469229_G_C	Self-reported hypertension	EFO_0000537	UKBB	European	0.005	0.001	2.88E-05	337159	87690	1	risk diff	6.52E-03
rs2584662	rs9916130	17:47469229_G_C	Allergic disease	EFO_0003785	29083406	European	0.025	0.006	3.72E-05	360838	180129	13	log OR	8.07E-03
rs2584662	rs2584662	17:47470487_C_A	Eosinophil count	EFO_0004586	27863252	European	-0.027	0.004	8.30E-14	173480	0	2	IVNT	1.53E-10
rs2584662	rs2584662	17:47470487_C_A	Asthma	EFO_0000270	UKBB	European	-0.006	0.001	9.27E-14	336782	38791	1	risk diff	1.66E-10
rs2584662	rs2584662	17:47470487_C_A	Vascular or heart problems diagnosed by doctor: none of the above	-	UKBB	European	0.008	0.001	4.10E-13	336683	236530	1	risk diff	6.21E-10
rs2584662	rs2584662	17:47470487_C_A	Sum eosinophil basophil counts	EFO_0004586	27863252	European	-0.026	0.004	6.54E-13	173480	0	2	IVNT	9.46E-10
rs2584662	rs2584662	17:47470487_C_A	Impedance of whole body	-	UKBB	European	0.013	0.002	1.39E-12	331284	0	1	IVNT	1.74E-09
rs2584662	rs2584662	17:47470487_C_A	No blood clot, bronchitis, emphysema, asthma, rhinitis, eczema or allergy diagnosed by doctor	-	UKBB	European	0.008	0.001	1.40E-12	336782	228530	1	risk diff	1.74E-09
rs2584662	rs2584662	17:47470487_C_A	Self-reported asthma	EFO_0000270	UKBB	European	-0.006	0.001	1.99E-12	337159	39049	1	risk diff	2.36E-09

sentinel	SNP	chr_bp_eff_other	trait	efo	pmid	ancestry	beta	se	p	n	n_cases	n_studies	unit	FDR
rs2584662	rs2584662	17:47470487_C_A	Impedance of leg right	-	UKBB	European	0.015	0.002	8.55E-12	331301	0	1	IVNT	9.13E-09
rs2584662	rs2584662	17:47470487_C_A	Impedance of leg left	-	UKBB	European	0.015	0.002	9.92E-12	331296	0	1	IVNT	1.05E-08
rs2584662	rs2584662	17:47470487_C_A	Eosinophil percentage of white cells	EFO_0004586	27863252	European	-0.024	0.004	3.29E-11	173480	0	2	IVNT	3.06E-08
rs2584662	rs2584662	17:47470487_C_A	Vascular or heart problems diagnosed by doctor: high blood pressure	EFO_0004325	UKBB	European	-0.007	0.001	1.71E-10	336683	91033	1	risk diff	1.35E-07
rs2584662	rs2584662	17:47470487_C_A	Eosinophil percentage of granulocytes	EFO_0004586	27863252	European	-0.022	0.004	6.98E-10	173480	0	2	IVNT	4.92E-07
rs2584662	rs2584662	17:47470487_C_A	Wheeze or whistling in the chest in last year	EFO_0009715	UKBB	European	-0.006	0.001	1.09E-09	331257	68531	1	risk diff	7.10E-07
rs2584662	rs2584662	17:47470487_C_A	Impedance of arm left	-	UKBB	European	0.010	0.002	2.04E-09	331292	0	1	IVNT	1.20E-06
rs2584662	rs2584662	17:47470487_C_A	Self-reported hypertension	EFO_0000537	UKBB	European	-0.006	0.001	4.40E-09	337159	87690	1	risk diff	2.38E-06
rs2584662	rs2584662	17:47470487_C_A	Neutrophil percentage of granulocytes	EFO_0004586	27863252	European	0.021	0.004	7.30E-09	173480	0	2	IVNT	3.78E-06
rs2584662	rs2584662	17:47470487_C_A	Height	EFO_0004339	UKBB	European	0.010	0.002	1.33E-08	336474	0	1	IVNT	6.56E-06
rs2584662	rs2584662	17:47470487_C_A	Medication for cholesterol, blood pressure or diabetes: blood pressure medication	EFO_0007056	UKBB	European	-0.009	0.002	1.33E-08	154702	38548	1	risk diff	6.56E-06
rs2584662	rs2584662	17:47470487_C_A	Impedance of arm right	-	UKBB	European	0.009	0.002	5.04E-08	331279	0	1	IVNT	2.23E-05
rs2584662	rs2584662	17:47470487_C_A	Taking other prescription medications	EFO_0007056	UKBB	European	-0.007	0.001	6.04E-08	336330	158113	1	risk diff	2.64E-05
rs2584662	rs2584662	17:47470487_C_A	Number of treatments or medications taken	EFO_0007056	UKBB	European	-0.011	0.002	7.62E-08	337159	0	1	-	3.22E-05
rs2584662	rs2584662	17:47470487_C_A	Hayfever, allergic rhinitis or eczema	EFO_0003956; EFO_0005854; EFO_0000274	UKBB	European	-0.006	0.001	9.05E-08	336782	77891	1	risk diff	3.74E-05
rs2584662	rs2584662	17:47470487_C_A	Number of self-reported non-cancer illnesses	EFO_0009801	UKBB	European	-0.011	0.002	2.06E-07	337159	0	1	-	7.92E-05
rs2584662	rs2584662	17:47470487_C_A	Comparative body size at age 10	-	UKBB	European	-0.008	0.002	5.56E-07	331693	0	1	-	1.97E-04
rs2584662	rs2584662	17:47470487_C_A	Treatment with ventolin 100micrograms inhaler	EFO_0007056	UKBB	European	-0.002	0.000	6.59E-07	337159	9712	1	risk diff	2.28E-04
rs2584662	rs2584662	17:47470487_C_A	Coronary artery disease	EFO_0000378; EFO_0001645	29212778	Mixed	-0.028	0.006	1.88E-06	547261	122733	2	log OR	5.80E-04
rs2584662	rs2584662	17:47470487_C_A	Medication for cholesterol, blood pressure or diabetes: none of the above	EFO_0007056	UKBB	European	0.008	0.002	3.95E-06	154702	103004	1	risk diff	1.13E-03
rs2584662	rs2584662	17:47470487_C_A	Coronary artery disease	EFO_0000378; EFO_0001645	29212778	Mixed	-0.033	0.007	4.10E-06	296525	34541	1	log OR	1.17E-03
rs2584662	rs2584662	17:47470487_C_A	Atopic dermatitis	EFO_0000274	26482879	Mixed	-0.081	0.018	7.73E-06	103066	18900	22	log OR	2.07E-03
rs2584662	rs2584662	17:47470487_C_A	Allergic disease	EFO_0003785	29083406	European	-0.026	0.006	8.71E-06	360838	180129	13	log OR	2.30E-03
rs2584662	rs2584662	17:47470487_C_A	Doctor diagnosed asthma	EFO_0000270	UKBB	European	-0.007	0.002	2.97E-05	83529	10589	1	risk diff	6.67E-03
rs2584662	rs2584662	17:47470487_C_A	Overall health rating	-	UKBB	European	-0.007	0.002	3.57E-05	336020	0	1	-	7.78E-03
rs2584662	rs2584662	17:47470487_C_A	Long-standing illness, disability or infirmity	-	UKBB	European	-0.005	0.001	4.54E-05	329663	107123	1	risk diff	9.61E-03
rs2584662	rs2584661	17:47470534_A_G	Asthma	EFO_0000270	UKBB	European	0.006	0.001	2.98E-12	336782	38791	1	risk diff	3.41E-09
rs2584662	rs2584661	17:47470534_A_G	Self-reported asthma	EFO_0000270	UKBB	European	0.006	0.001	1.80E-11	337159	39049	1	risk diff	1.79E-08
rs2584662	rs2584661	17:47470534_A_G	Eosinophil count	EFO_0004586	27863252	European	0.025	0.004	2.95E-11	173480	0	2	IVNT	2.81E-08
rs2584662	rs2584661	17:47470534_A_G	Sum eosinophil basophil counts	EFO_0004586	27863252	European	0.025	0.004	3.61E-11	173480	0	2	IVNT	3.32E-08
rs2584662	rs2584661	17:47470534_A_G	Eosinophil percentage of white cells	EFO_0004586	27863252	European	0.024	0.004	2.63E-10	173480	0	2	IVNT	2.03E-07
rs2584662	rs2584661	17:47470534_A_G	No blood clot, bronchitis, emphysema, asthma, rhinitis, eczema or allergy diagnosed by doctor	-	UKBB	European	-0.007	0.001	5.38E-10	336782	228530	1	risk diff	3.90E-07
rs2584662	rs2584661	17:47470534_A_G	Eosinophil percentage of granulocytes	EFO_0004586	27863252	European	0.022	0.004	2.62E-09	173480	0	2	IVNT	1.53E-06
rs2584662	rs2584661	17:47470534_A_G	Neutrophil percentage of granulocytes	EFO_0004586	27863252	European	-0.022	0.004	3.32E-09	173480	0	2	IVNT	1.90E-06
rs2584662	rs2584661	17:47470534_A_G	Vascular or heart problems diagnosed by doctor: none of the above	-	UKBB	European	-0.006	0.001	3.33E-08	336683	236530	1	risk diff	1.54E-05
rs2584662	rs2584661	17:47470534_A_G	Wheeze or whistling in the chest in last year	EFO_0009715	UKBB	European	0.005	0.001	3.93E-07	331257	68531	1	risk diff	1.44E-04
rs2584662	rs2584661	17:47470534_A_G	Hayfever, allergic rhinitis or eczema	EFO_0003956; EFO_0005854; EFO_0000274	UKBB	European	0.005	0.001	6.32E-07	336782	77891	1	risk diff	2.20E-04
rs2584662	rs2584661	17:47470534_A_G	Comparative body size at age 10	-	UKBB	European	0.009	0.002	7.88E-07	331693	0	1	-	2.68E-04

sentinel	SNP	chr_bp_eff_other	trait	efo	pmid	ancestry	beta	se	p	n	n_cases	n_studies	unit	FDR
rs2584662	rs2584661	17:47470534_A_G	Number of treatments or medications taken	EFO_0007056	UKBB	European	0.010	0.002	9.12E-07	337159	0	1	-	3.05E-04
rs2584662	rs2584661	17:47470534_A_G	Coronary artery disease	EFO_0000378; EFO_0001645	29212778	Mixed	0.028	0.006	2.87E-06	547261	122733	2	log OR	8.54E-04
rs2584662	rs2584661	17:47470534_A_G	Impedance of whole body	-	UKBB	European	-0.009	0.002	2.93E-06	331284	0	1	IVNT	8.70E-04
rs2584662	rs2584661	17:47470534_A_G	Atopic dermatitis	EFO_0000274	26482879	Mixed	0.087	0.019	4.82E-06	103066	18900	22	log OR	1.36E-03
rs2584662	rs2584661	17:47470534_A_G	Impedance of leg left	-	UKBB	European	-0.011	0.002	5.09E-06	331296	0	1	IVNT	1.42E-03
rs2584662	rs2584661	17:47470534_A_G	Vascular or heart problems diagnosed by doctor: high blood pressure	EFO_0004325	UKBB	European	0.005	0.001	6.32E-06	336683	91033	1	risk diff	1.74E-03
rs2584662	rs2584661	17:47470534_A_G	Taking other prescription medications	EFO_0007056	UKBB	European	0.006	0.001	7.65E-06	336330	158113	1	risk diff	2.06E-03
rs2584662	rs2584661	17:47470534_A_G	Impedance of leg right	-	UKBB	European	-0.010	0.002	9.04E-06	331301	0	1	IVNT	2.38E-03
rs2584662	rs2584661	17:47470534_A_G	Coronary artery disease	EFO_0000378; EFO_0001645	29212778	Mixed	0.032	0.007	1.00E-05	296525	34541	1	log OR	2.58E-03
rs2584662	rs2584661	17:47470534_A_G	Number of self-reported non-cancer illnesses	EFO_0009801	UKBB	European	0.009	0.002	2.59E-05	337159	0	1	-	5.94E-03
rs2584662	rs2584661	17:47470534_A_G	Self-reported hypertension	EFO_0000537	UKBB	European	0.005	0.001	3.01E-05	337159	87690	1	risk diff	6.74E-03
rs2584662	rs2584661	17:47470534_A_G	Medication for cholesterol, blood pressure or diabetes: blood pressure medication	EFO_0007056	UKBB	European	0.007	0.002	3.12E-05	154702	38548	1	risk diff	6.91E-03
rs2584662	rs2671657	17:47473616_A_G	Asthma	EFO_0000270	UKBB	European	-0.006	0.001	9.18E-14	336782	38791	1	risk diff	1.65E-10
rs2584662	rs2671657	17:47473616_A_G	Eosinophil count	EFO_0004586	27863252	European	-0.027	0.004	2.19E-13	173480	0	2	IVNT	3.63E-10
rs2584662	rs2671657	17:47473616_A_G	Vascular or heart problems diagnosed by doctor: none of the above	-	UKBB	European	0.008	0.001	4.78E-13	336683	236530	1	risk diff	7.07E-10
rs2584662	rs2671657	17:47473616_A_G	Impedance of whole body	-	UKBB	European	0.013	0.002	1.22E-12	331284	0	1	IVNT	1.59E-09
rs2584662	rs2671657	17:47473616_A_G	No blood clot, bronchitis, emphysema, asthma, rhinitis, eczema or allergy diagnosed by doctor	-	UKBB	European	0.008	0.001	1.34E-12	336782	228530	1	risk diff	1.68E-09
rs2584662	rs2671657	17:47473616_A_G	Sum eosinophil basophil counts	EFO_0004586	27863252	European	-0.026	0.004	1.56E-12	173480	0	2	IVNT	1.90E-09
rs2584662	rs2671657	17:47473616_A_G	Self-reported asthma	EFO_0000270	UKBB	European	-0.006	0.001	2.03E-12	337159	39049	1	risk diff	2.40E-09
rs2584662	rs2671657	17:47473616_A_G	Impedance of leg right	-	UKBB	European	0.015	0.002	1.33E-11	331301	0	1	IVNT	1.35E-08
rs2584662	rs2671657	17:47473616_A_G	Impedance of leg left	-	UKBB	European	0.015	0.002	1.40E-11	331296	0	1	IVNT	1.41E-08
rs2584662	rs2671657	17:47473616_A_G	Eosinophil percentage of white cells	EFO_0004586	27863252	European	-0.024	0.004	6.13E-11	173480	0	2	IVNT	5.31E-08
rs2584662	rs2671657	17:47473616_A_G	Vascular or heart problems diagnosed by doctor: high blood pressure	EFO_0004325	UKBB	European	-0.007	0.001	1.94E-10	336683	91033	1	risk diff	1.52E-07
rs2584662	rs2671657	17:47473616_A_G	Wheeze or whistling in the chest in last year	EFO_0009715	UKBB	European	-0.006	0.001	7.90E-10	331257	68531	1	risk diff	5.48E-07
rs2584662	rs2671657	17:47473616_A_G	Eosinophil percentage of granulocytes	EFO_0004586	27863252	European	-0.022	0.004	9.28E-10	173480	0	2	IVNT	6.26E-07
rs2584662	rs2671657	17:47473616_A_G	Impedance of arm left	-	UKBB	European	0.011	0.002	1.47E-09	331292	0	1	IVNT	9.05E-07
rs2584662	rs2671657	17:47473616_A_G	Self-reported hypertension	EFO_0000537	UKBB	European	-0.006	0.001	4.32E-09	337159	87690	1	risk diff	2.35E-06
rs2584662	rs2671657	17:47473616_A_G	Neutrophil percentage of granulocytes	EFO_0004586	27863252	European	0.021	0.004	8.35E-09	173480	0	2	IVNT	4.27E-06
rs2584662	rs2671657	17:47473616_A_G	Medication for cholesterol, blood pressure or diabetes: blood pressure medication	EFO_0007056	UKBB	European	-0.009	0.002	1.20E-08	154702	38548	1	risk diff	6.01E-06
rs2584662	rs2671657	17:47473616_A_G	Height	EFO_0004339	UKBB	European	0.010	0.002	2.20E-08	336474	0	1	IVNT	1.06E-05
rs2584662	rs2671657	17:47473616_A_G	Impedance of arm right	-	UKBB	European	0.010	0.002	3.95E-08	331279	0	1	IVNT	1.79E-05
rs2584662	rs2671657	17:47473616_A_G	Number of treatments or medications taken	EFO_0007056	UKBB	European	-0.011	0.002	5.70E-08	337159	0	1	-	2.51E-05
rs2584662	rs2671657	17:47473616_A_G	Taking other prescription medications	EFO_0007056	UKBB	European	-0.007	0.001	5.82E-08	336330	158113	1	risk diff	2.56E-05
rs2584662	rs2671657	17:47473616_A_G	Hayfever, allergic rhinitis or eczema	EFO_0003956; EFO_0005854; EFO_0000274	UKBB	European	-0.006	0.001	9.21E-08	336782	77891	1	risk diff	3.80E-05
rs2584662	rs2671657	17:47473616_A_G	Number of self-reported non-cancer illnesses	EFO_0009801	UKBB	European	-0.011	0.002	2.11E-07	337159	0	1	-	8.11E-05
rs2584662	rs2671657	17:47473616_A_G	Comparative body size at age 10	-	UKBB	European	-0.008	0.002	5.33E-07	331693	0	1	-	1.90E-04
rs2584662	rs2671657	17:47473616_A_G	Treatment with ventolin 100micrograms inhaler	EFO_0007056	UKBB	European	-0.002	0.000	6.58E-07	337159	9712	1	risk diff	2.28E-04
rs2584662	rs2671657	17:47473616_A_G	Coronary artery disease	EFO_0000378; EFO_0001645	29212778	Mixed	-0.028	0.006	1.01E-06	547261	122733	2	log OR	3.30E-04

sentinel	SNP	chr_bp_eff_other	trait	efo	pmid	ancestry	beta	se	p	n	n_cases	n_studies	unit	FDR
rs2584662	rs2671657	17:47473616_A_G	Medication for cholesterol, blood pressure or diabetes: none of the above	EFO_0007056	UKBB	European	0.008	0.002	3.26E-06	154702	103004	1	risk diff	9.55E-04
rs2584662	rs2671657	17:47473616_A_G	Coronary artery disease	EFO_0000378; EFO_0001645	29212778	Mixed	-0.032	0.007	5.60E-06	296525	34541	1	log OR	1.55E-03
rs2584662	rs2671657	17:47473616_A_G	Allergic disease	EFO_0003785	29083406	European	-0.027	0.006	6.03E-06	360838	180129	13	log OR	1.67E-03
rs2584662	rs2671657	17:47473616_A_G	Atopic dermatitis	EFO_0000274	26482879	Mixed	-0.080	0.018	1.04E-05	103066	18900	22	log OR	2.67E-03
rs2584662	rs2671657	17:47473616_A_G	Doctor diagnosed asthma	EFO_0000270	UKBB	European	-0.007	0.002	2.98E-05	83529	10589	1	risk diff	6.68E-03
rs2584662	rs2671657	17:47473616_A_G	Long-standing illness, disability or infirmity	-	UKBB	European	-0.005	0.001	3.44E-05	329663	107123	1	risk diff	7.53E-03
rs2584662	rs2671657	17:47473616_A_G	Overall health rating	-	UKBB	European	-0.007	0.002	3.57E-05	336020	0	1	-	7.78E-03
rs2584662	rs2671659	17:47474529_A_G	Asthma	EFO_0000270	UKBB	European	-0.006	0.001	3.33E-12	336782	38791	1	risk diff	3.76E-09
rs2584662	rs2671659	17:47474529_A_G	Self-reported asthma	EFO_0000270	UKBB	European	-0.005	0.001	2.15E-11	337159	39049	1	risk diff	2.10E-08
rs2584662	rs2671659	17:47474529_A_G	Eosinophil count	EFO_0004586	27863252	European	-0.025	0.004	4.39E-11	173480	0	2	IVNT	3.99E-08
rs2584662	rs2671659	17:47474529_A_G	Sum eosinophil basophil counts	EFO_0004586	27863252	European	-0.025	0.004	5.44E-11	173480	0	2	IVNT	4.84E-08
rs2584662	rs2671659	17:47474529_A_G	Eosinophil percentage of white cells	EFO_0004586	27863252	European	-0.023	0.004	4.17E-10	173480	0	2	IVNT	3.10E-07
rs2584662	rs2671659	17:47474529_A_G	No blood clot, bronchitis, emphysema, asthma, rhinitis, eczema or allergy diagnosed by doctor	-	UKBB	European	0.007	0.001	7.61E-10	336782	228530	1	risk diff	5.33E-07
rs2584662	rs2671659	17:47474529_A_G	Asthma	EFO_0000270	29273806	European	-0.083	0.014	3.70E-09	127669	19954	56	log OR	2.06E-06
rs2584662	rs2671659	17:47474529_A_G	Eosinophil percentage of granulocytes	EFO_0004586	27863252	European	-0.022	0.004	3.93E-09	173480	0	2	IVNT	2.16E-06
rs2584662	rs2671659	17:47474529_A_G	Neutrophil percentage of granulocytes	EFO_0004586	27863252	European	0.022	0.004	5.06E-09	173480	0	2	IVNT	2.70E-06
rs2584662	rs2671659	17:47474529_A_G	Asthma	EFO_0000270	29273806	Mixed	-0.078	0.014	9.58E-09	142486	23948	66	log OR	4.89E-06
rs2584662	rs2671659	17:47474529_A_G	Vascular or heart problems diagnosed by doctor: none of the above	-	UKBB	European	0.006	0.001	3.47E-08	336683	236530	1	risk diff	1.59E-05
rs2584662	rs2671659	17:47474529_A_G	Wheeze or whistling in the chest in last year	EFO_0009715	UKBB	European	-0.005	0.001	3.15E-07	331257	68531	1	risk diff	1.18E-04
rs2584662	rs2671659	17:47474529_A_G	Number of treatments or medications taken	EFO_0007056	UKBB	European	-0.010	0.002	6.95E-07	337159	0	1	-	2.38E-04
rs2584662	rs2671659	17:47474529_A_G	Comparative body size at age 10	-	UKBB	European	-0.009	0.002	8.56E-07	331693	0	1	-	2.89E-04
rs2584662	rs2671659	17:47474529_A_G	Hayfever, allergic rhinitis or eczema	EFO_0003956; EFO_0005854; EFO_0000274	UKBB	European	-0.005	0.001	9.19E-07	336782	77891	1	risk diff	3.05E-04
rs2584662	rs2671659	17:47474529_A_G	Atopic dermatitis	EFO_0000274	26482879	Mixed	-0.092	0.019	1.01E-06	103066	18900	22	log OR	3.30E-04
rs2584662	rs2671659	17:47474529_A_G	Impedance of whole body	-	UKBB	European	0.009	0.002	2.36E-06	331284	0	1	IVNT	7.14E-04
rs2584662	rs2671659	17:47474529_A_G	Coronary artery disease	EFO_0000378; EFO_0001645	29212778	Mixed	-0.028	0.006	2.65E-06	547261	122733	2	log OR	7.96E-04
rs2584662	rs2671659	17:47474529_A_G	Impedance of leg left	-	UKBB	European	0.011	0.002	5.05E-06	331296	0	1	IVNT	1.42E-03
rs2584662	rs2671659	17:47474529_A_G	Vascular or heart problems diagnosed by doctor: high blood pressure	EFO_0004325	UKBB	European	-0.005	0.001	6.43E-06	336683	91033	1	risk diff	1.76E-03
rs2584662	rs2671659	17:47474529_A_G	Impedance of leg right	-	UKBB	European	0.010	0.002	9.26E-06	331301	0	1	IVNT	2.42E-03
rs2584662	rs2671659	17:47474529_A_G	Taking other prescription medications	EFO_0007056	UKBB	European	-0.006	0.001	9.28E-06	336330	158113	1	risk diff	2.42E-03
rs2584662	rs2671659	17:47474529_A_G	Coronary artery disease	EFO_0000378; EFO_0001645	29212778	Mixed	-0.032	0.007	1.30E-05	296525	34541	1	log OR	3.23E-03
rs2584662	rs2671659	17:47474529_A_G	Self-reported hypertension	EFO_0000537	UKBB	European	-0.005	0.001	2.82E-05	337159	87690	1	risk diff	6.41E-03
rs2584662	rs2671659	17:47474529_A_G	Medication for cholesterol, blood pressure or diabetes: blood pressure medication	EFO_0007056	UKBB	European	-0.007	0.002	3.10E-05	154702	38548	1	risk diff	6.88E-03
rs2584662	rs2671659	17:47474529_A_G	Mitral annular calcification	EFO_0005262	23388002	Mixed	NA	NA	3.15E-05	3795	568	2	log OR	6.96E-03
rs2584662	rs2671659	17:47474529_A_G	Mitral annular calcium	EFO_0005262	23388002	Mixed	NA	NA	3.15E-05	6942	-	-	-	6.97E-03
rs2584662	rs2671659	17:47474529_A_G	Number of self-reported non-cancer illnesses	EFO_0009801	UKBB	European	-0.009	0.002	3.27E-05	337159	0	1	-	7.20E-03

e-Table 12: Results of bivariate LD score regression between ACO, asthma, and FEV/FVC, plus ACO and autoimmune and smoking traits
ACO=asthma-COPD overlap; FEV1/FVC (meta) = forced expiratory volume in 1 sec to forced vital capacity ratio in meta-analysis of UK Biobank and SpiroMeta (N=400,102); rg=genetic correlation, SE=standard error; P=p-value

Trait 1	Trait 2	PMID	Ethnicity	LD-Hub Notes	rg	SE	P
**Calculated by authors**							
Asthma	Asthma (moderate-to-severe)	29273806/30552067	European		0.798	0.071	9.32E-30
Asthma	FEV1/FVC (meta)	29273806/30804560	European		-0.333	0.035	1.04E-21
Asthma (moderate-to-severe)	FEV1/FVC (meta)	30552067/30804560	European		-0.338	0.037	1.39E-19
Asthma	Eosinophils	29273806/27863252	European		0.371	0.072	3.15E-07
Asthma (moderate-to-severe)	Eosinophils	30552067/27863252	European		0.374	0.066	1.21E-08
FEV1/FVC (meta)	Eosinophils	30804560/27863252	European		-0.070	0.022	0.0016
COPD	FEV1/FVC (meta)	30804561/30804560	European		-0.842	0.014	0
COPD	Asthma	30804561/29273806	European		0.405	0.042	7.18E-22
COPD	Asthma (severe)	30804561/30552067	European		0.466	0.045	1.79E-25
COPD	Eosinophils	30804561/27863252	European		0.130	0.029	4.83E-06
ACO	Eosinophils	27863252	European		0.292	0.044	4.87E-11
ACO	Asthma (moderate-to-severe)	30552067	European		0.833	0.055	2.81E-52
ACO	FEV1/FVC (meta)	30804560	European		-0.692	0.027	1.71E-140
ACO	COPD	30804561	European		0.828	0.022	3.19E-299
ACO	Asthma	29273806	European		0.743	0.053	6.18E-44
**LD-Hub: autoimmune category**							
ACO	Asthma	17611496	European		0.778	0.087	4.54E-19
ACO	Eczema	26482879	Mixed	Caution: using this data may yield less robust results due to minor departure of the LD structure	0.255	0.088	3.70E-03

Trait 1	Trait 2	PMID	Ethnicity	LD-Hub Notes	rg	SE	P
ACO	Multiple sclerosis	21833088	European	Caution: using this data may yield results outside bounds due to relative low Z score of the SNP heritability of the trait	0.323	0.127	1.10E-02
ACO	Primary biliary cirrhosis	26394269	European		0.107	0.091	2.42E-01
ACO	Primary sclerosing cholangitis	27992413	Mixed	Caution: using this data may yield results outside bounds due to relative low Z score of the SNP heritability of the trait	0.074	0.078	3.42E-01
ACO	Ulcerative colitis	26192919	European		0.048	0.058	4.12E-01
ACO	Inflammatory Bowel Disease (Euro)	26192919	European		0.038	0.050	4.54E-01
ACO	Celiac disease	20190752	European		0.048	0.069	4.85E-01
ACO	Systemic lupus erythematosus	26502338	European		0.038	0.072	5.99E-01
ACO	Crohns disease	26192919	European		0.027	0.053	6.09E-01
ACO	Rheumatoid Arthritis	24390342	European		0.009	0.056	8.80E-01
**LD-Hub: UKBB category**							
ACO	Medication for cholesterol_blood pressure or diabetes: Insulin	NA	European		0.158	0.125	2.04E-01
ACO	Non-cancer illness code_self-reported: ankylosing spondylitis	NA	European		0.091	0.167	5.86E-01
ACO	Non-cancer illness code_self-reported: hyperthyroidism/thyrotoxicosis	NA	European		0.033	0.087	7.02E-01
ACO	Non-cancer illness code_self-reported: hypothyroidism/myxoedema	NA	European		0.015	0.045	7.32E-01
ACO	Non-cancer illness code_self-reported: psoriasis	NA	European		-0.031	0.092	7.34E-01
**LD-Hub: smoking behaviour category**							
ACO	Ever vs never smoked	20418890	European		0.046	0.057	4.17E-01
